# Cell type transcriptomic modules reveal shared molecular mechanisms in Alzheimer’s and Parkinson’s disease

**DOI:** 10.1093/gigascience/giag059

**Published:** 2026-05-21

**Authors:** Anwesha Bhattacharya, Edward A Fon, Alain Dagher, Yasser Iturria-Medina, Jo Anne Stratton, Chloe Savignac, Jack Stanley, Liam Hodgson, Badr Ait Hammou, David A Bennett, Danilo Bzdok

**Affiliations:** Department of Biological and Biomedical Engineering, McGill University, 3775, rue University, Montréal, QC H3A 2B4, Canada; Mila - Quebec Artificial Intelligence Institute, 6666 Saint-Urbain Street, Montréal H2S 3H1, Canada; The Neuro, Montreal Neurological Institute (MNI), McGill University, 3801 Rue University, Ville-Marie, Montreal, QC H3A 2B4, Canada; Department of Neurology and Neurosurgery, MNI, McGill University, 3801 Rue University, Ville-Marie, Montréal, QC H3A 2B4, Canada; Department of Psychology, MNI, McGill University, 2001 McGill College Ave, Montreal H3A 1G1, Canada; McConnell Brain Imaging Centre (BIC), MNI, 3801 University Street, Montreal H3A 2B4, Canada; Department of Neurology and Neurosurgery, MNI, McGill University, 3801 Rue University, Ville-Marie, Montréal, QC H3A 2B4, Canada; McConnell Brain Imaging Centre (BIC), MNI, 3801 University Street, Montreal H3A 2B4, Canada; Ludmer Centre for Neuroinformatics and Mental Health, 2001, McGill College Ave, suite 1310, Montreal H3A 1G1, Canada; Department of Neurology and Neurosurgery, MNI, McGill University, 3801 Rue University, Ville-Marie, Montréal, QC H3A 2B4, Canada; Department of Biological and Biomedical Engineering, McGill University, 3775, rue University, Montréal, QC H3A 2B4, Canada; Mila - Quebec Artificial Intelligence Institute, 6666 Saint-Urbain Street, Montréal H2S 3H1, Canada; The Neuro, Montreal Neurological Institute (MNI), McGill University, 3801 Rue University, Ville-Marie, Montreal, QC H3A 2B4, Canada; Mila - Quebec Artificial Intelligence Institute, 6666 Saint-Urbain Street, Montréal H2S 3H1, Canada; The Neuro, Montreal Neurological Institute (MNI), McGill University, 3801 Rue University, Ville-Marie, Montreal, QC H3A 2B4, Canada; Quantitative Life Sciences, McGill University, 550 Sherbrooke West, Montreal H3A 1E3, Canada; Mila - Quebec Artificial Intelligence Institute, 6666 Saint-Urbain Street, Montréal H2S 3H1, Canada; The Neuro, Montreal Neurological Institute (MNI), McGill University, 3801 Rue University, Ville-Marie, Montreal, QC H3A 2B4, Canada; School of Computer Science, McGill University, 3480 University Street, Montreal H3A 0E9, Canada; Department of Biological and Biomedical Engineering, McGill University, 3775, rue University, Montréal, QC H3A 2B4, Canada; Mila - Quebec Artificial Intelligence Institute, 6666 Saint-Urbain Street, Montréal H2S 3H1, Canada; The Neuro, Montreal Neurological Institute (MNI), McGill University, 3801 Rue University, Ville-Marie, Montreal, QC H3A 2B4, Canada; Rush Alzheimer’s Disease Center, Rush University Medical Center, 1750 West Harrison Street, Suite 1000, Chicago, IL 60612, USA; Department of Biological and Biomedical Engineering, McGill University, 3775, rue University, Montréal, QC H3A 2B4, Canada; Mila - Quebec Artificial Intelligence Institute, 6666 Saint-Urbain Street, Montréal H2S 3H1, Canada; The Neuro, Montreal Neurological Institute (MNI), McGill University, 3801 Rue University, Ville-Marie, Montreal, QC H3A 2B4, Canada; School of Computer Science, McGill University, 3480 University Street, Montreal H3A 0E9, Canada

**Keywords:** latent gene programs, latent gene programs, cytoskeleton dynamics, heavy metal processing, synapse pruning, cross-disorder analysis

## Abstract

**Background:**

Historically, Alzheimer’s disease (AD) and Parkinson’s disease (PD) have been investigated as 2 distinct disorders of the brain. However, a few similarities in neuropathology and clinical symptoms have been documented over the years. Traditional single-gene centric studies, such as differential gene expression analyses, have struggled to unravel the molecular basis for the observed pathological links between AD and PD.

**Results:**

We tailor a latent factor framework to analyze synchronous gene co-expression changes in AD or PD at sub-cell-type resolution. Utilizing large, single-nucleus transcriptomics datasets in AD (70,634 nuclei) and PD (340,902 nuclei) from postmortem human brains, we systematically extract and juxtapose disease-critical molecular signatures in the brain. Our transcriptomic analysis reveals shared molecular programs between AD and PD that localize to specific glial and neuronal cell types. In neurons, convergent gene groups in AD and PD relate to cytoskeletal dynamics and mitochondrial stress mechanisms. In microglia, overlapping gene modules implicate T cell activation mechanisms and synapse pruning pathways. In parallel, AD- and PD-associated gene groups in astrocytes are involved in heavy metal processing; oligodendrocytes highlight convergent dysregulation in myelin synthesis. Additionally, our analysis reveals apolipoprotein E gene (an AD risk gene), and the synuclein alpha gene (a PD risk gene) to have disease predictive roles in both AD- and PD-associated gene modules.

**Conclusion:**

Our multi-module sub-cell-type approach offers novel insights into the molecular basis of shared neuropathology in AD and PD.

## Introduction

Alzheimer’s disease (AD) and Parkinson’s disease (PD) are 2 of the most prevalent disorders in today’s aging societies [1, 2]. There has been intensive research with the grand aim of altering and ultimately halting the course of these diseases. Despite educated forecasts predicting significant advances by this decade [3], AD and PD remain challenging to unravel. This difficulty is compounded by a historically entrenched dichotomy that has limited transfer of research insights from one disease to the other. AD and PD are considered distinct entities due to differences in primary brain regions affected [4–7], age of onset, clinical progression, and treatment response. PD is notably responsive to therapeutics that do not affect cognition [8], and AD is without any “hard-currency” therapeutic to date [9].

This dichotomy between AD and PD continues to be reinforced by genomics and polygenic risk studies, which show minimal to no overlap of genes between AD and PD [10, 11]. Indeed, aggregating prior findings, a *Neuron* review recently concluded, “There is intriguingly little overlap between the risk genes for AD and PD, providing genetic evidence for different disease onset and progression mechanisms ” [12].

By contrast, autopsy examinations show that over half of PD patients have aggregates of tau [13] and around 30% of PD patients develop cognitive impairment, with many going on to dementia [14]. Conversely, AD pathology and Lewy bodies co-occur more frequently than by chance, and Lewy bodies are associated with cognitive decline [7, 15, 16]. Further, neurofibrillary tangles in the substantia nigra can give rise to symptoms of Parkinsonism [17]. Together, these observations raise the possibility of shared disease mechanisms underlying the pathology of AD and PD [18, 19].

More broadly, recent systems-level analysis further suggests that AD and PD share overlapping biological pathways [18]. Proposed mechanisms include mitochondrial dysfunction, neuroinflammation, and dysregulated protein homeostasis [20]. Involvement of these processes has also been suggested in several other neurodegenerative disorders, reinforcing the view that neurodegenerative mechanisms may arise from partially overlapping manifestations of shared molecular networks rather than completely independent disease mechanisms [19, 21].

Despite this evidence, most genomics studies directly questioning the genetic and molecular basis of AD and PD overlap have not identified clear underlying mechanisms. Methodologically, these studies have largely relied on univariate approaches that focus on contribution of individual genes to diease [22–25]. In reality, however, gene expression occurs within tightly regulated environments where gene products interact in highly combinatorial ways [26–29]. Thus, the pathogenesis of neurodegenerative diseases is likely driven by dysregulation of gene networks rather than by isolated gene anomalies [30–32].

Moreover, the effects of dysregulated genes are not identical across different cell types. Large-scale single-nucleus transcriptomic studies in AD [33, 34], and PD [4, 35], have reported disease-associated transcriptional changes that are highly cell type-specific. For example, in AD, APOE expression is increased in microglia and decreased in astrocytes and oligodendrocyte precursor cells (OPCs) [36, 37]. Given such complexity at the cellular level, it is crucial to account for cell type heterogeneity when comparing AD- and PD-related changes. This is evidenced by the minimal AD-PD overlap derived from analysis of bulk RNA sequencing data [38]. Recent advances in single-nucleus RNA sequencing (snRNA-seq) have enabled the resolution of transcriptional signatures with high cell-type specificity. However, in contrast to recent proteomics- and CSF-based AD-PD comparative analyses [39], no single transcriptomics study has employed multivariate approaches at the single-cell resolution to investigate AD and PD simultaneously at scale.

In this present investigation, we systematically revisited the problem of identifying candidate molecular mechanisms that overlap between AD and PD through the lens of transcriptomics. Comprehensive snRNA-seq datasets from AD (70,634 nuclei from 48 postmortem brains) [36] and PD (340,902 nuclei from 15 postmortem brains) [40] allowed us to leverage advanced machine learning techniques for our analysis [41]. Enabled by a supervised multivariate model [42], we extracted biologically interpretable and diease relevant gene modules at sub-cell type granularity from the AD and PD transcriptomes (16,936 protein-coding transcripts). By linking our gene modules to curated biological pathways, we identified shared candidate mechanisms of neurodegeneration. In addition, we validated our main findings and conclusions using an independent AD-PD snRNA-seq dataset pair. Overall, our supervised pattern learning-based comparative approach provided a statistically rigorous and unified framework for the automatic discovery and comparison of disease-associated molecular signatures across disorders.

## Results

### Rationale

Traditional approaches in single-cell transcriptomics, such as DGE, typically focus on individual genes in isolation. This offers a fragmented view of disease-related transcriptional changes. Recent studies employing univariate frameworks to identify disease-associated genes have reported modest overlap in AD- and PD-relevant genes, primarily limited to glial populations [43]. However, we hypothesized that such methods may fail to capture co-regulated molecular alterations in AD and PD. Further, these molecular changes likley have a cell type-specific component, which are missed by bulk sequencing methods. In addition, we reasoned that disease progression within each cell type was not driven by a single molecular axis. Rather, multiple distinct transcriptional programs were altered. Therefore, a biologically coherent AD and PD comparison would entail examining multiple gene programs within distinct cell types to capture the full complexity of their molecular convergence.

To address these dimensions, here we interrogated AD- and PD-transcriptional changes using gene modules representing coordinated gene activity across the transcriptome. We extracted disease-relevant gene modules using a supervised latent factor modeling framework, previously validated in AD [42]. This method combined latent structure discovery strength while simultaneously being aware of valuable contextual information—the disease state. Using these discovered gene modules, we a rigorous cross-disorder comparative framework to study AD- and PD-associated molecular changes.

Notably, by examining the entire recorded transcriptome, our data-driven pipeline enabled an unbiased assessment of genes, without prior assumptions about AD- or PD-association. In addition, our approach could assign a gene as being relevant to multiple gene modules within a given cell type—a feature absent from many previous gene network analysis techniques [31]. Overall, our multivariate analysis is an unbiased investigator of the transcriptomic landscape of AD and PD related molecular mechanisms.

### Disease predictive, cell type-specific gene modules identified using latent factor modeling

We explored the possibility of molecular overlap in AD and PD brains through transcriptional alterations captured in computationally derived gene modules (groups of co-expressed genes). The main analyses were conducted on 2 snRNA-seq datasets—Religious Orders Study and Memory and Aging Project (ROSMAP)-AD [36] (70,634 nuclei across 8 major cell types; the “Methods” section) and Kamath-PD [40] (340,902 nuclei across 11 cell types). Our analytical framework employed partial least squares discriminant analysis (PLS-DA) to gain an overview of 16,936 protein-coding genes (see the “Methods” section).

**Figure 1:**
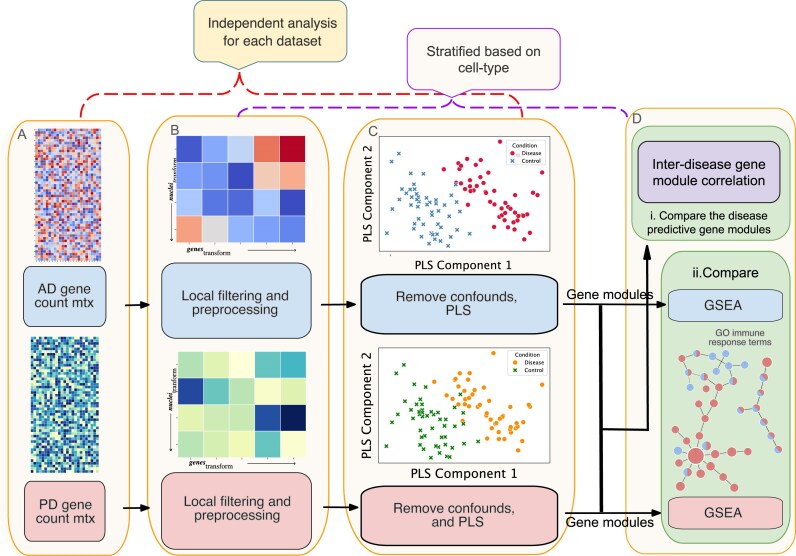
Workflow diagram to test for AD-PD overlap: bottom-up approach. Overview of workflow. (A) Single nucleus RNA sequencing (snRNA-seq) datasets for AD and PD were downloaded from public databases. Filtering, quality control, and cell type annotations were performed by source authors. (B) Preprocessing step. Performed independently for each cell type, this step followed recommended guidelines for data transformation, removed lowly expressed genes, and corrected for disease versus control class imbalance. (C) Gene module identification step. PLS-DA was performed per cell type to extract weighted gene lists, referred to as gene modules, that were disease predictive. (D) Comparative analysis. We aggregated the results from the 2 analysis arms and assessed overlap using parallel methods—(i) direct correlation of cross-disease gene module pairs and (ii) gene set enrichment analysis to identify overlapping biological processes, molecular functions, and cellular components. We discover significant molecular similarities between AD and PD across cell types. PLS, partial least squares; AD, Alzheimer’s disease; PD, Parkinson’s disease; GSEA, gene set enrichment analysis; GO, gene ontology.

**Figure 2:**
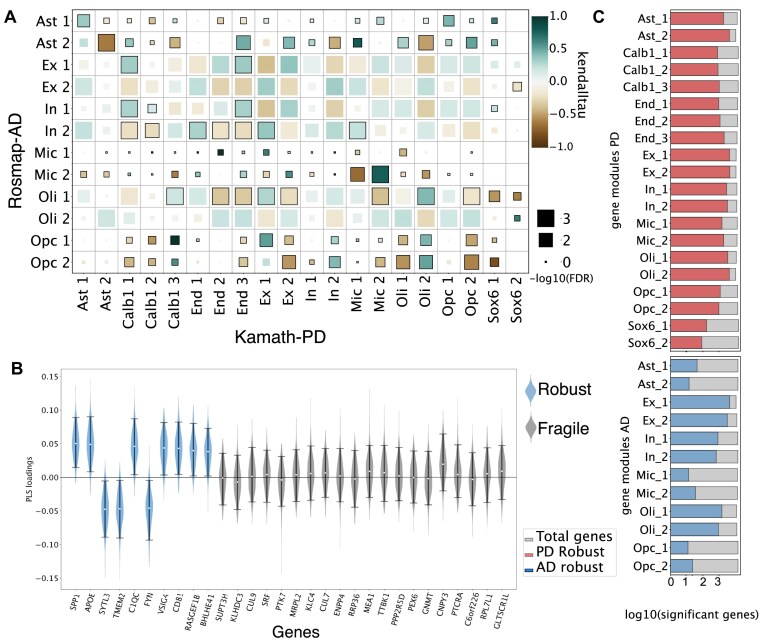
Convergence of transcriptomic signatures in Alzheimer’s and Parkinson’s disease across cell types when zoomed in on disease-predictive gene modules. We probed 2 snRNA-seq datasets (ROSMAP-AD, Kamath-PD) to explore the transcriptomic overlap between AD and PD. By training 15 PLS models (one for each cell type in AD (6) or PD (9)), we extracted latent representations of gene expression (gene modules) that maximized the separation between disease and control nuclei. (A) The colored squares represent Kendall’s tau-b (τ_b_), quantifying the degree of association between AD and PD gene modules. Darker colors represent stronger genetic associations, indicating similar ranking trends of robust genes in the module pair. Highlighted boxes with borders denote statistically significant τ_b_ exceeding 2.5/97.5% CIs based on label-shuffled permutation tests (*n* = 1,000). Square size is proportional to FDR-corrected statistical significance of overlap. The wide range of correlation strengths suggests that AD and PD share significant molecular similarity spanning the cell type landscape. (B) An illustration of the robustness assessment of genes in a gene module. The 30 genes with the highest empirical predictive weights from the first PLS component in ROSMAP-AD microglia are shown. Violins represent the loading distribution of genes derived from a BS resampling scheme (*n* = 500). Robust genes have non-zero predictive weights (2.5/97.5% CIs). Genes that do not meet this criterion are grayed out. APOE, a gene strongly associated with AD, was highlighted as a major disease predictor in this component. (C) The colored portions of the bars represent the number of genes with robust disease-predictive weights for each gene module. The gray regions denote the total number of analyzed genes for the cell type. Robust genes were assessed based on a 500-iteration BS resampling scheme. Ast, astrocyte; Ex, excitatory neuron; In, inhibitory neuron; Mic, microglia; Oli, oligodendrocyte; OPC, oligodendrocyte precursor cell; PLS, partial least squares.

In either AD or PD, we fitted cell-type-level PLS_cell_ models across nuclei from all donors in the dataset (Fig. 1; the “Methods” section). This yielded gene modules as latent projections of gene expression matrices that assisted in distinguishing cells of patients from controls. Comparative assessments of these thus-derived gene modules highlighted shared molecular mechanisms between AD and PD that were more stable than expected by chance (Fig. 2A).

As a first modelling step, we independently analyzed the transcriptomes of AD and PD to identify disease- and cell type-specific modeling hyperparameters. Specifically, the optimal number of gene modules that best distinguished diseased from healthy cells (in unseen cells) was selected using a 10-fold nested cross-validation (CV) scheme (see the “Methods” section). In ROSMAP-AD, 2 gene modules per cell type were determined to be optimal for all 6 cell categories. In Kamath-PD, the optimal number of gene modules was determined to be 2 for each major cell type, except endothelial cells (3 modules) and CALB1 dopaminergic neurons (3 modules).

The fitted cell type specific PLS_cell_ models (6 AD and 9 PD models; optimal hyperparameters) exhibited robust above-chance out-of-sample accuracy in differentiating disease samples from control (Fig. S1A). Unbiased classification accuracy was estimated based on a patient-partitioned CV scheme (see the “Methods” section). Specifically, in the AD versus control group contrast, the predictive power measured by AUROC ranged highest for microglia (AUROC: 0.66 ± 0.06 std across partitions) to lowest for OPCs (0.56 ± 0.08 std across partitions). For the PD versus control group contrast, the highest AUROC was for endothelial cells (0.89 ± 0.13 std across partitions), and the lowest was for excitatory neurons (0.69 ± 0.35 std across partitions). These observations strongly supported the role of gene modules in directly informing the disease phenotype across all examined cell types and conditions.

Our derived gene modules (PLS_cell_ components) thus featured a combination of several genes whose co-expression signature was associated with a disease state (AD versus control or PD versus control). We assessed the statistical significance of each module by comparing its empirical disease prediction performance to a null distribution of performance metrics derived by a label-shuffling permutation procedure (the “Methods” section). Only significant gene modules were considered for subsequent analyses (empirical module *ρ* > 97.5th percentile of permutation derived *ρ* distribution; Fig. S1B). In particular, 2 cell types in ROSMAP-AD, pericytes and ependymal cells, did not pass our significance test and were removed from further analysis. Two cell types in Kamath-PD, macrophages and ependymal cells, were similarly removed.

We identified stable genes within each PLS_cell_ gene module (12 AD modules, 20 PD modules). Specifically, using a bootstrap (BS) resampling technique (see the “Methods” section), we assessed which gene effects were statistically robust (zero not included in the 2.5/97.5% confidence interval (CI) of the BS distribution of each gene), and thus, reliably affected prediction outcomes. As an illustration, we visualized the PLS_Mic_ loadings for the first gene module from ROSMAP-AD microglial cells (Fig. 2B). Overall, each gene module yielded a variable set of robust genes distributed across the transcriptome (Fig. 2C).

We further investigated the characteristics of the derived modules using a clustering algorithm. For a cell type, we assigned each observed nucleus to exactly one of its modules based on the component harboring the maximum PLS_cell_ score. In doing so, we were able to visualize the distribution of the gene modules assigned to the nuclei in a 2-dimensional embedding space (Fig. S2; PHATE embedding space; the “Methods” section). No clear clustering among the components was observed suggesting that our gene modules did not necessarily correspond to cellular subtypes. Instead, they likely corresponded more closely with different functional programs within a given major cell type. That is, any given cell belonging to a type could exhibit several of our distinct gene programs to various continuous degrees.

In a stringent external validation analysis using untouched datasets, we derived independent AD and PD gene modules in 2 additional snRNA-seq datasets—Seattle-AD and Smajić-PD (detailed cohort and sample description in the “Methods” section). We repeated all main analyses, from scratch, and derived sub-cell-level disease predictive gene modules from these datasets (Fig. S3B; the “Methods” section). In Seattle-AD, 15 significant gene modules emerged across 10 examined cell types. Independently, in Smajić-PD, 25 significant gene modules emerged across 7 cell types. Significance of modules was determined, as before, using a label-shuffle permutation test. These independently derived gene modules were subsequently used to corroborate our findings from the primary AD-PD overlap analysis (see next section).

We assessed whether the latent gene modules were influenced by potential demographic variables. Concretely, we examined the contribution of age and sex to variation in module scores, quantified by the proportion of variance explained using a linear regression model (the “Methods” section). Across gene modules, the contribution of sex toward explaining the variance in module scores was low within each dataset (mean ± standard deviation across modules): ROSMAP-AD, *R*^2^ = 0.01 ± 0.01; Kamath-PD, *R*^2^ = 0.07 ± 0.09; Seattle-AD, *R*^2^ = 0.017 ± 0.018; Smajić-PD, *R*^2^ = 0.12 ± 0.10. Similarly, age explained little variance in module scores: ROSMAP-AD, *R*^2^ = 0.003 ± 0.006; Kamath-PD, *R*^2^ = 0.04 ± 0.08; Seattle-AD, *R*^2^ = 0.018 ± 0.020; Smajić-PD, *R*^2^ = 0.04 ± 0.06. Overall, across all gene modules from the 4 datasets, age and sex explained only a small fraction of the variance in module scores relative to the variance captured by diagnosis, the primary variable of interest (Table S10).

Importantly, in all analyses so far, the gene expression samples from AD and PD were not merged. Instead, these were conducted in parallel with independent supervision targets (AD-control or PD-control). Thus, we identified robust AD- and PD-predictive gene modules in a cell type-specific manner. These thus derived modules, specific to AD or PD, enabled subsequent comparative analyses aimed at examining the shared molecular alterations between AD and PD.

### Shared molecular signatures uncovered between AD and PD, across major brain cell types

We subsequently moved to our comparative analysis between AD and PD. To quantify the coupling between AD- and PD-derived gene modules, we used Kendall’s tau-b (τ_b_) metric ( the “Methods” section). This calculated the degree of similarity between 2 vectors of PLS_cell_ predictive weights of the overlapping robust genes (not the gene expression measurements) from an AD-PD gene module pair. By comparing the empirical correlation strength with a permutation-derived null distribution (obtained by correlating permutation modules derived from a label-shuffled dataset), we identified the module pairs with significant associations (empirical τ_b_ more extreme than 2.5/97.5% CI of the null distribution). Notably, we reported only those associations that survived this exhaustive permutation-based validation.

As the most important results of our investigation so far, we noted strong and robust associations across several AD-PD module pairs (robust to 1,000 iterations label shuffle permutation test; τ_b_ false discovery rate (FDR) *q*-value < 0.05; Fig. 2A; Table S1; the “Methods” section). Across all pairwise combinations, the most significant correlation (smallest *q*-value) emerged between the first oligodendrocyte module (represented as Oli 1) from ROSMAP-AD and the second oligodendrocyte module (Oli 2) from Kamath-PD (represented as Oli 1_Oli 2; τ_b, abs_ = 0.44, number of shared genes = 1,351, *q* = 5.4e-128). In addition to Oli 1_Oli 2, other top significant pairs included Oli 1_Ex 1, Oli 1_Ex 2, In 2_Ex 1 (exhibited τ_b, abs_ > 0.3, and shared over 500 robust genes).

In contrast, the strongest associations (ranked by absolute τ_b_) were observed between glial modules—astrocytes, microglia, and OPCs. Specifically, the highest association strength was observed between Mic 2_Mic 1 (τ_b, abs_= 0.67; *q* = 0.009), followed by Ast 2_Ast 2 (τ_b, abs_ = 0.62; *q* = 4.2e-5). These results suggest that in glial cells, disease associated molecular programs are broadly shared between AD and PD.

On the flipside, inter-neuron module pairs (Ex and In pairs) between AD and PD showed the least overlap, across all module pairs. However, dopaminergic neurons in PD—CALB1, and SOX6—showed significant similarities with AD-critical gene programs in neurons (Ex 1_Calb1 1, τ_b, abs_ = 0.35, *q* = 3.1e-20; In 1_Calb1 1, τ_b, abs_ = 0.32, *q* = 1e-5), along with AD-associated oligodendrocyte and astrocyte modules (Oli 1_Calb1 3, τ_b, abs_ = 0.19, *q* = 8.21e-5). Thus, in contrast to the major recorded PD neuronal populations (Ex and In), disease-critical PD-neurons (highlighted in original study [40]) had significant molecular similarities with several AD-associated molecular programs.

As a sanity check, we assessed how the cross-cell type module associations compared within a single disease. Specifically, we looked at the correlation effect sizes (statistically significant under a label shuffled permutation test, *q* < 0.05; the “Methods” section) across gene modules in a ROSMAP-AD versus ROSMAP-AD comparison, and a Kamath-PD versus Kamath-PD comparison (Fig. S1D). The presence of robust association signatures between gene modules from different cell types within the same disease supports the cross-cell type module-level overlaps observed between AD and PD.

We validated our primary findings by examining molecular similarity between Seattle-AD- and Smajić-PD-derived gene modules. Our analysis revealed significant module-level overlaps between AD and PD, scattered across different cell types (Fig. S3A; Table S2). Across all modules, inter- and intra-oligodendrocyte module pairs from AD and PD took center stage, aligning with our primary analysis. The strongest association was observed between oligodendrocytes from Seattle-AD and Smajić-PD (Oli 1_Oli 1, τ_b, abs_= 0.51, *q* = 5.5e-26), followed closely by Ast 1_Oli 1 (τ_b, abs_= 0.26, *q* = 9.2e-16). Strong significant associations were also observed between different combinations of neuron and glial cell-derived modules (Ast 1_L4_it 1, τ_b, abs_= 0.45, *q* = 1.6e-9; Oli 2_In 3, τ_b, abs_= 0.86; *q* = 0.05). Further, inhibitory neuron-derived modules showed sparse similarities between AD and PD, similar to our observations within the primary datasets. Overall, this external validation of shared transcriptomic signatures between AD and PD indicated that the observed overlaps were unlikely to be driven by dataset-specific factors such as transcriptomic platform, cohort composition, or brain region selection.

We further assessed the generalizability of our comparison model by randomly partitioning the ROSMAP-AD and Kamath-PD datasets into 2 non-overlapping subset pairs (split-half test; see the “Methods” section). We then repeated the full analysis pipeline on each subset and compared the resulting pairwise associations between the subset-derived gene modules. Our analysis revealed strong associations across different realizations of the partitioned, but otherwise identical, analyses (Pearson’s rho = 0.92 ± 0.02 standard deviation; Fig. S1C). This result provided additional quantitative support for the associations from the full datasets, indicating that the observed relationships were robust to sample size.

We assessed the specificity of our findings from the AD-PD comparative analysis with respect to other systemic diseases. Specifically, we performed a comparison of AD- or PD-derived modules with chronic obstructive pulmonary disease (COPD) of the lung (neurological versus non-neurological). COPD was selected as a negative control condition given the expectedly different cellular composition and tissue context relative to the brain. Following our established analytical pipeline, we computed pairwise similarities between AD- and PD-associated gene programs with COPD-associated gene programs across major recorded lung cell populations (AD-Lung, PD-Lung; derived following Fig. 1 pipeline; Tables S5–S8).

Overall, we observed limited overlap between brain and lung disease modules. The modest similarities that were detected primarily involved immune-related cell populations and endothelial cells from the lung and the brain. Specifically, neuronal populations from all 4 AD and PD datasets showed little overlap with the lung gene modules (maximum Mye 1_Ex 1 (Lung_Kamath-PD), τ_b, abs_ = 0.49, FDR *q* = 1.43e-181). In contrast to the neuronal cells from the brain, lung myeloid (Mye) and lymphoid (Lymp) cells showing significant overlap with brain glial cells (maximum Lymp 1_Mic 1 (Lung_ROSMAP-AD), τ_b, abs_ = 0.74, FDR *q* = 6.4e-4). Taken together, these results suggest that neuronal gene programs identified in AD and PD are largely brain-specific, while a subset of immune-related signatures are shared across tissues and disorders.

Collectively, these findings revealed significant molecular overlap between AD and PD at a sub-cell type resolution. The degree and specificity of these overlaps varied between cell type-specific gene module pairs, with oligodendrocyte and neuron-based gene module combinations in AD and PD signaling the strongest similarities. Our external validation experiments replicated these core findings in independent datasets, further substantiating our conclusions regarding the shared genetic architecture between these neurodegenerative diseases.

### Cell type-specific gene modules reveal GWAS derived genes as key predictors of disease

**Figure 3:**
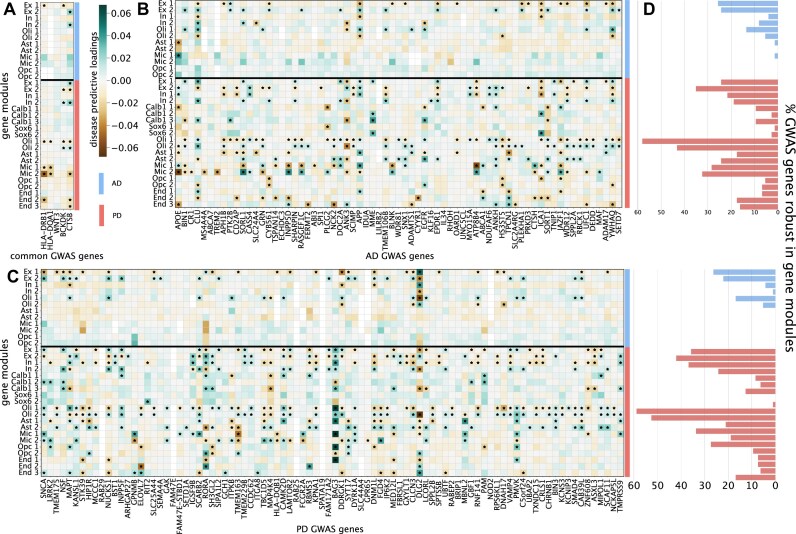
Cell type-specific gene modules reveal GWAS-associated genes as key disease predictors. We mapped the contribution of 164 candidate GWAS-nominated genes, compiled from the largest AD and PD GWAS studies, within our gene modules. Color saturation represents the strength of the predictive weight of a gene in a module as determined by the PLS model. Robust genes are highlighted with black stars, defined as those with BS-derived 2.5/97.5% CIs that do not include zero. (A–C) Genes are grouped based on the nominating disease. Group (A) contains 5 common genes nominated independently in both AD and PD. (B) shows the genes implicated in AD GWAS. (C) shows genes implicated in PD GWAS. (D) The bars on the right summarize the connection of a module with GWAS-mapped genes. The lengths indicate the percentage of GWAS-mapped genes with robust loadings in the module. Cell type-specific module-level localization of risk genes was observed. APOE, a major AD risk gene, showed strong predictive loading in ROSMAP-AD microglial and astrocyte modules. For PD, SNCA had strong predictive signals in Kamath-PD oligodendrocyte, microglia, excitatory neurons, and CALB1 dopaminergic neuron modules. Several genes that were implicated as being AD-relevant in GWAS had robust predictive loadings in Kamath-PD gene modules and vice versa. For example, APOE, APP, and other AD genes mapped to risk loci had robust predictive weights in Kamath-PD modules. Likewise, SNCA, MAPT, and other PD risk genes had robust predictive weights in ROSMAP-AD modules. Ast, astrocyte; End, endothelial; Per, pericyte; Ex, excitatory neuron; In, inhibitory neuron; Mic, microglia; Oli, oligodendrocyte; OPC, oligodendrocyte precursor cell; GWAS, genome-wide association study.

We contextualized our gene modules post hoc to understand their relationship with known risk genes from genomic studies. Drawing from the most recent GWAS that reported AD [44] or PD [45], we investigated 164 genes (Table S3; see the “Methods” section), locating them within our gene modules. Most genes implicated by major GWAS risk loci (here refered to as GWAS genes) showed robust disease-predictive loadings in at least one gene module (Fig. 3). The top gene module harboring the most AD GWAS genes was in AD Ex 1, with 25.6% of AD GWAS genes present, followed by AD Ast 1 with 24.3% GWAS genes present. In PD, the top modules with most PD GWAS genes was Oli 1 with 58.9% PD GWAS genes followed by Ex 2 with 42%. Moreover, we found clear cell type localization of these genes within our modular framework. For example, APOE, a broadly accepted AD risk gene, showed robust predictive loadings in Ast 1 and Mic 1 ROSMAP-AD modules. Similarly, LRRK2, one of the major PD risk genes, was implicated in distinct Kamath-PD gene modules. The strongest effect was observed in PD Mic 2.

Next, we analyzed the effects of GWAS genes from one disease within gene modules linked to the other neurodegenerative disease. In other words, we investigated whether genes mapped to AD GWAS risk loci were highlighted in any PD modules and vice versa. Among the AD GWAS genes, top genes, including APOE, APP, BIN1, and CLU (based on *P*-value from GWAS [44]) had robust disease predictive loadings in gene modules associated with PD (Kamath-PD modules). Concretely, APOE had strong predictive weight in PD Mic 2 (gene loading = −0.06, max absolute loading for any gene in this module was 0.07, absolute rank of this gene = 23), APP in PD Mic 1 (loading = 0.04, max_abs_ = 0.07, rank = 262), BIN1 was found in PD Calb1 2 (loading = −0.02, max_abs_ = 0.04, rank = 1,626), and CLU had the strongest robust weight in PD Oli 2 (loading = 0.03, max_abs_ = 0.08, rank = 405). It is important to note that these genes were not the highest-ranked in the respective PD modules. In other words, they were not the primary disease-indicative genes (rank = 1). Instead, these genes likely played supporting roles that become apparent only within the context of the gene groups.

Conversely, we made similar observations for previously reported genes implicated in PD GWAS studies [46] within our (ROSMAP-)AD modules. The gene enconding synuclein alpha (SNCA) had strong predictive loading in AD Ex 1 (−0.03, max_abs_ = 0.07, rank = 397), microtubule-associated protein tau (MAPT) in AD In 1 (−0.02, max_abs_ = 0.07, rank = 979), and TMEM175 in AD Ex 1 (−0.02, max_abs_ = 0.07, rank = 1,869). Notably, while the genes implicated in AD GWAS had very clear cell type localizations, the GWAS genes associated with PD tended to be distributed across modules derived from multiple cell types, with particularly high effect sizes in neuronal modules. This observation is consistent with a previous genomic enrichment study showing that PD risk loci are not confined to specific cell types. Instead, they are associated with broad cellular processes observable across multiple cell types [47]. Collectively, our observations suggested that the bona fide GWAS genes not only tracked the disease they were implicated in, but also proved relevant in gene modules associated with the other disease.

In our external validation analysis, GWAS-mapped genes exhibited consistently strong disease-predictive loadings in gene modules derived from the Seattle-AD and Smajić-PD datasets (Fig. S4). Notably, all key observations from our primary dataset analysis were replicated. First, the genes tracked disease-corresponding modules; microglia and astrocyte Seattle-AD modules faithfully tracked APOE (interestingly, we observed robust predictive loading for APOE in Seattle-AD OPC modules, consistent with a previous study [36]). Similarly, PD GWAS genes, such as SNCA and LRRK2, were tracked by Smajić-PD gene modules. Second, mirroring our primary dataset pair, several GWAS-mapped genes were enriched in modules associated with the opposite disease (for instance, APOE tracked Smajić-PD modules and SNCA tracked Seattle-AD modules), further reinforcing the presence of cross-disease molecular convergence.

In summary, here we contextualized the suspected genes from AD- or PD-GWAS risk loci within the scope of transcriptome derived gene modules. This led us to identify significant effects of GWAS genes beyond the disease in which they were initially reported. For example, we observed significant impact of APOE not only in AD (microglia and astrocyte-specific modules), but also in PD-derived modules (microglia, astrocytes, and CALB1 DA neurons). These cross-disease results were replicated in independent validation datasets. Crucially, these observations were only possible through our approach, which evaluated the joint contribution of statistically meaningful gene sets to disease status. Thus, for example, although APOE may not show a strong univariate effect in PD, it might play a significant role when considered within the context of co-expressed genes in PD gene modules.

### Overlapping biological functions between AD and PD gene modules

We contextualized the biological relevance of the observed AD-PD overlap using comprehensive gene set enrichment analyses (GSEAs). GSEA was performed independently for each gene module (12 ROSMAP-AD and 20 Kamath-PD). Notably, in our analysis, a single gene could contribute significantly to disease via multiple modules within the same cell type. This enabled us to capture likely gene effects on complementary, co-regulated pathways.

By screening the widely relied upon gene ontology (GO) databases corresponding to 3 complementary domains—biological processes (BPs), molecular functions (MFs), and cellular components (CCs)—we identified AD or PD relevant GO terms. Across all gene modules from ROSMAP-AD, 284 BP, 56 MF, and 102 CC terms were identified. In Kamath-PD, we obtained 715 BP, 152 MF, and 209 CC terms. In total, a universe of 27,993 GO BP, 11,271 GO MF, and 4,039 CC terms was analyzed.

**Figure 4:**
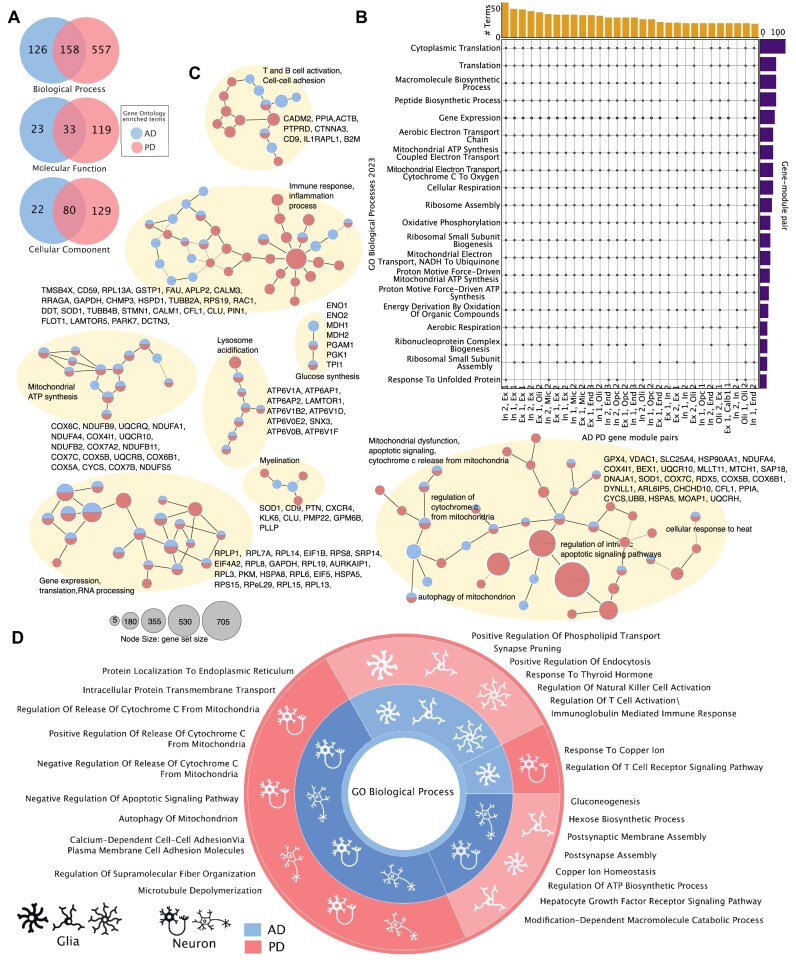
GO terms mapped to gene modules are shared between AD and PD. GSEA results for the derived gene modules. For each gene module derived from ROSMAP-AD or Kamath-PD, we mapped the ranked genes (based on predictive weights) to terms in the GO database. (A) Overlapping terms in GO biological process (BP), cellular component (CC), and molecular function (MF) databases. The Venn diagrams depict the number of unique and shared terms across all gene modules, grouped by AD or PD. (B) Top 30 most frequently shared GO terms in AD-PD gene-module combinations. Solid black dots indicate that a term (vertical axis) is enriched in the corresponding gene-module pair (horizontal axis). Bar plots on the horizontal axes show counts of the total number of shared terms for the gene-module pair. Bar plots on the vertical axis show the total number of cross-disease gene-module pairs in which a term is present in (top 10 terms). (C) Graph visualization of top shared GO biological themes across AD and PD. Nodes are GO terms, and colors represent disease label. Node size indicates the GO-term gene-set size. Group names summarize the main themes from the terms in the group. Top AD-PD shared genes (robust PLS loadings) are annotated within each group. This overview highlights key BPs involved in both AD and PD. (D) Shared GO BP terms between AD and PD that are unique to either neurons or glia. The inner circle denotes the cell type group from AD, while the outer circle denotes the PD group. Darker shades represent terms enriched exclusively in neuronal modules (excitatory and inhibitory neurons, CALB1, SOX6), and lighter shades represent terms enriched exclusively in glial cell modules (microglia, astrocyte, oligodendrocyte, OPC, endothelial cells). BPs related to altered cytoskeleton dynamics, impaired mitochondrial function, and apoptotic signaling are enriched across gene modules from neurons in both AD and PD. Immune response, synapse maintenance, and lipid transport-related terms are enriched in one or more glial cell modules in both AD and PD.

Among all terms across gene modules, 158 BP, 33 MF, and 80 CC terms were shared between AD and PD (Table S4). The relatively small subset of overlapping terms highlighted the non-random and specific nature of our gene modules (Fig. 4A). The greatest number of GO term overlaps emerged in neuron–neuron AD-PD module pairs, along with pairs involving oligodendrocytes and OPCs (>160 terms per module pair, BP, MF, and CC combined; Fig. S5A, note Fig. 4B horizontal axis). Additionally, both AD and PD microglial gene modules shared, on average, 20 terms with other gene modules. In contrast, module pairs involving astrocytes featured lower overlaps, with the maximum number of shared terms being 13 between AD Ex 1 and PD Ast 1.

By sorting all BPs based on their frequency of shared occurrence across AD-PD module pairs, we identified the commonly shared pathways. These were related to protein translation, cellular respiration, and mitochondrial energy synthesis (Fig. 4C). To further summarize the terms systematically, we devised a visualization procedure to obtain a synoptic summary of the overarching BPs (see the “Methods” section). Key shared biological themes emerged between AD and PD (Fig. 4D): protein synthesis and misfolding (top shared genes included RPLP1, RPL7A, RPL14, EIF1B, RPS8, SRP14), immune response (TMSB4X, CD59, RPL13A, GSTP1, FAU, APLP2, CALM3, RRAGA, GAPDH, TUBB2A/4B, RPS19, RAC1, DDT, CALM1, CFL1, CLU), lysosome acidification (ATP6V/6A, LAMTOR1, SNX3), glucose metabolism (ENO1, ENO2, MDH1, MDH2, PGAM1, PGK1, TPI1), mitochondrial dysfunction (COX6C/4I1/7A2/7C/5B, NDUFB9/A1/A4/B2/B11/S5, UQCRQ/10/B, CYCS), and myelination (SOD1, CD9, PTN, CXCR4, KLK6, CLU, PMP22, GPM6B, PLLP).

To investigate cell type localized BPs, we designed a probe to filter out general cellular injury-related pathways. We first categorized the gene modules into 2 groups—neuronal modules and glial modules. Given the fundamental anatomical and functional differences between neurons and glia, these groups can be expected to exhibit distinct responses to disease. The neuronal group included gene modules from excitatory neurons, inhibitory neurons, CALB1, and SOX6 cell types. The glial group comprised astrocytes, microglia, oligodendrocytes, and OPCs. We then removed all GO BP terms that were enriched in both neuronal and glial modules. This resulted in a refined set of GO terms that were exclusive to either neurons or glia (Fig. 4E; cf. Fig. S5B for gene module level grouping). By comparing these terms between AD and PD, we identified neuron and glia specific shared mechanisms of overlap.

Neurons shared the greatest number of exclusive (unique to cell types belonging to this category) GO BP terms between AD and PD. We identified several shared terms associated with microtubule depolymerization and cytoskeleton dynamics between Kamath-PD and ROSMAP-AD neuron modules (PD Calb1 1, Ex 1, Ex 2 and AD In 1, In 2, and Ex 1). Shared genes associated with these terms included MAPT, FKBP4, GBA2, MAP1A, MAP1B, MAP1S, MAP2, MAPRE3, STMN1, STMN2, STMN3, and STMN4. We also observed terms related to mitochondrial release of cytochrome c regulation (PINK1, PRELID1, CLU, BNIP3, DNM1L, GHITM, GPX1, MFF, MLLT11, MOAP1). Specifically, Ex 1 and Ex 2 in Kamath-PD, and In 1 and In 2 in ROSMAP-AD highlighted these terms. Terms related to iron homeostasis were noted in Ex 1 from PD and In 1 and Ex 1 from AD (SOD1, several ATP genes, CCDC115, FTH1, FTL, ISCU, NDFIP1, and SLC22A17).

We observed that glia-exclusive terms had several themes centered around the immune and complement systems along with synapse pruning, lipid transport, metal ion homeostasis, and thyroid hormone (TH) balance. T cell activation pathways were enriched in PD Mic 2, End 2, and AD Mic 2. These modules shared genes including B2M, HLA-A, HLA-B, HLA-C, HLA-DPA1, HLA-DRA, HLA-DRB1, HLA-DRB5, and HLA-E. Lipid and phospholipid transport showed up exclusively in microglial modules in AD Mic 1 and PD Mic 2 (APOE, TSPO, and PRELID1). Response to TH was recorded in PD Mic 2 and AD Mic 1 (CTSB, CTSH).

We also noted a few terms exclusive to opposite categories in AD and PD. For example, “response to copper ion” was identified exclusively in AD glia modules and PD neuron modules. However, functionally related terms, like cellular response to copper ion, copper ion binding, and copper ion homeostasis, were enriched in AD Ast 1, Opc 1, Ex 1, and Ex 2 and in PD Mic 1, End 1, Ex 1, Ex 2, and In 1. A closer inspection of all terms belonging to cross-category modules, AD neuron-PD glia or AD glia-PD neuron suggested that these differences largely reflect the granularity and naming conventions of GO annotations. In contrast, for AD neuron-PD neuron and AD glia-PD glia specific terms were functionally distinct, reflecting meaningful biological differences rather than annotation-related effects. For instance, a manual search for “cytoskeleton” or “microtubule” highlighted only neuronal modules in both AD and PD. These neuron and glia specific overlaps were further supported by replication in independent datasets (Seattle-AD and Smajić-PD).

For validation of these results, we turned to our external dataset pair. Analogous to our first analysis pair, we applied the GSEA pipeline to the gene modules derived from the Seattle-AD and Smajić-PD datasets (Fig. S6A). We confirmed that AD and PD neurons, oligodendrocytes, and OPC modules had the largest occurrence of shared terms (Fig. S6C; Table S9). Moreover, these results aligned with the broad biological themes highlighted from the primary analysis (Fig. S6B)—the most frequent AD and PD shared terms were related to mitochondrial energy metabolism (CD44, HSPA1A, CLU), myelination (SOD1, TENM4, PLP1), and glucose metabolism (APOD, RORA, HSPA5).

Thus, our GSEA successfully identified a variety of matching biological, cellular, and molecular processes across AD and PD. Our observations highlighted key themes that localized to certain cell types in AD and PD. Neurons in AD and PD demonstrated enrichment of terms related to cytoskeleton structural integrity, mitochondrial transport, and mitochondrial energy synthesis. Alternatively, glia-derived gene modules highlighted terms related to several regulatory mechanisms, including synapse pruning, lipid transport, immune, and inflammatory systems.

### Latent gene modules reveal higher cross-disease transcriptomic convergence than traditional differential gene expression

We compared our gene module-based comparative framework to the widely adopted univariate method in RNA-seq—differential gene expression analysis. This method quantifies differences in gene expression between 2 groups by comparing expression profiles in diseased versus neurotypical cell states. We conducted a parallel AD-PD overlap analysis based on differentially expressed genes (DGEs; MAST; the “Methods” section) derived independently in the ROSMAP-AD (adDGEs) and Kamath-PD (pdDGEs) datasets. Within each dataset, DGEs were computed separately for each cell type (analogous to our main analysis; the “Methods” section). Statistically significant DEGs (FDR-corrected *P*-value < 0.05; the “Methods” section) were subject to further comparison between AD and PD cell type pairs.

**Figure 5:**
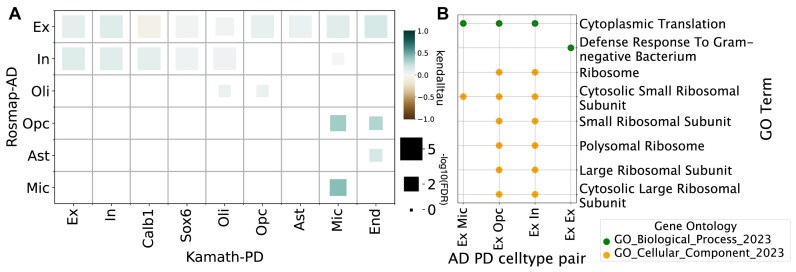
Differential expression analysis revealed modest similarity between AD- and PD-associated transcriptomic signatures. We benchmarked our latent factor model-derived AD-PD overlap with differential gene expression-derived AD-PD overlap. (A) Pairwise associations between AD and PD DGEs are shown (Kendall’s tau-b). For each cell type pair, statistical significance of association was assessed using a permutation test. Colored squares indicate significant associations (FDR < 0.05). Increasing intensity denotes greater similarity (+1) or anti-similarity (-1) between the log-fold changes of significant DEGs from an AD-PD cell type pair. Square size is proportional to −log_10_(FDR). Compared to our PLS gene-module-based overlap analysis (Fig. 2A), significantly smaller correlations emerge from this univariate approach. The maximum correlation observed was 0.4 between AD and PD microglia. (B) Dots represent shared GO terms between AD and PD from gene set enrichment of DGEs. Significant terms in AD or PD from GSEA were assessed (FDR *q* < 0.1). In contrast to 213 shared terms across pairwise AD-PD gene modules (Fig. 4A), only 7 shared terms emerged between AD and PD from our DGE analysis. Ast, astrocyte; Ex, excitatory neuron; In, inhibitory neuron; Mic, microglia; Oli, oligodendrocyte; OPC, oligodendrocyte precursor cell; End, endothelial.

We examined the overlap between the adDEGs and pdDEGs across AD and PD cell types (Kendall’s tau-b associations; Fig. 5A; the “Methods” section). Across 54 pairwise comparisons (6 AD and 9 PD cell types), the highest significant association observed was 0.4, occurring between microglia adDEGs and pdDEGs (FDR *q*-value < 0.05; *P*-values corrected for multiple comparisons). Notably, this highest association among all possible cell type pairings was significantly lower than the maximum similarity observed from our gene module analysis (cf. Fig. 2A).

We then systematically tested the overall difference in mean correlations between AD and PD associations based on pairwise gene module τ_b_ from PLS (12 × 20) versus pairwise cell type τ_b_ from DGE (6 × 9). Using Welch’s *t*-test, which accounts for unequal sample sizes, we observed a significant difference in correlation strengths between the 2 methods. Across pairwise comparisons, PLS_cell_ τ_b_ values were higher than DGE-derived τ_b_ values (Welch’s *t* = 5.51; *P*-value < 0.001). This finding suggested that associations between AD and PD similarity were systematically stronger when using our gene module approach compared to the classical DGE method.

Next, we performed a GSEA of the DGEs using GO databases (GO BP, MF, and CC). Independently for each disease, we created our ranked gene list based on the fold change significance level (conditioned on cell type; the “Methods” section) and used GSEA to identify significantly enriched terms (FDR *q* < 0.1). We observed 7 common terms, in total, between AD and PD across all 3 GO databases (Fig. 2B). Overall, these terms represented only a small subset of the broader set of shared AD-PD terms identified through our gene-module-based analyses (213 GO terms). Notably, the shared terms emerging from DGE analysis (e.g., cytoplasmic translation) appeared among the most frequently recurring terms across gene modules from the PLS analysis (cf. Fig. 4C), suggesting that DGE may primarily capture the strongest disease overlaps from the gene expression matrices.

We reassessed AD-PD overlap with DGEs from Seattle-AD and Smajić-PD dataset pair (Fig. S7). Across all pairwise combinations of relevant AD-PD cell types, the maximum observed correlation was between DEGs and between endothelial cells (−0.5; false discovery rate (FDR) *q*-value < 0.05) and microglia (0.3; FDR *q*-value < 0.05). In addition, a GSEA comparative analysis between AD and PD revealed extremely low number of shared terms (2 terms; Fig. S7B). Taken together, these results suggested that the univariate DEGs identified limited molecular overlap between AD and PD.

In summary, these findings demonstrated that DGE captured only modest overlaps between the molecular signatures of AD and PD brains. In contrast, our supervised latent factor modeling approach not only recapitulated the strongest DGE-derived AD-PD molecular overlaps (cross-disorder microglia), but also revealed a substantially broader set of shared, disease-relevant signatures. This comparative analysis highlighted the added value of identifying cross-disease associations through a multivariate, transcriptome-wide modeling of gene modules, rather than relying solely on individual gene-level differences.

### GWAS-seeded co-expression networks also indicate AD-PD transcriptomic overlap

**Figure 6:**
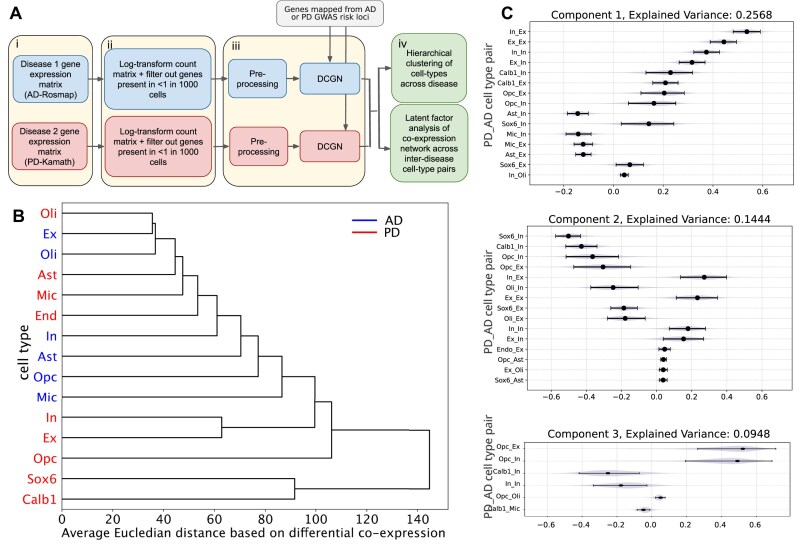
DGCN identifies hints of overlap between AD and PD. To corroborate our findings in a technical replicate, we contrasted DGCNs between AD and PD. We constructed DGCNs based on genes whose expression patterns were co-expressed with seed genes (164 GWAS-nominated genes from AD or PD), but whose synchrony was altered in disease compared with control conditions. (A) Overview of workflow. (i) Single-nucleus RNA seq datasets were downloaded from open-source repositories. (ii) Local processing was performed independently for each cell type, following recommended guidelines. (iii) GCN analysis. For each gene from a set of GWAS-implicated genes from AD and PD, a co-expression network was created for the disease and control groups independently. The results were subtracted to generate the DGCNs. (iv) The disease-specific DGCNs were compared between AD and PD to identify cross-disease cell type associations. (B) Hierarchical clustering of cell types based on the similarity of the co-expression patterns captured by DGCN. The dendrogram captures the average Euclidean distance of DGCNs across all cell type pairs between AD and PD. (C) Kendall’s tau-b was used to calculate the pairwise associations between DGCNs. Cell type pair loadings for the top 3 principal components are shown. The pairs are presented in descending order, based on the loading magnitude. Dots represent the empirical PCA loading. Error bars represent the 20/80% CI from a 1,000-iteration BS analysis, which sampled rows from a DGCN with replacement. Pairs that did not include zero in their BS interval are shown. The first and second principal components are dominated by cell type pairings involving mainly excitatory and inhibitory neurons in AD and PD. The second component emphasizes PD dopaminergic neurons. The third component focuses on glial and vascular cell types from PD across cell types from AD.

In an alternative set of analyses, we pursued the same research question, the extent of AD-PD molecular overlap, using a complementary quantitative workflow. Devising a top-down framework (Fig. 6A) seeded with 164 genes mapped from GWAS risk loci (GWAS in AD or PD), we constructed disease-specific differential gene co-expression networks (DGCNs) using the ROSMAP-AD and Kamath-PD datasets. The notion of gene co-expression networks (GCN) rests on the assumption that genes with co-varying expression profiles across cell transcriptomes often share functional or regulatory relationships [48–50].

We computed DGCNs independently for the ROSMAP-AD and Kamath-PD datasets and separately for each cell type within a dataset (the “Methods” section). By quantifying the alignment between our 2 analysis arms, we measured the correspondence between the relevant genes from DGCN (top-down) and the PLS-derived gene modules (bottom-up) (see the “Methods” section). Specifically, we looked at the number of robust genes that were common in each gene module—DGCN pair (Fig. S8A). We observed strong PLS_cell_ module—DGCN alignment within the same cell type pairs. For example, co-expression signatures from astrocytes shared, on average, 20% of genes (significant ρ at FDR < 0.01) with PLS_cell_ ROSMAP-AD Ast 2. Similarly, excitatory neurons shared, on average, 58.4% of genes (significant ρ at FDR < 0.01) with PLS_cell_ ROSMAP-AD Ex 1 and 42.2% of genes with PLS_cell_ ROSMAP-AD Ex 1.

We quantified the overlap between AD and PD at the DGCN level using a hierarchical clustering analysis (the “Methods” section). This grouped cell types from AD and PD based on their gene co-variation patterns centered on GWAS genes (Fig. 6B). Importantly, as in our primary analysis arm, we did not combine the gene expression matrices; instead, these DGCNs were derived separately for AD and PD. As an overarching observation, this secondary analytical approach recapitulated the main observation from the gene-module-level findings. Specifically, AD-oligodendrocytes and PD-oligodendrocytes were grouped together, suggesting similar co-expression profile alterations in AD and PD.

We then analyzed the extent of AD and PD overlap in yet another way. Using Kendall’s tau-b, we computed the similarity between the same seed-derived DGCNs across AD-PD cell type pairs (see the “Methods” section). Embeddings derived from this similarity matrix (164 GWAS genes × 54 AD-PD cell type pairs; PCA; the “Methods” section) revealed systematic AD-PD cell pairings that shared similar co-expression profiles (Fig. 6C). The first principal component was dominated by robust AD-PD neuronal cell types (25.6% explained variance of co-expression patterns; subjected to BS robustness check; see the “Methods” section). The second component grouped DA neurons in PD (CALB1 and SOX6) with excitatory and inhibitory neurons from AD (14.4% of total variance). The third component was dominated by combinations of microglia in AD and oligodendrocytes and OPCs in PD (9.5% of total variance).

Taken together, these results from our DGCN analysis recapitulated the gene module-based observations, confirming the strongest AD-PD overlap between oligodendrocytes and specific neurons. Thus, this parallel analysis reinforced the robustness of our earlier results through a technical replication.

## Discussion

In this study, we examined the molecular ties between AD and PD using single-cell transcriptomics data. By analyzing the entire protein-coding transcriptome, our multivariate approach uncovered AD- and PD-deviant genes forming co-expressed modules at sub-cell type granularity. Our mappings of these gene modules to disease-relevant biological programs illuminated complex, cell type-specific mechanisms that might lead to shared disease phenotypes in AD and PD. On a broader level, we provide single-cell genomics scientists with a tool to compare any pair of diseases from a global transcriptome perspective.

Our primary analytical protocol was enabled by access to the ROSMAP-AD dataset with 70,634 nuclei from 8 major cell types, and the Kamath-PD dataset with 340,902 recorded nuclei from 11 major cell types. In both datasets, we extracted multiple gene modules within each cell type. These gene modules signified distinct modes of disease-related transcriptional changes at the sub-cell type level. Our grading of the alignment of gene importance between pairwise gene modules from AD and PD demonstrated sizable overlaps. In the spotlight were modules derived from AD and PD oligodendrocytes, astrocytes, and microglia. Significant, albeit more limited, overlap also emerged between AD and PD neuron-derived gene programs.

Similarly high degree of mirrored transcriptional changes between AD and PD were also highlighted in a secondary analysis arm which compared GCNs between the 2 diseases. Additionally, we were able to replicate these findings in an external AD-PD snRNA-seq dataset pair (Seattle-AD, Smajić-PD). Overall, our multivariate transcriptomic analyses offered rigorous and systematic evidence of shared molecular signatures between AD and PD.

Our investigations of GWAS gene hits situated several of these genes within our transcriptome-derived gene modules. For example, APOE, a major AD risk gene, emerged in AD modules and SNCA, a PD risk gene, emerged in PD modules [36, 37, 54].

Central to our investigation, several AD-relevant GWAS genes surfaced in PD-associated gene modules and vice versa, highlighting the interconnectedness between the 2 disease categories. For example, APOE had robust PD associations through several PD gene modules, including those pulled from microglia, astrocytes, oligodendrocytes, and neurons. Indeed, prior clinical studies have highlighted APOE to be predictive of cognitive decline in PD patients [51, 52]. Additionally, the APOE genotype has been shown to exacerbate synuclein pathology in transgenic mouse models [53]. As another example, we considered SNCA. In the PD brain, misfolded SNCA protein is a primary neuropathological marker [55]. Here, along with several PD neuron and glial modules, SNCA emerged as a strong contributor in AD excitatory neuron modules. Previously, APP transgenic mice with SNCA knockout have demonstrated a significant reduction in amyloid burden, hinting at connections between this PD gene and AD pathology [56, 57]. Taken together, this study situated GWAS AD or PD genes within the fuller context of disease-specific gene modules, further expanding the implication of these genes to potential neurodegeneration [12].

Drawing biological insights from pre-curated gene ontologies, we identified overlapping disease mechanisms between AD and PD. Our gene modules were related to shared alterations in cellular energy metabolism and stress response, inflammation, lipid signaling, protein folding, and protein degradation cascades. Some of these overarching disruptions in BPs have been discussed in previous reviews summarizing the collective understanding from decades of neurodegeneration research [57].

For example, a shared feature of neurodegeneration is the presence of abnormal protein aggregates [58, 59]. Consistent with this, our enrichment analysis revealed that protein misfolding (ER stress) and its associated BPs were widespread across all major cell types, diseases, and datasets. We also detected extensive alterations in molecular pathways related to protein degradation (ubiquitin protein ligase binding and clathrin-mediated endocytosis), indicating a potential breakdown in protein disposal systems across multiple diseased cell types. These results align with findings from animal models where malfunctions in ubiquitin-dependent protein clearance are linked to general neurodegeneration [60–62]. Overall, using a clean bottom-up computational workflow, this study confirms several prior findings, while going beyond them to carefully localize disease-relevant molecular processes to cell-type-specific gene modules.

### Impaired molecular mechanisms shared between AD and PD in neurons

Neurons are particularly sensitive to proteasomal turnover due to their longevity and delicate synaptic regulatory requirements [62]. Zooming in on neuronal gene modules, we identified neuron exclusive mechanisms that were shared between AD and PD. Microtubule-associated processes localized almost uniquely to neuronal modules in both AD and PD (genes included APP, NEFL, TUBA1B, GAPDH, TUBB2A, CALM3, and MAPT). Several lines of prior evidence, including microscopy and genetic studies, have cited defects in cytoskeleton dynamics as major contributors to neuronal death [63]. Further, alterations in post-translational modifications of microtubule acetylation levels have been reported in in vitro and in vivo studies of both AD and PD [64, 65].

In addition, our neuronal modules were also enriched for terms related to alterations in mitochondrial functions, including axonal transport of mitochondria, and protein localization to mitochondria (all 4 datasets). This alludes to a vicious cycle between dysregulated microtubules and impaired mitochondrial transport [66]. Previous research has shown that alterations to these dynamics lead to the overproduction of mitochondrial reactive oxidative species (ROS) [67, 68]. ROS, in turn, exacerbates the levels of free tubulin [69], which in turn has been shown to interact with proteins like α-synuclein, promoting the formation of oligomeric aggregates in the form of Lewy bodies [70], or tau tangles [68].

The involvement of the MAPT gene in disease-relevant neuron modules from both AD and PD was also noteworthy. This gene encodes the protein tau and is responsible for stabilizing axon microtubules. Disruptions and alterations in this gene have been previously associated with multiple tauopathies and PD. Tau, in its aggregated form, demonstrates prion-like behavior, passing from neuron to neuron across synapses, a mechanism increasingly recognized in both AD and PD [71–75]. In our analysis, the emergence of MAPT across both disorders highlights a shared therapeutic target, motivating cross-disease strategies aimed at limiting pathological protein spread and neuronal death.

### Apoptosis and cytochrome c regulation pathways

The emergence of terms related to the regulation of cytochrome c release and the apoptotic signaling pathway in several AD and PD neuronal gene modules was notable. In an intact human cell, cytochrome c is present in the mitochondrial intramembranous space. However, oligomeric forms of amyloid-β, α-synuclein, and tau have been shown to increase mitochondrial membrane permeability, causing leakage of cytochrome c into the cell cytosol [76]. This loss disrupts cytochrome c oxidase function and compromises ATP production. Once in the cytosol, cytochrome c initiates mitochondria-mediated apoptosis [77], a form of neuronal death long suspected as an early event in the pathophysiological cascade leading up to AD [78] and PD [79, 80]. Specific to PD, in a self-reinforcing manner, impaired cytochrome c release from mitochondria is thought to escalate α-synuclein oligomerization via radical formation [81]. Whether similar mechanisms exacerbate plaque and tangle aggregation in AD remains an important avenue for further investigation.

### Oligodendrocyte and oligodendrocyte precursor cell modules in AD and PD

In oligodendrocytes and OPCs, we located gene modules with high similarity between AD-PD. Three key observations stand out related to these modules.

First, we observed that a sizeable portion of GWAS genes localized to cross-disease oligodendrocyte modules. Notably, PD oligodendrocyte modules contained more GWAS genes than modules from other cell types. Previous transcriptomic studies have pointed out that several disease risk loci are associated with oligodendrocytes in both AD [82] and PD [83]. Our study confirms and expands on this observation.

Second, in our enrichment analysis, several BPs revolving around myelination and regulation of axonogenesis were specific to these cell type modules and were shared between AD and PD. Prior correlative macroscopic brain-imaging studies have linked deteriorating myelin health to AD progression [84]. Further, a recent invasive experiment posited a causal link between aging myelin and AD [85]. In a few AD mouse models and a human PD model, transcriptomic analysis identified changes in oligodendrocyte transcription specifically related to impaired myelination [35, 86, 87].

Finally, in addition to strong intra-cell-type cross-disorder module associations, the AD oligodendrocyte modules also exhibited a high degree of association with AD and PD excitatory neuron gene modules. Such similarity in transcriptional modifications between oligodendrocyte and excitatory neurons was previously reported in the ROSMAP-AD transcriptomic study, in terms of shared DEGs [36]. The extension of this inter-cell-type overlap to cross-disorder settings might suggest pervasive crosstalk between excitatory neurons and oligodendrocytes in neurodegeneration.

### Shared role of heavy metals is highlighted between AD and PD

In our study, gene modules from astrocytes showed exclusive enrichment for response to zinc ions in both AD and PD. Several metallothionein (MT) genes were common between these AD and PD modules (MT1E, MT1G, MT2A). Astrocytes, which remove excess heavy metals from the brain parenchyma [90], are susceptible to A1-type reactive astrogliosis in the presence of excess zinc and promote synaptic degeneration in neurons [91]. In general, human and animal research has shown that dysregulated homeostasis of multiple heavy metal ions can lead to an increased risk of onset and progression of neurodegenerative diseases [88, 89].

Another metal ion, copper (Cu), was notably enriched across our AD and PD neuronal and glial modules. This is consistent with a previously implicated metal dyshomeostasis axis linked to ROS and protein misfolding [92–94]. Biophysical and biochemical experiments have underscored alterations in Cu ion levels that trigger misfolding of α-synuclein [95] and amyloid-β [96].

However, here, a key distinction emerged between the neuronal and glial copper-handling gene modules—glial modules included genes related to buffering/detoxification responses (via MT1E, MT2A, MT3, and APP), whereas excitatory neuron modules uniquely enriched copper-binding genes tied to oxidative stress and proteostasis (via critical antioxidative enzymes [SOD1, PARK], SNCA, and copper chaperone protein [ATOX1]). Prior *in silico* analyses of microarray data on brain tissue had reported a similar grouping of copper-handling genes (metallothionein group and the enzyme binding group) [97]. Therapeutically, these findings support cell type-targeted interventions; for example, enhancing glial copper-buffering capacity (e.g., boosting metallothionein pathways) to stabilize extracellular redox balance, while simultaneously protecting neurons with copper-modulating and antioxidant strategies (e.g., targeting SOD1- or ATOX1-lined pathways) to reduce ROS-driven proteotoxicity. Thus, our cell-type-specific module-based approach provides important clues for precise drug treatment design.

In parallel, a third metal ion, iron ion, homeostasis terms were found to be enriched in neuron gene modules from both AD and PD. Across all datasets, excitatory and inhibitory neuron modules were involved. In the brain, iron plays a key role in myelin synthesis, neurotransmitter production, and overall metabolism [98]. However, elevated levels of redox-active iron, often originating from degenerating mitochondria, accumulate in several neurodegenerative diseases as evidenced by biochemical, and transgenic animal studies [99–102].

Together, these findings position disruptions of heavy metal pathways as shared therapeutic targets in AD and PD.

### Shared microglia-specific molecular changes in AD and PD

Functionally, microglia are the primary immune cells of the CNS [103], and neuroinflammation and immune system dysfunction are believed to be key components of neurodegeneration [104]. Consistently, microglial gene modules from our study showed enrichment for several immune-related processes. For example, T cell activation terms were associated with microglial modules from both AD and PD (all 4 datasets). Microglia, upon activation by neuronal stress, are thought to release pro-inflammatory cytokines and upregulate MHC class I and II molecules [105]. Further, the inflammatory cytokines can induce the expression of adhesion molecules on brain endothelial cells, compromising the integrity of the blood–brain barrier (BBB). This BBB breakdown accelerates peripheral immune cell entry. Thus, in a chicken-egg scenario, microglia and endothelial cells drive a chain reaction of T cell activation, oxidative stress, and neuroinflammation [106–108]. This domino effect may have been captured in one of our PD endothelial modules whose pathways were associated with T cell activation terms as well as association with BPs like leukocyte adhesion to vascular cells, blood vessel morphogenesis, and diameter maintenance, pointing to the dysregulation of the BBB. This cascade of immune response events might exacerbate ROS production and neuronal damage [106, 107].

Further, we found a robust response to TH-related terms in microglial gene modules from both AD and PD. Within the brain, TH imbalances are an important contributing factor to increased ROS [109]. Epidemiological evidence has linked multiple thyroid-related autoimmune diseases to increased prevalence of both AD [110, 111] and PD [112]. However, the exact contribution of TH to either AD or PD pathophysiology has not been fully established. Recently, an AD mouse model has linked brain hypothyroidism with reduced microglial reactions to inflammatory stimuli and aberrant amyloid-β [113]. In PD, an α-synuclein PD mouse model found connections between TH and glucocerebrosidase activity in microglia [114]. Our findings, which highlight TH involvement in both AD and PD microglial gene modules, warrant further investigation of the thyroid axis.

We also identified microglial gene modules linked to synapse pruning in AD and PD. These modules implicated key genes, including TREM2 and several C1q genes from the complement system, an integral component for synaptic refinement. Synapse loss is frequently observed as an early event of neurodegeneration in animal models [115–117]. Variants of the TREM2 protein and aberrant activation of C1q genes have been shown to cause irregular synapse pruning in studies on AD mouse models [117–119]. In a PD mouse model, suppressing TREM2 gene products in microglial cells was shown to accelerate the loss of DA neurons [120, 121]. Yet, the totality of glia-synapse interactions, especially the role of the complement system in PD, is under-investigated [122, 123]. Once again, our bottom-up approach identified a common transcriptomic signature in AD and PD in the form of genes related to synapse loss—a potential area for further investigation.

In addition, lipid transport regulation terms were enriched in both AD and PD microglial gene modules (lipid, phospholipid, cholesterol, and sterol transfer terms) and involved key genes including APOE, TSPO, TMEM30A, several ATP-binding cassette subfamilies (ABC) genes (ABCG1, ABCA5), and NPC2. Excess lipid has been reported to accumulate as droplets in human iPSC-derived microglia, reducing their phagocytic capabilities and increasing the secretion of pro-inflammatory cytokines [124, 125]. These microglia mediated impairments ultimately exacerbate ROS burden and neurotoxic buildups leading to neurodegeneration.

In summary, our proof-of-principle investigation of the transcriptomic terrain intersecting AD and PD identified and characterized several shared molecular basis of neurodegeneration. Specifically, we were able to (i) quantify the degree of transcriptomic overlap between AD- and PD-related changes and (ii) localized these overlapping molecular changes to distinct cell types. In addition, the cell type-specific genes identified within convergent AD-PD–associated modules may represent potential therapeutic targets warranting further investigation.

Several limitations should be considered when interpreting our findings. While the present study focuses on the computational identification of disease-relevant gene programs and a subsequent comparison between AD and PD, future experimental work using cellular systems or animal models targeting the highlighted modules and shared pathways will be necessary to further investigate AD-PD convergence *in vivo*. For example, recent animal research on gut–brain axis disruptions in AD identified common therapeutic intervention strategies that might be applicable across neurodegenerative disorders [32, 126]. In addition, although the datasets analyzed in this study are among the largest currently available, larger and balanced cohorts will likely enable more robust estimation of gene modules, providing more granular insights into sex- and cell type-specific transcriptional basis of AD-PD overlap.

Future work can expand our study to include a greater diversity of neurodegenerative, neurodevelopmental, and psychiatric diseases. Moreover, applying a similar computational framework to different, readily accessible source transcriptomes, like blood or cerebrospinal fluid, can identify critical biomarkers for neurodegeneration.

## Materials and methods

### Single genomics data resources

#### Primary datasets

ROSMAP Alzheimer’s dataset [36]: The snRNA-seq dataset was derived from postmortem brain tissue from the prefrontal cortex (BA10) of individuals participating in the ROSMAP [127]. The dataset was collected from 48 subjects who were carefully matched in terms of age and sex (24 males and 24 females). Of these, 24 individuals were diagnosed with AD, and 24 were control subjects. The mean age was 85 years. The recorded cell types included excitatory neurons (*n* = 34,976), oligodendrocytes (*n* = 18,235), inhibitory neurons (*n* = 9,196), astrocytes (*n* = 3392), OPC (*n* = 2,627), microglia (*n* = 1,920), pericytes (*n* = 167), and endothelial cells (*n* = 121). The dataset encompassed transcript counts for 17,926 protein-coding genes, aligned with the human reference transcriptome hg38 (GRCh38.p5).

PD, Kamath dataset [40], GEO accession number GSE178265: This dataset included snRNA transcriptomes from postmortem human midbrain and dorsal striatum (caudate nucleus and substantia nigra) tissue. Tissue samples were derived from an age- and sex-matched cohort of 21 subjects in total (control, PD, and Lewy body disease). Samples derived from patients with Lewy body disease (4) were excluded from our analysis. To match the mean age of the ROSMAP-AD cohort, 3 patients <50 years of age were excluded. The final dataset consisted of 6 PD patients, while the remaining 7 were controls. Among this group, 6 were males, and 7 were females. The mean age of included individuals was 83 years. Major cell types in this dataset included oligodendrocyte (*n* = 134,940), excitatory neuron (*n* = 40,956), inhibitory neuron (*n* = 31,545), SOX6 (*n* = 25,482), astrocytes (*n* = 24,475), microglia (*n* = 24,038), CALB1 (*n* = 15,871), endothelial (*n* = 12,609), OPC (*n* = 9,603), macrophage (*n* = 955), and ependyma (*n* = 167). The dataset provided transcript counts for 33,692 genes, which were aligned to the hg19 genome.

#### External validation datasets

Seattle Alzheimer’s dataset [37]: The authors of this resource sourced brain specimens from the Adult Changes in Thought Study and the University of Washington’s Alzheimer’s Disease Research Center. Brain tissue samples were drawn from the middle temporal gyrus. The study included participants from age groups ranging from <65 years to more than 90 years. To maintain consistency with our other datasets, we considered only participants over the 65–77-year age bracket. This gave us 76 individuals, with 47 females and 29 males. Out of these, 36 subjects had recorded dementia, and 40 were controls. The mean age of the individuals was 88 years. The preprocessed datasets for the cell types labeled “L4 IT,” “L5 IT,” “Vip,” “Pvalb,” “Sst,” “Sncg,” “Oligodendrocyte,” “Microglia,” “Astrocyte,” and “OPC” were used. This resulted in the following nuclei being available for our analysis: sncg (*n* = 22,168), sst (*n* = 58,265), pvalb (*n* = 90,804), astro (*n* = 70,009), endo (*n* = 2,069), opc (*n* = 32,493), micro (*n* = 40,000), oligo (*n* = 111,194), l4_it (*n* = 168,860), l5_it (*n* = 128,090), and vip (*n* = 104,514). In total 36,517 genes were recorded and mapped to hg38 (GRCh38-2020-A) human reference genome.

PD, Smajić dataset [35], GEO accession number GSE157783: The creators of this resource worked with postmortem midbrain tissue sections that were linked with clinical and neuropathological data from the Parkinson’s UK Brain Bank and the Newcastle Brain Tissue Resource. The dataset included age and sex-matched nuclei samples from 6 controls and 5 idiopathic PD patients, all of whom exhibited severe neuronal loss in the substantia nigra and had no family history of the disease. The mean age of the subjects was 80 years. The major cell types were oligodendrocytes (*n* = 21,268), astrocytes (*n* = 4,708), microglia (*n* = 3,903), excitatory neurons (*n* = 3,037), OPC (*n* = 2,754), endothelial cells (*n* = 1,723), inhibitory neurons (*n* = 1,548), and pericytes (*n* = 1,229). Four cell types had fewer than 500 nuclei and were excluded from our analysis—ependymal cells (*n* = 536), GABA (*n* = 535), CADPS2+ neurons (*n* = 120), and DaNs (*n* = 74). The total number of genes was 24,005, and these were mapped to hg38 reference genome.

#### Control dataset

Lung disease, Kaminski dataset [128], GEO accession number GSE136831: The authors of this COPD dataset sourced nuclei from human distal lung parenchyma specimens. In total, 18 COPD patients and 28 control donor lungs were sampled. This resulted in the following nuclei being available for our analysis: myeloid (*n* = 174,146), multiplet (*n* = 3,765), lymphoid (*n* = 34,626), stromal (*n* = 6,607), endothelial (*n* = 2,069), epithelial (*n* =18,030). In total, 32,922 genes were recorded and mapped to hg38 (GRCh38) human reference.

#### Preprocessing pipeline at source

We relied on the preprocessed datasets from the authors responsible for the data collection (cf. above). This maximized reproducibility and compatibility with other studies working with these resources. The transcriptomic datasets were processed in the source studies using standardized snRNA-seq processing pipelines. These included quality control for cell inclusion, including doublet detection, the removal of low-quality and outlier cells, the removal of lowly expressed genes, and sample-level batch correction procedures.

Cell type classification—which cell belongs to which cell population—was taken from the original studies as a basis for our investigations. Exact details can be found in the Methods section of the independent research.

#### Primary local preprocessing

Different cell types perform widely diverse functions, each arising from the functional recruitment of distinct gene groups. In our pursuit to discover biologically meaningful gene groups, we implemented our quantitative analysis pipeline for a given disease on a cell type-by-cell type basis. This agenda enabled us to extract coherent latent components (latent factor/loading vector, hereby referred to as gene module) specific to each cell type.

To ensure an even sample size in the disease and control group, we randomly sub-sampled transcriptomes of a given cell type to have comparable counts of nuclei from both disease and control instances. On this sub-sampled data, we removed genes that were captured in fewer than 1 out of 1,000 cells to reduce our model’s degrees of freedom (*scanpy.pp.filter_genes*), which is in line with previous research [129, 130]. To reduce technical variation from sequencing depth, we normalized the data by dividing the raw UMI count by the total number of detected UMIs in each cell (*scanpy.pp.normalize_total(*data, target_sum=1e4)). To account for the heteroskedasticity originating from differences in highly expressed versus lowly expressed genes, we log-transformed the normalized gene expression data (*scanpy.pp.log1p*(data)). Taken together, these dataset transformations have been shown to work well as a preparatory step for downstream dimensionality reduction [131].

In an additional data cleaning step, postmortem interval (PMI) was included as a covariate to account for potential confounding effects on gene expression. PMI has been shown to be a potential source of confounding elsewhere [132, 133]. Specifically, here we regressed out the variation in gene expression attributable to differences in PMI. The resulting adjusted, cleaned, and standardized transcriptomic profiles for each examined cell were used for subsequent steps in our modeling pipeline.

We restricted the feature space to protein-coding genes in each dataset. This step reduced the ambient gene space dimensionality, which is beneficial for any high-dimensional analysis [134, 135]. Specifically, for each dataset, we considered the genes that overlapped with the set of 17,019 protein-coding genes in ROSMAP-AD. This filtering resulted in 16,936 genes in the Kamath-PD dataset, 12,681 genes in Seattle-AD, and 15,137 genes in Smajić-PD. As a result, the analyzed datasets were embedded in ambient feature space of comparable dimensionality, with a similar set and order of genes, with broadly consistent biological properties being evaluated across analysis.

Crucially, the transcriptomic datasets from different studies were at no point merged or jointly integrated for our analyses. Instead, each dataset was analyzed independently to derive disease-associated gene modules. Within each dataset, model fitting and hyperparameter optimization were performed separately. Thus, by avoiding cross-dataset integration, our analysis circumvented the need for cross-dataset batch-effect corrections. In summary, comparisons between AD and PD were performed at the level of model-derived gene loadings, and not experimentally recorded gene expression.

#### Identifying gene modules: supervised latent factor modeling

At the heart of this study, we sought to identify synchronous gene expression changes that occur in the brain in association with disease state, and compared them between AD and PD. To achieve this, we employed a latent factor approach to identify gene programs in a multivariate framework, rather than examining individual genes independently. Unsupervised latent factor models have bee previously used in snRNA-seq analyses to identify hidden gene programs [136–138]. In contrast, our implementation uses a supervised approach that identifies structured patterns in the high-dimensional gene space while simultaneously modeling the relationship between gene expression and the associated disease status.

This work builds on a prior study in which the multivariate method, PLS-DA [139], was employed to derive AD-predictive gene modules across major brain cell types [42]. In contrast to the original study, where the analyzed dataset had favorable observations-to-features ratio, several datasets analyzed here comprised substantially fewer samples within individual cell types. In these cases, PLS-DA is susceptible to overfitting, particularly given the inherent noise and sparsity of snRNA-seq data [140]. To address this limitation, we applied principal component analysis (PCA) denoising on the input gene expression matrix, prior to model fitting. Under a low-rank assumption on the input feature space (gene expression), dimensionality reduction with PCA yields an optimal low-rank approximation that is robust to noise and sparsity [27, 141]. In practice, such transformations are routinely applied in single-cell and single-nucleus analysis, where the ambient gene space is well approximated by a low-rank structure [142].

Specifically, for each cell type within individual datasets, we applied PCA to the input gene expression matrix. These components were then used as input variables for PLS-DA to obtain a projection maximizing disease versus control class separation. To guard against spurious derived structures [143], we performed strict model validations (see below), including label-shuffled permutation tests to assess the statistical significance of derived components (Figs S1B and S3B).

Formally, let ${X}_{{\mathrm{ ambient}}}\in\ {R}^{{N}_c \times {\mathrm{M}}}$ be the input gene expression matrix, and $y\in\ {R}^{{N}_c \times 1}$, where *M* is the number of genes, and ${N}_c$ the number of observations (nuclei) for a cell type *c*. *Y* represents the disease label (+1 for disease and −1 for control) for each nucleus. Let ${M}_c$ be the number of chosen PCA components. Then, the input datasets to the PLS-DA models can be denoted as $X\in\ {R}^{{N}_c \times {{\mathrm{M}}}_c}$.

Concretely, PLS-DA can be viewed to consist of 2 key equations:


\begin{eqnarray*}
X = T{P}^T + E,
\end{eqnarray*}

\begin{eqnarray*}
Y = U{Q}^T + F,
\end{eqnarray*}

where *T* and *U* are ${N}_c \times {k}_c$ score matrices of *k_c_* extracted components, *P* is a ${M}_c \times {k}_c$ loading matrix (effect size) of *X*, and *Q* is a $1 \times {k}_c$ loading vector of *Y*, respectively. *E* and *F* are the residual matrices of *X* and *Y*, respectively. The decomposition of *X* and *Y* is set to the solution of the optimization objective:


\begin{eqnarray*}
\mathrm{ cov}{\left( {t,{\mathrm{\ }}u} \right)}^2 = {\mathrm{\ }}\mathrm{ cov}{\left( {Xw,{\mathrm{\ }}Yh} \right)}^2,
\end{eqnarray*}

where $\mathrm{ cov}( {t,\ u} )$ is the captured covariance $\frac{{{t}^Tu}}{{{N}_c}}$, *w* and *h* are weight vectors that are extracted using the NIPALS algorithm [144].

To transition from the PCA embedding space (${M}_c$) back to the gene space (*M*), we projected the PLS loadings back into the original gene expression space (approximated using sklearn *PCA.inverse_transform)*. This ensured that the domain interpretability of the PLS estimates was preserved in the biological ambient space—high absolute gene loadings signaled strong contributions (positive to the target disease, negative to the control group), while near-zero loadings indicated minimal impact. To assess the statistical robustness of derived gene-level loadings, we focused on genes that were consistently selected across model refits based on BS resamples of the dataset (see below). This procedure allowed us to identify genes that were stable across different realizations of the data, further reducing the likelihood that the reported results were driven by sampling noise or unstable features.

#### Model selection, training, and performance assessment

After preprocessing and cleaning the transcriptomic data resources, we carried out the training of our supervised learning models. First, the number of PCA components was chosen using $\mathrm{ min}( {500,\ {N}_c} )$, where ${N}_c$ was the number of recorded nuclei for the given cell type *c* in the dataset. Next, the optimal number of latent components for each cell type-specific PLS model was determined using a rigorous 10-fold CV scheme. Selecting this optimal number of latent components was crucial—choosing too few components implied losing out on important information and too many could lead to overfitting. To do this hyperparameter selection, the set of cells was randomly split into 10 equal-sized data point subsets. We ensured that the disease-to-control ratio of cells for each subset reflected that of the full dataset. Screening a range of component choices (1–8) in each iteration, 9 out of these 10 data subsets were combined and used for training a PLS model, while the out-of-bag subset was used to assess the component number choice. The model’s performance was evaluated based on the area under the receiver operating characteristic curve (AUROC) in disease discrimination. This was performed for all combinations of training and validation subsets (*scikit-learn model_selection.GridSearchCV* function with *PLSRegression* as the “estimator,” “scoring” set to “roc_auc”, and “n_components” parameter set to 1–8). The number of components yielding the maximum mean AUROC over the CV subsets was noted as optimal for the given cell type.

For each cell type, we then fitted optimal PLS models on the full set of transcriptome observations (PLS model specified using *sklearn.cross_decomposition* module *PLSRegression*). To audit the performance of the individual PLS models, we employed AUROC of disease classification as our evaluation metric. Given that cell samples from a patient can exhibit significant autocorrelation, it was crucial to account for this when evaluating the model. Traditional test-train splitting methods often involve blindly partitioning the dataset. This can result in overly optimistic test performance and makes it challenging to detect overfitting during the testing phase [145]. In light of this, we employed a variation of CV combined with bootstrapped Latin partitions [146] that ensured patient-level stratification.

Concretely, in each iteration, a random sample of subjects (not cells) was drawn with replacement. This formed the basis of the training dataset’s nuclei source. Based on the subset of patients, a random sample of cells was drawn, with replacement, while ensuring that the disease-to-control ratio of nuclei was reflective of the empirical dataset. The percentage of cell samples from any given subject was also preserved in each iteration. This analytical protocol ensured that transcription signatures from the same patient were not present in the train and test sets at the same time. Individual PLS models were fitted on the re-sampled train dataset. This fitted model was then evaluated based on AUROC scores on the test set nuclei, i.e., the transcripts from subjects that were not included in the training step. We performed 1,000 iterations of this BS-based model disease classification performance evaluation. This allowed for a principled assessment of the disease discrimination strength of the PLS solutions based on the cell type-specific gene transcription signatures.

#### Statistical tests of PLS-derived gene modules and gene loadings

Our cell type-specific hyperparameter selection allowed us to independently derive the number of PLS-DA components (gene modules) that maximized disease-versus-control identification within each cell type. The statistical significance of an overall gene module was assessed in a principled, non-parametric permutation procedure. In 1,000 permutation iterations, the transcriptome signatures were held constant, while the disease labels (outcome of model) were shuffled randomly. The resulting surrogate datasets preserved the statistical structure of gene expression profiles while selectively destroying the association of the transcription profiles (model input) with diagnosis (model output). This approach generated a null distribution with minimal modeling assumptions [147, 148].

The empirical covariance (test statistic) between the gene expression and disease signature captured by each module ($\mathrm{ cov}( {t,\ u} )$ defined above; *t*=*model*.x_scores_; *u*=*model*.y_scores_) was compared with the resulting permutation distribution. This distribution reflected the null hypothesis of random association between gene transcription and the disease designation, against which the actual model instance was tested. We deemed significant a module’s input-output covariance in the latent space if fewer than 5% of the null models yielded a better covariance strength than the original covariance from the actual model instance (Fig. S1B). In case a module failed to pass this label-shuffling permutation test, it was dropped from further analysis, along with all underlying gene modules for that cell type. Thus, in a data-driven approach, we were able to determine which gene modules in a cell type at hand carried enough information that allowed us to discern a biological signal from noise.

To identify the subset of the examined genes that robustly contributed to disease detection in each gene module from a cell type, we implemented a 500-iteration BS scheme. The BS resampling was done by selecting nuclei, with replacement, from the cell type observations before applying dimensionality reduction. This approach simulated random nuclei sample draws that could have been derived from the broader cell population. Dimensionality reduction (PCA) and PLS model estimation were performed on the resampled BS dataset in an identical fashion (cf. above).

An inherent ambiguity of the class of latent factor models (i.e., aspects of model non-identifiability), including PCA and PLS, is the reflection invariance of derived latent vectors. To remedy this source of indeterminacy, we computed the cosine similarity (γ; range −1 to +1), between a BS loading vector and its corresponding empirical loading vector. In the scenario where γ was <0, indicating a flipped (“mirrored”) loading vector, we multiplied the loading vector of the BS model elementwise by −1 to align it with the original loading vector. This method has been employed by previous authors to address the issue of reflection [42].

The resulting distribution of loadings for a gene in a module was compared to its counterpart in the original model estimate. We disregarded any genes whose model coefficient (PLS loading corresponding to this gene) included zero in its 5/95% BS-CI for that module. That is, the gene effect was removed by setting the loading value to zero. The resulting “robust” gene modules are denoted as ${P}^*$ (dimension $p \times {k}_c$; cf. PLS definition section above) in future references.

To evaluate the potential influence of demographic confounding factors, specifically age and sex, we modeled the component scores for each latent gene module as a function of diagnosis, age, and sex. For each gene module, component scores were regressed on diagnosis (primary variable of interest) together with age and sex (potential confounders) using ordinary least squares regression from the Python *statsmodel* package (v0.14.4). Following model fitting, an analysis of variance was performed to quantify the proportion of variance in component scores (*R*^2^) that was explained by each predictor (anova_lm, typ=2; *statsmodel* v0.14.4). In total, 72 gene modules were analyzed (12 ROSMAP-AD, 20 Kamath-PD, 25 Seattle-AD, and 15 Smajić-PD). This step provided a unique estimate of the contribution by each predictor while accounting for other variables in the models.

#### PHATE visualization

To gain a synopsis of the uniqueness of different gene modules from a single cell type, we created a concise, low-dimensional representation of high-dimensional cellular transcriptomes. For this purpose, we applied dimensionality reduction using PHATE [149]. Compared to prevalent visualization techniques like tSNE or UMAP, PHATE is well suited to preserve both local and global structures in a dataset and can capture non-linear relationships in the transcriptomic information. We estimated a separate PHATE model for each cell type and projected their transcriptomes to independent low-rank spaces (using the *scanpy external.tl.phate* function with default parameters except n_pca = 500). We colored the cells in the PHATE embedding space based on their PLS score from each gene module (*PLSRegression x_scores_*).

#### Quantifying degree of associations between cross-disease latent gene modules

To quantify the similarity between AD and PD at the level of gene expression patterns, we computed the association between the AD and PD gene modules. Importantly, a similarity metric (correlation) was computed across the derived model’s predictive rules for a disease. That is, we did not pit the raw gene expression measurements against each other.

Formally, for a disease *d* and cell type *c*, the *i*th robust gene module (cf. above) was denoted as $p^{*d}_{c,i}$, where p* is a vector of dimension $1 \times M$ (number of genes in the input feature space). A robust gene module could contain tens to thousands of genes with non-zero loadings (out of ∼17,000 genes), while the remaining loadings were zero (cf. above). To minimize the impact of tied zero loadings in the correlation metric calculation between 2 modules, we considered only those genes that had non-zero weights in both modules (AND conjunction). This approach worked well to identify groups of genes with similar disease contributions between 2 modules while ignoring genes that had robust effect sizes in one disease but not the other.

Concretely, we employed Kendall’s tau-b ranked correlation metric (${\tau }_b$) to evaluate pairwise correlations between AD and PD gene modules. Kendall’s tau-b correlation effectively handled tied ranks and provided a more accurate measure of ordinal association between gene modules. This was unlike Pearson’s *r*, which assumes monotonicity and is unstable, or Spearman’s *r*, which is biased and difficult to interpret [150]. For all pairwise gene modules from AD (12 modules across all cell types) and PD (20 modules across all cell types), Kendall’s tau-b rank correlation coefficient, ${\tau }_b( {( {c1,i} ),( {c2,j} )} )$, was calculated using the scipy function *stats.kendalltau*(${p}_{( {c1,i} )}^{*\mathrm{ AD}},{\mathrm{\ }}{p}_{( {c2,j} )}^{*\mathrm{ PD}}$), where $( {c1,i} )$ was the *i*th gene module for the cell type $c1$ present in the AD dataset, $( {c2,j} )$ was the *j*th gene module from the cell type $c2$ present in the PD dataset.

To independently assess the statistical significance of each coupled module association, we employed a non-parametric permutation procedure with the null hypothesis of random association between gene modules from different diseases. For each gene module pair, we reutilized the label shuffling derived module weights (cf. above) to calculate a null distribution from 1,000 permutation iterations. We only interpreted a module-pair’s correlation coefficient that emerged as statistically significant against a 5/95% CI threshold.

In parallel, we reported the FDR corrected *q*-values for the computed pairwise ${\tau }_b$, with multiple testing controlled using the Benjamini–Hochberg procedure [151] across all pairwise comparisons.

A split-half test was also conducted to estimate the statistical sensitivity of sample size to the coupled associations of cross-disease gene modules. Across 500 iterations, we randomly bisected the empirical AD and PD datasets (before cleaning and standardizing) into 2 pairs of AD-PD subsets. We ensured to preserve the original proportion of cell type nuclei and the disease-control ratio for each cell type. Since the bisection reduced the effective observation size of each dataset to half, the downstream PLS fit enabled a robustness check of sample size for derived gene modules. For each AD-PD subset pair, we ran our workflow steps A-D in parallel (Fig. 1), resulting in 2 analogous sets of gene modules (a couple of 12 AD and 20 PD modules). We then performed Kendall’s tau-b correlation across these modules, giving us 2 correlation matrices of dimension 12 × 20. We unraveled these matrices and calculated Pearson correlated ($\rho $) of the absolute ${\tau }_b$ values. We used absolute values since we were interested only in association strength, not direction. This analysis allowed us to compare the ${\tau }_b$ correlation levels between different gene module pairs derived from smaller subsets of the AD and PD datasets.

### Differential gene expression

Differential gene expression is ubiquitously used in snRNA-seq analysis to identify genes that show statistically significant differences in expression levels between 2 conditions or groups [152]. It is a univariate method, meaning it examines each gene individually, thus losing key information hidden in gene co-expression patterns. We employed this traditional method to serve as an acid test for the proposed approach in this study.

Differential gene expression was performed using MAST (v1.36.0), implemented in R and accessed from Python using rpy2 [153]. Within each dataset, log-normalized single-nucleus expression data were analyzed separately for each cell type. For each gene, a generalized linear hurdle model was fitted with diagnosis (disease versus control) as the primary variable of interest. Cellular detection rates were included to account for differences in gene detection across cells. Age, PMI, and sex were included as covariates to account for potential confounding effects.

Significance was assessed using likelihood ratio tests as implemented in MAST. Multiple testing correction was performed across all assessed genes using the Benjamini–Hochberg method [151]. Effect sizes were quantified using the estimated log_2_ fold change between disease and control groups. Genes meeting the statistical significance threshold after correction were designated as DGEs. The final DEGs were referred to as adDEGs for AD and pdDEGs for PD.

To estimate the pairwise association between cell-type-specific adDEGs and pdDEGs, we computed Kendall’s tau-b using the log-fold change values of genes in the AD-PD AND conjunction set (see above). This resulted in a similarity matrix of dimension 6 × 9, corresponding to the number of AD (6) and PD (9) cell types. Statistical significance for each association was evaluated using a permutation test with 1,000 iterations, in which the fold change values of overlapping genes were randomly shuffled. FDR correction was performed across all comparisons (54) using the Benjamini–Hochberg procedure.

### Quantifying difference in association strengths between PLS- and DGE-derived conclusions: Welch’s *t*-test

To formally quantify the cross-disease association information extracted by our latent factor approach versus DGE, we used a statistical test to compare the central tendencies of the respective correlation measures. Welch’s *t*-test was a natural choice of method here as the number of correlated combinations being compared were different (240 ${\tau }_b$ gene module combinations from PLS and 54 ${\tau }_b$ cell type combinations from DGE), and the variances were not assumed to be equal [154, 155]. Welch’s *t*-test can be formally computed as


\begin{eqnarray*}
t = \frac{{\overrightarrow{X1}}-{\overrightarrow{X2}}}{{\sqrt{\left(s1^{2}/N1 \right)+\left(s2^{2}/N2\right)}}}.
\end{eqnarray*}

In our case, ${\overrightarrow{X1}}$ was the unraveled PLS correlation vector (1 × 240; cf. Fig. 2A), and ${\overrightarrow{X2}}$ was the unraveled DGE correlation vector (1 × 54; cf. Fig. 5). $s{1}^2$ and $s{2}^2$ were the variances of these vectors, $N1$ was the total number of cross-disease gene module pairs from the latent factor analysis, and $N2$ was the number of cross-disease cell type pairs considered in the DGE analysis.

### Identifying biological signaling pathways from gene modules: GO enrichment analysis

To query the biological meaning of our gene modules, we performed a GSEA [28]. Here, we used the GSEApy Python package [156], which itself uses Enrichr [157]. GSEApy is designed to extract statistically over-represented gene sets (example pathways) from a ranked gene list encompassing the whole genome. We used the GO BP, MF, and CC [158, 159] databases as gene sets of interest. We focused on GO, as collectively, they cover the largest fraction of the genome. Concretely, for each gene module, we fed the PLS gene loadings for the entire transcriptome recorded in our datasets to the enrichment tool (*gseapy.Prerank* tool with parameters rnk = gene loadings, min_size = 15, max_size = 1,500, and permutation = 1,000 for significance testing). We reported the pathways that had an FDR threshold of at most 0.05. This step was repeated identically and independently for all gene modules from each cell type, across all datasets.

To verify that the gene set enrichment results were not an artifact of noise in the PLS modeling but rather had actual biological relevance, we turned to our permutation test-derived gene modules. In 1,000 permutation iterations, we destroyed the relation between gene expression and disease label. Thus, the extracted gene modules captured noise. We fed the gene loadings from these modules into our GSEA pipeline to verify the specificity of the empirically enriched terms.

### Gene network visualization

GO terms are organized in the form of a hierarchical tree [160] which can be downloaded here (OBO 1.4). This hierarchical organization often results in hundreds of hits from an enrichment analysis. One of the techniques widely used to crunch down this dense information is via network visualization [161, 162]. This technique can help identify broad groupings of terms based on a chosen parameter of interest, for example, shared genes between different terms.

For illustration purposes, we utilized Cytoscape [163] to create a structured network of disease-relevant GO-BPs identified by an enrichment analysis (cf. above). Each node in the network represented a GO BP hit. The edges were formed based on predefined relationships between nodes conditioned on shared genes and whether they were part of the same regulatory network. The resulting network layout (*yFiles.organic* layout) automatically clustered the enriched terms into biologically meaningful groups, allowing us to identify major functional themes that were shared between AD and PD.

### Differential GCN

As a complementary analytical pipeline, we sought to explore the transcription profile of the RNA-seq datasets in a top-down GCN analysis approach. Specifically, we started with candidate genes mapped to AD or PD GWAS risk loci. Using these genes as seeds, we created networks of correlated genes; that is, we identified groups of genes whose differential expression change between disease and control closely matched a seed gene. Seeded DGCNs have been previously used to identify regulatory changes in gene expressions across various conditions [48, 50]. By taking a contrastive approach between disease and control (differential), the effects of housekeeping genes could be limited, and the residual patterns of covariation could be attributed to the effects of a disease.

To identify our set of seed genes, we utilized the most recent GWAS studies that reported AD- or PD-associated risk genes significant at the whole genome level. We identified 108 GWAS hits associated with AD [44] and 129 GWAS hits associated with PD [45]. Out of these 237 genes, 5 were common (CTSB, WNT3, BCKDK, HLA-DQA1, and HLA-DRB1) between AD and PD, giving us 232 unique genes. Of these, a total of 164 genes (out of the 232 genes) were present across all considered transcriptomic datasets.

Next, we split each individual dataset into disease and control groups based on the diagnosis labels provided. We then subdivided each of these groups based on cell types. For each cell type $ct$, the co-expression vector for a single GWAS gene *i* with another gene *j* recorded in the snRNA-seq dataset was calculated as ${\tau }_b( {{e}_i,{e}_j} )$, where ${\tau }_b$ is the Kendall’s tau-b correlation metric, ${e}_i$ is the read count vector for $i\ \forall $ observations (nuclei), and ${e}_j$ is the read count vector of $j\ \forall $ observations (nuclei).

Evaluating ${\tau }_b$ across all recorded genes gave us $g_{ct,i}^{\mathrm{ AD}}\in R$, where *M* was the number of genes common to both AD and PD datasets (*M* = 16,936). Thus, each element of the matrix $g_{ct,\ i}^{\mathrm{ AD}}$ was a numerical value between −1 and 1, capturing the degree of correlation of gene *j* with GWAS gene *i*. Stacking the vectors for all GWAS genes gave us gene co-expression matrices $G_{C,\ ct}^{\mathrm{ AD}}\in{R}^{N \times M}$ and $G_{D,\ ct}^{\mathrm{ AD}}\in{R}^{N \times M},$ for the control and disease groups, respectively, where *N* was the number of GWAS genes (*N* = 164). From this, we formally computed the differential gene co-expression matrix for a single cell type $G_{ct}^{\mathrm{ AD}}\in {R}^{N \times M}$ as follows,


\begin{eqnarray*}
G_{ct}^{\mathrm{ AD}} = G_C^{\mathrm{ AD}} - {\mathrm{\ }}G_D^{\mathrm{ AD}}.
\end{eqnarray*}

Next, we systematically explored the mutual relationships between the DGCNs across different cell types, without discriminating them based on disease. To this end, we employed a hierarchical clustering analysis. Our goal was to probe for clusters of cell types that featured similar genome-wide co-deviation of gene transcription.

Concretely, we unraveled $G_{ct}^{\mathrm{ AD}}$ into a vector $u_{ct}^{\mathrm{ AD}}\in{R}^{1 \times NM}$, where $NM = 164\ \times 16,936 = 2,777,504,$ and combined them across 6 AD and 9 PD cell types to get $U\in{R}^{C \times NM}$, where $C = 15$. We computed a linkage matrix based on the Euclidean distance between 2 unraveled differential co-expression vectors for each cell type (*scipy.cluster.hierarchy.linkage*, parameters method = “average,” metric = “euclidean”). The linkage algorithm hierarchically clustered the 15 cell types, across AD and PD, with the cluster groups indicating cell types with the closest co-expression patterns. We visualized these clusters as a dendrogram in Python (*scipy.cluster.hierarchy.dendrogram*).

We refined our clustering-based qualitative approach to rigorously quantify the association between cross-disease DGCNs. Toward this goal, for the *i*th GWAS gene, we computed Kendall’s tau-b correlation metric (${\tau }_b$) between $g_{ct,\ i}^{\mathrm{ AD}}$ and $g_{ct,\ i}^{\mathrm{ PD}}$ giving us differential co-expression correlation matrix ${G}_i\in{R}^{6 \times 9}$. Each of these matrices encoded a similarity (−1 to +1; 0 being no association) between AD and PD co-expression networks for one gene.

To congregate this cross-association information encoded by 164 GWAS risk genes, we vertically stacked the unraveled matrix ${G}_i$ (unraveled to ${g}_i\in{R}^{1 \times 54}$) into a matrix $P\in{R}^{164 \times 54}$. Thus, *P*, in essence, captured multiple modes of information condensed into one matrix: (i) differential gene co-expression between disease and control, (ii) quantified similarity of the expression changes between AD and PD stratified at the level of cell types. Note that the GWAS genes acted as seeds not only for the disease in which they were identified but also for the other disease. We finally distilled *P* using PCA. This uncovered linear combinations of cross-disease cell type pairs that were most related in terms of their alterations to transcription in response to disease.

The number of significant latent factors that captured biologically meaningful information was determined using a principled permutation testing framework. In 100 permutation iterations, we randomly shuffled the unraveled correlation vector ${g}_i$, individually for each *i*, thus breaking the inherent meaningful patterns of covariation across cell type pairs. Across these 100 iterations, we fitted individual PCA models and computed the explained variances of the derived components. After comparing the permutation variances with our empirical component variances, we retained 4 latent factors as statistically significant based on the 5/95% CI (Fig. S8B). These 4 embeddings were by construction uncorrelated and rank-ordered, with the first component capturing the highest amount of variance in *P*.

We conducted a BS analysis on the extracted latent embeddings to formally assess the robustness of the cell type pairs that emerged as being closely associated with each other. Across 1,000 BS iterations, we sampled different rows (encapsulating all pairwise cell type co-deviations for a GWAS gene) with replacement to simulate a random seed gene collection that could have been sampled from the empirical population. We fitted individual PCA models to each of the thus-derived samples.

To handle the inherent order invariance (changed sequence, especially for later components with small explained variance) and reflection invariance (sign flipping of derived singular vectors) of PCA components [164], we applied the Jonker–Volgenant algorithm for component matching and Pearson’s correlation ($\rho $) for sign matching. The Jonker–Volgenant algorithm is a widely used technique [165] that can identify a one-to-one mapping of latent embeddings derived from 2 separate BS iterations. The similarity between a pair of components from 2 runs was scored using the cosine similarity (cf. above). Subsequently, we solved this optimization problem to maximize the similarity between component orderings from 2 runs across all pairwise combinations of the first 10 empirical and BS-derived PCA components (*scipy.optimize.linear_sum_assignment*, maximize = True). To align directionality, $\rho $ was computed between the empirical PCA component loadings and the BS-component loadings. For cases where $\rho $ was <1, the latent vector loadings were multiplied by −1.

Thus, in a complementary data-driven approach to our latent factor modeling, we identified potential combinations of cell types that had the closest associations of gene expression changes between disease and control states in AD and PD.

## Additional files

**Supplementary Figure S1:** Gene modules derived by supervised latent factor modeling perform above chance in out-of-sample disease classification. (A) Unbiased classification performance of PLS models fitted on individual cell types in Seattle-AD and Smajić-PD snRNA-seq datasets. Box plots represent clustered bootstrap performance results (*n* = 1,000). Each dot is one bootstrap iteration, where a random sample of donors was chosen with replacement from the bag of all donors. Disease and control donors were chosen in equal proportion. For the subset of donors, nuclei were chosen with replacement. The model performance was evaluated on the held-out donor nuclei. (B) Disease predictive power of PLS components. (Left) Kamath-PD, (right) ROSMAP-AD. Component-disease alignment was quantified using Pearson’s *ρ* between component’s disease prediction (*x*-score) and true disease representation (*y*-score). The black dot represents the model’s empirical *ρ*. Violins depict null distributions of *ρ* generated from label permutation of the empirical dataset (*n* = 1,000). Dashed lines represent 2.5/97.5% CI. (C) Kendall’s tau-b (τ_b_) correlation between ROSMAP-AD ROSMAP-AD gene modules (top) and Kamath-PD Kamath-PD gene modules (bottom). Each pairwise τ_b_ is statistically significant, exceeding the 2.5/97.5% confidence interval (CI) based on a 1,000-iteration permutation test. Darker colors indicate stronger association strengths, while larger square sizes indicate a greater number of shared genes. Strong correlation (absolute magnitude) is observed for the same cell type-derived gene modules. Significant cross cell type within the gene modules from the same disease correlations are also observed. (D) Stability and consistency of gene module correlations across AD and PD were tested in a split-half analysis. The initial datasets were bisected into 2 pairs of AD-PD sub-datasets. The histogram illustrates the distribution of Pearson’s rho obtained by comparing the overlap of cross-disease gene module pairs from split pairs (*n* = 500). This analysis underscored the robustness of our analytical pipeline, revealing a stable mean correlation coefficient of 0.92 ± 0.02. The mean is represented by a red dashed line.

**Supplementary Figure S2:** Gene modules do not necessarily represent cell subtypes. PHATE visualization of transcriptomes for all nuclei per each cell type. Each nucleus (dot) is colored based on the component with the highest disease-predictive score. No distinct separation of cells was observed, highlighting that the gene modules captured different modes of transcription changes that were pervasive across cells of a given type. AD- represents PHATE plots of ROSMAP-AD cell types. PD- represent PHATE plots of Kamath-PD cell types.

**Supplementary Figure S3:** AD-PD overlap replicated in an independent snRNA-seq dataset pair. Independent disease predicting PLS models were fitted for each Seattle-AD and Smajić-PD cell type. (A) Kendall’s tau-b (τ_b_) correlation between Seattle-AD and Smajić-PD gene modules. Darker colors indicate stronger association strengths, while square size indicates statistical significance (FDR corrected *P*-values). Black boxes represent statistically significant τ_b_ (exceeding 2.5/97.5% confidence interval) derived from permutation models fitted on the original datasets (*n* = 1,000). (B) Disease predictive power of PLS components. (Red) Smajić-PD cell type, (blue) Seattle-AD cell type. Component-disease alignment was quantified using Pearson’s *ρ* between component’s disease prediction (*x*-score) and true disease representation (*y*-score). The black dot represents the model’s empirical *ρ*. Violins depict null distributions of *ρ* generated from label permutation of the empirical dataset (*n* = 1,000). Dashed lines represent 2.5/97.5% CI.

**Supplementary Figure S4:** Situating AD and PD GWAS risk genes within AD and PD gene modules from an independent snRNA-seq dataset pair. (A–C) Mapping 164 GWAS genes (from both AD and PD GWAS) within Seattle-AD and Smajić-PD gene modules. Color intensity reflects the loading magnitude of each gene within the module. Only robust genes passing the 2.5/97.5% CI in a bootstrap permutation test are displayed. (D) Percentage of GWAS genes with robust disease-predictive weights across all gene modules (blue = AD gene modules, red = PD gene modules). Cellular localization of genes was observed; for example, APOE showed strong predictive loading in astrocytes, microglia, and oligodendrocyte precursor cell modules in AD.

**Supplementary Figure S5:** Gene Ontology terms shared between AD PD across cell types. (A) Number of shared GO terms between ROSMAP-AD and Kamath-PD gene module pairs is shown. Brighter colors represent a higher number of shared terms. White grids represent zero overlapping terms. Neuron gene modules in both AD and PD had the highest number of shared terms, closely followed by oligodendrocyte-related module combinations from PD. (B) Shared GO BP terms between ROSMAP-AD and Kamath-PD that are unique to cell type groups are shown. The inner circle denotes the cell type from AD, while the outer circle denotes the PD cell type. Each wedge represents a GO BP term shared between one AD and one PD gene module. Colors represent distinct cell types, and the numerical values indicate the PLS component in which the GO term was identified for that cell type. All GO terms shown are exclusive to the corresponding AD-PD module pair (that is, not occurring in any other module pair combination). This highlighted specific, non-redundant biological processes that converged across AD and PD in a cell type dependent manner.

**Supplementary Figure S6:** GSEA of gene modules identifies shared terms between Seattle-AD and Smajić-PD, an external validation snRNA-seq dataset pair. Shared GO terms across pairwise gene modules from AD and PD. Gene ontology (GO) biological process, molecular function, and cellular component terms are combined. Brighter color indicates a higher number of shared terms, while the white grid represents no overlap. (A) Total number of overlapping terms across all gene modules in AD (blue) or PD (red). The intersection region shows the number of shared terms. (B) Graph visualization of selected biological processes from the 2023 Gene Ontology database, focusing on both AD and PD. The largest subnetworks from the full GO hierarchical tree are shown. Node colors correspond to the disease label, and node size reflects the gene-set size. Core mechanisms such as immune processes, glucose metabolism, and ATP synthesis match the results from the ROSMAP-AD and Kamath-PD analysis arm. The top shared genes (robust genes per module are considered) across all AD-PD gene modules enriching for the representative terms are annotated. (C) Ranked GO BP terms based on their frequency of occurrence across AD-PD module pairs. Mitochondrial energy synthesis terms rank highest among all GO terms.

**Supplementary Figure S7:** Comparison between Seattle-AD- and Smajić-PD-associated differentially expressed genes. (A) Pairwise associations between Seattle-AD and Smajić-PD differentially expressed genes (DEGs) are shown (Kendall’s tau-b). For each cell type pair, statistical significance of association was assessed using a permutation test. Colored squares indicate significant associations (FDR < 0.05). Darker green (brown) denotes greater similarity (anti) between the log-fold change of significant DEGs from an AD-PD cell type pair. Square size is proportional to −log_10_(FDR). (B) Shared GO terms from gene set enrichment analysis of DEGs between Seattle-AD and Smajić-PD. Significant terms in AD or PD from GSEA analysis were assessed (FDR *q* < 0.1).

**Supplementary Figure S8:** Implicated genes are paralleled between the 2 analysis arms- DGCN and PLS. (A) The degree of similarity between PLS modules (Fig. 2A) and differential gene co-expression network (DGCN) is highlighted here. The fraction of robust genes shared between a PLS gene module and a GWAS-seeded DGCN, relative to the total number of genes in the GWAS-seeded DGCN for a given cell type, was used to quantify the degree of similarity. Left: ROSMAP-AD; Right: Kamath-PD. Each large grid represents a cell type combination (e.g., the top left grid shows the overlap of genes between excitatory neuron modules and excitatory neuron-derived co-expression networks). Lines within a larger grid correspond to a unique GWAS-seeded DGCN. Brighter colors indicate a higher degree of similarity, that is, a greater number of shared genes. Strong signatures along the diagonal suggested that similar gene cliques were derived for the same cell types using 2 orthogonal approaches, validating both analysis arms. (B) Distribution of explained variances for the first 12 principal components based on permutation analysis. Each vertical bar represents the mean explained variance for each PCA component across 100 permutations of the data. The error bars represent the 5/95% CI, indicating the range of variability under permutation. The empirical explained variance is marked with a red star for each component.

Supplementary_table_revision.xlsx.

## Supplementary Material

giag059_Supplemental_Files

giag059_Authors_Response_To_Reviewer_Comments_original_submission

giag059_GIGA-D-25-00403_Original_Submission

giag059_GIGA-D-25-00403_Revision_1

giag059_Reviewer_1_Report_original_submission Reviewer 1 -- 11/9/2025

giag059_Reviewer_2_Report_original_submission Reviewer 2 -- 12/29/2025

giag059_Reviewer_2_Report_revision_1 Reviewer 2 -- 4/28/2026

## Data Availability

The snRNA-seq PFC data originated from Mathys et al. [36] are available through Synapse under the doi 10.7303/syn184851755. The data are available under controlled use conditions set by human privacy regulations. The snRNA-seq MTG data originating from Gabitto et al. [37] are available through SEA-AD consortium’s web portal at SEA-AD.org. The snRNA-seq substantia nigra data originating from Kamath et al. [40] are available at the Gene Expression Omnibus (GEO) with accession number GSE178265. The snRNA-seq midbrain data originating from Smajić et al. [35] are available for download from the Gene Expression Omnibus (GEO) with accession number GSE157783. The snRNA-seq COPD dataset originating from Adams et al. [128] are available for download from the Gene Expression Omnibus (GEO) with accession number GSE136831. All custom analysis code is available on GitHub at https://github.com/dblabs-mcgill-mila/AD-PD-overlap-study. DOME-ML (Data, Optimization, Model, and Evaluation in Machine Learning) annotations are available via the DOME registry (accession v09w7a6a5r) [166]. All additional supplementary materials are available in the GigaScience repository, GigaDB [167].
