# Supplementary material for: Cell type transcriptomic modules reveal shared molecular mechanisms in Alzheimer’s and Parkinson’s disease
Source: Gigascience. 2026 May 21;15:giag059. doi: 10.1093/gigascience/giag059 (PMC13289754; doi:10.1093/gigascience/giag059)
Supplement: giag059_Supplemental_Files [file giag059_supplemental_files.zip › Supplementary.docx]

SUPPLEMENTARY FIGURES


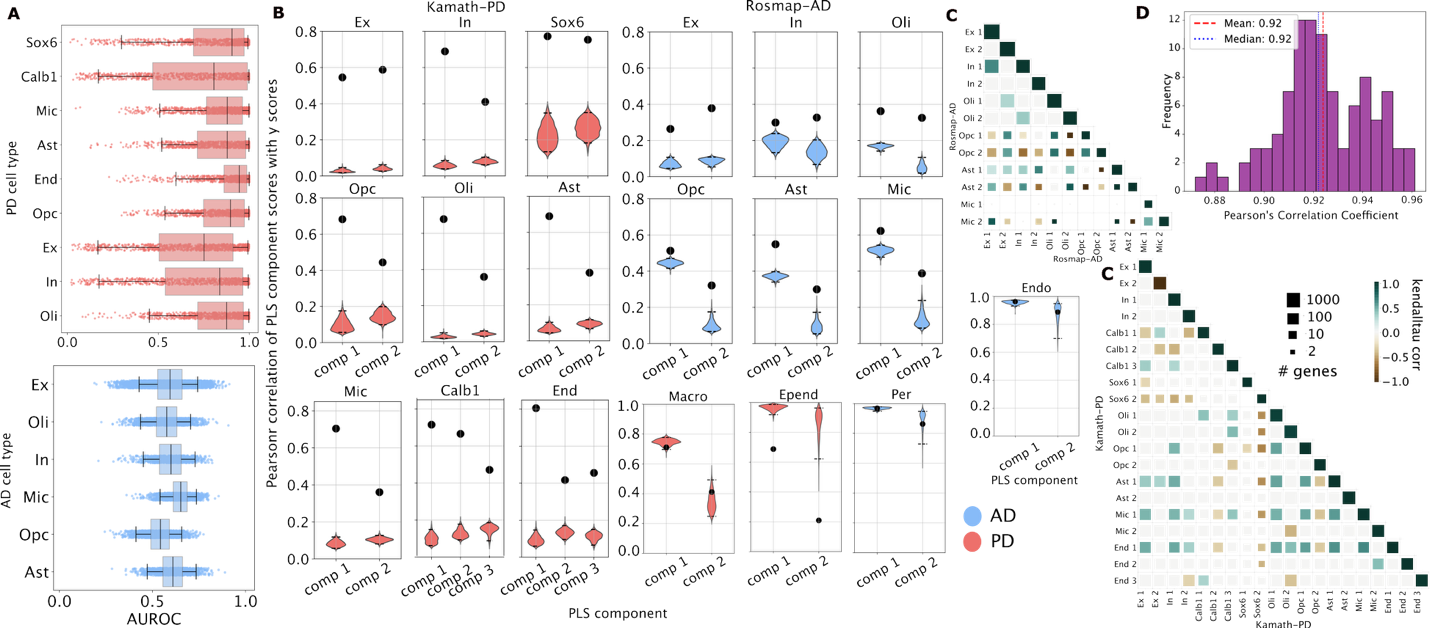


Fig. S1. Gene modules derived by supervised latent factor modeling perform above chance in out-of-sample disease classification.

**(A)** Unbiased classification performance of PLS models fitted on individual cell types in Seattle-AD and Smajić-PD snRNA-seq datasets. Box plots represent clustered bootstrap performance results (n = 1000). Each dot is one bootstrap iteration, where a random sample of donors was chosen with replacement from the bag of all donors. Disease and control donors were chosen in equal proportion. For the subset of donors, nuclei were chosen with replacement. The model performance was evaluated on the held-out donor nuclei. **(B)** Disease predictive power of PLS components. (Left) Kamath-PD, (right) Rosmap-AD. Component-disease alignment was quantified using Pearson’s *ρ* between component’s disease prediction (x-score) and true disease representation (y-score). The black dot represents the model’s empirical *ρ*. Violins depict null distributions of *ρ* generated from label permutation of the empirical dataset (n=1000). Dashed lines represent 2.5/97.5% CI. **(C)** Kendall’s tau-b (𝜏_b_) correlation between Rosmap-AD Rosmap-AD gene modules (top) and Kamath-PD Kamath-PD gene modules (bottom). Each pairwise 𝜏_b_ is statistically significant, exceeding the 2.5/97.5% confidence interval (CI) based on a 1000-iteration permutation test. Darker colors indicate stronger association strengths, while larger square sizes indicate a greater number of shared genes. Strong correlation (absolute magnitude) is observed for the same cell type derived gene modules. Significant cross cell type within the gene modules from the same disease correlations are also observed. **(D)** Stability and consistency of gene module correlations across AD and PD were tested in a split-half analysis. The initial datasets were bisected into two pairs of AD-PD sub-datasets. The histogram illustrates the distribution of Pearson's rho obtained by comparing the overlap of cross-disease gene module pairs from split pairs (n=500). This analysis underscored the robustness of our analytical pipeline, revealing a stable mean correlation coefficient of 0.92±0.02. The mean is represented by a red dashed line.


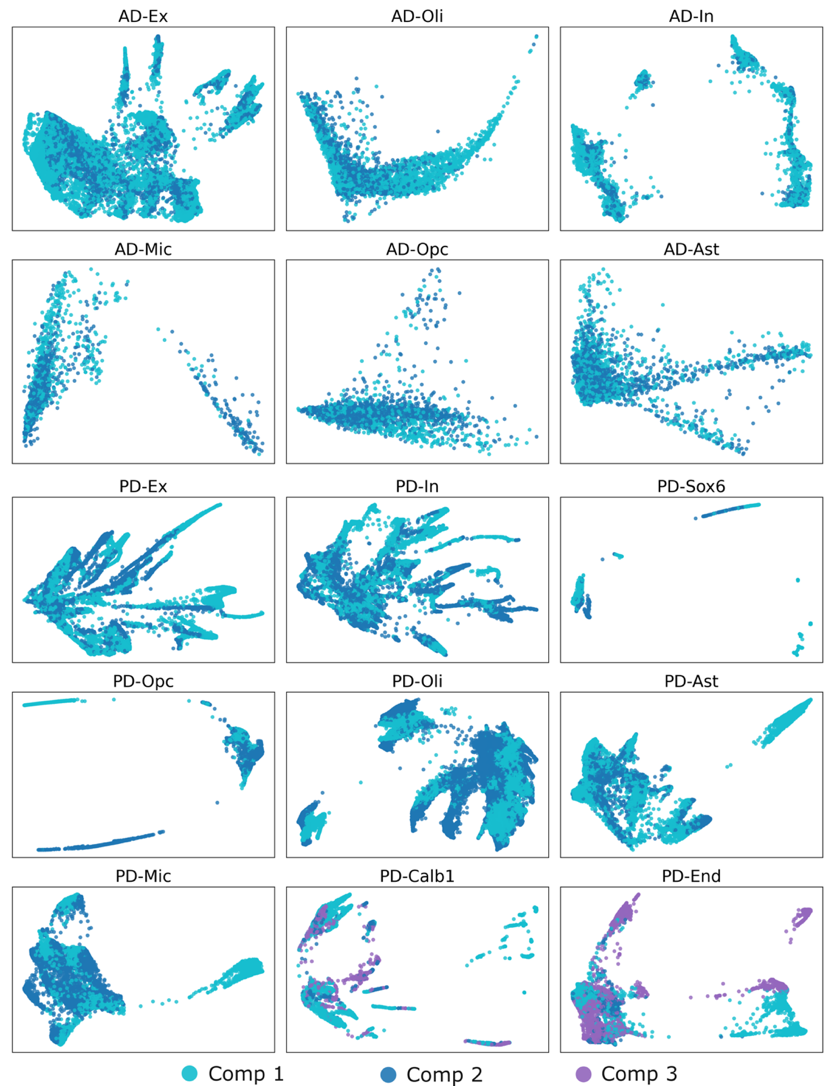


Fig. S2. Gene modules do not necessarily represent cell subtypes.

PHATE visualization of transcriptomes for all nuclei per each cell type. Each nucleus (dot) is coloured based on the component with the highest disease-predictive score. No distinct separation of cells was observed, highlighting that the gene modules captured different modes of transcription changes that were pervasive across cells of a given type. AD- represents PHATE plots of ROSMAP-AD cell types. PD- represent PHATE plots of Kamath-PD cell types.


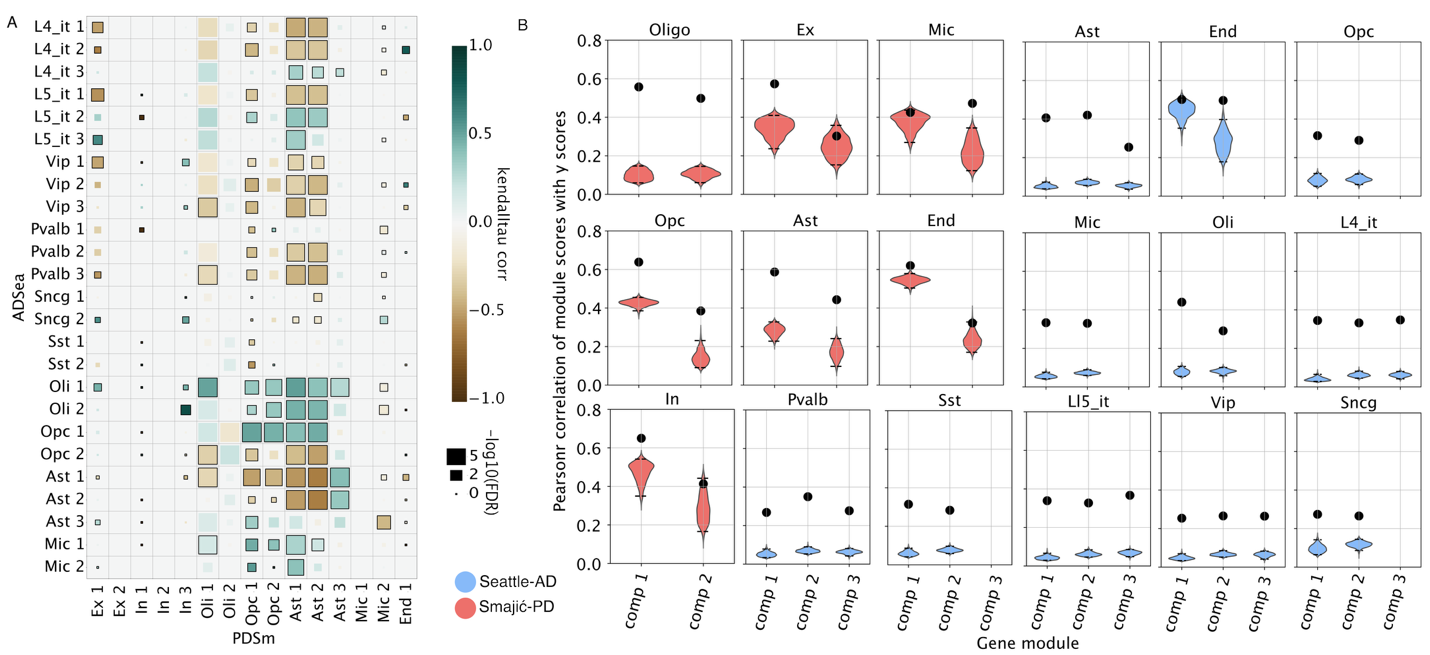
 Fig. S3. AD-PD overlap replicated in an independent snRNA-seq dataset pair. Independent disease predicting PLS models were fitted for each Seattle-AD and Smajić-PD cell type. (A) Kendall’s tau-b (𝜏_b_) correlation between Seattle-AD and Smajić-PD gene modules. Darker colors indicate stronger association strengths, while square size indicates statistical significance (FDR corrected p-values). Black boxes represent statistically significant 𝜏_b_ (exceeding 2.5/97.5% confidence interval) derived from permutation models fitted on the original datasets (n=1000). (B) Disease predictive power of PLS components. (Red) Smajić-PD cell type, (blue) Seattle-AD cell type. Component-disease alignment was quantified using Pearson’s *ρ* between component’s disease prediction (x-score) and true disease representation (y-score). The black dot represents the model’s empirical *ρ*. Violins depict null distributions of *ρ* generated from label permutation of the empirical dataset (n=1000). Dashed lines represent 2.5/97.5% CI.


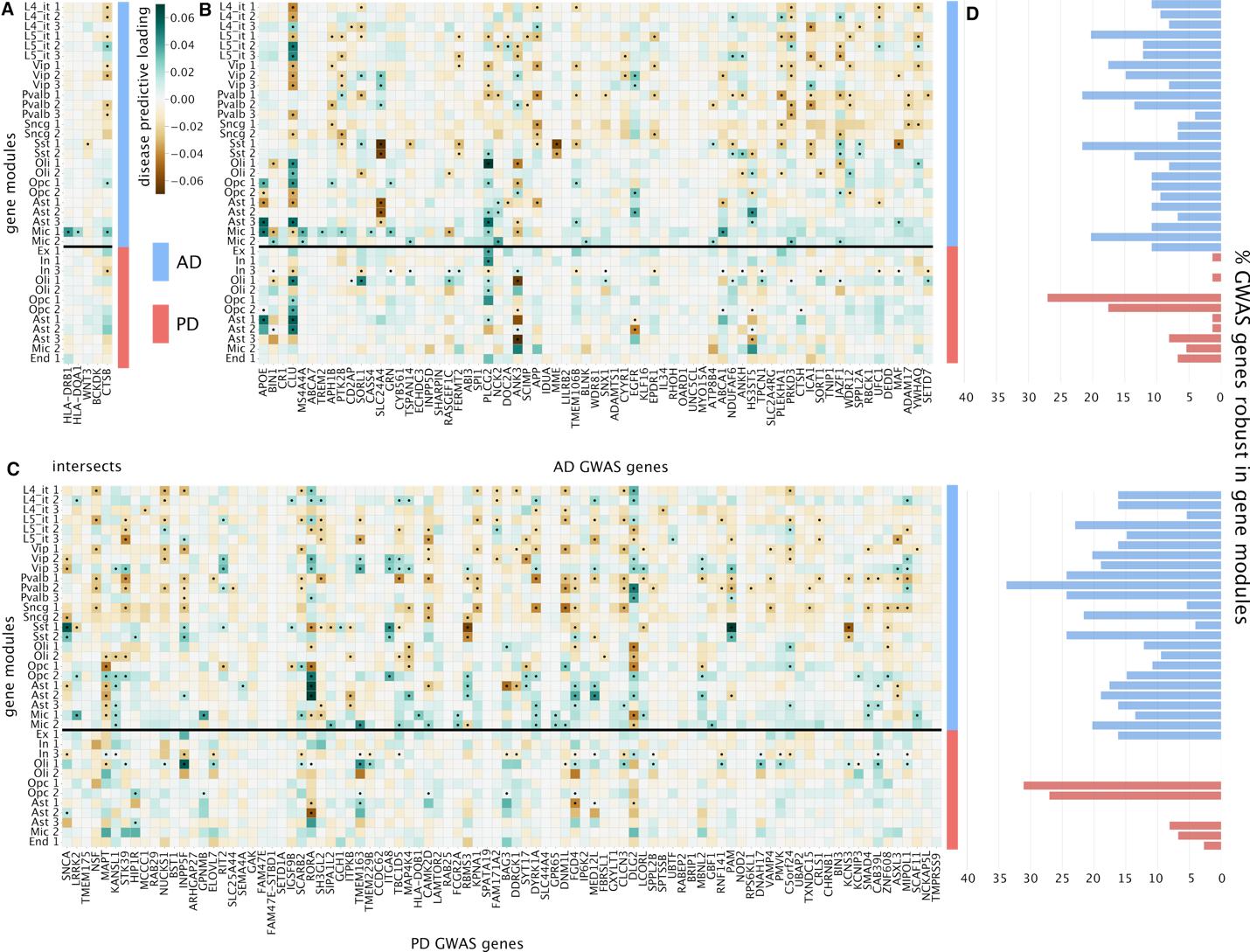


Fig. S4. Situating AD and PD GWAS risk genes within AD and PD gene modules from an independent snRNA-seq dataset pair.

**(A-C)** Mapping 164 GWAS genes (from both AD and PD GWAS) within Seattle-AD and Smajić-PD gene modules. Color intensity reflects the loading magnitude of each gene within the module. Only robust genes passing the 2.5/97.5% CI in a bootstrap permutation test are displayed. **(D)** Percentage of GWAS genes with robust disease-predictive weights across all gene modules (blue = AD gene modules, red = PD gene modules). Cellular localization of genes was observed; for example, APOE showed strong predictive loading in astrocytes, microglia, and oligodendrocyte precursor cell modules in AD.


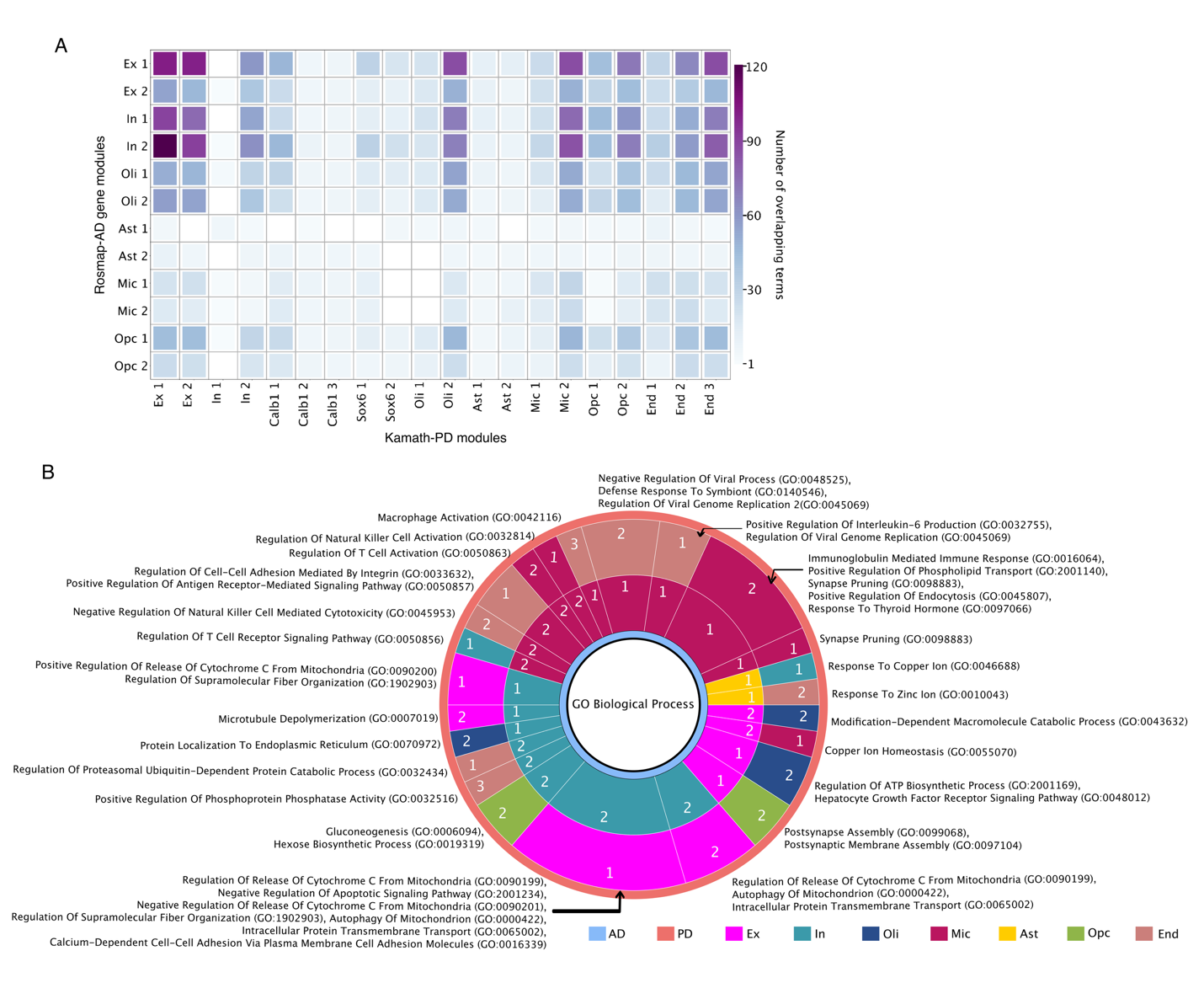


**Fig. S5. Gene Ontology terms shared between AD PD across cell types. (A)** Number of shared GO terms between Rosmap-AD and Kamath-PD gene module pairs are shown. Brighter colors represent a higher number of shared terms. White grids represent zero overlapping terms. Neuron gene modules in both AD and PD had the highest number of shared terms, closely followed by oligodendrocyte-related module combinations from PD. **(B)** Shared GO BP terms between Rosmap-AD and Kamath-PD that are unique to cell type groups are shown. The inner circle denotes the cell type from AD while the outer circle denotes the PD cell type. Each wedge represents a GO BP term shared between one AD and one PD gene module. Colors represent distinct cell types, and the numerical values indicate the PLS component in which the GO term was identified for that cell type. All GO terms shown are exclusive to the corresponding AD-PD module pair (that is, not occurring in any other module pair combination). This highlighted specific, non-redundant biological processes that converged across AD and PD in a cell type dependent manner.


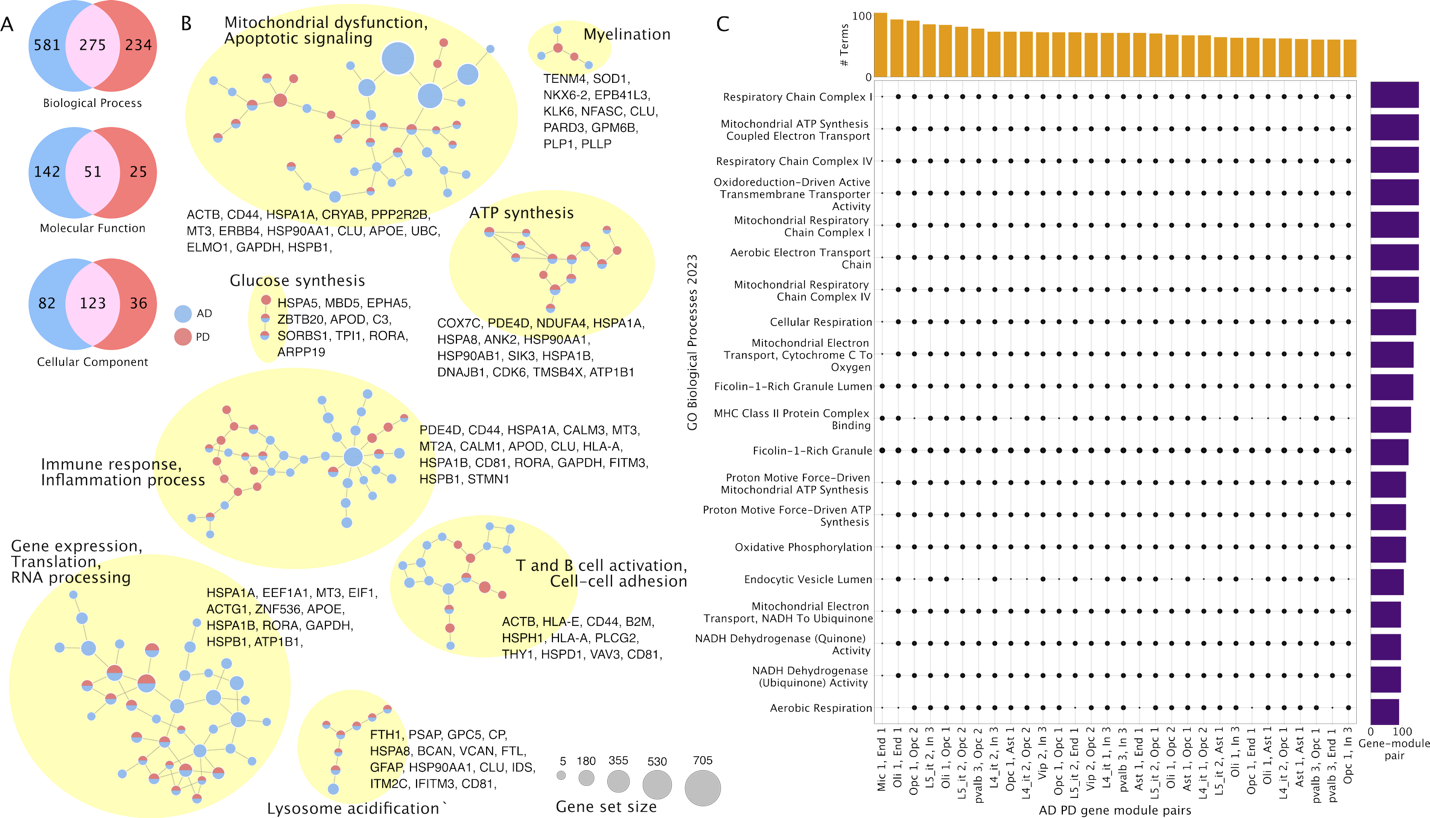


**Fig. S6. GSEA of gene modules identifies shared terms between Seattle-AD and Smajić-PD, an external validation snRNA-seq dataset pair.**

Shared GO terms across pairwise gene modules from AD and PD. Gene ontology (GO) Biological Process, Molecular function, and Cellular Component terms are combined. Brighter color indicates a higher number of shared terms, while the white grid represents no overlap. **(A)** Total number of overlapping terms across all gene modules in AD (blue) or PD (red). The intersection region shows the number of shared terms. **(B)** Graph visualization of selected biological processes from the 2023 Gene Ontology database, focusing on both AD and PD. The largest subnetworks from the full GO hierarchical tree are shown. Node colors correspond to the disease label, and node size reflects the gene-set size. Core mechanisms such as immune processes, glucose metabolism, and ATP synthesis match the results from the ROSMAP-AD and Kamath-PD analysis arm. The top shared genes (robust genes per module are considered) across all AD-PD gene modules enriching for the representative terms are annotated. **(C)** Ranked GO BP terms based on their frequency of occurrence across AD-PD module pairs. Mitochondrial energy synthesis terms rank highest among all GO terms.


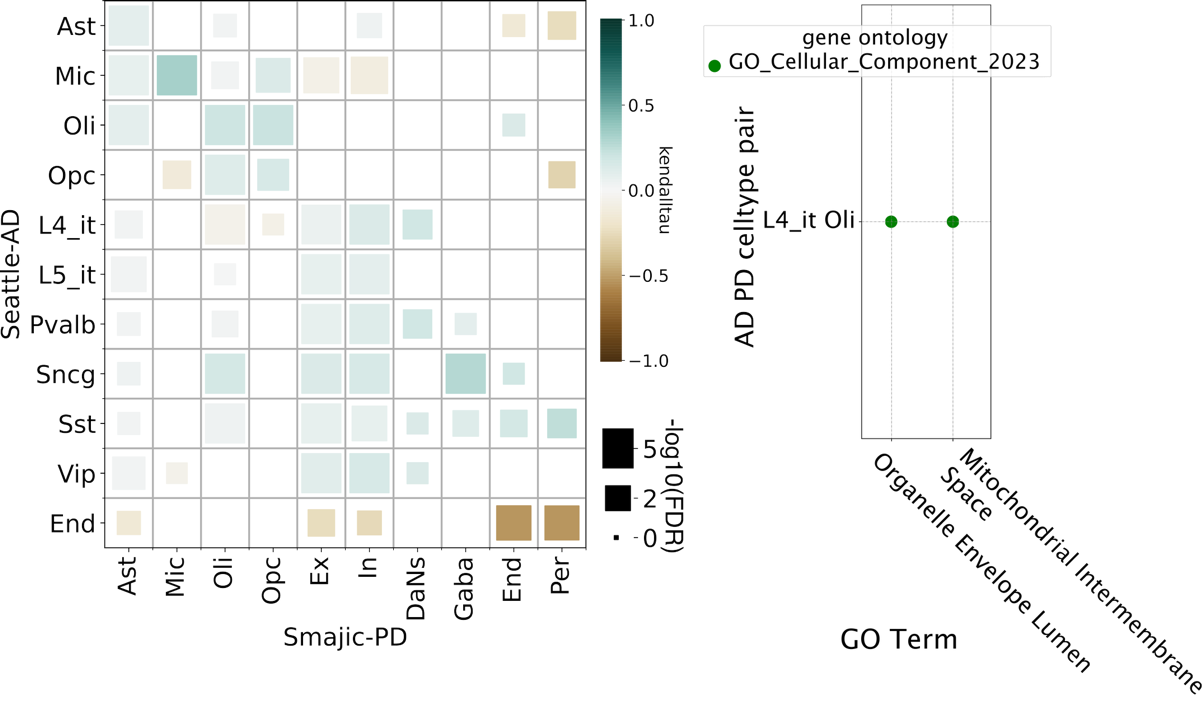


Fig. S7. Comparison between Seattle-AD- and Smajić-PD-associated differentially expressed genes. (A) Pairwise associations between Seattle-AD and Smajić-PD differentially expressed genes (DEGs) are shown (Kendall’s tau-b). For each cell type pair, statistical significance of association was assessed using a permutation test. Colored squares indicate significant associations (FDR < 0.05). Darker green (brown) denotes greater similarity (anti) between the log-fold change of significant DEGs from an AD-PD cell type pair. Square size is proportional to -log_10_(FDR). (B) Shared GO terms from gene set enrichment analysis of DEGs between Seattle-AD and Smajić-PD. Significant terms in AD or PD from GSEA analysis were assessed (FDR q<0.1).


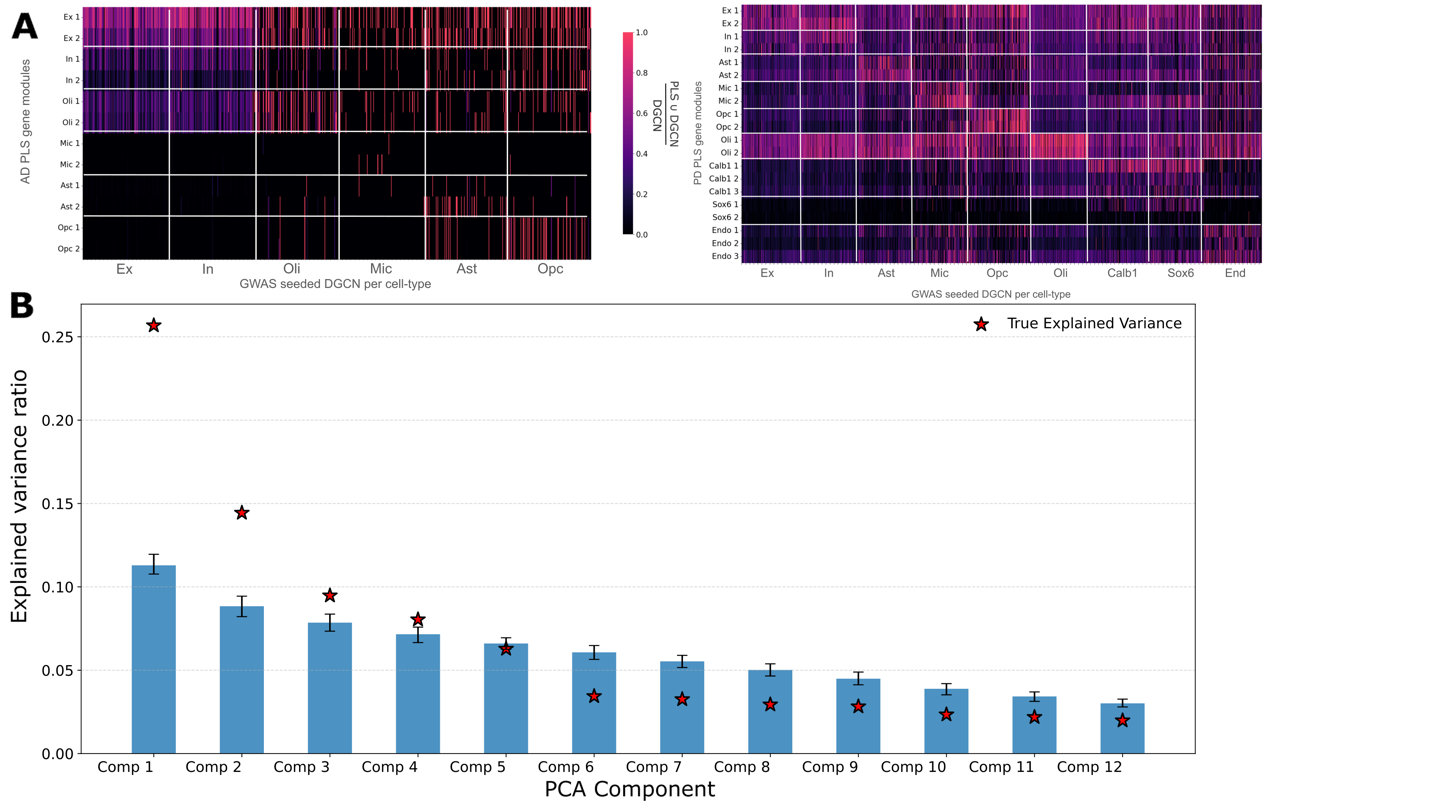


**Fig. S8. Implicated genes are paralleled between the two analysis arms- DGCN and PLS.**

**(A)** The degree of similarity between PLS modules (Fig 2A) and differential gene co-expression network (DGCN) is highlighted here. The fraction of robust genes shared between a PLS gene module and a GWAS-seeded DGCN, relative to the total number of genes in the GWAS-seeded DGCN for a given cell type, was used to quantify the degree of similarity. Left: ROSMAP-AD; Right: Kamath-PD. Each large grid represents a cell type combination (e.g., the top left grid shows the overlap of genes between excitatory neuron modules and excitatory neuron-derived co-expression networks). Lines within a larger grid corresponds to a unique GWAS-seeded DGCN. Brighter colors indicate a higher degree of similarity, that is, a greater number of shared genes. Strong signatures along the diagonal suggested that similar gene cliques were derived for the same cell types using two orthogonal approaches, validating both analysis arms. **(B)** Distribution of explained variances for the first 12 principal components based on permutation analysis. Each vertical bar represents the mean explained variance for each PCA component across 100 permutations of the data. The error bars represent the 5/95% CI, indicating the range of variability under permutation. The empirical explained variance is marked with a red star for each component.
