# Supplementary material for: Cell type transcriptomic modules reveal shared molecular mechanisms in Alzheimer’s and Parkinson’s disease
Source: Gigascience. 2026 May 21;15:giag059. doi: 10.1093/gigascience/giag059 (PMC13289754; doi:10.1093/gigascience/giag059)
Supplement: giag059_GIGA-D-25-00403_Original_Submission [file giag059_giga-d-25-00403_original_submission.pdf]

GigaScience

Cell type transcriptomics reveal shared genetic mechanisms in Alzheimer's and Parkinson's disease

--Manuscript Draft--

Manuscript Number:	GIGA-D-25-00403
Full Title:	Cell type transcriptomics reveal shared genetic mechanisms in Alzheimer's and Parkinson's disease
Article Type:	Research
Funding Information:	
Abstract:	Historically, Alzheimer's disease (AD) and Parkinson's disease (PD) have been investigated as two distinct disorders of the brain. However, a few similarities in neuropathology and clinical symptoms have been documented over the years. Traditional single gene-centric studies, including GWAS and differential gene expression analyses, have struggled to unravel the molecular basis for the observed pathological links between AD and PD. To address this, we tailor a pattern-learning framework to analyze synchronous gene co-expression at sub-cell-type resolution. Utilizing recently published single-nucleus AD (70,634 nuclei) and PD (340,902 nuclei) datasets from post-mortem human brains, we systematically extract and juxtapose disease-critical gene modules. Our findings reveal shared molecular programs across AD and PD that are not only detectable but also systematically localized to specific glial and neuronal cell types. In neurons, similar disrupted cytoskeletal dynamics and mitochondrial stress highlight convergence in key gene groups; AD and PD microglial modules share T-cell activation responses and synapse pruning mechanisms, oligodendrocytes highlight convergent dysregulation in myelin synthesis, and astrocytes in heavy metal processing. Our multi-module sub-cell-type approach offers unique insights into the molecular basis of shared neuropathology in AD and PD.
Corresponding Author:	Anwesha Bhattacharya<br>McGill University<br>Montreal, Quebec CANADA
Corresponding Author Secondary Information:	
Corresponding Author's Institution:	McGill University
Corresponding Author's Secondary Institution:	
First Author:	Anwesha Bhattacharya
First Author Secondary Information:	
Order of Authors:	Anwesha Bhattacharya
	Edward A. Fon
	Alain Dagher
	Yasser Iturria-Medina
	Jo Anne Stratton
	Chloe Savignac
	Jack Stanley
	Liam Hodgson
	Badr Ait Hammou
	David A Bennett
	Danilo Bzdok
Order of Authors Secondary Information:	

Powered by Editorial Manager® and ProduXion Manager® from Aries Systems Corporation

Additional Information:	
**Question**	**Response**
Are you submitting this manuscript to a special series or article collection?	No
**Experimental design and statistics**<br><br>Full details of the experimental design and statistical methods used should be given in the Methods section, as detailed in our Minimum Standards Reporting Checklist. Information essential to interpreting the data presented should be made available in the figure legends.<br><br>Have you included all the information requested in your manuscript?	Yes
**Resources**<br><br>A description of all resources used, including antibodies, cell lines, animals and software tools, with enough information to allow them to be uniquely identified, should be included in the Methods section. Authors are strongly encouraged to cite Research Resource Identifiers (RRIDs) for antibodies, model organisms and tools, where possible.<br><br>Have you included the information requested as detailed in our Minimum Standards Reporting Checklist?	Yes
**Availability of data and materials**<br><br>All datasets and code on which the conclusions of the paper rely must be either included in your submission or deposited in publicly available repositories (where available and ethically appropriate), referencing such data using a unique identifier in the references and in the "Availability of Data and Materials" section of your manuscript.	Yes

Powered by Editorial Manager® and ProduXion Manager® from Aries Systems Corporation

Have you have met the above requirement as detailed in our Minimum Standards Reporting Checklist?	
GigaScience has policies and guidelines in place for the use of generative AI-writing tools such as ChatGPT. If you have used such writing tools to assist with writing the manuscript this must be declared and cited in the text. Authors should not list AI-writing tools and other AI-assisted technologies as an author or co-author and should acknowledge that they are fully responsible for text generated or refined by AI-writing tools.<p><br><br>A summary of use (particularly in the introduction or among methods) needs to be included at the end of the paper, and the outputs should also be included as a supplementary file hosted in GigaDB or other open repositories. Please read our guidelines for more information. <p><br><br>By submitting to GigaScience, you are aware of the journal's AI-writing tools policy, and if you have declared use of such tools below, you have acknowledged this where appropriate in your manuscript and have made a summary of use and outputs available. <p><br><b>AI-assisted writing tools have been used in the preparation of this manuscript?	No

Manuscript Click here to access/download;Manuscript;Anwesha_NCB.docx

Cell type transcriptomics reveal shared genetic mechanisms in Alzheimer's and Parkinson's disease

Anwesha Bhattacharya[1,2,10], Edward A. Fon[3], Alain Dagher[4,5], Yasser Iturria-Medina[3,5,6], Jo Anne Stratton[3], Chloe Savignac[1,2,10], Jack Stanley[7,2,10], Liam Hodgson[8,2,10], Badr Ait Hammou[1,2,10], David A Bennett[9], Danilo Bzdok[1,2,8,10]*

[1]Department of Biological and Biomedical Engineering, McGill University; Montréal, Canada

[2]Mila - Quebec Artificial Intelligence Institute; Montréal, Canada

[3]Department of Neurology and Neurosurgery, Montreal Neurological Institute (MNI), McGill University; Montréal, Canada.

[4]Department of Psychology, MNI, McGill University; Montreal, Canada.

[5]McConnell Brain Imaging Centre (BIC), MNI; Montreal, Canada.

[6]Ludmer Centre for Neuroinformatics and Mental Health; Montreal, Canada.

[7]Quantitative Life Sciences, McGill University; Montreal, Canada.

[8]School of Computer Science, McGill University; Montreal, Canada.

[9]Rush Alzheimer's Disease Center, Rush University Medical Center; Chicago, USA.

[10]The Neuro, MNI, BIC, McGill University; Montreal, Canada.

*Corresponding author: danilo.bzdok@mcgill.ca

Abstract

Historically, Alzheimer's disease (AD) and Parkinson's disease (PD) have been investigated as two distinct disorders of the brain. However, a few similarities in neuropathology and clinical symptoms have been documented over the years. Traditional single gene-centric studies, including GWAS and differential gene expression analyses, have struggled to unravel the molecular basis for the observed pathological links between AD and PD. To address this, we tailor a pattern-learning framework to analyze synchronous gene co-expression at sub-cell-type resolution. Utilizing recently published single-nucleus AD (70,634 nuclei) and PD (340,902 nuclei) datasets from post-mortem human brains, we systematically extract and juxtapose disease-critical gene modules. Our findings reveal shared molecular programs across AD and PD that are not only detectable but also systematically localized to specific glial and neuronal cell types. In neurons, similar disrupted cytoskeletal dynamics and mitochondrial stress highlight convergence in key gene groups; AD and PD microglial modules share T-cell activation responses and synapse pruning mechanisms, oligodendrocytes highlight convergent dysregulation in myelin synthesis, and astrocytes in heavy metal processing. Our multi-module sub-cell-type approach offers unique insights into the molecular basis of shared neuropathology in AD and PD.

Introduction

Alzheimer's disease (AD) and Parkinson's disease (PD) are two of the most prevalent disorders in today's aging societies[1,2]. There has been intensive research with the grand aim of altering and ultimately halting the course of these diseases. Despite educated forecasts predicting significant advances by this decade[3], AD and PD remain challenging to unravel. This difficulty is compounded by a historically entrenched dichotomy that has limited transfer of research insights from one disease to the other. AD and PD are considered distinct entities due to differences in primary brain regions affected[4–7], age of onset, clinical progression, and treatment response. PD is notably responsive to therapeutics that do not affect cognition[8], and AD is without any "hard-currency" therapeutic to date[9].

This dichotomy continues to be reinforced by genomics and polygenic risk studies, which show minimal to no overlap of genes between AD and PD[10,11]. Indeed, aggregating prior findings, a *Neuron* review recently concluded, "There is intriguingly little overlap between the risk genes for AD and PD, providing genetic evidence for different disease onset and progression mechanisms"[12]. However, over half of PD patients show aggregates of tau[13] and around 30% of PD patients develop cognitive impairment with many going on to dementia[14]. Conversely, AD pathology and Lewy bodies co-occur more frequently than by chance, with Lewy bodies associated with fluctuating cognitive decline[7,15,16]. Further, the substantia nigra in AD can harbor tangles which are symptomatic of parkinsonism[17]. These hints raise the possibility of shared disease mechanisms between AD and PD at the molecular level[18,19].

Most studies investigating the genetic basis of the neuropathological overlap between AD and PD have primarily employed univariate approaches focusing on single genes[20–23]. However, gene expression occurs within tightly regulated environments where gene products interact in highly combinatorial ways[24–27]. Thus, the pathogenesis of neurodegenerative diseases is likely driven by molecular dysregulation within gene networks rather than by isolated gene anomalies[28,29]. Furthermore, the effects of dysregulated genes are not identical across different cell types. For example, in AD, APOE expression is increased in microglia and decreased in astrocytes and oligodendrocyte precursor cells[30,31]. Given this complexity, it is crucial to account for cell type heterogeneity when comparing AD and PD. Bulk RNA sequencing analysis has lacked the granularity to distinguish cell-type differences[32]. Recent advances in single-nucleus RNA sequencing (snRNA-seq) have enabled the resolution of transcriptional changes with high cell-type specificity. For example, large-scale single-nucleus transcriptomic studies in AD[33,34] and PD[4,35] have reported significant disease-associated transcriptional changes that are highly specific to certain cell type populations. However, to date, no studies have employed single-cell analyses combined with multivariate approaches to investigate both AD and PD [36].

In the present investigation, we systematically revisited the problem of identifying candidate molecular mechanisms that might overlap between AD and PD. Our unique approach focused on studying the concerted effects of genes at a sub-cell-type granularity. Comprehensive datasets from AD (70,634 nuclei from 48 post-mortem brains)[30] and PD (340,902 nuclei from 15 post-mortem brains)[37] allowed us to leverage advanced machine learning techniques to quantitatively characterize and compare the key disease-causing gene expression pathways underlying both diseases[38]. Enabled by a supervised multivariate model[39], we extracted and

compared several biologically interpretable gene modules within cell types from the AD and PD transcriptomes (16,936 protein-coding transcripts). Additionally, by focusing our inter-disease comparative analysis solely on disease predictive gene module compositions, we circumvented the potential batch effects of combining the raw transcriptomic datasets that could have compromised our analysis. By linking our gene programs to a catalog of biological pathways in the brain, we identified several shared cellular bases of neurodegeneration. We validated our main findings and conclusions using an independent AD and PD dataset pair[31,35]. The supervised pattern learning based comparative approach presented in this work introduces a statistically rigorous and unified framework for the automatic discovery of shared gene expression networks implicated across diseases.

Results

Rationale

Traditional approaches in single cell transcriptomics, such as DGE and univariate regression analyses, typically focus on individual genes in isolation. This offers a fragmented view of disease-related transcriptional changes. In contrast, we sought to interrogate two central hypotheses. First, gene co-expression modules, representing coordinated activity across the transcriptome, may provide a more integrative and biologically grounded framework to capture the molecular convergence between AD and PD. Second, we reasoned that disease progression withing each cell type is driven not by a single molecular axis, but by multiple distinct transcriptional programs. Therefore, comparing two diseases requires integrating these sub-cell type specific programs to capture the full complexity of their molecular convergence. Recent studies employing univariate frameworks have reported limited overlap in differentially expressed genes, primarily within select glial populations[1]. However, we hypothesized that such methods may fail to capture shared co-regulatory programs, particularly those present in neurons affected in both AD and PD. To address these questions, we deployed a supervised latent factor modeling framework, previously validated in AD, for its capacity to extract disease relevant modules from gene expression data[39]. This method combined the latent structure discovery strength of techniques like tSNE, UMAP, and variational autoencoders[40] while simultaneously being aware of valuable contextual information - the disease state. Here, we extend this approach in a rigorous comparative cross disorder setting. demonstrating for the first time that transcriptomic analysis can uncover shared molecular processes between AD and PD. This provides genetic support for the observed pathological overlaps between these two neurogenerative disorders.

Genetic correlation between AD and PD uncovered in multiple gene modules across major brain cell types

We explored the possibility of molecular overlap in AD and PD brains through transcriptional alterations captured in gene modules (groups of co-expressed genes). Our main analyses were conducted on a snRNA-seq AD dataset[30,41] (70,634 nuclei across 8 major cell types; referred to as ROSMAP-AD) and a PD dataset[37] (340,902 nuclei across 11 cell types; referred to as Kamath-PD). Our analytical framework employed partial least squares discriminant analysis (PLS-DA) to gain an overview of 16,936 protein-coding transcripts (Fig. 1; see Methods). In either AD or PD, we fitted cell type level PLS_{cell} models across nuclei from all donors in the dataset. This

yielded gene modules as latent projections of gene expression matrices that assisted in distinguishing cells of patients from controls[39]. Comparative assessments of these thus-derived gene modules highlighted shared genetic mechanisms between AD and PD that were more stable than expected by chance (Fig. 2A).

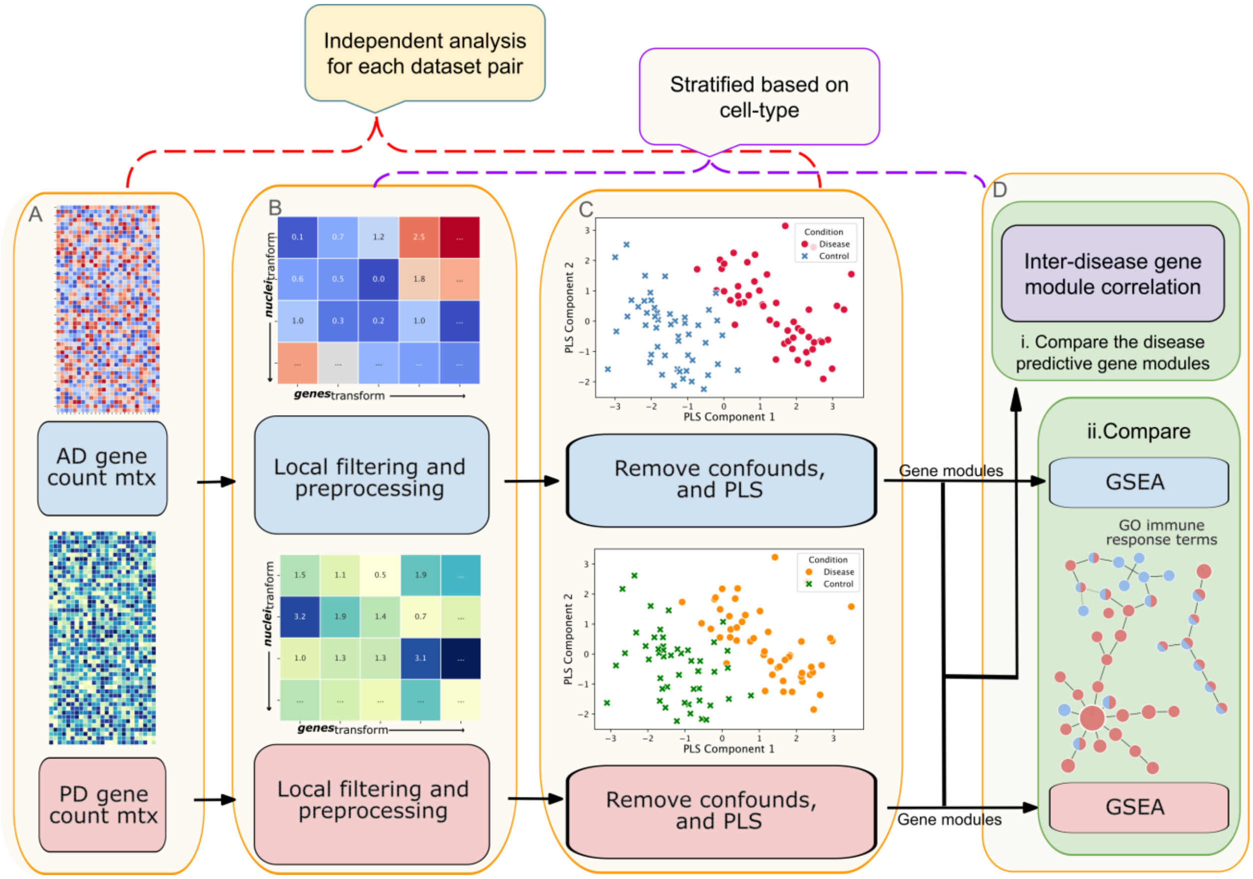

Figure 1. Workflow diagram to test for AD-PD overlap: bottom-up approach. Overview of workflow. (A) Single nucleus RNA sequencing (snRNA-seq) datasets for AD and PD were downloaded from public databases. Filtering and quality control of recordings were already done by source authors, as were cell type annotations. (B) Pre-processing step. Performed independently for each cell type, this step followed recommended guidelines for data transformation, removed lowly expressed genes, and corrected disease vs control class imbalance. (C) Gene module identification step. PLS discriminant analysis was performed per cell type to extract weighted gene lists, referred to as gene modules, that were disease predictive. (D) Comparative analysis. We aggregated the results from the two analysis arms and looked at the level of overlap using parallel methods – i. direct correlation of cross-disease gene module pairs and ii. gene set enrichment analysis to identify overlapping biological processes, molecular functions and cellular components. We discover significant molecular similarities between AD and PD across cell types. PLS, Partial least squares; AD, Alzheimer's disease; PD, Parkinson's disease; GSEA, gene set enrichment analysis; GO, gene ontology.

As a first step, we explored the transcriptomes of AD and PD individually to identify disease-specific modeling parameters. For each cell type, the number of modules that best distinguished diseased from healthy cells in unseen brain samples was treated as an hyperparameter in our model selection. We used a 10-fold cross-validation scheme on the stratified

dataset containing nuclei from one cell type to estimate this. Thus, in a data driven way, we found 2 gene modules per cell type were optimal for 6 cell categories in AD. In PD, the optimal number of gene modules was also determined to be 2 for each of the 9 major cell types, except for endothelial cells (3 modules) and CALB1 dopaminergic neurons (3 modules). Each gene module, on its own, represented combinations of several genes whose co-expression signature was associated with a disease state (AD vs. control or PD vs. control). The statistical significance of a module was assessed by comparing its empirical disease prediction performance to a null distribution of performance metrics derived from a label-shuffling permutation procedure (one-sided p-value < 0.05). As an illustration, Fig. 2B visualized the PLS_{Mic} loadings for the first gene module from ROSMAP-AD microglial cells.

All thus chosen PLS_{cell} models (6 AD and 9 PD models), exhibited robust above-chance out-of-sample accuracy in differentiating disease samples from control, as evidenced by the area under the receiver operating characteristic curve (AUROC) scores (Fig. S1A). Unbiased classification accuracy was estimated based on a patient-partitioned cross-validation scheme (see Methods). In the AD vs control group contrast, the predictive power measured by AUROC ranged highest for microglia (AUROC: 0.66 ± 0.06 std across partitions) to lowest for oligodendrocyte precursor cells (OPCs) (0.56 ± 0.08 std across partitions). For the PD vs control group contrast, the highest AUROC was for endothelial cells (0.89 ± 0.13 std across partitions), and the lowest was for excitatory neurons (0.69 ± 0.35 std across partitions). This strongly supported the role of gene modules in directly informing the disease phenotype across all examined cell types and conditions. Next, we inspected the 16,936 gene coefficients in each PLS_{cell} module (12 AD modules, 20 PD modules) that captured the contribution of a gene within the module. Specifically, using a bootstrap (BS) resampling technique (see Methods), we assessed which gene effects were statistically robust (zero not included in the 2.5/97.5% confidence interval (CI) of the BS distribution of each gene), and thus, robustly affected prediction outcomes. Each gene module yielded a variable set of genes distributed across the transcriptome (Fig. 2C).

To further explore the characteristics of the derived modules, we investigated if they represented cellular subpopulations which would be highlighted in a typical clustering algorithm. For a cell type, we assigned each observed nucleus to exactly one of its modules based on the component harboring the maximum PLS_{cell} score. In doing so, we were able to visualize the distribution of the gene modules assigned to the nuclei in a two-dimensional embedding space. The embedding space was derived using PHATE[42] from the ambient gene space containing all nuclei from the cell type (Fig. S2). No clear clustering among the components was observed. Thus, we concluded that our gene modules did not necessarily correspond to cellular subtypes. They likely corresponded more closely with different functional programs or biological states within a given cell type. That is, any given cell belonging to a type could exhibit several of the distinct expression programs, captured via our gene modules, to various continuous degrees.

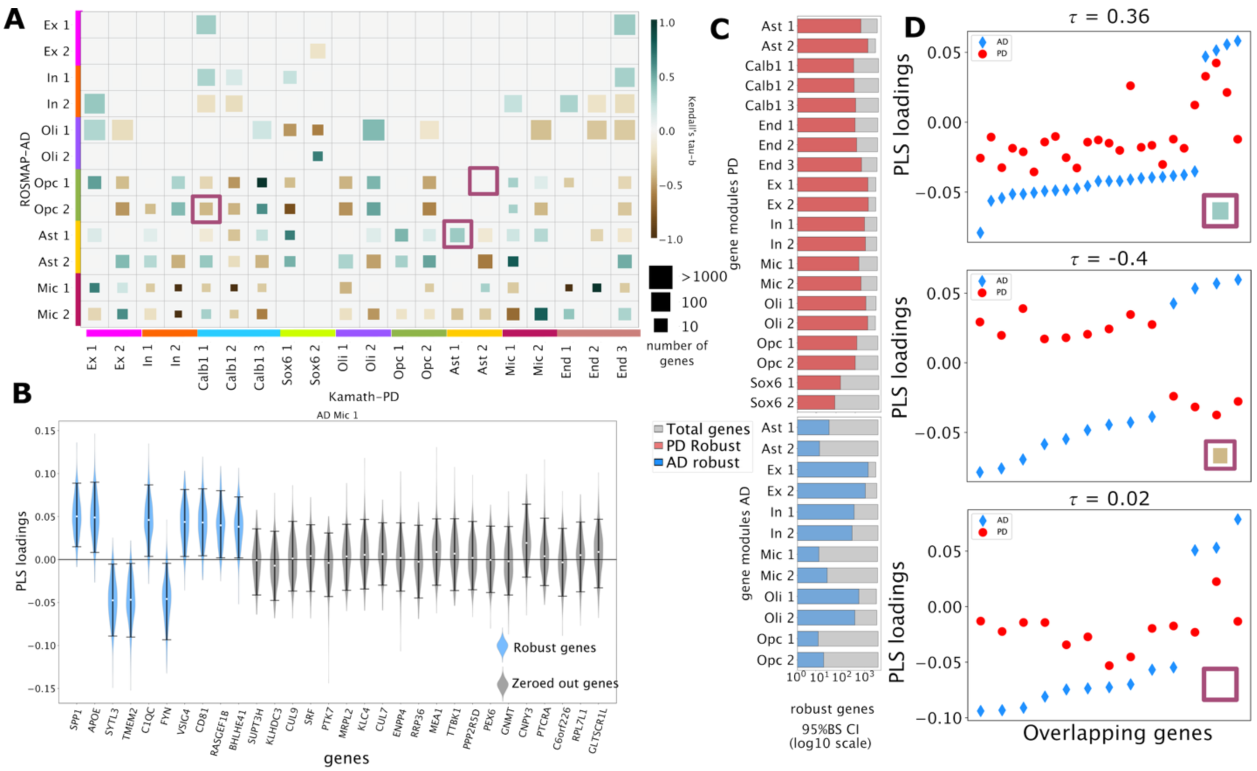

Figure 2. Convergence of disease mechanisms in Alzheimer's and Parkinson's disorders across cell-types when zoomed in on disease-predictive gene groups. We probed two snRNA-seq datasets (ROSMAP-AD, Kamath-PD) to explore the genetic overlap between AD and PD. By training 15 PLS models (one for each cell type in AD or PD), we extracted latent representations of gene expressions (gene modules) that maximized the separation between disease and healthy nuclei. (A) The coloured squares represents Kendall's tau-b (τb), quantifying the degree of association between AD-PD gene modules. Each pairwise τb is statistically significant exceeding 2.5/97.5% CI based on label shuffled permutation test (n=1000). Darker colors represent stronger genetic associations, indicating similar ranking trends of robust genes in the module pair. Square size represents number of robust genes shared between the two modules. Robust genes were assessed based on a 500-iteration bootstrap resampling scheme. Each colored bar (left vertical and bottom horizontal) represents a unique PLS model fitted on a cell type pre-identified in the dataset. The wide range of correlation strengths suggested that AD and PD shared significant genetic similarity spanning the sub-cell type landscape. (B) An illustration of the robustness assessment of genes in a gene module. The top 30 genes with the highest empirical predictive weights from the first PLS component in AD microglia are shown. Violins represent the loading distribution of genes derived from a bootstrap resampling scheme (n=500). Robust genes (blue) have non-zero predictive weights (2.5/97.5% confidence interval). Genes that do not meet this criterion are greyed out. APOE, a gene strongly associated with AD, was highlighted as a major disease predictor in this component. (C) The bars show the number of genes with robust disease predictive weight for each gene module. The colored portion of the bars denotes the number of robust genes in a module, while the grey region shows the total analyzed genes for that cell type. Different modules have different numbers of robust genes. (D) Select gene module correlations are visualized. Top, an example of a strong positive τ. Center, strong negative τ. Bottom, weak τ. Non-zero overlapping genes between a chosen gene module pair are represented on the x-axis. Ast, Astrocyte; Ex, Excitatory neuron; In, Inhibitory neuron; Mic, Microglia; Oli, Oligodendrocyte; Opc, Oligodendrocyte precursor cell; PLS, Partial least squares.

Importantly, the samples from AD and PD datasets were not merged. Instead, all analyses, so far, was conducted with parallel supervision targets (AD-control or PD-control). We also noted that our data-driven pipeline examined the entire transcriptome without assumptions about specific genes generally associated with AD or PD. In contrast, previous work using gene clustering or co-expression networks (which also aim to identify gene groups) often started by seeding the networks with apriori determined genes to build interaction graphs[43,44]. Moreover, our approach could assign a single gene as being relevant to multiple modules, a feature absent from most gene network analysis techniques[29].

We subsequently moved to our comparative analysis between AD and PD. To quantify the coupling between pairwise gene modules, we used Kendall's tau-b (τ_b) metric. It calculated the degree of similarity between two vectors of PLS_{cell} predictive weights (not the gene expression measurements; Fig. 2D) of the overlapping robust genes from an AD-PD gene module pair. By comparing the empirical correlation strength with a permutation-derived null distribution (obtained by correlating modules derived from a label-shuffled dataset), we identified the module pairs with significant associations (empirical τ_b more extreme than 2.5/97.5% CI of the null distribution). To further test the generalizability of our comparison model, we randomly divided our original datasets from AD and PD into two non-overlapping subset pairs (split-half test; see Methods) and observed strong correlations (Pearson's rho = 0.92 ± 0.02 standard deviation) across different realizations of the partitioned, but otherwise identical, analysis (Fig. S1C).

As the most important results of our investigation so far, we noted strong and robust associations among inhibitory neuron modules, among excitatory neuron modules, and among oligodendrocyte modules from AD and PD (p-value < 0.05, n=1000 iterations, label shuffle permutation test; Fig. 2A; table S1). Across all pairwise combinations, the strongest correlation (robust to label shuffle permutation test, n = 1000 iterations, p-value < 0.05) was found between the first oligodendrocyte module (represented as Oli 1) module from AD and the second oligodendrocyte module (Oli 2) from PD (represented as Oli 1_Oli 2; $\tau_{b, abs}$= 0.44, number of shared genes = 1351, p-value = 3.2e-27). We noted that in a parallel analysis involving DGE (described in a later section), a cross-disease cell-level comparison (based on the log-fold change between disease and control) reported a maximum association of $\tau_{b,abs}$= 0.20. In contrast, our native whole-transcriptome analysis described multiple sets of module combinations as having high degrees of similarity.

In addition to Oli 1_Oli 2, several other pairs (Oli 1_Ex 1, Oli 1_Ex 2, In 2_Ex 1) featured $\tau_{b, abs}$ > 0.3, and shared over 500 robust genes. Notably, dopaminergic neurons from midbrain tissue samples in PD, CALB1, and SOX6, also showed significant similarities with AD-critical gene programs from neurons, oligodendrocytes, and OPCs (In 1_Calb1 1, $\tau_{b, abs}$ = 0.32, p-value = 2.4e-6; Opc 2_Calb1 1, $\tau_{b, abs}$ = 0.41, p-value = 5.7e-2). On the flipside, inhibitory neuron module pairs between AD and PD showed the least overlap between each other. This suggests potentially different genetic contributions of these cell types to AD and PD disease mechanisms. Congruently, AD-derived astrocyte, microglia, and OPC gene modules demonstrated strong correlations with most PD modules. Particularly, Mic 2 from AD featured correlation strengths of $\tau_{b, abs}$ = 0.78 Mic 2 from PD (p-value = 1.7e-4). Similarly, Ast 2_Ast 2 featured $\tau_{b,abs}$ = 0.62, p-value = 1e-5. To assess how the cross-cell type module associations compared within a single disease, we looked at the correlation effect sizes (statistically significant under a label shuffled permutation test, p-

value < 0.05; Methods) across gene modules in a Rosmap-AD versus Rosmap-AD comparison and a Kamath-PD versus Kamath-PD (Fig. S1D). The presence of robust association signatures between gene modules from different cell types within the same disease provided additional confirmation of the cross cell type module level overlaps observed between AD and PD. Thus, these findings located significant sub-cell level genetic associations between AD and PD-relevant gene modules with the strongest associations among combinations of neuron and oligodendrocyte modules from AD and PD.

To replicate our primary findings, we examined the transcriptomic space using a different pair of snRNA-seq datasets related to AD (Seattle-AD) and PD (Smajić-PD), with independent cohorts. We repeated all main analyses and derived sub-cell-level gene modules from these datasets (detailed cohort and sample description in Methods). As before, our external validation analysis also revealed significant gene module overlaps between AD and PD, scattered across different cell types (Fig. S3A; table S2). Oligodendrocyte module pairs from AD and PD, once again, took center stage with strong associations (p-value<0.05, n=1000 iterations, label shuffle permutation test) between each other as well as with modules from neurons, astrocytes, and microglia. The strongest association was observed between oligodendrocytes from Seattle-AD and astrocytes from Smajić-PD (Ast 1_Oli 1, $\tau_{b, abs}$= 0.53, p-value = 4.2e-21) followed closely by Oli 1_Oli 1 ($\tau_{b, abs}$= 0.51, p-value = 2e-88). Strong significant associations were also observed between different combinations of neuron and glial cell-derived modules (Ast 1_L5_it 1, $\tau_{b, abs}$= 0.36, p-value = 5e-9; Mic 1_Oli 1, $\tau_{b, abs}$= 0.17p-value = 3.9e-6). As in the primary analysis, inhibitory neuron-derived modules showed sparse similarities between AD and PD. Overall, our external validation of shared genetic signatures between AD and PD supported the conclusion that the primary findings were not artifacts of dataset-specific factors such as transcriptomic platform, cohort composition, or brain region selection.

Taken together, these findings revealed significant molecular overlap between AD and PD at sub-cell resolution. The gene module associations extended between and across distinct AD-PD cell types. The degree and specificity of these overlaps varied among cell types, with oligodendrocyte and neuron-based gene module combinations in AD and PD signaling the strongest similarities. Our external validation experiments replicated these core findings in independent datasets, further substantiating our conclusions regarding the shared genetic architecture between these neurodegenerative diseases.

Cell type-specific gene modules reveal GWAS derived genes as key predictors of disease

We contextualized our gene modules post-hoc to understand their relationship with known gene variants from genomic studies. Drawing from the most recent GWAS that reported AD[45] or PD[46], we looked at 164 genes (table S3; see Methods) and located them within our gene modules. Most GWAS genes showed significant disease-predictive loadings in at least one gene module (Fig. 3). The top gene module harboring the most GWAS genes in AD was Ex 1 with 25.6% of AD GWAS genes present, followed by Ast 1 with 24.3% GWAS genes present. In PD, the top modules were Oli 1 with 58.9% PD GWAS genes followed by Ex 2 with 42%. Moreover, we found clear cell type localization of these genes within this modular framework. For example,

APOE, a broadly accepted AD risk gene, showed robust predictive loadings in the Ast 1 and Mic 1 derived from AD. Similarly, LRRK2, one of the major PD risk genes, was implicated in distinct PD-related gene modules. The strongest effect was observed in PD Mic 2. This cellular localization further corroborated our gene module modeling approach.

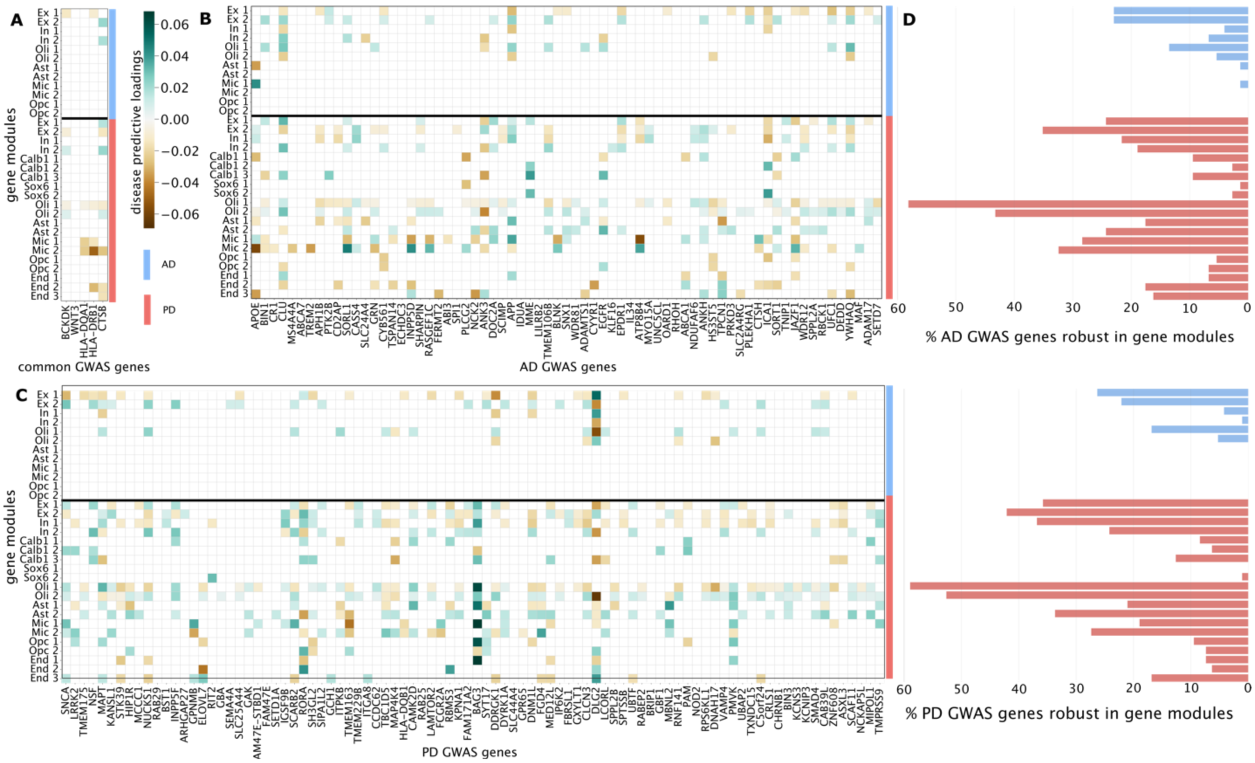

Figure 3. Cell type-specific gene modules reveal GWAS-linked genes as key disease predictors. We mapped the contribution of 164 candidate GWAS genes (combined from the largest AD GWAS study and PD GWAS study) within our gene modules. The color saturation represents the strength of the predictive weight of a gene in a module as deduced by the PLS model (robust loading according to bootstrap iteration scheme passing 2.5/97.5% CI). **(A-C)** Genes grouped based on the nominating disease. Group (A) harbors 5 common genes nominated independently in both AD and PD. (B) Shows the genes implicated in AD GWAS. (C) Shows genes implicated in PD GWAS. **(D)** The bars on the right summarize the connection of a module with GWAS mapped genes. The lengths indicate the percentage of GWAS mapped genes with robust loading in the module (blue = AD modules, red = PD modules). Cell-level localization of several key risk genes was observed. For example, APOE, a major AD risk gene, showed strong predictive loading in AD microglial and astrocyte modules. Similarly, SNCA had strong predictive signals in PD-based oligodendrocytes, microglia, excitatory neurons, and CALB1 dopaminergic neurons. This corroborated the gene compositions in our modules with previous complementary research. Notably, as a novel find, several genes which were implicated as being AD relevant in GWAS, had robust predictive loadings in PD gene modules and vice versa. For example, APOE, APP, and other AD genes mapped to risk loci had robust predictive weights in PD modules. Likewise, SNCA, MAPT, and other PD risk genes had robust predictive weights in AD modules. Ast, Astrocyte; End, Endothelial; Per, Pericyte; Ex, Excitatory neuron; In, Inhibitory neuron; Mic. Microglia; Oli, Oligodendrocyte; Opc, Oligodendrocyte precursor cell; GWAS, Genome-wide association study.

Next, we analyzed the effects of GWAS genes from one disease within gene modules linked to the other neurodegenerative disease. In other words, we investigated whether genes

mapped to AD GWAS risk loci were highlighted in any PD modules and vice versa. Among the AD GWAS genes, top genes, including APOE, APP, BIN1, and CLU (based on p-value from GWAS [45]) had robust disease predictive loadings in gene modules associated with PD. Concretely, APOE had the strongest predictive weight in PD Mic 2 (gene loading = -0.06, max absolute loading for any gene in this module was 0.07, absolute rank of this gene = 23), APP's maximum loading was in PD Mic 1 (loading = 0.04, max_{abs} = 0.07, rank = 262), BIN1 was found in PD Calb1 2 (loading = -0.02, max_{abs} = 0.04, rank = 1626), and CLU had the strongest robust weight in PD Oli 2 (loading = 0.03, max_{abs} = 0.08, rank = 405). It is important to note that these genes were not the highest-ranked in the PD modules. In other words, they were not the primary disease-indicative genes (rank = 1). Instead, these genes likely played supporting roles that become apparent only within the context of gene modules.

Conversely, we made similar observations for previously reported PD GWAS genes[47] within our AD modules. SNCA had strong predictive loading in AD Ex 1 (-0.03, max_{abs} = 0.07, rank = 397), MAPT in AD In 1 (-0.02, max_{abs} = 0.07, rank = 979), and TMEM175 in AD Ex 1 (-0.02, max_{abs} = 0.07, rank = 1869). Interestingly, while the genes implicated in AD GWAS had very clear cell type localizations, the risk genes associated with PD tended to be distributed across modules implicating multiple cell types, with particularly high effect sizes in neuronal modules. This observation aligned with a previous genomic enrichment study showing that PD risk loci are not confined to specific cell types. Instead, they are associated with broad cellular processes observable across multiple cell types[48]. Collectively, we observed that the bona fide GWAS genes not only tracked the disease they were implicated in, but known GWAS hits also proved relevant in gene modules associated with the other disease.

In our external validation analysis, GWAS-mapped genes exhibited consistently strong disease-predictive loadings across gene modules derived from the Seattle-AD and Smajić-PD datasets. Notably, all key observations from our primary dataset analysis could be replicated (Fig. S3 B-E). First, the genes tracked disease-corresponding modules; Microglia and astrocyte AD modules faithfully tracked APOE (interestingly, we observed robust predictive loading for APOE in Seattle-AD OPC modules[30]). Similarly, PD GWAS-mapped genes, such as SNCA and LRRK2, were tracked by PD predictive modules. Second, mirroring our primary dataset pair, several GWAS-mapped genes were enriched in modules associated with the opposite disease (for instance, APOE tracked PD modules and SNCA tracked AD modules), further reinforcing the presence of cross-disease genetic convergence.

We thus successfully contextualized genes mapped to established GWAS risk loci within the scope of gene modules. This led us to identify significant effects of these genes (within the context of functionally bound modules) beyond the disease in which they were initially reported in. For example, not only did we validate the significant impact of APOE in AD microglia and astrocyte-specific modules, but we also observed robust disease-predictive signatures in PD modules corresponding to microglia, astrocytes, and CALB1 DA neurons. These cross-disease results were consistently replicated in independent validation datasets, reinforcing our primary findings. Crucially, such observations were only possible through our approach, which evaluates the joint contribution of statistically meaningful gene sets to disease status. Thus, although APOE may not show a strong univariate effect in PD, it plays a significant role when considered within the context of co-expressed genes in gene modules.

Overlapping biological functions between AD and PD gene modules

To explore the biological relevance of the gene modules, we conducted comprehensive gene set enrichment analyses (GSEA) across the modules identified for each cell type in our latent factor analysis. We carried out this contextualization separately for AD and PD, analogous to our previous analysis steps. Notably, in our analysis, a single gene can contribute significantly to a disease via multiple modules within the same cell type; this, in turn, enabled us to capture its effect on complementary, co-regulated pathways. We screened the widely relied upon gene ontology databases (GO 2023) corresponding to three complementary domains - biological processes (BP), molecular functions (MF), and cellular components (CC). We focused on GO, as collectively, they cover the largest fraction of the genome. Each gene module was enriched in several significant GO terms (Benjamini-Hochberg FDR<0.05). Across all gene modules, GO enrichment for AD highlighted 196 BP, 35 MF, and 73 CC terms. For PD, we obtained 561 BP, 103 MF, and 163 CC terms.

We next examined if any enriched terms were shared between AD and PD. In total, 122 BP terms overlapped between AD and PD from a universe of 27,993 GO BP terms, 26 MF out of 11,271, and 65 CC out of 4,039. These overlaps highlighted the non-random and specific nature of our gene modules (Fig. 4A). The greatest number of GO term overlaps emerged in neuron-neuron AD-PD module pairs and pairs involving oligodendrocytes and oligodendrocyte precursor cells (>160 terms per module pair, BP, MF, and CC combined; see Fig. 4B, also note Fig. 4C horizontal axis). Additionally, both AD and PD microglial gene modules shared on average 20 common term hits with other gene modules. In contrast, module pairs involving astrocytes featured lower overlaps, with the maximum number of shared terms being 13 between AD Ex 1 and PD Ast 1.

By sorting all biological processes based on their frequency of shared occurrence across AD-PD module pairs, we identified the top 30 terms. These were related to protein translation, cellular respiration, and mitochondrial energy synthesis (Fig. 4C). To further summarize the terms systematically, we devised a visualization procedure to obtain a synoptic summary of the overarching biological processes. For this, we leveraged the predefined hierarchical tree structure of GO terms[49]. Each node in the tree represented a GO term, and the edges described shared genes or functional relationships determined by careful experimentation. We subsetted this tree to 841 enriched GO BP terms derived across all AD and PD modules. Drawing these out in a network analysis tool, we extracted biological themes shared between AD and PD (Fig. 4D): protein synthesis and misfolding, immune response, lysosome acidification, glucose metabolism, mitochondrial dysfunction, and myelination.

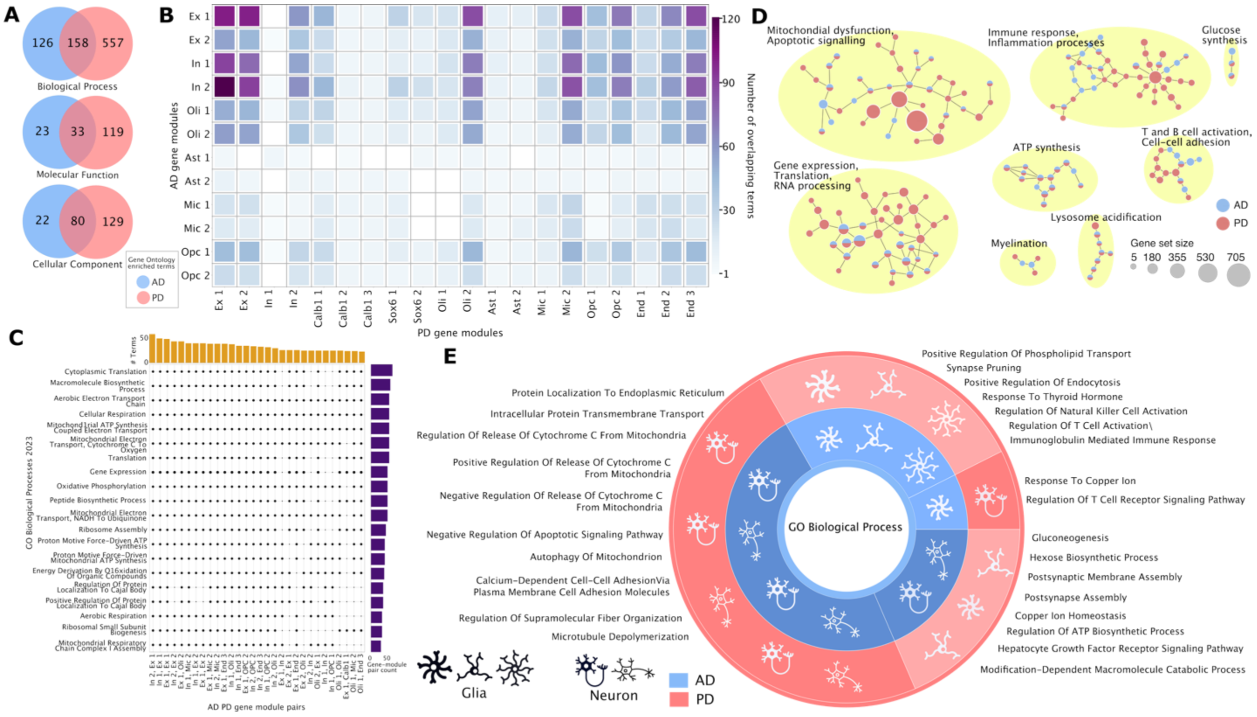

Figure 4. Gene ontology terms mapped to gene modules are shared between AD and PD. We performed gene set enrichment analysis on the gene modules derived from our previous analysis. For each gene module in AD or PD, we mapped the ranked genes (based on predictive weights) to terms in the gene ontology (GO) 2023 database. This helped ground the gene modules to pre-curated biologically relevant ontologies. **(A)** Overlapping terms from GO — Biological Process, Cellular Component, Molecular Functions. The venn diagrams depict the number of unique and shared terms across all gene modules, grouped by AD or PD. **(B)** Number of shared GO terms between every AD-PD gene module pair is shown. Brighter colors represent a higher number of shared terms. White grids represent zero overlapping terms. Neuron gene modules in both AD and PD had the highest number of shared terms, closely followed by oligodendrocyte-related module combinations from PD. **(C)** Zooming in on a few key terms summarizing the most frequent shared terms and gene-module combinations. Solid black dots indicate that a term (in the vertical axis) is enriched in the corresponding gene-module pair (in the horizontal axis). The bar plots on the horizontal axes are counts of the total number of terms common between AD and PD for the gene-module pair (arranged in decreasing order of term counts, first 30 pairs shown). Bar plots on the vertical axis represent the total number of cross-disease gene-module pairs that a term is present in (arranged in decreasing order of gene-module pair counts, first 10 terms shown). **(D)** Graph visualization of select biological processes across AD and PD from GO. Nodes are colored based on disease label and node size indicates the gene-set size. Groups names summarize the main themes from the terms in the group. This zoomed-out view highlighted key biological processes involved in both AD and PD. **(E)** Shared GO BP terms between AD and PD that are unique to broad cell type groups are shown. The inner circle denotes the cell type group from AD while the outer circle denotes the PD group. Darker shade represents terms enriched exclusively in neuron modules (excitatory and inhibitory neurons, CALB1, SOX6) and lighter shade represents terms enriched exclusively in glial modules (microglia, astrocyte, oligodendrocyte, OPC, endothelial cells). Biological processes related to altered cytoskeleton dynamics, impaired mitochondrial function, and apoptotic signaling are enriched across gene modules from neurons in both AD and PD. Immune response, synapse maintenance, and lipid transport-related terms are altered in one or more glial cell modules in both AD and PD.

For validation of these results, we turned to our external dataset pair. Analogous to our first analysis pair, we applied the GSEA pipeline to the gene modules derived from the Seattle-AD and Smajić-PD datasets. We confirmed that AD and PD neurons, oligodendrocytes, and OPC modules had the largest occurrence of shared terms. Moreover, this also corroborated the broader biological themes of protein synthesis, mitochondrial energy metabolism, myelination, and glucose metabolism as shared between AD and PD (Fig. S4).

In another overview analysis, we drilled down into the multitude of terms highlighted as being shared between AD and PD. We observed that the most frequently highlighted terms across all examined gene modules (cf. previous section; table S4) were shared between several cell types and were mainly cell injury response pathways. This finding aligns with existing knowledge that cellular responses to injury are often common across neurons, glia, vascular cells, and other systems of the central nervous system (CNS)[50–54].

To investigate cell type localized biological processes, we designed a probe to filter out potential cellular injury-related pathways. We first categorized the gene modules into two groups – neuronal modules and glial modules. Given the fundamental anatomical and functional differences between neurons and glia, these groups are expected to exhibit distinct responses to disease. The neuronal group included gene modules from excitatory neuron, inhibitory neuron, CALB1, and SOX6 cell types. The glial group comprised astrocyte, microglia, oligodendrocyte, and OPC. We then removed all GO BP terms that were enriched in both neuronal and glial modules. This resulted in a refined set of GO terms that were exclusive to either neurons or glia (Fig. 4E; cf. Fig. S5 for gene module level grouping). By comparing these terms between AD and PD, we identified neuron and glia specific shared mechanisms of overlap.

Neurons shared the greatest number of exclusive (unique to cell types belonging to this category) GO BP terms between AD and PD. We identified several terms associated with microtubule depolymerization and cytoskeleton dynamics shared between PD and AD neuron modules (PD Calb1 1, Ex 1, Ex 2 and AD In 1, In 2 and Ex 1. Shared genes associated with these terms included MAPT, FKBP4, GBA2, MAP1A, MAP1B, MAP1S, MAP2, MAPRE3, STMN1, STMN2, STMN3, STMN4). Another biological theme highlighted involved the alteration in mitochondrial release of cytochrome c regulation. Ex 1 and Ex 2 in PD and In 1 and In 2 in AD highlighted these terms (PINK1, PRELID1, CLU, BNIP3, DNM1L, GHITM, GPX1, MFF, MLLT11, MOAP1). Alterations in iron homeostasis were noted in Ex 1 from PD and In 1 and Ex 1 from AD (SOD1, several ATP genes, CCDC115, FTH1, FTL, ISCU, NDFIP1, SLC22A17).

We noted that glia-exclusive terms had several themes centered around the immune and complement systems along with synapse pruning, lipid transport, metal ion homeostasis, and thyroid hormone balance. T cell activation was enriched in PD Mic 2, End 2, and AD Mic 2. These modules shared genes such as B2M, HLA-A, HLA-B, HLA-C, HLA-DPA1, HLA-DRA, HLA-DRB1, HLA-DRB5, and HLA-E. Lipid and phospholipid transport showed up exclusively in microglial modules, in AD Mic 1 and PD Mic 2 (APOE, TSPO, and PRELID1). Response to thyroid hormone was recorded in PD Mic 2 and AD Mic 1 (CTSB, CTSH).

We also noted a few terms exclusive to opposite categories in AD and PD. For example, “response to copper ion” was identified exclusively in AD glia modules and PD neuron modules.

However, functionally related terms, like cellular response to copper ion, copper ion binding, and copper ion homeostasis, were enriched in AD Ast 1, Opc 1, Ex 1, and Ex 2 and in PD Mic 1, End 1, Ex 1, Ex 2, and In 1. A closer inspection of all terms belonging to cross-category modules, AD neuron-PD glia or AD glia-PD neuron suggested that these differences largely reflect the granularity and naming conventions of GO annotations. In contrast, for AD neuron-PD neuron and AD glia-PD glia specific terms were functionally distinct, reflecting meaningful biological differences rather than annotation-related effects. For instance, a manual search for “cytoskeleton” or “microtubule” highlighted only neuronal modules in both AD and PD. These neuron and glia specificity were further supported by replication in independent datasets (Seattle-AD and Smajić-PD).

Thus, our gene set enrichment analysis successfully identified a variety of matching biological, cellular, and molecular processes across AD and PD. Our observations highlighted key themes which localized to certain cell types in AD and PD. Neurons in AD and PD demonstrated alterations to cytoskeleton structural integrity, mitochondrial transport, and mitochondrial energy synthesis. Alternatively, glia-derived gene modules highlighted alterations to several regulatory mechanisms including synapse pruning, lipid transport, immune, and inflammatory systems.

Latent gene modules better reveal cross disease transcriptomic convergence than traditional differential gene expression

We compared our gene modules framework to the widely adopted univariate method in RNA-seq - DGE analysis. DGE identifies differences in gene expression between two groups (AD-control or PD-control in our case) by comparing expression profiles in diseased versus neurotypical cell states. We conducted a DGE analysis independently for the AD and PD datasets, focusing on each cell type separately (analogous to our main analysis). For each gene, we computed its log-fold change, contrasted between case and control, and identified a set of differentially expressed genes (DEGs). Statistically significant DEGs (FDR corrected p-value < 0.05; see Methods) were subject to further comparison between AD and PD cell types.

We estimated the overlap between the adDEGs and pdDEGs by computing Kendall’s tau-b (τ_b) coefficient (Fig. 5) between the log-fold change values of significant DEGs for 54 pairwise comparisions across 6 AD and 9 PD cell types. The highest significant correlation observed was 0.2 (p-value<0.05, n=1000 iterations label shuffle permutation test), occurring between oligodendrocyte adDEGs and OPC pdDEGs. Notably, this maximum correlation among all possible pairings was significantly lower than the maximum τ_b observed from our gene module analysis (cf. Fig. 2A).

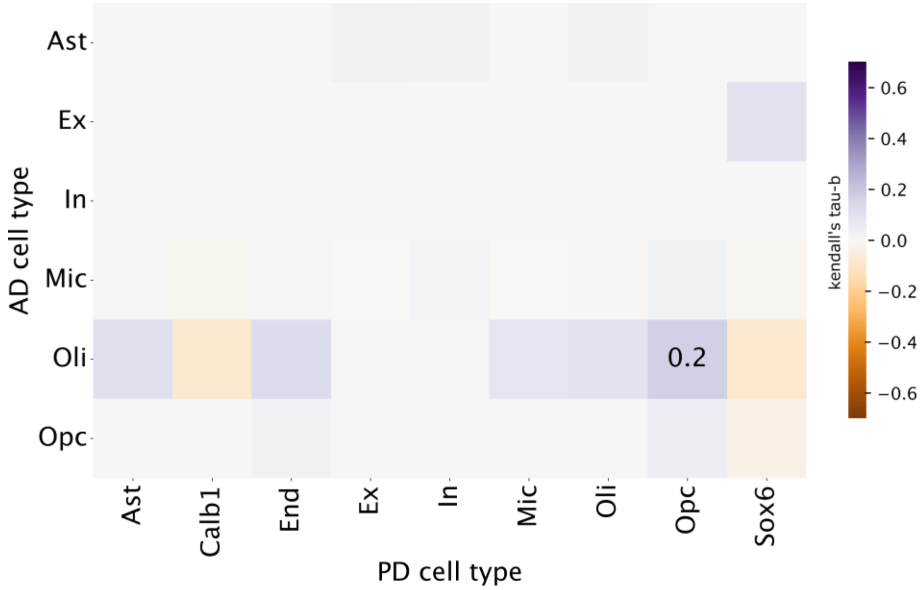

Figure 5. Differentially expressed genes compared between AD and PD to quantify overlap. To benchmark our latent factor modeling approach in identifying AD PD overlap, we conducted a differential gene expression analysis for AD and PD individually. The differentially expressed genes (DEGs) were identified using Wilcoxon rank-sum test contrasting transcription signatures between disease and control groups (adjusted $p < 0.05$ with Bonferroni correction for multiple testing), conditional on cell types. Here, we compared the DEGs between AD and PD and depict their overlap between all cell type pairs. Darker colors denote higher Kendall's tau-b of the log-fold changes in significant DEGs for each cell type, compared between AD and PD. Compared to our PLS gene-module-based overlap analysis (Fig. 2A), univariate DGE extract significantly smaller correlations. The maximum correlation observed is 0.2 for AD oligodendrocyte and PD oligodendrocyte precursor cells. Ast, Astrocyte; Ex, Excitatory neuron; In, Inhibitory neuron; Mic, Microglia; Oli, Oligodendrocyte; Opc, Oligodendrocyte precursor cell; End, Endothelial.

We then systematically quantified the overall difference in mean correlations between cross-disease associations based on pairwise gene module τ_b from PLS (12 x 20) versus pairwise cell type τ_b from DGE (6 x 9). We used Welch's t-test, suitable for samples of unequal sizes. Our results indicated a significant difference in mean correlation strengths. PLS_{cell} τ_bs were, on average, 3.88 points higher than DGE derived τ_bs (p-value<0.001). This finding suggested that associations between AD-PD similarity were systematically stronger when using our gene module approach compared to the classical DGE method.

Next, we performed a gene set enrichment analysis of the differentially expressed genes using GO databases (GO BP, MF, and CC). Independently for each disease, we created our ranked gene list based on the log-transformed fold change (conditioned on cell type) and used GSEA to identify significantly enriched terms (Benjamini-Hochberg FDR<0.05). We observed 20 common terms, in total, between AD and PD across all 3 GO databases (Fig. S6). These terms represented only a small subset of the broader set of shared AD-PD terms that were identified through our gene-module based analyses (213 GO terms). Notably, the terms derived from DGE appeared among the most frequently recurring terms across gene modules from the PLS analysis (cf. Fig.

4C and Fig. S6), suggesting that DGE may primarily capture the strongest disease overlaps from the gene expression matrices.

In summary, these findings demonstrated how a traditional univariate methodsm, DGE, can fail to capture the intricate mechanisms that might drive overlapping disease signatures in AD and PD brains. Our supervised latent factor modeling approach not only recapitulates the strongest AD-PD molecular overlap between oligodendrocytes, as uncovered by DGE, but also reveals a substantially broader set of shared, disease-relevant signatures. This highlights the added value of identifying cross-disease associations through a transcriptome-wide modeling of gene modules, rather than relying solely on individual gene-level differences.

GWAS-seeded co-expression networks also indicate AD-PD genetic overlap

In an alternative set of analyses, we pursued the same research question, the extent of AD-PD overlap, using a complementary quantitative analysis workflow. Devising a top-down framework (Fig. 6A) seeded with 164 genes mapped from GWAS risk loci (GWAS in AD or PD), we constructed disease-specific differential gene co-expression networks (DGCN) using the ROSMAP-AD and Kamath-PD datasets.

The notion of gene co-expression networks (GCN) rests on the assumption that genes with similar expression profiles, across cell transcriptomes, often share functional or regulatory relationships[55–57]. Our DGCN analysis integrated a contrastive element to GCN that compared disease and control groups. This allowed the elimination of the effects of housekeeping genes. Any residual patterns of covariation were then attributed to the effects of a disease. We computed DGCNs independently for the AD and PD datasets and separately for each cell type within a dataset. Concretely, for a given cell type, we computed Pearson's correlation (ρ) between the expression profile for each GWAS mapped gene (164) and the expression signatures of the remaining gene transcripts (16,936 transcripts), across all healthy cells. We then repeated the same analysis for diseased cells. The difference between the disease and healthy ρ gave us cell type-specific differential co-expression for each of the 164 genes based on GWAS risk loci. We did this analysis separately for ROSMAP-AD and Kamath-PD.

To quantify the alignment between our two analysis arms (PLS module arm and seed gene derived co-expression analysis arm), we measured the correspondence between the relevant genes from the DGCN analysis (top-down) and our PLS-derived gene modules (bottom-up). We looked at the number of robust genes that were common in each gene module - GWAS DGCN pair (Fig. S7A). Our findings suggested high PLS_{cell} module-DGCN alignments for the same cell types. For example, co-expression signatures from astrocytes shared, on average (across 164 seed genes), 20% of genes (significant ρ at FDR < 0.01) with PLS_{cell} Ast 2. Similarly, excitatory neurons shared, on average, 58.4% of genes (significant ρ at FDR < 0.01) with PLS_{cell} Ex 1 and 42.2% of genes (significant ρ at FDR < 0.01) with Ex 2. The substantial module-DGCN alignment supports the intrinsic validity of our two analysis approaches.

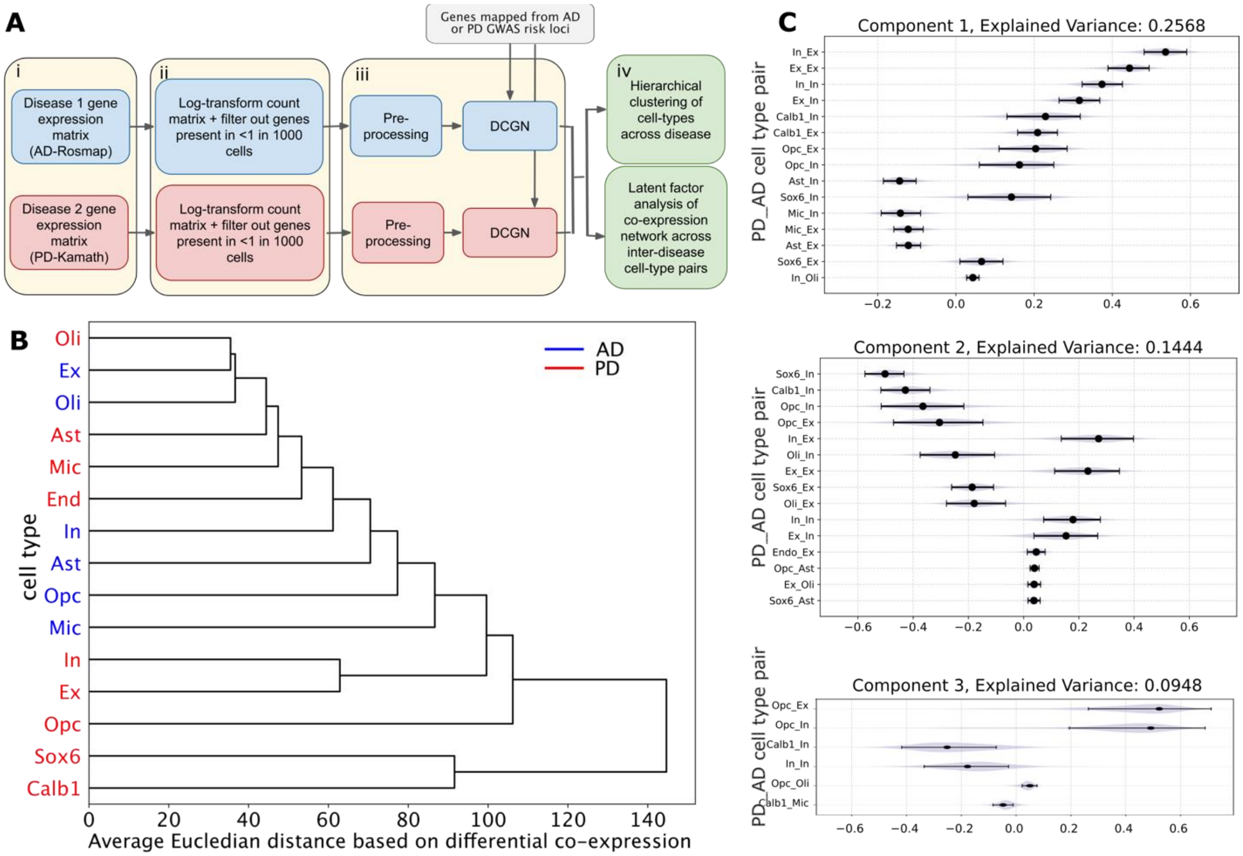

Figure 6. Differential gene co-expression network, a complementary analysis, identifies hints of overlap between AD and PD.

To corroborate our findings in a technical replicate, we contrasted differential gene co-expression networks (DGCN) between AD and PD. We constructed DGCNs to identify genes (out of 16,936) whose expression patterns were harmonious with seed genes (164 GWAS genes from AD or PD), but whose synchrony was altered in disease compared to control conditions. (A) Overview of workflow. (i) Single-nucleus RNA seq datasets were downloaded from open-source repositories. Preprocessing done by source authors included quality control, normalization and cell type annotation. (ii) Local processing was performed independently for each cell type. This step followed recommended guidelines for data transformation and removed lowly expressed genes. (iii) Gene co-expression network analysis. For each gene from a set of GWAS implicated genes from AD and PD, a co-expression network was created for the disease and control groups independently. The results were subtracted to give the differential gene co-expression networks (DGCN). 164 seed genes, implicated by the largest AD or PD studies, were used to construct the DGCNs. (iv) Post DGCN analysis. The disease-specific DGCNs were compared between AD and PD to identify cross-disease cell type-cell type associations. (B) displays the hierarchical clustering of cell types based on the similarity of the co-expression patterns captured by DGCN. The dendrogram is based on the average Euclidean distance of the DGCNs for all cell type pairs between AD and PD. (C) Kendall's tau-b (τ-DGCN) was used to calculate the pairwise associations between the DGCNs from two cell types. Cell type pair loadings for the top 3 principal components explain 50% of the total variance across all pairwise cross-disease DGCNs. Here, the pairs are presented in descending order based on the loading magnitude. The dots represent the empirical PCA loading. Error bars represent the 20/80% CI from 1000 iteration bootstrap analysis which sampled rows of a DGCN with replacement. Only pairs that did not include zero in their bootstrap interval are shown. The first and second principal

components are dominated by cell type pairings involving mainly excitatory and inhibitory neurons in AD and PD. The second component emphasize PD dopaminergic neurons. The third component focusses on glial and vascular cell types from PD across cell types from AD. Ast, Astrocyte; Ex, Excitatory neuron; In, Inhibitory neuron; Mic, Microglia; Oli, Oligodendrocyte; Opc, Oligodendrocyte precursor cell; End, Endothelial.

Finally, we proceeded to quantify the overlap between AD and PD at the co-expression network level. To this end, we performed a hierarchical clustering on the combined AD and PD DGCN matrix which comprised 15 cell types (6 AD and 9 PD) and 2,777,504 seed-gene-by-transcriptome feature pairs (see Methods). The clustering grouped cell types from AD and PD based on their gene expression co-variation patterns centered on GWAS genes (Fig. 6B). Importantly, we did not combine the gene expression matrices; instead, the DGCNs were derived separately for AD and PD. As an overarching observation, our secondary analytical approach recapitulated the main observation from our primary latent factor modeling arm. Specifically, excitatory neurons from AD grouped closest to oligodendrocytes from PD, indicating that their underlying disease-related deviations in gene co-expression profiles were similar to each other. AD-oligodendrocytes and PD-oligodendrocytes were also grouped closely, suggesting shared similar co-expression profile alterations in AD and PD. Overall, the cross-disease similarity clustering observed here corroborated several key findings in our PLS analysis arm.

We then assessed the extent of AD and PD overlap in yet another way. We used Kendall's tau-b to quantify the degree of similarity between the same seed-derived DGCNs from an AD-PD cell type pair (see Methods). Principal component analysis (PCA) was used to extract a 2D embedding from the 164 (seed genes) x 54 (AD-PD cell type pairs) correlation coefficients. The first 4 PCA components were determined to be statistically significant based on a permutation test (0.5/99.5% CI; Fig. S7B; see Methods). This examination quantitatively revealed systematic AD-PD cell pairings that shared similar co-expression profiles (Fig. 6C). The first principal component, accounting for 25.6% of the variance in seed-derived co-expression patterns, was dominated by robust AD-PD neuronal cell types (subjected to bootstrap robustness check; see Methods). The second component (14.4% of total variance) grouped DA neurons in PD with excitatory and inhibitory neuron combinations from AD. This was followed closely by combinations of microglia in AD and oligodendrocyte and OPCs in PD in the third component (9.5% of total variance). These results independently recapitulated our gene module-based findings, confirming the strongest AD-PD overlap in oligodendrocyte- and neuron-derived modules. Thus, our parallel analysis reinforces the robustness of our earlier results through a technical replication.

Discussion

In this study, we examined the molecular, genetic, and cellular ties between Alzheimer's and Parkinson's disease. By analyzing the entire protein-coding transcriptome at single-cell resolution, our multivariate approach uncovered AD- and PD-deviant genes forming co-expressed modules at sub-cell type granularity. We compared and quantified these gene modules demonstrating molecular overlap between AD and PD. Further, we mapped the gene modules to disease-relevant biological programs. This illuminated the complex genetic underpinnings leading to shared disease neuro-phenotypes. Thus, we provide single-cell genomics scientists with a tool to compare any pair of diseases from a global transcriptome perspective.

Our analytical protocol was enabled by access to an AD dataset (ROSMAP-AD) with 70,634 nuclei from 8 major cell types and a PD dataset (Kamath-PD) with 340,902 recorded nuclei from 11 major cell types. From both datasets, we extracted multiple gene modules for each cell type. These signified distinct modes of sub-cell type disease-deviated transcriptional changes. Our grading of the alignment of gene importance between pairwise gene modules from AD and PD demonstrated sizeable overlaps. In the spotlight were modules derived from AD or PD neurons, oligodendrocytes, and OPCs. This high degree of transcriptional differences mirrored between AD-PD was also highlighted in a secondary analysis arm, which compared gene co-expression networks between the two diseases. Additionally, we were able to replicate these findings in an external AD-PD snRNA-seq dataset pair. To our knowledge, these links are the first to reveal shared genetic control underlying measurable transcriptional differences in AD and PD.

To situate our findings within the context of GWAS, we examined whether established GWAS hits, from either AD or PD, were included in our gene modules. We found that these marker genes played robust roles within our functionally integrated gene modules despite being extracted in a transcriptome-wide approach. Additionally, we observed clear cell type localization of the popular genes within our modules. For example, APOE (the notorious AD risk gene) signatures were localized to microglia and astrocyte gene modules, agreeing with previous single-cell transcriptomics studies in AD[30,31]. Central to our investigation, several AD-relevant marker genes surfaced in PD-associated gene modules and vice versa, highlighting the interconnectedness between the two disease categories. For example, APOE had robust PD associations in several of our PD gene modules, including those pulled from microglia, astrocytes, oligodendrocytes, and neurons. Indeed, APOE has previously been shown to be predictive of cognitive decline in PD patients based on clinical studies[58,59]. Additionally, APOE has been shown to exacerbate PD pathology in mouse models[60]. As another example, we consider SNCA, a major gene implicated in PD GWA studies[61]. In the PD brain, misfolded SNCA protein, α-synuclein, is found in Lewy bodies and is a primary neuropathological marker[62]. Here, along with several neuron and glial PD modules, SNCA emerges as a strong contributor to AD excitatory neuron modules. Indeed, APP transgenic mice with SNCA knockout have demonstrated a significant reduction in amyloid burden, hinting at connections between this PD gene and AD pathology[63,64]. Taken together, we have successfully replicated the roles of important AD or PD genes while placing them within the fuller context of gene modules. We have further expanded the implication of these genes to cross-disease correspondences, to which previous univariate approaches were systematically blindfolded[12].

In addition to comparison with GWAS, we provided biological insights to our AD and PD comparison by grounding our gene modules in pathways from pre-curated gene ontologies. We identified shared alterations in cellular energy metabolism and stress response, inflammation, lipid signaling, protein folding, and protein degradation cascades. Some of these overarching disruptions in biological processes have been discussed in reviews summarizing the collective understanding from decades of neurodegeneration research[50]. In a clean bottom-up workflow, our study confirmed these prior findings while going beyond previous studies by carefully localizing these molecular processes to cell-type-specific gene modules. For example, one potentially shared feature of neurodegeneration, as evidenced in previous brain tissue staining, was the presence of abnormal protein aggregates[50,65]. These can vary in the type of protein (tau, amyloid-β, α-synuclein), the form of the aggregate (beta sheets, fibrils, oligomers), or the cell type they plague

(neurons, astrocytes, oligodendrocytes). In line with this, our enrichment analysis revealed that protein misfolding (ER stress) and its associated biological processes were widespread across all major cell types, diseases, and datasets. We also detected extensive alterations in molecular pathways related to protein degradation (ubiquitin protein ligase binding and clathrin-mediated endocytosis[66]), indicating a potential breakdown in protein disposal systems. Indeed, cellular assays had identified malfunctions in ubiquitin-dependent protein clearance in neurodegeneration[67,68].

Shared molecular mechanisms between AD and PD in neurons

Neurons are particularly sensitive to proteasomal turnover due to their longevity and delicate synaptic regulatory requirements[66]. The toxic effects of accumulated proteins ultimately lead to neuron death and usually mark the final stages of neurodegeneration. Zooming in on neurons, we identified specific mechanisms shared between AD and PD that were exclusive to neuron gene modules. These were primarily related to alterations in cytoskeleton dynamics, impaired mitochondrial functions, and apoptosis mechanisms.

Defects in cytoskeleton dynamics as major contributors to neuronal death have been cited by several lines of evidence, including microscopy and genetic studies[69]. In our gene module enrichment, we found microtubule-associated processes to almost uniquely localize to neuronal modules in both AD and PD (in all 4 examined datasets). Alterations to post-translational modifications of neuronal microtubule's acetylation levels have been reported before by in-vitro and in-vivo studies in both AD and PD[70,71]. The involvement of the MAPT gene in these gene modules was noteworthy. This gene encodes for the protein tau which is responsible for stabilizing the axon microtubules. In cell culture studies, tau aggregates demonstrate prion-like behavior (prion-like characteristics of misfolded proteins imply the ability of self-propagation through seeding), passing from neuron to neuron across synapses[72–74]. Overall, the convergence of our findings with previous research hints at dedicated mechanisms that are possibly aligned between AD and PD and which can ultimately lead to prion-like manifestations[75,76].

Of note, the same gene modules that encoded dysfunctional cytoskeleton dynamics were also enriched in several terms related to the mitochondria, including axonal transport of mitochondria and protein localization to mitochondria (all 4 datasets). This observation alludes to a vicious cycle between dysregulated microtubules and impaired mitochondrial transport accompanied by oxidative stress. Intact microtubules in neurons serve as highways for cargo transport (proteins, mRNA, organelles like mitochondria) through axons and dendrites[77]. Previous research have shown that alterations to its stability leads to the breakdown of mitochondrial transport, which in turn leads to mitochondrial reactive oxidative species (ROS)[52,78]. ROS, in turn, exacerbates the levels of free tubulin[79] which in turn has been shown to interact with proteins like α-synuclein, promoting the formation of oligomeric aggregates in the form of Lewy bodies[80], or tau, promoting intraneuronal neurofibrillary tangles (NFTs)[78]. Notably, in PD pathogenesis, α-synuclein oligomers are also highly effective at seeding further protein misfolding in a prion-like fashion[81].

Cells maintain a state far from thermal equilibrium by continuously extracting energy from their environment. Neurons, in particular, have especially high energy demands, and mitochondria

are a major source of energy in these cells. We have observed alterations in the regulation of cytochrome c release and the apoptotic signaling pathway in several AD and PD neuronal gene modules. In the intact human cell, cytochrome c is present in the mitochondrial intermembranous space. The presence of oligomeric forms of amyloid-β, α-synuclein, and tau has been shown to increase mitochondrial membrane permeability. It can cause leakage of cytochrome c into the cell cytosol[82]. The loss of cytochrome c from mitochondria compromises the downstream functionality of cytochrome c oxidase which is used for ATP energy production. Instead, once in the cytosol, cytochrome c is known to initiate mitochondria-mediated apoptosis[83]. This form of neuronal death has long been suspected as an early event in the pathophysiological cascade leading up to both AD[84] and PD[85,86]. Specific to PD, in a self-reinforcing manner, impaired cytochrome c release from mitochondria is thought to escalate α-synuclein oligomerization via radical formation[81]. Together, impaired mitochondrial functions along with a disrupted axonal transport system may individually or collectively be a universal mechanism of neurodegeneration in AD and PD.

Oligodendrocyte and Oligodendrocyte precursor cell modules in AD and PD

In oligodendrocytes and OPCs, we located additional gene modules with high AD-PD overlaps. Three key observations stand out related to these modules. First, we observed that a sizeable portion of GWAS genes localized to cross-disease oligodendrocyte modules. Notably, PD oligodendrocyte modules contained more GWAS genes than modules from other cell types. Previous transcriptomic studies have pointed out that several disease risk loci from GWAS are associated with oligodendrocytes in both AD[87] and PD[88]. Our study confirms and expands on this observation. Second, in our enrichment analysis, several biological processes revolving around myelination and regulation of axonogenesis were specific to these cell type modules and were shared between AD and PD. Correlative macroscopic brain-imaging studies have linked deteriorating myelin health to AD progression[89]. A recent invasive experiment posited a causal link between aging myelin and AD[90]. In a few AD mouse models and a human PD model, RNA-seq analysis identified changes in oligodendrocyte transcription related to impaired myelination[35,91,92]. Finally, here, in addition to strong intra-cell-type module associations, AD oligodendrocyte modules also exhibited a high degree of association with excitatory neuron modules from PD. Such similarity between oligodendrocyte and excitatory neuron transcriptional modifications in AD was previously reported in the ROSMAP-AD transcriptomic study, in terms of shared DEGs[30]. The extension of this overlap to cross-cell-type modules from AD and PD suggests pervasive crosstalk between excitatory neurons and oligodendrocytes in neurodegeneration.

Shared role of heavy metals is highlighted between AD and PD

In general, dysregulated homeostasis of heavy metals leads to an increased risk of the onset and progression of neurodegenerative diseases, as evidenced by studies in both humans and animals[93,94]. Astrocytes, in particular, have been shown to remove excess heavy metals from the brain parenchyma[95]. In our study, gene modules from astrocytes showed exclusive enrichment for response to zinc ions in both AD and PD. Several metallothionein (MT) genes were common between these modules (MT1E, MT1G, MT2A). Previous in vitro models have shown that zinc induces harmful A1-type reactive astrogliosis which promotes synaptic degeneration in neurons[96].

We found consistent enrichment of copper (Cu) ion regulation and interaction in both neuronal and glial modules across all examined AD and PD datasets. These modules featured several MT genes (MT1E, MT2A, MT3), and APP. In the brain, unregulated Cu readily derails redox chemistry (Fenton/Haber-Weiss reactions), thus forming more cytotoxic ROS[97–99]. Biophysical and biochemical experiments underscore our metal pathway findings, showing that altered Cu ion levels triggered misfolding of α-synuclein[100] and amyloid-β[101]. Interestingly, in our study, a distinct set of genes related to copper ion binding appeared exclusively in the excitatory neuron modules from AD and PD. These genes were linked to critical antioxidative enzymes (SOD1, PARK), SNCA, and the copper chaperone protein (ATOX1). Prior in-silico analyses of microarray data on brain tissue had reported a similar grouping of copper-handling genes (metallothionein group and the enzyme binding group)[102]. Using our analytical framework, we were able to localize these effects to specific gene sets within distinct brain cell populations.

Another metal ion, iron ion, homeostasis terms were found to be enriched in neuron gene modules from both AD and PD. Across all datasets, excitatory and inhibitory neuron gene modules were involved. In the brain, iron plays a key role in myelin synthesis, neurotransmitter production, and overall metabolism[103]. However, elevated levels of redox-active iron, often originating from degenerating mitochondria, accumulate in several neurodegenerative diseases as evidenced by biochemical, cell biological, and transgenic animal studies[104–107]. Thus, understanding disruptions of these heavy metal pathways that extend across AD and PD is an interesting avenue for further investigation.

Common mechanisms of microglia's involvement in AD and PD

In contrast to neurons, overlapping microglia-implicated terms in AD and PD were highly diverse in their functional themes. This underscores the broad roles that these cells play in neurodegeneration, transcending brain regions. We posited that the difference in developmental origins of microglia compared to other CNS cell types, such as neurons and macroglia (astrocytes and oligodendrocytes), may explain a part of this phenomenon. Unlike neurons and macroglia, which derive from the neuroectoderm[108], microglia originate from peripheral mesodermal tissue[109,110] and exhibit considerable homogeneity across brain regions. Most microglial genes exhibit similar expression patterns extending across cortical regions (origin of our AD datasets) and striatal regions (origin of our PD datasets)[111–113]. This uniformity in gene expression might explain why several biological functions altered in the degenerative state were shared between AD and PD in microglia modules.

Functionally, microglia are the primary immune cells of the CNS[114], and neuroinflammation and immune system dysfunction are believed to be key components of neurodegeneration[115]. Consistently, microglial gene modules from our study were enriched for several immune-related processes. For example, we noted T cell activation in both AD and PD (all 4 datasets). Microglia, upon activation by neuronal stress, are thought to release pro-inflammatory cytokines and upregulate MHC class I and II molecules[116]. Further, the inflammatory cytokines can induce the expression of adhesion molecules on brain endothelial cells, compromising the integrity of the blood-brain barrier (BBB). This BBB breakdown accelerates peripheral immune cell entry. Thus, in a chicken-egg scenario, microglia and endothelial cells drive a chain reaction of T cell activation, oxidative stress, and neuroinflammation[117–119]. This domino effect may have

been captured in a PD endothelial module which featured T cell activation simultaneously with biological processes like leukocyte adhesion to vascular cells, blood vessel morphogenesis, and diameter maintenance, pointing to the dysregulation of the BBB. This cascade of immune response events exacerbates ROS production and neuronal damage[117,118].

Another contributing factor to increased ROS is imbalances in thyroid hormones (TH) [120]. We found a robust overlap for response to TH in microglial gene modules from both AD and PD. Epidemiological evidence have linked multiple thyroid-related autoimmune diseases to increased prevalence of both AD[121,122] and PD[123]. However, the exact contribution of TH to either AD or PD pathophysiology has not been fully established. Recently, an AD mouse model has linked brain hypothyroidism with reduced microglial reactions to inflammatory stimuli and aberrant amyloid-β[124]. Following this, an α-synuclein PD mouse model found connections between TH and glucocerebrosidase activity in microglia[125]. Our findings that highlight TH involvement in AD and PD demonstrated the efficacy of our data-driven model in opening important avenues for investigation.

Our approach also mapped microglial gene modules from AD and PD to synapse pruning. These modules implicated key genes, including TREM2 and several C1q genes from the complement system, which is an integral component for synaptic refinement. Synapse loss is frequently observed as an early event in animal models of neurodegeneration[126–128]. Variants in the TREM2 protein and aberrant activation of C1q genes have been shown to cause irregular synapse pruning in studies on AD mouse models[128–130]. In a PD mouse model, suppressing TREM2 gene products in microglial cells was shown to accelerate the loss of DA neurons[131,132]. Yet, the totality of glia-synapse interactions, especially the role of the complement system in PD, is under-investigated[133,134]. Once again, our bottom-up approach identified a common disease outcome in AD and PD in the form of synapse loss – a potential area for further investigation.

In addition, lipid transport regulation was enriched in both AD and PD microglial gene modules (lipid, phospholipid, cholesterol, and sterol transfer terms) implicating key genes including APOE, TSPO, TMEM30A, several ATP-binding cassette subfamilies (ABC) genes (ABCG1, ABCA5) and NPC2. Excess lipids from dysfunctional and stressed neurons are transported to glial cells via apolipoproteins E and D[135]. This excess lipid has been reported to accumulate as droplets in human iPSC-derived microglia, reducing their phagocytic capabilities and increasing the secretion of pro-inflammatory cytokines[136,137]. To sum up, the above impairments mediated by microglia ultimately exacerbates ROS burden and neurotoxic buildups leading to neurodegeneration.

In conclusion, our proof-of-principle investigation of the transcriptomic terrain intersecting AD and PD identified and characterized key shared genetic components of neurodegeneration. We were able to (i) quantify the degree of overlap between unique gene modules from AD and PD, (ii) chart the extracted molecular changes in brain tissue to distinct cell types that matched in AD and PD, as well as (iii) interpret our in-silico derived gene module functional associations with several lines of existing experimental evidence. Future work can expand our study to include a greater diversity of neurodegenerative, neurodevelopmental, and psychiatric diseases. Moreover, applying a similar framework to different, readily accessible source transcriptomes, like blood or cerebrospinal fluid, can identify critical biomarkers for neurodegeneration.

Materials and Methods

Single genomics data resources

The advent of single-cell RNA sequencing has revolutionized cell biology by enabling the definition of cellular identity and heterogeneity through transcriptome data in both healthy and diseased tissues[138–140]. As a variant of this, single-nucleus RNA sequencing (snRNA-seq) is particularly well-suited for tissues like the brain, where the availability of fresh tissue samples is limited[141,142]. Our present study benefited from recently emerged and expansive snRNA-seq data, pertinent to AD or PD, from four separate and independent studies.

Primary datasets

ROSMAP Alzheimer's dataset[30]: We examined an exceptionally valuable dataset of gene expression – the first investigation of AD using snRNA-seq. This dataset was derived from post-mortem brain tissue sourced from the prefrontal cortex (BA10) of individuals participating in the Religious Orders Study or the Rush Memory and Aging Project (ROSMAP)[41]. All participants enroll without known dementia and agree to annual clinical evaluation and brain donation. Both studies were reviewed by an Institutional Review Board of Rush University Medical Center, and all participants signed informed and repository consents and an Anatomic Gift Act. The dataset was collected from 48 subjects who were carefully matched in terms of age and sex. These subjects consisted of 24 males and 24 females, with an equal amount of 24 individuals diagnosed with AD and 24 control subjects. The mean age of the individuals was 85 years. The recorded cell types included inhibitory neurons, excitatory neurons, oligodendrocytes, oligodendrocyte precursor cells, astrocytes, microglia, endothelial, and pericytes. The dataset comprised a total of 70,634 cell transcriptomes from 8 cell types and encompassed transcript counts for 17,926 protein-coding genes, aligned with the human reference transcriptome (hg38 GRCh38.p5).

Parkinson's disease, Kamath dataset[37], GEO accession number **GSE178265**: This dataset included snRNA transcriptomes from post-mortem human midbrain and dorsal striatum (caudate nucleus and substantia nigra) tissue. Tissue samples were derived from an age and sex-matched cohort of 15 subjects, consisting of 7 males and 8 females. Among this group, 6 individuals had been diagnosed with PD, while the remaining 9 were controls. The mean age of the individuals was 83 years. Major cell types in this dataset included inhibitory and excitatory neurons, oligodendrocytes, oligodendrocyte precursor cells, astrocytes, microglia, and endothelial cells. The dataset also recorded a specialized subset of dopaminergic neurons from the midbrain, CALB1, and SOX6. The dataset provided transcript counts for 33,692 genes (genes were aligned to hg19) from 11 cell types and encompassed 340,902 single nuclei transcriptomes.

External validation datasets

Seattle Alzheimer's dataset[31]: The authors of this resource sourced brain specimens from the Adult Changes in Thought Study and the University of Washington's Alzheimer's Disease Research Center. It is worth noting that the cohort detailed in this research represents the entire spectrum of Alzheimer's disease severity. Brain tissue samples were drawn from the middle temporal gyrus. The study included participants from age groups ranging from less than 65 years

to more than 90 years. To maintain consistency with our other datasets, we considered only participants over the 65 to 77-year age bracket. This gave us 76 individuals, with 47 females and 29 males. Out of these, 36 subjects were cases of recorded dementia, and 40 were controls. The mean age of the individuals was 88 years. The preprocessed datasets for the cell types labeled 'L4 IT', 'L5 IT', 'Vip', 'Pvalb', 'Sst', 'Sncg', 'Oligodendrocyte', 'Microglia', 'Astrocyte' and 'OPC' were downloaded from the cellxgene platform. The final dataset featured 683,260 nuclei across 10 cell types and recorded 36,517 genes.

Parkinson's disease, Smajić dataset[35], GEO accession number **GSE157783**: The creators of this resource worked with post-mortem midbrain tissue sections and linked with the associated clinical and neuropathological data from the Parkinson's UK Brain Bank and the Newcastle Brain Tissue Resource. The dataset included age and sex-matched nuclei samples from adult human post-mortem midbrain tissue from 5 cases of idiopathic Parkinson's disease, all of whom exhibited severe neuronal loss in the substantia nigra and had no family history of the disease. 6 control midbrain tissue samples were also collected to match the characteristics of the idiopathic Parkinson's disease patients. The mean age of the subjects was 80 years. In total, 24,005 genes (mapped to hg38) were recorded in 41,435 single nuclei from 10 cell types.

Preprocessing pipeline at source

We relied on the preprocessed datasets from the authors responsible for the data collection (cf. above). This maximizes reproducibility and compatibility with other studies working with these resources. The transcriptomic datasets were processed in the source studies using standard snRNA-seq processing pipelines. This included quality control for cell inclusion, including doublet detection, the removal of low-quality and outlier cells, the removal of lowly expressed genes, and cell clustering into cell types (exact details can be found in the Methods sections of individual research). Importantly, the cell type classification - which cell belongs to which cell population - was taken from the original studies as a basis for our investigations.

Primary local preprocessing

To maintain comparability across the different datasets that recorded a variable number of genes, in our study we looked at the effects of genes that were recorded in all 4 AD and PD datasets. This gave us a total of 16,936 protein-coding genes that we used in further analyses, enabling us to make an apples-to-apples comparison. By considering only protein-coding transcripts, we also reduced our feature space size which is beneficial for any high dimensional analysis[143,144].

Identifying gene modules: supervised latent factor modeling

At the heart of this study, we wanted to explore synchronous gene expression changes that occur in the brain accompanying a disease state and compare them between AD and PD. Supervised latent factor models are a natural choice to uncover the hidden patterns in a high-dimensional feature space, simultaneously accounting for the relationships between the observed variables and their associated disease-vs-control labels. Here, gene transcription signatures were the input variables that are to be embedded in a space maximizing disease versus control class separation. Gene expression measurements are known to have high correlations among

themselves[25], giving rise to an additional modeling consideration (multicollinearity). Partial Least Squares Regression (PLS-R)[145] is a multivariate statistical technique that is particularly well suited in situations with multicollinearity (high correlation among features) or when dealing with high-dimensional data with considerable noise and small sample size.

In the present analysis, we utilized a variant of PLS-R for classification with one dependent categorical variable. This is known as PLS discriminant analysis (PLS-DA)[146]. In doing so, we developed a predictive modeling framework to categorize disease versus control diagnosis based on input gene expressions. Formally, our input datasets are denoted by, $X \in R^{N_c \times M}$ and $y \in R^{N_c \times 1}$, where N_c was the number of observations (nuclei) for a cell type c, M the number of measured features (genes; $M = 16{,}936$), $X_{i,j}$ holds the recorded transcript count for the ith nuclei and j^{th} gene, and y_i was the disease label (+1 for disease and -1 for control). The goal was to construct a low-rank projection from the 16,936-dimensional gene space that maximized the within-class separation between disease and control.

Concretely, PLS-DA can be viewed to consist of two key equations:

$$X = TP^T + E,$$

$$Y = UQ^T + F,$$

where T and U are $N_c \times k_c$ score matrices of k_c extracted components, P is a $M \times k_c$ (gene-wise) loading matrix (effect size) of X and Q is a $1 \times k_c$ loading vector of Y respectively. E and F are the residual matrices of X and Y respectively. The decomposition of X and Y is set to the solution of the optimization objective:

$$cov(t,u)^2 = cov(Xw, Yh)^2$$

where $cov(t,u)$ is the captured covariance $\frac{t^T u}{N_c}$, w and h are weight vectors that are extracted using the NIPALS algorithm[147]. Interpreting loading vectors in PLS classification is essential to understanding gene expression features' impact on disease detection. High absolute loadings signal strong contributions (positive to the target disease, negative to the control group), while near-zero loadings indicate minimal impact[39].

PLS-DA is prone to data overfitting when the number of features far exceeds the number of observations, especially in the case of inherently noisy, highly collinear single-nucleus data[148]. To address this issue, we first applied principal component analysis (PCA) for dimensionality reduction to the gene expression input matrices. Applying dimensionality reduction techniques has been merited as a pre-processing for PLS-DA[149]. The optimal number of PCA components was kept to $min(500, N_c)$, where N_c was the number of recorded nuclei for the given cell type c in the dataset. We used this transformed gene space for the PLS-DA analysis described above. To transition from the PCA embedding space back to the gene space, we projected the PLS loadings (in the lower dimensional space) back into the original gene expression space. This ensured that the domain interpretability of the PLS estimates was preserved in the biological ambient space.

Model selection, training, and performance assessment

Different cell types perform widely diverse functions, each arising from the functional recruitment of distinct gene families. In our pursuit to discover biologically meaningful gene groups, we implemented our quantitative analysis pipeline for a given disease on a cell type-by-cell type basis. This agenda enabled us to extract coherent latent components (latent factor/loading vector, hereby referred to as gene module) specific to each cell type. To ensure an even sample size in the disease and control group, we randomly sub-sampled transcriptomes of a given cell type to have comparable counts of nuclei from both disease and control instances. On this sub-sampled data, we removed genes that were captured in fewer than 1 out of 1000 cells to reduce our model's degrees of freedom (*scanpy.pp.filter_genes*), which is in line with previous research[150,151]. To reduce technical variation from sequencing depth, we normalized the data by dividing the raw UMI count by the total number of detected UMIs in each cell (*scanpy.pp.normalize_total(*data, target_sum=1e4)). Next, to account for the heteroskedasticity originating from differences in highly expressed vs lowly expressed genes, we log-transformed the normalized data from the previous step (*scanpy.pp.log1p*(data)). These dataset normalization approaches have been shown to work well as a preparatory step for downstream dimensionality reduction[152]. In a data cleaning step, inter-individual variation in gene expression that could be explained by differences in post-mortem interval was regressed out[153,154]. The thus derived balanced, cleaned, and standardized transcriptomic profiles for each examined cell type were used for subsequent steps in our pattern-learning analysis pipeline.

After preprocessing and cleaning the transcriptomic data resources, we carried out the estimation or training of our supervised learning model. The PLS classifier needs tuning of one hyperparameter - the number of hidden components of variation in the data. Selecting this optimal number of latent components is crucial, choosing too few components implies losing out on crucial information and too many invariably leads to overfitting. We adopted a rigorous 10-fold cross-validation (CV) scheme to inform this model selection problem, carried out separately in each cell type. The set of transcriptomes was randomly split into 10 equal-sized data point subsets. We ensured that the disease-to-control ratio of cells for each subset reflected that of the full dataset. Screening a range of component choices (1 to 8), in each iteration, 9 out of these 10 data subsets were combined and used for training a PLS model, while the held-out subset was used for evaluating the choice of component number. The model's performance was evaluated based on the area under the receiver operating characteristic curve (AUROC) in disease discrimination. This was performed for all combinations of training and validation subsets (*scikit-learn model_selection.GridSearchCV* function with *PLSRegression* as the 'estimator', 'scoring' set to 'roc_auc' and 'n_components' parameter set to 1-8). The number of components yielding the maximum mean AUROC over the CV subsets was noted as optimal for the given cell type. Note that this approach allowed us to independently derive the number of components that maximized disease-vs-control identification for each cell type, without overfitting to the input transcript space. This gave us 12 gene modules in AD across 6 cell types (2 cell types in ROSMAP-AD, pericytes and ependymal cells, did not pass our significance test. They were removed from further analysis) and 20 gene modules from 9 PD cell types (2 cell types in Kamath-PD, macrophages and ependymal cells, did not pass our significance test. They were removed from further analysis).

Next, we fitted PLS models on the full set of transcriptome observations for each cell type (PLS *model* specified using *sklearn.cross_decomposition* module *PLSRegression*). In doing so, we extracted multiple, unique disease-relevant gene modules. The statistical significance of an overall gene module was assessed in a principled, non-parametric permutation procedure. In 1000 permutation iterations, the transcriptome signatures were held constant, while the disease labels (outcome of model) were shuffled randomly across transcriptomes. The resulting surrogate datasets preserved the statistical structure of gene expression profiles while selectively destroying the association of the transcription profiles (model input) with diagnosis (model output). This approach generated a null distribution with minimal modeling assumptions[155,156]. The empirical covariance (test statistic) between the gene expression and disease signature captured by each module ($cov(t, u)$ defined above, t being $model$.x_scores_ and u being $model$.y_scores_) was compared with the resulting permutation distribution. This distribution reflected the null hypothesis of random association between gene transcription and the disease designation, which we test the actual model instance against. We deemed significant a module's input-output covariance in the latent space if fewer than 5% of the null models yielded a better covariance strength than the original covariance from the actual model instance (Fig. S1B). In case a module failed to pass this label-shuffling permutation test, it was dropped from further analysis, along with all underlying gene modules for that cell type. Thus, in a data-driven approach, we were able to determine which gene modules in a cell type at hand carried enough information that allowed us to discern a biological signal from noise. Consequently, modules from cell types with few recorded nuclei were discarded, as their corresponding PLS models did not robustly pass the described rigorous permutation scheme.

To identify the subset of the examined genes that robustly contributed to disease detection in each gene module from a cell type, we implemented a 500-iteration bootstrap (BS) scheme. The bootstrap resampling was done by selecting nuclei, with replacement, from the cell type observations before applying dimensionality reduction. This approach simulated random nuclei sample draws that could have been derived from the broader cell population. Dimensionality reduction (PCA) and PLS model estimation were performed on the resampled bootstrap dataset in an identical fashion (cf. above). We disregarded any genes whose model coefficient effect size (PLS loading corresponding to this gene) included zero in its 5/95% BS-confidence interval (CI) for that module (i.e., its effect was removed by setting the loading value to zero). The derived 'robust' gene modules will henceforth be denoted as P^* (dimension $p \times k_c$; cf. PLS definition section above) in future references.

An inherent ambiguity of the class of latent factor models (i.e., aspects of model non-identifiability), including PCA and PLS, is the reflection invariance of derived latent vectors. To remedy this source of indeterminacy, we computed the cosine similarity (γ; range -1 to +1), between a BS loading vector and its corresponding empirical loading vector. In the scenario where γ was less than 0, indicating a flipped ("mirrored") loading vector, we multiplied the loading vector of the BS model elementwise by -1 to align it with the original loading vector. This method has been employed by previous authors to address the issue of reflection[39]. The resulting distribution of loadings for a gene in a module was compared to its counterpart in the original model estimate.

Motivated by our overarching goal to quantify the association between gene modules from two diseases, we first sought to explore the degree of similarity and difference between pairs of

gene modules obtained from the same disease. Each gene module from a cell type captures a unique and complementary view of the relative roles of genes for successful disease prediction, hence we expected to see a low degree of association. In contrast, we did not rule out the existence of cross-cell-type gene module similarities. Kendall's tau-b correlation metric (τ_b; ±1 being a high degree of association and 0 being no association) was chosen to gauge this overlap signal (cf. rationale of τ_b metric discussed later). We calculated τ_b for all combinations of module-module pairs (${}^{k_c}_{2}C$ combinations, for k_c modules), resulting in a k_c^2 correlation matrix. A low magnitude τ_b indicated complementary modules whereas a magnitude of τ_b close to 1 indicated a higher degree of similarity in the compared gene modules.

To audit the performance of the individual PLS models calibrated for each cell type in classifying disease versus control, we employed AUROC of disease classification as our evaluation metric. Given that cell samples from a patient can exhibit significant autocorrelation, it is crucial to account for this when evaluating the model. Traditional test-train splitting methods often involve blindly partitioning the dataset. This can result in overly optimistic test performance and makes it challenging to detect overfitting during the testing phase[157]. In light of this, we employed a variation of cross-validation combined with bootstrapped Latin partitions[158] to ensure patient-level stratification. Concretely, in each iteration, a random sample of subjects (not cells) was drawn with replacement. This formed the basis of the training dataset's nuclei source. Based on the subset of patients, a random sample of cells was drawn, with replacement, while ensuring that the disease-to-control ratio of nuclei was reflective of the empirical dataset. The percentage of cell samples from any given subject was also preserved in each iteration. This analytical protocol ensured that transcription signatures from the same patient were not present in the training and testing set at the same time. Individual PLS models were fitted on the re-sampled train dataset. This fitted model was then evaluated based on AUROC scores on the out-of-bag nuclei (test set), i.e., the transcripts from subjects that were not included in the training step. We performed 1000 iterations of this bootstrap-based model disease classification performance evaluation. This allowed for a principled assessment of the disease discrimination strength of the PLS solutions based on the cell type-specific gene transcription signatures.

PHATE visualization

To gain a synopsis of the uniqueness of different gene modules from a single cell type, we created a concise, low-dimensional representation of high-dimensional cellular transcriptomes. For this purpose, we applied an especially flexible dimensionality reduction using PHATE[42]. Compared to prevalent visualization techniques like tSNE or UMAP, PHATE is well suited for noisy snRNA-seq data. It has been shown to preserve both local and global structures in a dataset and can capture non-linear relationships in the transcriptomic information, by mapping them into diffusion maps relative to each other. We estimated a separate PHATE model for each cell type. We projected the transcriptomes from each cell type into independent low-rank spaces (using the *scanpy external.tl.phate* function with default parameters except n_pca = 500). Instead of using specific disease marker genes as done traditionally to identify subtypes for cells, we colored the cells in the PHATE embedding space based on their PLS score for each latent component (*PLSRegression x_scores_*).

Quantifying disease overlaps at the gene-module level

To quantify the similarity between AD and PD at the level of gene expression patterns, we computed the association between functional gene programs of AD and PD. Importantly, a similarity metric (correlation) was computed across the derived model's predictive rules for a disease. That is, we did not pit the raw gene expression measurements against each other.

Formally, for a disease d and cell type c, the ith robust gene module (cf. above) can be denoted as $p^{*d}_{c,i}$, where p^* is a vector of dimension $1 \times M$ (number of genes in the input feature space). A robust gene module can contain tens to thousands of genes with non-zero loadings (out of ~17,000 genes) while the remaining loadings are zero (cf. above). To minimize the impact of tied zero loadings in the correlation metric calculation between two modules, we considered only those genes that had non-zero weights in both modules (AND conjunction). This approach works well to identify groups of genes with similar disease contributions between two modules while ignoring genes that might have robust effect sizes in one disease but not the other.

Concretely, we employed Kendall's tau-b ranked correlation metric (τ_b) to evaluate pairwise correlations between AD and PD gene modules. Kendall's tau-b correlation effectively handles tied ranks and provides a more accurate measure of ordinal association between gene modules. This is unlike Pearson's-r, which assumes monotonicity and is unstable, or Spearman's-r, which is biased and difficult to interpret [159]. For all pairwise gene modules from AD (12 modules across all cell types) and PD (20 modules across all cell types), Kendall's tau-b rank correlation coefficient, $\tau_b\big((c1,i),(c2,j)\big)$, was calculated using the scipy function *stats.kendalltau*($p^{*AD}_{(c1,i)}$, $p^{*PD}_{(c2,j)}$), where $(c1,i)$ is the ith gene module for the cell type $c1$ present in the AD dataset, $(c2,j)$ is the jth gene module from the cell type $c2$ present in the PD dataset. To independently assess the statistical significance of each coupled module association, we employed a non-parametric permutation procedure with the null hypothesis of random association between gene modules from different diseases. For each gene module pair, we re-utilized the label shuffling derived module weights (cf. above) to calculate a null distribution from 1000 permutation iterations. We only interpreted a module-pair's correlation coefficient that emerged as statistically relevant against a 5/95% CI threshold.

Yet another test was conducted to estimate the statistical sensitivity of the coupled associations of cross-disease gene modules. Across 500 iterations, we randomly bisected the empirical AD and PD datasets (before cleaning and standardizing) ensuring to preserve the original proportion of cell type nuclei and the disease-control ratio for each cell type, into two pairs of AD-PD subsets. Since the bisection reduced the effective observation sample size of each dataset to half, the downstream PLS fit enabled a suitable robustness check of derived gene modules. For each AD-PD subset pair, we ran our workflow pipeline (Fig. 1), steps A-D in parallel, resulting in 2 analogous sets of gene modules (a couple of 12 AD and 20 PD modules). We then performed Kendall's tau-b correlation across these modules, giving us 2 correlation matrices of dimension 12 x 20. We unraveled these matrices and calculated Pearson correlated (ρ) of the absolute τ_b values. We used absolute values since we were interested only in association strength, not direction. In doing so, we essentially compared the τ_b correlation levels between different gene module pairs derived from complementary subset pairs. The estimated mean ρ gave us an indicator for the

reliability of the quantified correlations between gene module pairs. A high ρ would signify a substantial degree of agreement between the association strengths of cross-disease gene modules, which in turn would attest to the robustness of the similarity analysis.

Differential gene expression

Differential Gene Expression is ubiquitously used in snRNA-seq analysis to identify genes that show statistically significant differences in expression levels between two conditions or groups[160]. It is a univariate method, meaning it examines each gene individually, thus losing key information hidden in gene co-expression patterns. We employed this traditional method to serve as an acid test for our new approach. Considering one gene at a time, we estimated the log-fold change of gene expression between the disease and control group[161]. Log fold change is calculated by taking the logarithm (usually base 2) of the ratio of the mean expression levels of a gene between two conditions. To determine the statistical significance of these changes, we used the nonparametric Wilcoxon's rank sum test, to help identify genes that had significantly different distributions in cases when compared to controls. We corrected for multiple testing using the Benjamini-Hochberg method[162]. This gave us a set of statistically significant differentially expressed genes (DEGs; FDR<0.05). We did this separately for every identified cell type within a snRNA-seq dataset (*scanpy* tool *tl.rank_genes_groups* with method= ‘wilcoxon’). The final DEGs are referred to as adDEGs for AD and pdDEGs for PD.

To estimate the pairwise association between cell-type-specific adDEGs and pdDEGs, we employed Kendall's tau-b correlation of the log-fold change values on the AND conjunction genes (cf. above). In doing so, we calculated a correlation matrix of dimension 6 (number of AD cell types) x 9 (number of PD cell types). To test for statistical significance, we performed a 1000-iteration permutation test. We randomized the disease label for each individual and subsequently identified a different set of adDGEs and pdDGEs. Based on the null hypothesis of a random association between AD deviant gene expression and PD deviant gene expression, we identified cell type pairs whose empirical association signal was significantly different in at least 95% of the 1000 permutation iterations.

Quantifying difference in association strengths between PLS and DGE derived conclusions: Welch’s t-test

To formally quantify the cross-disease association information extracted by our latent factor approach versus DGE, we turned to a statistical test that can compare the central tendencies of the respective correlation measures. Welch's t-test was a natural choice of method here as the number of correlated combinations being compared were different (240 τ_b gene module combinations from PLS and 54 τ_b cell type combinations from DGE), and the variances were not assumed to be equal[163,164]. Welch's t-test can be formally computed as:

$$t = \frac{\overrightarrow{X1} - \overrightarrow{X2}}{\sqrt{(s1^2\,/\,N1)\; + \;(s2^2\,/\,N2)}}$$

In our case, $\overrightarrow{X1}$ was the unraveled PLS correlation vector (1 x 240; cf. Fig. 2A) and $\overrightarrow{X2}$ was the unraveled DGE correlation vector (1 x 54; cf. Fig. 5), $s1^2$ and $s2^2$ were the variances of these vectors, $N1$ was the total number of cross-disease gene module pairs from the latent factor analysis and $N2$ was the number of cross-disease cell type pairs considered in the DGE analysis.

Identifying biological signaling pathways from gene modules: Gene Ontology enrichment analysis.

To query gene co-expression patterns regarding possible underlying biologically meaningful gene programs, we performed a gene set enrichment analysis (GSEA) [26]. This method is widely used by researchers to gain mechanistic insights into biology based on gene lists derived from omics experiments[165]. Here, we used the GSEApy python package [166], which itself uses Enrichr[167]. GSEApy is designed to extract statistically over-represented gene sets (example pathways) from a ranked gene list encompassing the whole genome. We focused on the gene ontology (GO) biological process (BP), molecular functions (MF), and cellular component (CC)[168,169] databases. Concretely, for each gene module, we fed the gene loadings across the entire protein-coding transcriptome recorded in our datasets to the enrichment tool (*gseapy.Prerank* tool with parameters rnk = gene loadings, min_size = 15, max_size = 1500, and permutation = 1000 for significance testing). We reported the pathways that had a FDR threshold of at most 0.05. This step was repeated identically and independently for all gene modules in each cell across all datasets.

To further verify that the gene set enrichment results were not an artifact of noise in the PLS modeling but rather had actual biological relevance, we turned to our permutation test-derived gene modules. In 1000 permutation iterations, we destroyed the relation between gene expression and disease label, and the thus extracted gene modules captured noise. We fed the gene loadings from these modules into our GSEA pipeline to verify the specificity of the empirical enriched terms.

Gene network visualization

GO terms are organized in the form of a hierarchical tree which can be downloaded here (OBO 1.4). This hierarchical organization often results in hundreds of hits from an enrichment analysis. One of the techniques widely used to crunch down this dense information is via network visualization [165,170]. This technique can help identify broad groupings of terms based on a chosen parameter of interest, for example, shared genes between different terms. We utilized Cytoscape [171] to create a structured network of disease-relevant GO biological processes identified by an enrichment analysis (cf. above). Each node in the network represented a GO BP hit. The edges were formed based on predefined relationships between nodes conditioned on shared genes and whether they were part of the same regulatory network. The resulting network layout (*yFiles.organic* layout) automatically clustered the enriched terms into biologically meaningful groups, allowing us to identify major functional themes that were shared between AD and PD.

Differential gene co-expression network

As a complementary analytical pipeline, we sought to explore the transcription profile of the RNA-seq datasets in a top-down gene co-expression network (GCN) approach. Specifically, we started with candidate genes mapped to AD or PD GWAS risk loci. Using these genes as seeds, we created networks of correlated genes, that is, we identified groups of genes whose differential expression change between disease and control closely matched a seed gene. Seeded differential gene co-expression networks (DGCN) have been previously used to identify regulatory changes in gene expressions across various conditions[55,57]. At its core, seeded GCNs capture how the gene expressions across the transcriptome are related to the transcription of the seeding gene. By taking a contrastive approach between disease and control (differential), the effects of housekeeping genes are eliminated, and the residual patterns of covariation can be attributed to the effects of a disease. In this study, we used disease-dictated DGCNs to analytically compare the effects of AD or PD on gene expression changes.

To identify our set of seed genes, we searched the most recent GWAS studies that reported AD or PD-associated genes significant at the whole genome level. In doing so, we identified 108 GWAS hits associated with AD[45] and 129 GWAS hits associated with PD[46]. These GWAS hits are the latest nominations in the largest AD or PD-targeted studies. Out of these 237 genes, 5 were common (CTSB, WNT3, BCKDK, HLA-DQA1, and HLA-DRB1) which gave us 232 unique genes. As not all genes are recorded in a snRNA-seq experiment, due to several reasons including technical dropouts or library preparation biases[172], we focused on the genes that are present in both AD and PD datasets. We found a total of 164 genes out of the 232 genes that were read in all considered datasets.

Next, for each individual dataset, first, we split them into disease and control groups based on the diagnosis labels provided. We further subdivided each of these groups into subgroups based on cell types. For each cell type ct, the co-expression vector for a single GWAS gene i with another gene j recorded in the snRNA-seq dataset was calculated as $\tau_b(e_i, e_j)$, where τ_b is the Kendall's tau-b correlation metric, e_i is the read count vector for i ∀ observations (nuclei) and e_j is the read count vector of j ∀ observations (nuclei). Evaluating τ_b across all recorded genes gave us: $g_{ct,i}^{AD} \in R$, where M is the number of genes common to both AD and PD datasets (M = 16,936). Thus, each element of the matrix $g_{ct,i}^{AD}$ is a numerical value between -1 and 1, capturing the degree of correlation of j with GWAS gene i. Stacking the vectors for all GWAS genes gave us gene co-expression matrices $G_{C,ct}^{AD} \in R^{N\times M}$ and $G_{D,ct}^{AD} \in R^{N\times M}$, for the control and disease groups respectively, where N was the number of GWAS genes (N=164). From this, we formally computed the differential gene co-expression matrix for a single cell type $G_{ct}^{AD} \in R^{N\times M}$ as follows,

$$G_{ct}^{AD} = G_C^{AD} - G_D^{AD}$$

Next, we systematically explored the mutual relationships between the DGCNs across different cell types without discriminating them based on disease. To this end, we employed a hierarchical clustering analysis. Our goal was to probe for clusters of cell types that featured similar genome-wide co-deviation of gene transcription. Concretely, we unraveled G_{ct}^{AD} into a vector $u_{ct}^{AD} \in R^{1\times NM}$, where $NM = 164 \times 16{,}936 = 2{,}777{,}504$, and combined them across 6 AD and 9 PD cell types to get $U \in R^{C\times NM}$, where $C = 15$. We computed the linkage matrix based on the Euclidean distance between two unraveled differential co-expression vectors for each cell type

(*scipy.cluster.hierarchy.linkage,* parameters method = 'average', metric = 'euclidean'). The linkage algorithm hierarchically clustered the 15 cell types, across AD and PD, with the cluster groups indicating cell types with the closest co-expression patterns. We visualized these clusters as a dendrogram in python (*scipy.cluster.hierarchy.dendrogram*).

We refined our clustering-based qualitative approach to rigorously quantify the association between cross disease DGCNs. Towards this goal, for the ith GWAS gene, we computed Kendall's tau-b correlation metric (τ_b) between $g_{ct,i}^{AD}$ and $g_{ct,i}^{PD}$. giving us the differential co-expression correlation matrix $G_i \in R^{6\times9}$. Each of these matrices encoded the similarity (-1 to +1; 0 being no association) between AD and PD disease co-expression networks for one gene. To congregate this cross-association information encoded by all the GWAS genes, we vertically stacked the unraveled matrix G_i (unraveled to $g_i \in R^{1\times54}$) into $P \in R^{164\times54}$. P, in essence, captures multiple modes of information condensed into one matrix: (i) differential gene co-expression between disease and control, (ii) quantified similarity of the expression changes between AD and PD stratified at the level of cell types, (iii) GWAS genes act as seeds not only for the disease in which they were identified but also for other disease. We finally distill this using a classical latent factor method, PCA. This uncovers linear combinations of cross-disease cell types (latent factors) that are most related in terms of their alterations to transcription in response to disease.

The number of significant latent factors that capture biologically meaningful information was determined using a principled permutation testing framework. In 100 permutation iterations, we randomly shuffled the unraveled correlation vector g_i, individually for each i, thus breaking the inherent meaningful patterns of covariation across cell type pairs. Across the 100 iterations, we fit individual PCA models and computed the explained variances of the derived components. Comparing the permutation variances with our empirical component variances, we retained 4 latent factors as statistically significant based on the 5/95% CI. These four embeddings are by construction uncorrelated and rank-ordered, with the first component capturing the highest amount of variance in P.

We conducted a bootstrap analysis on the extracted latent embeddings to formally assess the robustness of the cell type pairs that are closely associated with each other. Across 1000 bootstrap iterations, we sampled different rows (encapsulating all pairwise cell type co-deviations for a GWAS gene) with replacement to simulate a random seed gene collection that could have been sampled from the empirical population. We fit individual PCA models to each of the thus derived samples. To handle the inherent order invariance (changed sequence, especially for later components with small explained variance) and reflection invariance (sign flipping of derived singular vectors) of PCA components, we applied the Jonker-Volgenant algorithm for component matching and Pearson's correlation (ρ) for sign matching. The Jonker-Volgenant algorithm is a widely used technique[173] that can identify a one-to-one mapping of latent embeddings derived from two separate bootstrap iterations. This adjustment step was necessary as components that explained similar amounts of variance might swap positions in different sampling runs[174]. The similarity between a pair of components from two runs was scored using the cosine similarity (cf. above). Subsequently, we solved the optimization problem to maximize the similarity between component orderings from two runs across all pairwise combinations of the first 10 empirical and BS-derived PCA components (*scipy.optimize.linear_sum_assignment,* maximize = True). To align directionality, ρ was computed between the empirical PCA component loadings and the BS-

component loadings. For cases where ρ was less than 1, the latent vector loadings were multiplied by -1. Thus, in a complementary data-driven approach to our latent factor modeling, we could identify combinations of cell types that had the closest associations of gene expression changes between disease and control states.

Resource Availability

Data Availability

The snRNA-seq PFC data originated from Mathys, H. et al. Single-cell transcriptomic analysis of Alzheimer's disease. Nature 570, 332–337 (2019), are available through Synapse (https://www.synapse.org/Synapse:syn18485175) under the doi 10.7303/syn184851755[30]. The data is available under controlled use conditions set by human privacy regulations.

The snRNA-seq MTG data originating from Gabitto, M. I. et al. Integrated multimodal cell atlas of Alzheimer's disease. Nature Neuroscience, 1–18, is available through SEA-AD consortium's web portal at SEA-AD.org. Sequencing data are available through controlled access at Sage Bionetworks (accession syn26223298). Sage Bionetworks provide instructions for access to data on the AD Knowledge Portal.

The snRNA-seq substantia nigra data originating from Kamath, T. et al. Single-cell genomic profiling of human dopamine neurons identifies a population that selectively degenerates in Parkinson's disease. Nature Neuroscience, 25(5) is available from Single Cell Portal (https://singlecell.broadinstitute.org/single_cell/study/SCP1768/)

The snRNA-seq midbrain data originating from Smajić, S. et al. Single-cell sequencing of the human midbrain reveals glial activation and a Parkinson-specific neuronal state. Brain, 145(3) is available for download from the Gene Expression Omnibus (GEO) with accession number GSE157783.

All individual numerical values underlying the summary data presented in the figures, along with the complete code utilized for their generation, will be made openly accessible and deposited in an established open-access repository, such as Zenodo (https://zenodo.org), upon publication of this work.

Code Availability

Our code will be made available in GitHub on publication at: https://github.com/dblabs-mcgill-mila/AD-PD-overlap-study.

Acknowledgements

ROSMAP is supported by P30AG10161, P30AG72975, R01AG15819, R01AG17917, U01AG46152, and U01AG61356. ROSMAP resources can be requested at https://www.radc.rush.edu. DB was supported by the Brain Canada Foundation, through the Canada Brain Research Fund, with the financial support of Health Canada, National Institutes of

Health (NIH R01 AG068563A, NIH R01 DA053301-01A1, NIH R01 MH129858-01A1), the Canadian Institute of Health Research (CIHR 438531, CIHR 470425), the Healthy Brains Healthy Lives initiative (Canada First Research Excellence fund), the IVADO R3AI initiative (Canada First Research Excellence fund), and by the CIFAR Artificial Intelligence Chairs program (Canada Institute for Advanced Research).

Author contributions

AB and DB conceptualized the project, planned the experiments and analyzed the results. All authors helped write the manuscript and analyze the results. DB led data analysis.

Ethics declarations

Competing interests

D.B. is an equity holder at MindState Design Labs, USA. The authors declare no other competing interests.

References

1. Bloem BR, Okun MS, Klein C. Parkinson's disease. *The Lancet*. 2021;397(10291):2284-2303. doi:10.1016/S0140-6736(21)00218-X

2. Gustavsson A, Norton N, Fast T, et al. Global estimates on the number of persons across the Alzheimer's disease continuum. *Alzheimer's & Dementia*. 2023;19(2):658-670. doi:10.1002/alz.12694

3. Melnikova I. Therapies for Alzheimer's disease. *Nature Reviews Drug Discovery*. 2007;6(5):341-342. doi:10.1038/nrd2314

4. Kamath T, Macosko EZ. Insights into Neurodegeneration in Parkinson's Disease from Single-Cell and Spatial Genomics. *Movement Disorders*. 2023;38(4):518-525. doi:10.1002/mds.29374

5. Albers MW, Gilmore GC, Kaye J, et al. At the interface of sensory and motor dysfunctions and Alzheimer's disease. *Alzheimer's & Dementia*. 2015;11(1):70-98.

6. Oldham MC, Konopka G, Iwamoto K, et al. Functional organization of the transcriptome in human brain. *Nat Neurosci*. 2008;11(11):1271-1282. doi:10.1038/nn.2207

7. Twohig D, Nielsen HM. α-synuclein in the pathophysiology of Alzheimer's disease. *Molecular Neurodegeneration*. 2019;14(1):23. doi:10.1186/s13024-019-0320-x

8. Aarsland D, Batzu L, Halliday GM, et al. Parkinson disease-associated cognitive impairment. *Nat Rev Dis Primers*. 2021;7(1):1-21. doi:10.1038/s41572-021-00280-3

9. Cummings J, Lee G, Ritter A, Sabbagh M, Zhong K. Alzheimer's disease drug development pipeline: 2020. *Alzheimer's & Dementia: Translational Research & Clinical Interventions*. 2020;6(1):e12050. doi:10.1002/trc2.12050

10. THE BRAINSTORM CONSORTIUM, Anttila V, Bulik-Sullivan B, et al. Analysis of shared heritability in common disorders of the brain. *Science*. 2018;360(6395):eaap8757. doi:10.1126/science.aap8757

11. Wightman DP, Savage JE, Tissink E, Romero C, Jansen IE, Posthuma D. The genetic overlap between Alzheimer's disease, amyotrophic lateral sclerosis, Lewy body dementia, and Parkinson's disease. *Neurobiology of Aging*. 2023;127:99-112. doi:10.1016/j.neurobiolaging.2023.03.004

12. Sriram Balusu, Praschberger R, Lauwers E. Neurodegeneration cell per cell. *Neuron*. 2023;111(6):767-786. doi:10.1016/j.neuron.2023.01.016

13. Zhang X, Gao F, Wang D, et al. Tau Pathology in Parkinson's Disease. *Front Neurol*. 2018;9:809. doi:10.3389/fneur.2018.00809

14. Aarsland D, Kurz MW. The epidemiology of dementia associated with Parkinson disease. *J Neurol Sci*. 2010;289(1-2):18-22. doi:10.1016/j.jns.2009.08.034

15. Schneider JA, Arvanitakis Z, Leurgans SE, Bennett DA. The Neuropathology of Probable Alzheimer's Disease and Mild Cognitive Impairment. *Ann Neurol*. 2009;66(2):200-208. doi:10.1002/ana.21706

16. Schneider JA, Arvanitakis Z, Yu L, Boyle PA, Leurgans SE, Bennett DA. Cognitive impairment, decline and fluctuations in older community-dwelling subjects with Lewy bodies. *Brain*. 2012;135(10):3005-3014. doi:10.1093/brain/aws234

17. Schneider JA, Li JL, Li Y, Wilson RS, Kordower JH, Bennett DA. Substantia nigra tangles are related to gait impairment in older persons. *Annals of Neurology*. 2006;59(1):166-173. doi:10.1002/ana.20723

18. Armstrong RA, Lantos PL, Cairns NJ. Overlap between neurodegenerative disorders. *Neuropathology*. 2005;25(2):111-124. doi:10.1111/j.1440-1789.2005.00605.x

19. Perl DP, Warren CO, Calne D. Alzheimer's disease and parkinson's disease: Distinct entities or extremes of a spectrum of neurodegeneration? *Annals of Neurology*. 1998;44(S1):S19-S31. doi:10.1002/ana.410440705

20. Desikan RS, Schork AJ, Wang Y, et al. Genetic overlap between Alzheimer's disease and Parkinson's disease at the MAPT locus. *Mol Psychiatry*. 2015;20(12):1588-1595. doi:10.1038/mp.2015.6

21. Sadeghi I, Gispert JD, Palumbo E, et al. Brain transcriptomic profiling reveals common alterations across neurodegenerative and psychiatric disorders. *Computational and Structural Biotechnology Journal*. 2022;20:4549-4561. doi:10.1016/j.csbj.2022.08.037

22. Wingo TS, Liu Y, Gerasimov ES, et al. Shared mechanisms across the major psychiatric and neurodegenerative diseases. *Nat Commun*. 2022;13(1):4314. doi:10.1038/s41467-022-31873-5

23. Le Bars S, Glaab E. Single-Cell Cortical Transcriptomics Reveals Common and Distinct Changes in Cell-Cell Communication in Alzheimer's and Parkinson's Disease. *Mol Neurobiol*. 2025;62(3):2655-2673. doi:10.1007/s12035-024-04419-7

24. Barabási AL, Gulbahce N, Loscalzo J. Network medicine: a network-based approach to human disease. *Nat Rev Genet*. 2011;12(1):56-68. doi:10.1038/nrg2918

25. Crow M, Gillis J. Co-expression in Single-Cell Analysis: Saving Grace or Original Sin? *Trends in Genetics*. 2018;34(11):823-831. doi:10.1016/j.tig.2018.07.007

26. Subramanian A, Tamayo P, Mootha VK, et al. Gene set enrichment analysis: A knowledge-based approach for interpreting genome-wide expression profiles. *Proceedings of the National Academy of Sciences*. 2005;102(43):15545-15550. doi:10.1073/pnas.0506580102

27. Zhang B, Horvath S. A General Framework for Weighted Gene Co-Expression Network Analysis. *Statistical Applications in Genetics and Molecular Biology*. 2005;4(1). doi:10.2202/1544-6115.1128

28. Gerstein MB, Kundaje A, Hariharan M, et al. Architecture of the human regulatory network derived from ENCODE data. *Nature*. 2012;489(7414):91-100. doi:10.1038/nature11245

29. Saelens W, Cannoodt R, Saeys Y. A comprehensive evaluation of module detection methods for gene expression data. *Nat Commun*. 2018;9(1):1090. doi:10.1038/s41467-018-03424-4

30. Mathys H, Davila-Velderrain J, Peng Z, et al. Single-cell transcriptomic analysis of Alzheimer's disease. *Nature*. 2019;570(7761):332-337. doi:10.1038/s41586-019-1195-2

31. Gabitto MI, Travaglini KJ, Rachleff VM, et al. Integrated multimodal cell atlas of Alzheimer's disease. *Nat Neurosci*. Published online October 14, 2024:1-18. doi:10.1038/s41593-024-01774-5

32. Pak V, Adewale Q, Bzdok D, Dadar M, Zeighami Y, Iturria-Medina Y. Distinctive whole-brain cell types predict tissue damage patterns in thirteen neurodegenerative conditions. Fornito A, Wong ML, eds. *eLife*. 2024;12:RP89368. doi:10.7554/eLife.89368

33. Xiong X, James BT, Boix CA, et al. Epigenomic dissection of Alzheimer's disease pinpoints causal variants and reveals epigenome erosion. *Cell*. 2023;186(20):4422-4437.e21. doi:10.1016/j.cell.2023.08.040

34. Mathys H, Peng Z, Boix CA, et al. Single-cell atlas reveals correlates of high cognitive function, dementia, and resilience to Alzheimer's disease pathology. *Cell*. 2023;186(20):4365-4385.e27. doi:10.1016/j.cell.2023.08.039

35. Smajić S, Prada-Medina CA, Landoulsi Z, et al. Single-cell sequencing of human midbrain reveals glial activation and a Parkinson-specific neuronal state. *Brain*. 2022;145(3):964-978. doi:10.1093/brain/awab446

36. Zhu B, Park JM, Coffey S, et al. *Single-Cell Transcriptomic and Proteomic Analysis of Parkinson's Disease Brains*. Neuroscience; 2022. doi:10.1101/2022.02.14.480397

37. Kamath T, Abdulraouf A, Burris SJ, et al. Single-cell genomic profiling of human dopamine neurons identifies a population that selectively degenerates in Parkinson's disease. *Nat Neurosci*. 2022;25(5):588-595. doi:10.1038/s41593-022-01061-1

38. Bzdok D, Ioannidis JPA. Exploration, Inference, and Prediction in Neuroscience and Biomedicine. *Trends in Neurosciences*. 2019;42(4):251-262. doi:10.1016/j.tins.2019.02.001

39. Hodgson L, Li Y, Iturria-Medina Y, et al. Supervised latent factor modeling isolates cell-type-specific transcriptomic modules that underlie Alzheimer's disease progression. *Commun Biol*. 2024;7(1):1-19. doi:10.1038/s42003-024-06273-8

40. Lopez R, Regier J, Cole MB, Jordan MI, Yosef N. Deep generative modeling for single-cell transcriptomics. *Nat Methods*. 2018;15(12):1053-1058. doi:10.1038/s41592-018-0229-2

41. Bennett DA, Buchman AS, Boyle PA, et al. Religious Orders Study and Rush Memory and Aging Project. *Journal of Alzheimer's Disease*. 2018;64(s1):S161-S189. doi:10.3233/JAD-179939

42. Moon KR, van Dijk D, Wang Z, et al. Visualizing Structure and Transitions in High-Dimensional Biological Data. *Nat Biotechnol*. 2019;37(12):1482-1492. doi:10.1038/s41587-019-0336-3

43. Gill R, Datta S, Datta S. A statistical framework for differential network analysis from microarray data. *BMC Bioinformatics*. 2010;11(1):95. doi:10.1186/1471-2105-11-95

44. Lai Y, Wu B, Chen L, Zhao H. A statistical method for identifying differential gene–gene co-expression patterns. *Bioinformatics*. 2004;20(17):3146-3155. doi:10.1093/bioinformatics/bth379

45. Bellenguez C, Küçükali F, Jansen IE, et al. New insights into the genetic etiology of Alzheimer's disease and related dementias. *Nat Genet*. 2022;54(4):412-436. doi:10.1038/s41588-022-01024-z

46. Nalls MA, Blauwendraat C, Vallerga CL, et al. Identification of novel risk loci, causal insights, and heritable risk for Parkinson's disease: a meta-genome wide association study. *Lancet Neurol*. 2019;18(12):1091-1102. doi:10.1016/S1474-4422(19)30320-5

47. Billingsley KJ, Bandres-Ciga S, Saez-Atienzar S, Singleton AB. Genetic risk factors in Parkinson's disease. *Cell Tissue Res*. 2018;373(1):9-20. doi:10.1007/s00441-018-2817-y

48. Reynolds RH, Botía J, Nalls MA, Hardy J, Gagliano Taliun SA, Ryten M. Moving beyond neurons: the role of cell type-specific gene regulation in Parkinson's disease heritability. *npj Parkinsons Dis*. 2019;5(1):1-14. doi:10.1038/s41531-019-0076-6

49. Carbon S, Ireland A, Mungall CJ, et al. AmiGO: online access to ontology and annotation data. *Bioinformatics*. 2009;25(2):288-289. doi:10.1093/bioinformatics/btn615

50. Gan L, Cookson MR, Petrucelli L, La Spada AR. Converging pathways in neurodegeneration, from genetics to mechanisms. *Nat Neurosci*. 2018;21(10):1300-1309. doi:10.1038/s41593-018-0237-7

51. Glass CK, Saijo K, Winner B, Marchetto MC, Gage FH. Mechanisms Underlying Inflammation in Neurodegeneration. *Cell*. 2010;140(6):918-934. doi:10.1016/j.cell.2010.02.016

52. Lin MT, Beal MF. Mitochondrial dysfunction and oxidative stress in neurodegenerative diseases. *Nature*. 2006;443(7113):787-795. doi:10.1038/nature05292

53. Lo EH. Degeneration and repair in central nervous system disease. *Nat Med*. 2010;16(11):1205-1209. doi:10.1038/nm.2226

54. Soto C, Pritzkow S. Protein misfolding, aggregation, and conformational strains in neurodegenerative diseases. *Nat Neurosci*. 2018;21(10):1332-1340. doi:10.1038/s41593-018-0235-9

55. Langfelder P, Horvath S. WGCNA: an R package for weighted correlation network analysis. *BMC Bioinformatics*. 2008;9(1):559. doi:10.1186/1471-2105-9-559

56. Roy S, Lagree S, Hou Z, Thomson JA, Stewart R, Gasch AP. Integrated Module and Gene-Specific Regulatory Inference Implicates Upstream Signaling Networks. *PLOS Computational Biology*. 2013;9(10):e1003252. doi:10.1371/journal.pcbi.1003252

57. Watson M. CoXpress: differential co-expression in gene expression data. *BMC Bioinformatics*. 2006;7(1):509. doi:10.1186/1471-2105-7-509

58. Szwedo AA, Dalen I, Pedersen KF, et al. and Impact Cognitive Decline in Parkinson’s Disease: A 10-Year Population-Based Study. *Movement Disorders*. 2022;37(5):1016-1027. doi:10.1002/mds.28932

59. Zenuni H, Bovenzi R, Bissacco J, et al. Clinical and neurochemical correlates of the *APOE* genotype in early-stage Parkinson’s disease. *Neurobiology of Aging*. 2023;131:24-28. doi:10.1016/j.neurobiolaging.2023.07.011

60. Davis AA, Inman CE, Wargel ZM, et al. APOE genotype regulates pathology and disease progression in synucleinopathy. *Science Translational Medicine*. 2020;12(529):eaay3069. doi:10.1126/scitranslmed.aay3069

61. Krüger R, Kuhn W, Müller T, et al. AlaSOPro mutation in the gene encoding α-synuclein in Parkinson’s disease. *Nat Genet*. 1998;18(2):106-108. doi:10.1038/ng0298-106

62. Devine MJ, Gwinn K, Singleton A, Hardy J. Parkinson’s disease and α-synuclein expression. *Movement Disorders*. 2011;26(12):2160-2168. doi:10.1002/mds.23948

63. Khan SS, LaCroix M, Boyle G, et al. Bidirectional modulation of Alzheimer phenotype by alpha-synuclein in mice and primary neurons. *Acta Neuropathol*. 2018;136(4):589-605. doi:10.1007/s00401-018-1886-z

64. Larson ME, Sherman MA, Greimel S, et al. Soluble α-Synuclein Is a Novel Modulator of Alzheimer's Disease Pathophysiology. *J Neurosci*. 2012;32(30):10253-10266. doi:10.1523/JNEUROSCI.0581-12.2012

65. Gao FB, Richter JD, Cleveland DW. Rethinking Unconventional Translation in Neurodegeneration. *Cell*. 2017;171(5):994-1000. doi:10.1016/j.cell.2017.10.042

66. Tai HC, Schuman EM. Ubiquitin, the proteasome and protein degradation in neuronal function and dysfunction. *Nat Rev Neurosci*. 2008;9(11):826-838. doi:10.1038/nrn2499

67. Bence NF, Sampat RM, Kopito RR. Impairment of the Ubiquitin-Proteasome System by Protein Aggregation. *Science*. 2001;292(5521):1552-1555. doi:10.1126/science.292.5521.1552

68. Qadir A, Kumar A, Nagpal R, Khan A, Wahi A, Jain P. Understanding the Ubiquitin Proteasome System: History and Revolution. In: Nandave M, Jain P, eds. *PROTAC-Mediated Protein Degradation: A Paradigm Shift in Cancer Therapeutics*. Springer Nature; 2024:1-20. doi:10.1007/978-981-97-5077-1_1

69. Millecamps S, Julien JP. Axonal transport deficits and neurodegenerative diseases. *Nat Rev Neurosci*. 2013;14(3):161-176. doi:10.1038/nrn3380

70. Andreu-Carbó M, Egoldt C, Velluz MC, Aumeier C. Microtubule damage shapes the acetylation gradient. *Nat Commun*. 2024;15(1):2029. doi:10.1038/s41467-024-46379-5

71. Naren P, Samim KS, Tryphena KP, et al. Microtubule acetylation dyshomeostasis in Parkinson's disease. *Translational Neurodegeneration*. 2023;12(1):20. doi:10.1186/s40035-023-00354-0

72. Diamond MI. Travels with tau prions. *Cytoskeleton*. 2024;81(1):83-88. doi:10.1002/cm.21806

73. Kaufman SK, Sanders DW, Thomas TL, et al. Tau Prion Strains Dictate Patterns of Cell Pathology, Progression Rate, and Regional Vulnerability In Vivo. *Neuron*. 2016;92(4):796-812. doi:10.1016/j.neuron.2016.09.055

74. Rauch JN, Olson SH, Gestwicki JE. Interactions between Microtubule-Associated Protein Tau (MAPT) and Small Molecules. *Cold Spring Harb Perspect Med*. 2017;7(7):a024034. doi:10.1101/cshperspect.a024034

75. Jaunmuktane Z, Brandner S. Invited Review: The role of prion-like mechanisms in neurodegenerative diseases. *Neuropathology and Applied Neurobiology*. 2020;46(6):522-545. doi:10.1111/nan.12592

76. Walker LC, Jucker M. The prion principle and Alzheimer's disease. *Science*. 2024;385(6715):1278-1279. doi:10.1126/science.adq5252

77. Guedes-Dias P, Holzbaur ELF. Axonal transport: Driving synaptic function. *Science*. 2019;366(6462):eaaw9997. doi:10.1126/science.aaw9997

78. Quntanilla RA, Tapia-Monsalves C. The Role of Mitochondrial Impairment in Alzheimer´s Disease Neurodegeneration: The Tau Connection. *Curr Neuropharmacol*. 2020;18(11):1076-1091. doi:10.2174/1570159X18666200525020259

79. Pellegrini L, Wetzel A, Grannó S, Heaton G, Harvey K. Back to the tubule: microtubule dynamics in Parkinson's disease. *Cell Mol Life Sci*. 2017;74(3):409-434. doi:10.1007/s00018-016-2351-6

80. Esteves AR, Arduino DM, Swerdlow RH, Oliveira C, Cardoso SM. Microtubule depolymerization potentiates alpha-synuclein oligomerization. *Front Aging Neurosci*. 2010;1. doi:10.3389/neuro.24.005.2009

81. Kumar A, Ganini D, Mason RP. Role of cytochrome c in α-synuclein radical formation: implications of α-synuclein in neuronal death in Maneb- and paraquat-induced model of Parkinson's disease. *Molecular Neurodegeneration*. 2016;11(1):70. doi:10.1186/s13024-016-0135-y

82. Camilleri A, Zarb C, Caruana M, et al. Mitochondrial membrane permeabilisation by amyloid aggregates and protection by polyphenols. *Biochim Biophys Acta*. 2013;1828(11):2532-2543. doi:10.1016/j.bbamem.2013.06.026

83. Cui J, Zhao S, Li Y, et al. Regulated cell death: discovery, features and implications for neurodegenerative diseases. *Cell Communication and Signaling*. 2021;19(1):120. doi:10.1186/s12964-021-00799-8

84. Datta SR, Dudek H, Tao X, et al. Akt phosphorylation of BAD couples survival signals to the cell-intrinsic death machinery. *Cell*. 1997;91(2):231-241. doi:10.1016/s0092-8674(00)80405-5

85. Erekat NS. Apoptosis and its Role in Parkinson's Disease. In: Stoker TB, Greenland JC, eds. *Parkinson's Disease: Pathogenesis and Clinical Aspects*. Codon Publications; 2018. Accessed August 1, 2024. http://www.ncbi.nlm.nih.gov/books/NBK536724/

86. Mochizuki H, Goto K, Mori H, Mizuno Y. Histochemical detection of apoptosis in Parkinson's disease. *J Neurol Sci*. 1996;137(2):120-123. doi:10.1016/0022-510x(95)00336-z

87. McKenzie AT, Moyon S, Wang M, et al. Multiscale network modeling of oligodendrocytes reveals molecular components of myelin dysregulation in Alzheimer's disease. *Mol Neurodegener*. 2017;12:82. doi:10.1186/s13024-017-0219-3

88. Agarwal D, Sandor C, Volpato V, et al. A single-cell atlas of the human substantia nigra reveals cell-specific pathways associated with neurological disorders. *Nat Commun*. 2020;11:4183. doi:10.1038/s41467-020-17876-0

89. Bartzokis G. Age-related myelin breakdown: a developmental model of cognitive decline and Alzheimer's disease. *Neurobiol Aging*. 2004;25(1):5-18; author reply 49-62. doi:10.1016/j.neurobiolaging.2003.03.001

90. Depp C, Sun T, Sasmita AO, et al. Myelin dysfunction drives amyloid-β deposition in models of Alzheimer's disease. *Nature*. 2023;618(7964):349-357. doi:10.1038/s41586-023-06120-6

91. Kenigsbuch M, Bost P, Halevi S, et al. A shared disease-associated oligodendrocyte signature among multiple CNS pathologies. *Nat Neurosci*. 2022;25(7):876-886. doi:10.1038/s41593-022-01104-7

92. Zhou Y, Song WM, Andhey PS, et al. Human and mouse single-nucleus transcriptomics reveal TREM2-dependent and TREM2-independent cellular responses in Alzheimer's disease. *Nat Med*. 2020;26(1):131-142. doi:10.1038/s41591-019-0695-9

93. Balali-Mood M, Naseri K, Tahergorabi Z, Khazdair MR, Sadeghi M. Toxic Mechanisms of Five Heavy Metals: Mercury, Lead, Chromium, Cadmium, and Arsenic. *Front Pharmacol*. 2021;12. doi:10.3389/fphar.2021.643972

94. Haidar Z, Fatema K, Shoily SS, Sajib AA. Disease-associated metabolic pathways affected by heavy metals and metalloid. *Toxicol Rep*. 2023;10:554-570. doi:10.1016/j.toxrep.2023.04.010

95. Li B, Xia M, Zorec R, Parpura V, Verkhratsky A. Astrocytes in heavy metal neurotoxicity and neurodegeneration. *Brain Res*. 2021;1752:147234. doi:10.1016/j.brainres.2020.147234

96. Huiliang Z, Mengzhe Y, Xiaochuan W, et al. Zinc induces reactive astrogliosis through ERK-dependent activation of Stat3 and promotes synaptic degeneration. *Journal of Neurochemistry*. 2021;159(6):1016-1027. doi:10.1111/jnc.15531

97. Gamez P, Caballero AB. Copper in Alzheimer's disease: Implications in amyloid aggregation and neurotoxicity. *AIP Advances*. 2015;5(9):092503. doi:10.1063/1.4921314

98. Pal A, Rani I, Pawar A, Picozza M, Rongioletti M, Squitti R. Microglia and Astrocytes in Alzheimer's Disease in the Context of the Aberrant Copper Homeostasis Hypothesis. *Biomolecules*. 2021;11(11):1598. doi:10.3390/biom11111598

99. Zhou Q, Zhang Y, Lu L, et al. Copper induces microglia-mediated neuroinflammation through ROS/NF-κB pathway and mitophagy disorder. *Food Chem Toxicol*. 2022;168:113369. doi:10.1016/j.fct.2022.113369

100. Uversky VN, Li J, Fink AL. Metal-triggered structural transformations, aggregation, and fibrillation of human alpha-synuclein. A possible molecular NK between Parkinson's disease and heavy metal exposure. *J Biol Chem*. 2001;276(47):44284-44296. doi:10.1074/jbc.M105343200

101. Sarell CJ, Wilkinson SR, Viles JH. Substoichiometric Levels of Cu2+ Ions Accelerate the Kinetics of Fiber Formation and Promote Cell Toxicity of Amyloid-β from Alzheimer Disease *. *Journal of Biological Chemistry*. 2010;285(53):41533-41540. doi:10.1074/jbc.M110.171355

102. Myhre O, Utkilen H, Duale N, Brunborg G, Hofer T. Metal Dyshomeostasis and Inflammation in Alzheimer's and Parkinson's Diseases: Possible Impact of Environmental Exposures. *Oxid Med Cell Longev*. 2013;2013:726954. doi:10.1155/2013/726954

103. Acosta-Cabronero J, Betts MJ, Cardenas-Blanco A, Yang S, Nestor PJ. In Vivo MRI Mapping of Brain Iron Deposition across the Adult Lifespan. *J Neurosci*. 2016;36(2):364-374. doi:10.1523/JNEUROSCI.1907-15.2016

104. Belaidi AA, Bush AI. Iron neurochemistry in Alzheimer's disease and Parkinson's disease: targets for therapeutics. *Journal of Neurochemistry*. 2016;139(S1):179-197. doi:10.1111/jnc.13425

105. Bjørklund G, Hofer T, Nurchi VM, Aaseth J. Iron and other metals in the pathogenesis of Parkinson's disease: Toxic effects and possible detoxification. *J Inorg Biochem*. 2019;199:110717. doi:10.1016/j.jinorgbio.2019.110717

106. Thomas GEC, Leyland LA, Schrag AE, Lees AJ, Acosta-Cabronero J, Weil RS. Brain iron deposition is linked with cognitive severity in Parkinson's disease. *J Neurol Neurosurg Psychiatry*. 2020;91(4):418-425. doi:10.1136/jnnp-2019-322042

107. Ward RJ, Zucca FA, Duyn JH, Crichton RR, Zecca L. The role of iron in brain ageing and neurodegenerative disorders. *Lancet Neurol*. 2014;13(10):1045-1060. doi:10.1016/S1474-4422(14)70117-6

108. He F, Sun YE. Glial cells more than support cells? *Int J Biochem Cell Biol*. 2007;39(4):661-665. doi:10.1016/j.biocel.2006.10.022

109. Kierdorf K, Erny D, Goldmann T, et al. Microglia emerge from erythromyeloid precursors via Pu.1- and Irf8-dependent pathways. *Nat Neurosci*. 2013;16(3):273-280. doi:10.1038/nn.3318

110. Prinz M, Mildner A. Microglia in the CNS: Immigrants from another world. *Glia*. 2011;59(2):177-187. doi:10.1002/glia.21104

111. Grabert K, Michoel T, Karavolos MH, et al. Microglial brain region−dependent diversity and selective regional sensitivities to aging. *Nat Neurosci*. 2016;19(3):504-516. doi:10.1038/nn.4222

112. Hickman S, Izzy S, Sen P, Morsett L, El Khoury J. Microglia in neurodegeneration. *Nat Neurosci*. 2018;21(10):1359-1369. doi:10.1038/s41593-018-0242-x

113. Siletti K, Hodge R, Mossi Albiach A, et al. Transcriptomic diversity of cell types across the adult human brain. *Science*. 2023;382(6667):eadd7046. doi:10.1126/science.add7046

114. Lenz KM, Nelson LH. Microglia and Beyond: Innate Immune Cells As Regulators of Brain Development and Behavioral Function. *Front Immunol*. 2018;9:698. doi:10.3389/fimmu.2018.00698

115. Tansey MG, Wallings RL, Houser MC, Herrick MK, Keating CE, Joers V. Inflammation and immune dysfunction in Parkinson disease. *Nat Rev Immunol*. 2022;22(11):657-673. doi:10.1038/s41577-022-00684-6

116. Khoury JE, Luster AD. Mechanisms of microglia accumulation in Alzheimer's disease: therapeutic implications. *Trends in Pharmacological Sciences*. 2008;29(12):626-632. doi:10.1016/j.tips.2008.08.004

117. Chen X, Firulyova M, Manis M, et al. Microglia-mediated T cell Infiltration Drives Neurodegeneration in Tauopathy. *Nature*. 2023;615(7953):668-677. doi:10.1038/s41586-023-05788-0

118. González H, Pacheco R. T-cell-mediated regulation of neuroinflammation involved in neurodegenerative diseases. *Journal of Neuroinflammation*. 2014;11(1):201. doi:10.1186/s12974-014-0201-8

119. Xu Y, Li Y, Wang C, et al. The reciprocal interactions between microglia and T cells in Parkinson's disease: a double-edged sword. *Journal of Neuroinflammation*. 2023;20(1):33. doi:10.1186/s12974-023-02723-y

120. Villanueva I, Alva-Sánchez C, Pacheco-Rosado J. The role of thyroid hormones as inductors of oxidative stress and neurodegeneration. *Oxid Med Cell Longev*. 2013;2013:218145. doi:10.1155/2013/218145

121. Ewins DL, Rossor MN, Butler J, Rogues PK, Mullen MJ, McGregor AM. Association between autoimmune thyroid disease and Familial Alzheimers disease. *Clinical Endocrinology*. 1991;35(1):93-96. doi:10.1111/j.1365-2265.1991.tb03502.x

122. Kalmijn S, Mehta KM, Pols HA, Hofman A, Drexhage HA, Breteler MM. Subclinical hyperthyroidism and the risk of dementia. The Rotterdam study. *Clin Endocrinol (Oxf)*. 2000;53(6):733-737. doi:10.1046/j.1365-2265.2000.01146.x

123. Mohammadi S, Dolatshahi M, Rahmani F. Shedding light on thyroid hormone disorders and Parkinson disease pathology: mechanisms and risk factors. *J Endocrinol Invest*. 2021;44(1):1-13. doi:10.1007/s40618-020-01314-5

124. Kim DK, Choi H, Lee W, et al. Brain hypothyroidism silences the immune response of microglia in Alzheimer's disease animal model. *Sci Adv*. 10(11):eadi1863. doi:10.1126/sciadv.adi1863

125. Seo BA, Kim D, Hwang H, et al. TRIP12 ubiquitination of glucocerebrosidase contributes to neurodegeneration in Parkinson's disease. *Neuron*. 2021;109(23):3758-3774.e11. doi:10.1016/j.neuron.2021.09.031

126. Hong S, Beja-Glasser VF, Nfonoyim BM, et al. Complement and microglia mediate early synapse loss in Alzheimer mouse models. *Science*. 2016;352(6286):712-716. doi:10.1126/science.aad8373

127. Song P, Peng W, Sauve V, et al. Parkinson's disease-linked parkin mutation disrupts recycling of synaptic vesicles in human dopaminergic neurons. *Neuron*. 2023;111(23):3775-3788.e7. doi:10.1016/j.neuron.2023.08.018

128. Tzioras M, Daniels MJD, Davies C, et al. Human astrocytes and microglia show augmented ingestion of synapses in Alzheimer's disease via MFG-E8. *CR Med*. 2023;4(9). doi:10.1016/j.xcrm.2023.101175

129. Das M, Mao W, Voskobiynyk Y, et al. Alzheimer risk-increasing TREM2 variant causes aberrant cortical synapse density and promotes network hyperexcitability in mouse models. *Neurobiol Dis*. 2023;186:106263. doi:10.1016/j.nbd.2023.106263

130. Filipello F, Morini R, Corradini I, et al. The Microglial Innate Immune Receptor TREM2 Is Required for Synapse Elimination and Normal Brain Connectivity. *Immunity*. 2018;48(5):979-991.e8. doi:10.1016/j.immuni.2018.04.016

131. Guo Y, Wei X, Yan H, et al. TREM2 deficiency aggravates α-synuclein-induced neurodegeneration and neuroinflammation in Parkinson's disease models. *FASEB J*. 2019;33(11):12164-12174. doi:10.1096/fj.201900992R

132. Shafi S, Singh A, Ibrahim AM, Alhajri N, Abu Izneid T, Pottoo FH. Role of triggering receptor expressed on myeloid cells 2 (TREM2) in neurodegenerative dementias. *Eur J Neurosci*. 2021;53(10):3294-3310. doi:10.1111/ejn.15215

133. Alexander JJ, Anderson AJ, Barnum SR, Stevens B, Tenner AJ. The complement cascade: Yin–Yang in neuroinflammation – neuro-protection and -degeneration. *Journal of Neurochemistry*. 2008;107(5):1169-1187. doi:10.1111/j.1471-4159.2008.05668.x

134. Carbutt S, Duff J, Yarnall A, Burn DJ, Hudson G. Variation in complement protein C1q is not a major contributor to cognitive impairment in Parkinson's disease. *Neurosci Lett*. 2015;594:66-69. doi:10.1016/j.neulet.2015.03.048

135. Liu L, MacKenzie KR, Putluri N, Maletić-Savatić M, Bellen HJ. The Glia-Neuron Lactate Shuttle and Elevated ROS Promote Lipid Synthesis in Neurons and Lipid Droplet Accumulation in Glia via APOE/D. *Cell Metabolism*. 2017;26(5):719-737.e6. doi:10.1016/j.cmet.2017.08.024

136. Hallett PJ, Engelender S, Isacson O. Lipid and immune abnormalities causing age-dependent neurodegeneration and Parkinson's disease. *Journal of Neuroinflammation*. 2019;16(1):153. doi:10.1186/s12974-019-1532-2

137. Sienski G, Narayan P, Bonner JM, et al. APOE4 disrupts intracellular lipid homeostasis in human iPSC-derived glia. *Science Translational Medicine*. 2021;13(583):eaaz4564. doi:10.1126/scitranslmed.aaz4564

138. Skene NG, Bryois J, Bakken TE, et al. Genetic identification of brain cell types underlying schizophrenia. *Nat Genet*. 2018;50(6):825-833. doi:10.1038/s41588-018-0129-5

139. Smillie CS, Biton M, Ordovas-Montanes J, et al. Intra- and Inter-cellular Rewiring of the Human Colon during Ulcerative Colitis. *Cell*. 2019;178(3):714-730. doi:10.1016/j.cell.2019.06.029

140. The GTEx Consortium. The GTEx Consortium atlas of genetic regulatory effects across human tissues. *Science*. 2020;369(6509):1318-1330. doi:10.1126/science.aaz1776

141. Eraslan G, Drokhlyansky E, Anand S, et al. Single-nucleus cross-tissue molecular reference maps toward understanding disease gene function. *Science*. 2022;376(6594):eabl4290. doi:10.1126/science.abl4290

142. Habib N, Avraham-Davidi I, Basu A, et al. Massively parallel single-nucleus RNA-seq with DroNc-seq. *Nat Methods*. 2017;14(10):955-958. doi:10.1038/nmeth.4407

143. Hughes G. On the mean accuracy of statistical pattern recognizers. *IEEE Transactions on Information Theory*. 1968;14(1):55-63. doi:10.1109/TIT.1968.1054102

144. Jia W, Sun M, Lian J, Hou S. Feature dimensionality reduction: a review. *Complex Intell Syst*. 2022;8(3):2663-2693. doi:10.1007/s40747-021-00637-x

145. Abdi H. Partial least squares regression and projection on latent structure regression (PLS Regression). *WIREs Computational Statistics*. 2010;2(1):97-106. doi:10.1002/wics.51

146. Barker M, Rayens W. Partial least squares for discrimination. *Journal of Chemometrics*. 2003;17(3):166-173. doi:10.1002/cem.785

147. Wold HOA. Nonlinear Iterative Partial Least Squares (NIPALS) Modelling: Some Current Developments. In: ; 1973. https://api.semanticscholar.org/CorpusID:118962244

148. Cloarec O. Can we beat over–fitting? *Journal of Chemometrics*. 2014;28(8):610-614. doi:10.1002/cem.2602

149. Lee LC, Liong CY, Jemain AA. Partial least squares-discriminant analysis (PLS-DA) for classification of high-dimensional (HD) data: a review of contemporary practice strategies and knowledge gaps. *Analyst*. 2018;143(15):3526-3539. doi:10.1039/C8AN00599K

150. Krishnaswami SR, Grindberg RV, Novotny M, et al. Using single nuclei for RNA-seq to capture the transcriptome of postmortem neurons. *Nat Protoc*. 2016;11(3):499-524. doi:10.1038/nprot.2016.015

151. Ma P, Liu X, Xu Z, et al. Joint profiling of gene expression and chromatin accessibility during amphioxus development at single-cell resolution. *Cell Reports*. 2022;39(12). doi:10.1016/j.celrep.2022.110979

152. Ahlmann-Eltze C, Huber W. Comparison of transformations for single-cell RNA-seq data. *Nat Methods*. 2023;20(5):665-672. doi:10.1038/s41592-023-01814-1

153. Zhu Y, Wang L, Yin Y, Yang E. Systematic analysis of gene expression patterns associated with postmortem interval in human tissues. *Sci Rep*. 2017;7(1):5435. doi:10.1038/s41598-017-05882-0

154. Ferreira PG, Muñoz-Aguirre M, Reverter F, et al. The effects of death and post-mortem cold ischemia on human tissue transcriptomes. *Nat Commun*. 2018;9(1):490. doi:10.1038/s41467-017-02772-x

155. Miller KL, Alfaro-Almagro F, Bangerter NK, et al. Multimodal population brain imaging in the UK Biobank prospective epidemiological study. *Nat Neurosci*. 2016;19(11):1523-1536. doi:10.1038/nn.4393

156. Spreng RN, Dimas E, Mwilambwe-Tshilobo L, et al. The default network of the human brain is associated with perceived social isolation. *Nat Commun*. 2020;11(1):6393. doi:10.1038/s41467-020-20039-w

157. Rodríguez-Pérez R, Fernández L, Marco S. Overoptimism in cross-validation when using partial least squares-discriminant analysis for omics data: a systematic study. *Anal Bioanal Chem*. 2018;410(23):5981-5992. doi:10.1007/s00216-018-1217-1

158. de Boves Harrington P. Statistical validation of classification and calibration models using bootstrapped Latin partitions. *TrAC Trends in Analytical Chemistry*. 2006;25(11):1112-1124. doi:10.1016/j.trac.2006.10.010

159. Arndt S, Turvey C, Andreasen NC. Correlating and predicting psychiatric symptom ratings: Spearman's r versus Kendall's tau correlation. *J Psychiatr Res*. 1999;33(2):97-104. doi:10.1016/s0022-3956(98)90046-2

160. Stark R, Grzelak M, Hadfield J. RNA sequencing: the teenage years. *Nat Rev Genet*. 2019;20(11):631-656. doi:10.1038/s41576-019-0150-2

161. Li Y, Ge X, Peng F, Li W, Li JJ. Exaggerated false positives by popular differential expression methods when analyzing human population samples. *Genome Biology*. 2022;23(1):79. doi:10.1186/s13059-022-02648-4

162. Benjamini Y, Hochberg Y. Controlling the False Discovery Rate: A Practical and Powerful Approach to Multiple Testing. *Journal of the Royal Statistical Society: Series B (Methodological)*. 1995;57(1):289-300. doi:10.1111/j.2517-6161.1995.tb02031.x

163. Ruxton GD. The unequal variance t-test is an underused alternative to Student's t-test and the Mann–Whitney U test. *Behavioral Ecology*. 2006;17(4):688-690. doi:10.1093/beheco/ark016

164. WELCH BL. THE GENERALIZATION OF 'STUDENT'S' PROBLEM WHEN SEVERAL DIFFERENT POPULATION VARLANCES ARE INVOLVED. *Biometrika*. 1947;34(1-2):28-35. doi:10.1093/biomet/34.1-2.28

165. Reimand J, Isserlin R, Voisin V, et al. Pathway enrichment analysis and visualization of omics data using g:Profiler, GSEA, Cytoscape and EnrichmentMap. *Nat Protoc*. 2019;14(2):482-517. doi:10.1038/s41596-018-0103-9

166. Fang Z, Liu X, Peltz G. GSEApy: a comprehensive package for performing gene set enrichment analysis in Python. *Bioinformatics*. 2023;39(1):btac757. doi:10.1093/bioinformatics/btac757

167. Chen EY, Tan CM, Kou Y, et al. Enrichr: interactive and collaborative HTML5 gene list enrichment analysis tool. *BMC Bioinformatics*. 2013;14(1):128. doi:10.1186/1471-2105-14-128

168. Ashburner M, Ball CA, Blake JA, et al. Gene Ontology: tool for the unification of biology. *Nat Genet*. 2000;25(1):25-29. doi:10.1038/75556

169. The Gene Ontology Consortium, Aleksander SA, Balhoff J, et al. The Gene Ontology knowledgebase in 2023. *Genetics*. 2023;224(1):iyad031. doi:10.1093/genetics/iyad031

170. Merico D, Isserlin R, Stueker O, Emili A, Bader GD. Enrichment Map: A Network-Based Method for Gene-Set Enrichment Visualization and Interpretation. *PLOS ONE*. 2010;5(11):e13984. doi:10.1371/journal.pone.0013984

171. Shannon P, Markiel A, Ozier O, et al. Cytoscape: a software environment for integrated models of biomolecular interaction networks. *Genome Res*. 2003;13(11):2498-2504. doi:10.1101/gr.1239303

172. Heumos L, Schaar AC, Lance C, et al. Best practices for single-cell analysis across modalities. *Nat Rev Genet*. 2023;24(8):550-572. doi:10.1038/s41576-023-00586-w

173. Crouse DF. On implementing 2D rectangular assignment algorithms. *IEEE Transactions on Aerospace and Electronic Systems*. 2016;52(4):1679-1696. doi:10.1109/TAES.2016.140952

174. Saltoun K, Adolphs R, Paul LK, et al. Dissociable brain structural asymmetry patterns reveal unique phenome-wide profiles. *Nat Hum Behav*. 2023;7(2):251-268. doi:10.1038/s41562-022-01461-0

Supplementary tables

Click here to access/download
Supplementary Material
Supplementary_table.xlsx

Supplementary figures

Click here to access/download
Supplementary Material
Supplementary.docx

Cover letter
Click here to access/download;Personal Cover;cover_letter_AD-PD.docx

neuro
Montreal Neurological Institute-Hospital

Danilo Bzdok, MD, PhD
Anwesha Bhattacharya
Montreal Neurological Institute
3801 rue University, Bureau #872D
Montréal (Québec) H3A 2B4
Canada

Xun Xu, Editor-in-Chief**,** *Giga Science*,

Dear Xun Xu, Dear members of the editorial board,

We are pleased to submit our manuscript, titled "Cell type transcriptomics reveal shared genetic mechanisms in Alzheimer's and Parkinson's disease," for consideration as an Article in *Giga Science*. The authors have no competing interests.

Alzheimer's disease (AD) and Parkinson's disease (PD) have been considered separate disease classes in research and clinical practice for decades, largely due to differences in age of onset, primary brain regions affected, distinct protein aggregates and clinical progression. Indeed, a *Neuron* review recently concluded that "There is intriguingly little overlap between the risk genes for AD and PD, providing genetic evidence for different disease onset and progression mechanisms" (Sriram Balusu et al., 2023). However, neuropathological evidence reveals overlapping protein aggregates occurring above chance with α-synuclein in AD brains and tau and amyloid in PD brains.

In the present investigation, by tailoring machine learning to emerging big biomedical data resources, we systematically revisit the problem of identifying candidate molecular mechanisms that might overlap between AD and PD. In contrast to prevailing focus on individual genes in mainstream single-cell genomics research, we show that transcriptome-wide gene co-expression networks yield substantial genetic overlap. For the first time, we can isolate cell type specific biological processes that are aligned between AD and PD: Axon microtubule stabilizing gene networks driven by the MAPT gene in neurons, for example, stick out. This gene encodes protein tau, abnormal aggregates of which are known to have prion-like behavior. We find further molecular signaling pathway overlaps between AD and PD involving stress response, glucose metabolism, metal ion homeostasis and mitochondrial energy metabolism. To our knowledge, this study provides the first glimpse of the genetic controls at a sub-cellular resolution, leading to measurable alterations in gene transcription shared in AD and PD. We also validate our primary data driven findings and conclusions using independent AD and PD transcriptomics datasets.

Given the expected 3-4-fold increase in neurodegeneration cases by 2050, our research could lay important foundations for therapeutic interventions that can improve the well-being of older adults. While informative, incumbent methods, like genome-wide association studies and single-gene transcriptomic analyses, are inherently limited, as they do not fully capture the complexity of gene regulation which occurs within tightly regulated networks of co-expressed genes. Overcoming these limitations, we place a tool in the hands of single-cell genomics scientists to compare any pair of diseases in the future.

All results and information presented in this manuscript are entirely novel and have not been published, submitted elsewhere, or shared on online platforms. Additionally, we have not previously discussed this work with a *Giga Science* editor.

We thank you in advance for your time and consideration.

Yours sincerely,

Danilo Bzdok, M.D., Ph.D.
MNI – Montreal Neurological Institute
MILA – Quebec Artificial Intelligence Institute

Anwesha Bhattacharya, Ph.D. candidate.
MNI – Montreal Neurological Institute
Mila – Quebec Artificial Intelligence Institute
