# Supplementary material for: Cell type transcriptomic modules reveal shared molecular mechanisms in Alzheimer’s and Parkinson’s disease
Source: Gigascience. 2026 May 21;15:giag059. doi: 10.1093/gigascience/giag059 (PMC13289754; doi:10.1093/gigascience/giag059)
Supplement: giag059_GIGA-D-25-00403_Revision_1 [file giag059_giga-d-25-00403_revision_1.pdf]

GigaScience

Cell type transcriptomic modules reveal shared molecular mechanisms in Alzheimer's and Parkinson's disease

--Manuscript Draft--

Manuscript Number:	GIGA-D-25-00403R1
Full Title:	Cell type transcriptomic modules reveal shared molecular mechanisms in Alzheimer's and Parkinson's disease
Article Type:	Research
Funding Information:	
Abstract:	Historically, Alzheimer’s disease (AD) and Parkinson’s disease (PD) have been investigated as two distinct disorders of the brain. However, a few similarities in neuropathology and clinical symptoms have been documented over the years. Traditional single-gene centric studies, such as differential gene expression analyses, have struggled to unravel the molecular basis for the observed pathological links between AD and PD. To address this, we tailor a latent factor framework to analyze synchronous gene co-expression at sub-cell-type resolution. Utilizing large, single-nucleus transcriptomics datasets in AD (70,634 nuclei) and PD (340,902 nuclei) from postmortem human brains, we systematically extract and juxtapose disease-critical molecular signatures in the brain. Our transcriptomic analysis reveals shared molecular programs between AD and PD that systematically localize to specific glial and neuronal cell types. In neurons, convergent gene groups in AD and PD relate to cytoskeletal dynamics and mitochondrial stress emerge as key mechanisms underlying disease pathology. Similarly, overlapping gene groups in microglia modules implicate T cell activation mechanisms and synapse pruning pathways. In parallel, AD- and PD-associated genes in astrocytes are involved in heavy metal processing; oligodendrocytes highlight convergent dysregulation in myelin synthesis. In addition, our analysis reveals APOE, an AD GWAS gene, has disease predictive roles in PD-associated gene modules. Conversely, SNCA, a PD GWAS gene, emerges within AD associated gene modules. Our multi-module sub-cell-type approach offers unique insights into the molecular basis of shared neuropathology in AD and PD.
Corresponding Author:	Anwesha Bhattacharya<br>McGill University<br>Montreal, Quebec CANADA
Corresponding Author Secondary Information:	
Corresponding Author's Institution:	McGill University
Corresponding Author's Secondary Institution:	
First Author:	Anwesha Bhattacharya
First Author Secondary Information:	
Order of Authors:	Anwesha Bhattacharya
	Edward A. Fon
	Alain Dagher
	Yasser Iturria-Medina
	Jo Anne Stratton
	Chloe Savignac
	Jack Stanley
	Liam Hodgson
	Badr Ait Hammou

Powered by Editorial Manager® and ProduXion Manager® from Aries Systems Corporation

	David A Bennett
	Danilo Bzdok
**Order of Authors Secondary Information:**	
**Response to Reviewers:**	The responses to the specific reviewer comments are attached to the manuscript submission package.
**Additional Information:**	
**Question**	**Response**
Are you submitting this manuscript to a special series or article collection?	No
**Experimental design and statistics**<br><br>Full details of the experimental design and statistical methods used should be given in the Methods section, as detailed in our Minimum Standards Reporting Checklist. Information essential to interpreting the data presented should be made available in the figure legends.<br><br>Have you included all the information requested in your manuscript?	Yes
**Resources**<br><br>A description of all resources used, including antibodies, cell lines, animals and software tools, with enough information to allow them to be uniquely identified, should be included in the Methods section. Authors are strongly encouraged to cite Research Resource Identifiers (RRIDs) for antibodies, model organisms and tools, where possible.<br><br>Have you included the information requested as detailed in our Minimum Standards Reporting Checklist?	Yes
**Availability of data and materials**<br><br>All datasets and code on which the conclusions of the paper rely must be either included in your submission or deposited in publicly available repositories	Yes

(where available and ethically appropriate), referencing such data using a unique identifier in the references and in the “Availability of Data and Materials” section of your manuscript.<br><br>Have you have met the above requirement as detailed in our [Minimum Standards Reporting Checklist]?	
GigaScience has policies and guidelines in place for the use of generative AI-writing tools such as ChatGPT. If you have used such writing tools to assist with writing the manuscript this must be declared and cited in the text. Authors should not list AI-writing tools and other AI-assisted technologies as an author or co-author and should acknowledge that they are fully responsible for text generated or refined by AI-writing tools.<p><br><br>A summary of use (particularly in the introduction or among methods) needs to be included at the end of the paper, and the outputs should also be included as a supplementary file hosted in GigaDB or other open repositories. Please read our guidelines for more information. <p><br><br>By submitting to GigaScience, you are aware of the journal's AI-writing tools policy, and if you have declared use of such tools below, you have acknowledged this where appropriate in your manuscript and have made a summary of use and outputs available. <p><br><b>AI-assisted writing tools have been used in the preparation of this manuscript?	No

Powered by Editorial Manager® and ProduXion Manager® from Aries Systems Corporation

Manuscript

Click here to access/download;Manuscript;Article_Gigascience.docx

Cell type transcriptomic modules reveal shared molecular mechanisms in Alzheimer's and Parkinson's disease

Anwesha Bhattacharya[1,2,10], Edward A. Fon[3], Alain Dagher[4,5], Yasser Iturria-Medina[3,5,6], Jo Anne Stratton[3], Chloe Savignac[1,2,10], Jack Stanley[7,2,10], Liam Hodgson[8,2,10], Badr Ait Hammou[1,2,10], David A Bennett[9], Danilo Bzdok[1,2,8,10]*

[1]Department of Biological and Biomedical Engineering, McGill University; Montréal, Canada

[2]Mila - Quebec Artificial Intelligence Institute; Montréal, Canada

[3]Department of Neurology and Neurosurgery, Montreal Neurological Institute (MNI), McGill University; Montréal, Canada.

[4]Department of Psychology, MNI, McGill University; Montreal, Canada.

[5]McConnell Brain Imaging Centre (BIC), MNI; Montreal, Canada.

[6]Ludmer Centre for Neuroinformatics and Mental Health; Montreal, Canada.

[7]Quantitative Life Sciences, McGill University; Montreal, Canada.

[8]School of Computer Science, McGill University; Montreal, Canada.

[9]Rush Alzheimer's Disease Center, Rush University Medical Center; Chicago, USA.

[10]The Neuro, MNI, BIC, McGill University; Montreal, Canada.

*Corresponding author: danilo.bzdok@mcgill.ca

Anwesha Bhattacharya [0009-0005-7786-1751]; Edward A Fon [0000-0002-5520-6239]; Alain Dagher [0000-0002-0945-5779]; Yasser Itturia-Medina [0000-0002-9345-0347]; Jo Anne Stratton [0000-0002-1205-1353]; Chloe Savignac [0000-0002-7730-8324]; Jack Stanley [0000-0001-7415-3139]; Liam Hodgson [0009-0001-8462-9863]; Badr Ait Hammou [0000-0001-5733-0287]; David A Bennett [0000-0003-3689-554X]; Danilo Bzdok [0000-0003-3466-6620].

Abstract

Historically, Alzheimer's disease (AD) and Parkinson's disease (PD) have been investigated as two distinct disorders of the brain. However, a few similarities in neuropathology and clinical symptoms have been documented over the years. Traditional single-gene centric studies, such as differential gene expression analyses, have struggled to unravel the molecular basis for the observed pathological links between AD and PD. To address this, we tailor a latent factor framework to analyze synchronous gene co-expression at sub-cell-type resolution. Utilizing large, single-nucleus transcriptomics datasets in AD (70,634 nuclei) and PD (340,902 nuclei) from postmortem human brains, we systematically extract and juxtapose disease-critical molecular signatures in the brain. Our transcriptomic analysis reveals shared molecular programs between

AD and PD that systematically localize to specific glial and neuronal cell types. In neurons, convergent gene groups in AD and PD relate to cytoskeletal dynamics and mitochondrial stress mechanisms. Similarly, overlapping gene groups in microglia modules implicate T cell activation mechanisms and synapse pruning pathways. In parallel, AD- and PD-associated genes in astrocytes are involved in heavy metal processing; oligodendrocytes highlight convergent dysregulation in myelin synthesis. In addition, our analysis reveals APOE, an AD GWAS gene, has disease predictive roles in PD-associated gene modules. Conversely, SNCA, a PD GWAS gene, emerges within AD associated gene modules. Our multi-module sub-cell-type approach offers unique insights into the molecular basis of shared neuropathology in AD and PD.

Introduction

Alzheimer's disease (AD) and Parkinson's disease (PD) are two of the most prevalent disorders in today's aging societies[1,2]. There has been intensive research with the grand aim of altering and ultimately halting the course of these diseases. Despite educated forecasts predicting significant advances by this decade[3], AD and PD remain challenging to unravel. This difficulty is compounded by a historically entrenched dichotomy that has limited transfer of research insights from one disease to the other. AD and PD are considered distinct entities due to differences in primary brain regions affected[4–7], age of onset, clinical progression, and treatment response. PD is notably responsive to therapeutics that do not affect cognition[8], and AD is without any "hard-currency" therapeutic to date[9].

This dichotomy between AD and PD continues to be reinforced by genomics and polygenic risk studies, which show minimal to no overlap of genes between AD and PD[10,11]. Indeed, aggregating prior findings, a *Neuron* review recently concluded, "There is intriguingly little overlap between the risk genes for AD and PD, providing genetic evidence for different disease onset and progression mechanisms"[12].

By contrast, autopsy examinations show that over half of PD patients have aggregates of tau[13] and around 30% of PD patients develop cognitive impairment with many going on to dementia[14]. Conversely, AD pathology and Lewy bodies co-occur more frequently than by chance, with Lewy bodies associated with cognitive decline[7,15,16]. Further, the substantia nigra in AD can harbor tangles which are symptomatic of parkinsonism[17]. These observations raise the possibility of disease mechanisms contributing to shared neuropathology between AD and PD at the molecular level[18,19].

More broadly, recent systems-level analysis further suggests that multiple neurodegenerative disorders, including AD and PD, share overlapping biological pathways and pathological mechanisms[18]. Proposed mechanisms include mitochondrial dysfunction, neuroinflammation, and dysregulated protein homeostasis[20]. Involvement of these processes have also been suggested in several other neurodegenerative disorders, reinforcing the view that neurodegenerative mechanisms may arise from partially overlapping manifestations of shared molecular networks rather than completely independent disease mechanisms [19,21].

Despite this evidence, most genomics studies directly questioning the genetic and molecular basis of AD and PD overlap have not identified clear molecular mechanisms underlying

this relationship. Methodologically, these studies have largely relied on univariate approaches that focus on individual genes[22–25]. In reality, however, gene expression occurs within tightly regulated environments where gene products interact in highly combinatorial ways[26–29]. Thus, the pathogenesis of neurodegenerative diseases is likely driven by molecular dysregulation within gene networks rather than by isolated gene anomalies[30–32].

Moreover, the effects of dysregulated genes are not identical across different cell types. Large-scale single-nucleus transcriptomic studies, individually in AD[33,34] and PD[4,35], have reported disease-associated transcriptional changes that are highly cell type-specific. For example, in AD, APOE expression is increased in microglia and decreased in astrocytes and oligodendrocyte precursor cells[36,37]. Given such complexity at the cellular level, it is crucial to account for cell type heterogeneity when comparing AD and PD related changes. This is evidenced by the minimal AD-PD overlap derived from bulk RNA sequencing-based analysis [38]. Recent advances in single-nucleus RNA sequencing (snRNA-seq) have enabled the resolution of transcriptional changes with high cell-type specificity. However, in contrast to recent proteomics- and CSF-based AD-PD comparative analysis [39], no single study has employed multivariate approaches at the single-cell resolution to investigate AD and PD simultaneously at scale.

In the present investigation, we systematically revisited the problem of identifying candidate molecular mechanisms that overlap between AD and PD, through the lens of transcriptomics. Comprehensive snRNA-seq datasets from AD (70,634 nuclei from 48 postmortem brains)[36] and PD (340,902 nuclei from 15 postmortem brains)[40] allowed us to leverage advanced machine learning techniques[41] to quantitatively characterize and compare key disease-associated gene expression signatures. Enabled by a supervised multivariate model[42], we extracted and compared several biologically interpretable gene modules at sub-cell type granularity from the AD and PD transcriptomes (16,936 protein-coding transcripts). By linking our gene modules to curated biological pathways, we identified shared candidate mechanisms of neurodegeneration. In addition, we validated our main findings and conclusions using an independent AD and PD dataset pair. Overall, our supervised pattern learning based comparative approach provides a statistically rigorous and unified framework for the automatic discovery and comparison of disease associated molecular signatures across disorders.

Results

Rationale

Traditional approaches in single-cell transcriptomics, such as DGE, typically focus on individual genes in isolation. This offers a fragmented view of disease-related transcriptional changes. Recent studies employing univariate frameworks to identify disease-associated genes have reported modest overlap in AD- and PD-relevant genes, primarily limited to glial populations [43]. However, we hypothesized that such methods may fail to capture shared molecular co-regulatory programs that might be altered in both AD and PD.

To address these limitations, here we interrogated AD- and PD-transcriptional changes using gene modules, representing coordinated gene activity across the transcriptome. In addition, we reasoned that disease progression within each cell type was not driven by a single molecular

axis. Rather, multiple distinct transcriptional programs were altered. Therefore, a biologically coherent AD and PD comparison would entail examining multiple distinct cell type-specific gene programs to capture the full complexity of their molecular convergence.

To extract disease relevant gene modules, we deployed a supervised latent factor modeling framework, previously validated in AD [42]. This method combined latent structure discovery strength while simultaneously being aware of valuable contextual information — the disease state. We extended the gene-module discovery framework to a rigorous cross-disorder comparative setting comparing AD- and PD-associated genes.

Moreover, by examining the entire recorded transcriptome, our data-driven pipeline enabled an unbiased assessment of genes, without prior assumptions about AD- or PD-association. In addition, our approach could assign a single gene as being relevant to multiple gene modules from a single cell type — a feature absent from many previous gene network analysis techniques [31]. Overall, our unbiased, multivariate analysis of the transcriptomic landscape of AD and PD revealed valuable insights about the molecular mechanisms potentially shared between these two neurodegenerative disorders.

Disease predictive, cell type-specific gene modules identified using latent factor modeling.

We explored the possibility of molecular overlap in AD and PD brains through transcriptional alterations captured in gene modules (groups of co-expressed genes). The main analyses were conducted on two snRNA-seq datasets — Rosmap-AD [36] (70,634 nuclei across 8 major cell types; Methods), and Kamath-PD [40] (340,902 nuclei across 11 cell types). Our analytical framework employed partial least squares discriminant analysis (PLS-DA) to gain an overview of 16,936 protein-coding transcripts (see Methods).

In either AD or PD, we fitted cell type level PLS_{cell} models across nuclei from all donors in the dataset (71; Methods). This yielded gene modules as latent projections of gene expression matrices that assisted in distinguishing cells of patients from controls. Comparative assessments of these thus-derived gene modules highlighted shared genetic mechanisms between AD and PD that were more stable than expected by chance (Fig. 2A).

As a first step, we explored the transcriptomes of AD and PD to identify disease- and cell type-specific modeling hyperparameters. Specifically, the optimal number of gene modules that best distinguished diseased from healthy cells (in unseen cells) was selected using a ten-fold nested cross-validation scheme (see Methods). In Rosmap-AD, two gene modules per cell type were determined to be optimal for all six cell categories. In Kamath-PD, the optimal number of gene modules was determined to be two for each major cell type, except for endothelial cells (three modules) and CALB1 dopaminergic neurons (three modules).

The fitted cell type specific PLS_{cell} models (6 AD and 9 PD models; optimal hyperparameters), exhibited robust above-chance out-of-sample accuracy in differentiating disease samples from control (Fig. S1A). Unbiased classification accuracy was estimated based on a patient-partitioned cross-validation scheme (see Methods). Specifically, in the AD vs control

group contrast, the predictive power measured by AUROC ranged highest for microglia (AUROC: 0.66 ± 0.06 std across partitions) to lowest for oligodendrocyte precursor cells (OPCs) (0.56 ± 0.08 std across partitions). For the PD vs control group contrast, the highest AUROC was for endothelial cells (0.89 ± 0.13 std across partitions), and the lowest was for excitatory neurons (0.69 ± 0.35 std across partitions). This strongly supported the role of gene modules in directly informing the disease phenotype across all examined cell types and conditions.

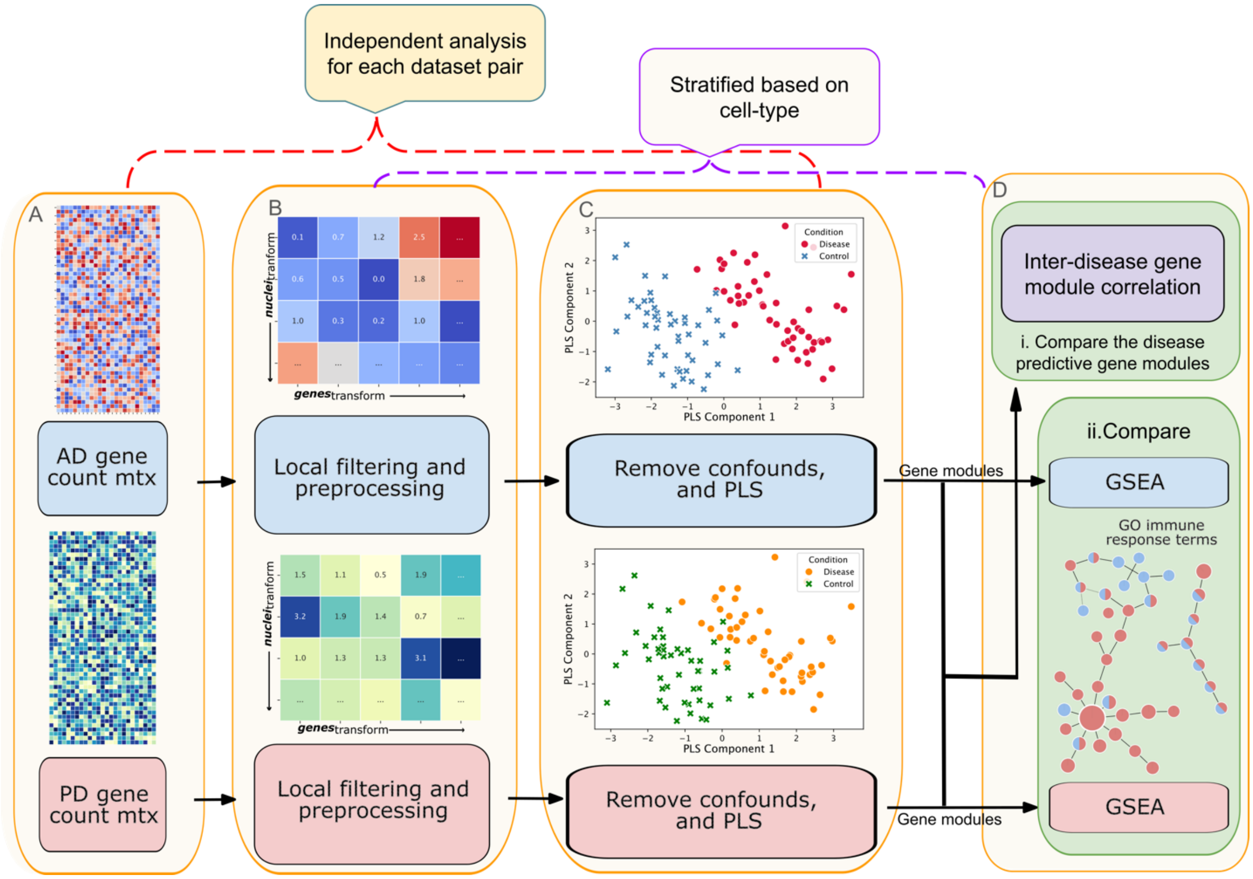

Figure 1. Workflow diagram to test for AD-PD overlap: bottom-up approach. Overview of workflow. (A) Single nucleus RNA sequencing (snRNA-seq) datasets for AD and PD were downloaded from public databases. Filtering, quality control, and cell type annotations were performed by source authors. (B) Preprocessing step. Performed independently for each cell type, this step followed recommended guidelines for data transformation, removed lowly expressed genes, and corrected for disease vs control class imbalance. (C) Gene module identification step. PLS discriminant analysis was performed per cell type to extract weighted gene lists, referred to as gene modules, that were disease predictive. (D) Comparative analysis. We aggregated the results from the two analysis arms and assessed overlap using parallel methods – i. direct correlation of cross-disease gene module pairs and ii. gene set enrichment analysis to identify overlapping biological processes, molecular functions, and cellular components. We discover significant molecular similarities between AD and PD across cell types. PLS, partial least squares; AD, Alzheimer's disease; PD, Parkinson's disease; GSEA, gene set enrichment analysis; GO, gene ontology.

Our thus derived gene modules (PLS_{cell} components) featured a combination of several genes whose co-expression signature was associated with a disease state (AD vs. control or PD vs. control). We assessed the statistical significance of each module by comparing its empirical disease prediction performance to a null distribution of performance metrics derived by a label-shuffling permutation procedure (Methods). Only significant gene modules were considered for subsequent analyses (empirical module $\rho > 97.5^{th}$ percentile of permutation derived ρ distribution; Fig. S1B). In particular, two cell types in Rosmap-AD, pericytes and ependymal cells, did not pass our significance test and were removed from further analysis. Two cell types in Kamath-PD, macrophages and ependymal cells, were similarly removed.

We identified stable genes within each PLS_{cell} gene module (12 AD modules, 20 PD modules). Specifically, using a bootstrap (BS) resampling technique (see Methods), we assessed which gene effects were statistically robust (zero not included in the 2.5/97.5% confidence interval (CI) of the BS distribution of each gene), and thus, reliably affected prediction outcomes. As an illustration, we visualized the PLS_{Mic} loadings for the first gene module from Rosmap-AD microglial cells (Fig. 2B). Overall, each gene module yielded a variable set of robust genes distributed across the transcriptome (Fig. 2C).

We further investigated the characteristics of the derived modules using a clustering algorithm. For a cell type, we assigned each observed nucleus to exactly one of its modules based on the component harboring the maximum PLS_{cell} score. In doing so, we were able to visualize the distribution of the gene modules assigned to the nuclei in a two-dimensional embedding space (Fig. S2; PHATE embedding space; Methods). No clear clustering among the components was observed suggesting that our gene modules did not necessarily correspond to cellular subtypes. Instead, they likely corresponded more closely with different functional programs within a given major cell type. That is, any given cell belonging to a type could exhibit several of our distinct gene programs to various continuous degrees.

In a stringent external validation analysis using untouched datasets, we derived independent AD and PD gene modules in two additional snRNA-seq datasets — Seattle-AD and Smajić-PD (detailed cohort and sample description in Methods). We repeated all main analyses, from scratch, and derived sub-cell-level disease predictive gene modules from these datasets (Fig. S3B; Methods). In Seattle-AD, 15 significant gene modules emerged across ten examined cell types. Independently, in Smajić-PD, 25 significant gene modules emerged across seven cell types. Significance of modules was determined, as before, using a label-shuffle permutation test. These independently derived gene modules were subsequently used to corroborate our findings from the primary AD-PD overlap analysis (see next section).

We assessed whether the latent gene modules were influenced by potential demographic variables. Concretely, we examined the contribution of age and sex to variation in module scores, quantified by the proportion of variance explained using a linear regression model (Methods). Across gene modules, the contribution of sex towards explaining the variance in module scores was low within each dataset (mean ± standard deviation across modules): Rosmap-AD, $R^2 = 0.01 \pm 0.01$; Kamath-PD, $R^2 = 0.07 \pm 0.09$; Seattle-AD, $R^2 = 0.017 \pm 0.018$; Smajić-PD, $R^2 = 0.12 \pm 0.10$. Similarly, age explained little variance in module scores: Romsap-AD, $R^2 = 0.003 \pm 0.006$; Kamath-PD, $R^2 = 0.04 \pm 0.08$; Seattle-AD, $R^2 = 0.018 \pm 0.020$; Smajić-PD, $R^2 =$

0.04±0.06. Overall, across all gene modules from the four datasets, age and sex explained only a small fraction of the variance in module scores relative to the variance captured by diagnosis, the primary variable of interest (table S10).

Importantly, in all analyses so far, the gene expression samples from AD and PD were not merged. Instead, these were conducted in parallel with independent supervision targets (AD-control or PD-control). Thus, we identified robust AD- and PD-predictive gene modules in a cell type-specific manner. These thus derived modules, specific to AD or PD, enabled subsequent comparative analyses aimed at examining the shared molecular alterations between AD and PD.

Shared molecular signatures uncovered between AD and PD, across major brain cell types.

We subsequently moved to our comparative analysis between AD and PD. To quantify the coupling between AD- and PD-derived gene modules, we used Kendall's tau-b (τ_b) metric (Fig. 2D; Methods). This calculated the degree of similarity between two vectors of PLS_{cell} predictive weights of the overlapping robust genes (not the gene expression measurements) from an AD-PD gene module pair (Fig. 2D). By comparing the empirical correlation strength with a permutation-derived null distribution (obtained by correlating permutation modules derived from a label-shuffled dataset), we identified the module pairs with significant associations (empirical τ_b more extreme than 2.5/97.5% CI of the null distribution). Notably, we reported only those associations that survived this exhaustive permutation-based validation.

As the most important results of our investigation so far, we noted strong and robust associations across several AD-PD module pairs (robust to 1000 iterations label shuffle permutation test; τ_b FDR q-value < 0.05; Fig. 2A; table S1; Methods). Across all pairwise combinations, the most significant correlation (smallest q-value) emerged between the first oligodendrocyte module (represented as Oli 1) from Rosmap-AD and the second oligodendrocyte module (Oli 2) from Kamath-PD (represented as Oli 1_Oli 2; $\tau_{b, abs}$= 0.44, number of shared genes = 1351, q = 5.4e-128). In addition to Oli 1_Oli 2, other top significant pairs included Oli 1_Ex 1, Oli 1_Ex 2, In 2_Ex 1 (exhibited $\tau_{b, abs}$ > 0.3, and shared over 500 robust genes).

In contrast, the strongest associations (ranked by absolute τ_b) were observed between glial modules — astrocytes, microglia, and OPCs. Specifically, the highest association strength was observed between Mic 2_Mic 1 ($\tau_{b, abs}$= 0.67; q = 0.009), followed by Ast 2_Ast 2 ($\tau_{b, abs}$ = 0.62; q = 4.2e-5). This suggests that in glial cells, disease associated molecular programs are broadly shared across AD and PD.

On the flipside, inter-neuron module pairs (Ex and In pairs) between AD and PD showed the least overlap, across all module pairs. This suggests potentially different molecular mechanisms in AD and PD within these major neuronal populations. However, dopaminergic neurons in PD — CALB1, and SOX6 — showed significant similarities with AD-critical gene programs in neurons (Ex 1_Calb1 1, $\tau_{b, abs}$ = 0.35, q = 3.1e-20; In 1_Calb1 1, $\tau_{b, abs}$ = 0.32, q = 1e-5), along with AD associated oligodendrocyte and astrocyte modules (Oli 1_Calb1 3, $\tau_{b, abs}$ = 0.19, q = 8.21e-5). Thus, in contrast to the major recorded PD neuronal populations (Ex and In), disease-

critical PD-neurons (highlighted in original study [40]) had significant molecular similarities with several AD-associated molecular programs.

As a sanity check, we assessed how the cross-cell type module associations compared within a single disease. Specifically, we looked at the correlation effect sizes (statistically significant under a label shuffled permutation test, $q < 0.05$; Methods) across gene modules in a Rosmap-AD versus Rosmap-AD comparison, and a Kamath-PD versus Kamath-PD comparison (Fig. S1D). The presence of robust association signatures between gene modules from different

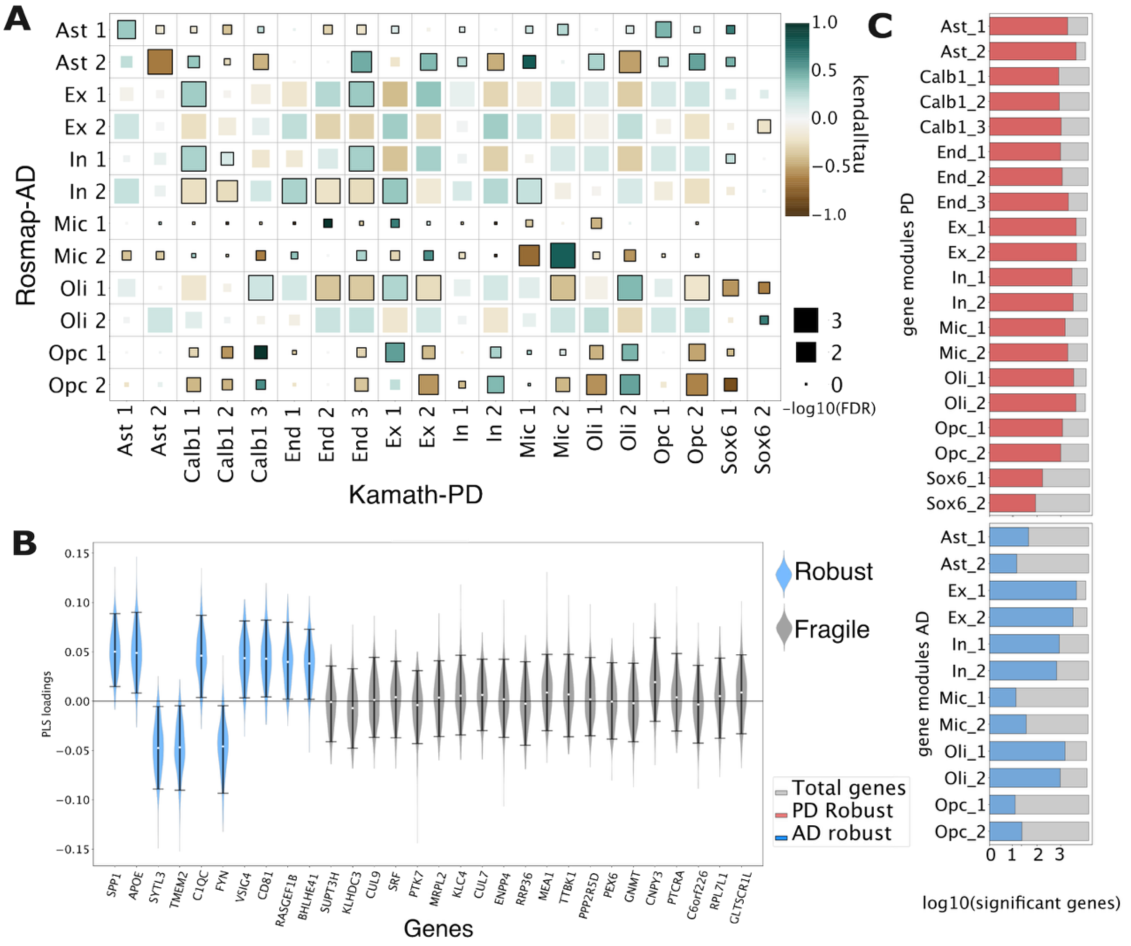

Figure 2. Convergence of transcriptomic signatures in Alzheimer's and Parkinson's disease across cell types when zoomed in on disease-predictive gene modules. We probed two snRNA-seq datasets (Rosmap-AD, Kamath-PD) to explore the transcriptomic overlap between AD and PD. By training 15 PLS models (one for each cell type in AD (6) or PD (9)), we extracted latent representations of gene expression (gene modules) that maximized the separation between disease and control nuclei. (A) The colored squares represent Kendall's tau-b (τ_b), quantifying the degree of association between AD-PD gene modules. Darker colors represent stronger genetic associations, indicating similar ranking trends of robust genes in the module pair. Black boxes denote statistically significant τ_b exceeding 2.5/97.5% CIs based on label-shuffled permutation tests (n = 1000). Square size is proportional to FDR-corrected statistical significance of overlap. The wide range of correlation strengths suggests that AD and PD share significant molecular similarity spanning the cell type landscape. (B) An illustration of the robustness assessment of genes in a gene module. The 30 genes with the highest empirical predictive weights from the first PLS component in Rosmap-AD microglia are shown. Violins represent the loading distribution of genes derived from a bootstrap resampling scheme (n=500). Robust genes (blue) have nonzero predictive weights (2.5/97.5% CIs). Genes that do not meet this criterion are greyed out. APOE, a gene strongly associated with AD, was highlighted as a major disease predictor in this component. (C) The colored portions of the bars represent the number of genes with robust disease-predictive weights for each gene module. The grey regions denotes the total number of analyzed genes for the cell type. Robust genes were assessed based on a 500-iteration bootstrap resampling scheme. Ast, Astrocyte; Ex, Excitatory neuron; In, Inhibitory neuron; Mic, Microglia; Oli, Oligodendrocyte; Opc, Oligodendrocyte precursor cell; PLS, Partial least squares.

cell types within the same disease provided additional confirmation of the cross-cell type module-

level overlaps observed between AD and PD.

We validated our primary findings by examining molecular similarity between Seattle-AD and Smajić-PD derived gene modules. Our analysis revealed significant module-level overlaps between AD and PD, scattered across different cell types (Fig. S3A; table S2). Across all modules, inter- and intra-oligodendrocyte module pairs from AD and PD took center stage, aligning with our primary analysis. The strongest association was observed between oligodendrocytes from Seattle-AD and Smajić-PD (Oli 1_Oli 1, $\tau_{b, abs}$= 0.51, q = 5.5e-26), followed closely by Ast 1_Oli 1 ($\tau_{b, abs}$= 0.26, q = 9.2e-16). Strong significant associations were also observed between different combinations of neuron and glial cell-derived modules (Ast 1_L4_it 1, $\tau_{b, abs}$= 0.45, q = 1.6e-9; Oli 2_In 3, $\tau_{b, abs}$= 0.86; q = 0.05). Further, inhibitory neuron-derived modules showed sparse similarities between AD and PD, similar to our observations in the primary analysis. Overall, the external validation of shared genetic signatures between AD and PD replicated our primary findings indicating that these are unlikely to be driven by dataset-specific factors such as transcriptomic platform, cohort composition, or brain region selection.

We further assessed the generalizability of our comparison model by randomly partitioning the original AD and PD datasets into two non-overlapping subset pairs (split-half test; see Methods). We then repeated the full analysis pipeline on each subset and compared the resulting pairwise associations between the subset-derived gene modules. Our analysis revealed strong associations across different realizations of the partitioned, but otherwise identical, analyses (Pearson's rho = 0.92 ± 0.02 standard deviation; Fig. S1C). These results provided additional quantitative support for the associations from the full datasets, indicating that the observed relationships were robust to sample size.

We assessed the specificity of our findings from the AD-PD comparative analysis with respect to other systemic diseases. Specifically, we performed a comparison of AD or PD derived modules with chronic obstructive pulmonary disease (COPD) of the lung (neurological versus non-neurological). COPD was selected as a negative control condition given the expectedly different cellular composition and tissue context relative to the brain. Following our established analytical pipeline, we computed pairwise similarities between AD- and PD-associated gene programs with COPD-associated gene programs across major recorded lung cell populations (AD-Lung, PD-Lung; derived following Fig.1 pipeline; table S5-S8).

Overall, we observed limited overlap between brain and lung disease modules. The modest similarities that were detected primarily involved immune related cell populations and endothelial cells from the lung and the brain. Specifically, neuronal populations from all 4 AD and PD datasets showed little overlap with the lung gene modules (maximum Mye 1_Ex 1 (Lung_Kamath-PD), $\tau_{b, abs}$ = 0.49, FDR q = 1.43e-181). In contrast to the neuronal cells from the brain, lung myeloid (Mye) and lymphoid (Lymp) cells showing significant overlap with brain glial cells (maximum Lymp 1_Mic 1 (Lung_Rosmap-AD), $\tau_{b, abs}$= 0.74, FDR q = 6.4e-4). Taken together, these results suggest that neuronal gene programs identified in AD and PD are largely brain-specific, while a subset of immune-related signatures are shared across tissues and disorders.

Collectively, these findings revealed significant molecular overlap between AD and PD at a sub-cell type resolution. The degree and specificity of these overlaps varied between cell type-

specific gene module pairs, with oligodendrocyte and neuron-based gene module combinations in AD and PD signaling the strongest similarities. Our external validation experiments replicated these core findings in independent datasets, further substantiating our conclusions regarding the shared genetic architecture between these neurodegenerative diseases.

Cell type-specific gene modules reveal GWAS derived genes as key predictors of disease

We contextualized our gene modules post-hoc to understand their relationship with known risk gene from genomic studies. Drawing from the most recent GWAS that reported AD[44] or PD[45], we investigated 164 genes (table S3; see Methods), locating them within our gene modules. Most GWAS genes showed robust disease-predictive loadings in at least one gene module (Fig. 3). The top gene module harboring the most GWAS genes in AD was Ex 1 with 25.6% of AD GWAS genes present, followed by Ast 1 with 24.3% GWAS genes present. In PD, the top modules were Oli 1 with 58.9% PD GWAS genes followed by Ex 2 with 42%. Moreover, we found clear cell type localization of these genes within this modular framework. For example, APOE, a broadly accepted AD risk gene, showed robust predictive loadings in the Ast 1 and Mic 1 derived from AD. Similarly, LRRK2, one of the major PD risk genes, was implicated in distinct PD-related gene modules. The strongest effect was observed in PD Mic 2.

Next, we analyzed the effects of GWAS genes from one disease within gene modules linked to the other neurodegenerative disease. In other words, we investigated whether genes mapped to AD GWAS risk loci were highlighted in any PD modules and vice versa. Among the AD GWAS genes, top genes, including APOE, APP, BIN1, and CLU (based on p-value from GWAS [44]) had robust disease predictive loadings in gene modules associated with PD. Concretely, APOE had strong predictive weight in PD Mic 2 (gene loading = -0.06, max absolute loading for any gene in this module was 0.07, absolute rank of this gene = 23), APP in PD Mic 1 (loading = 0.04, max_{abs} = 0.07, rank = 262), BIN1 was found in PD Calb1 2 (loading = -0.02, max_{abs} = 0.04, rank = 1626), and CLU had the strongest robust weight in PD Oli 2 (loading = 0.03, max_{abs} = 0.08, rank = 405). It is important to note that these genes were not the highest-ranked in the PD modules. In other words, they were not the primary disease-indicative genes (rank = 1). Instead, these genes likely played supporting roles that become apparent only within the context of gene modules.

Conversely, we made similar observations for previously reported PD GWAS genes[46] within our AD modules. SNCA had strong predictive loading in AD Ex 1 (-0.03, max_{abs} = 0.07, rank = 397), MAPT in AD In 1 (-0.02, max_{abs} = 0.07, rank = 979), and TMEM175 in AD Ex 1 (-0.02, max_{abs} = 0.07, rank = 1869). Interestingly, while the genes implicated in AD GWAS had very clear cell type localizations, the risk genes associated with PD tended to be distributed across modules implicating multiple cell types, with particularly high effect sizes in neuronal modules. This observation aligned with a previous genomic enrichment study showing that PD risk loci are not confined to specific cell types. Instead, they are associated with broad cellular processes observable across multiple cell types[47]. Collectively, we observed that the bona fide GWAS genes not only tracked the disease they were implicated in but known GWAS hits also proved relevant in gene modules associated with the other disease.

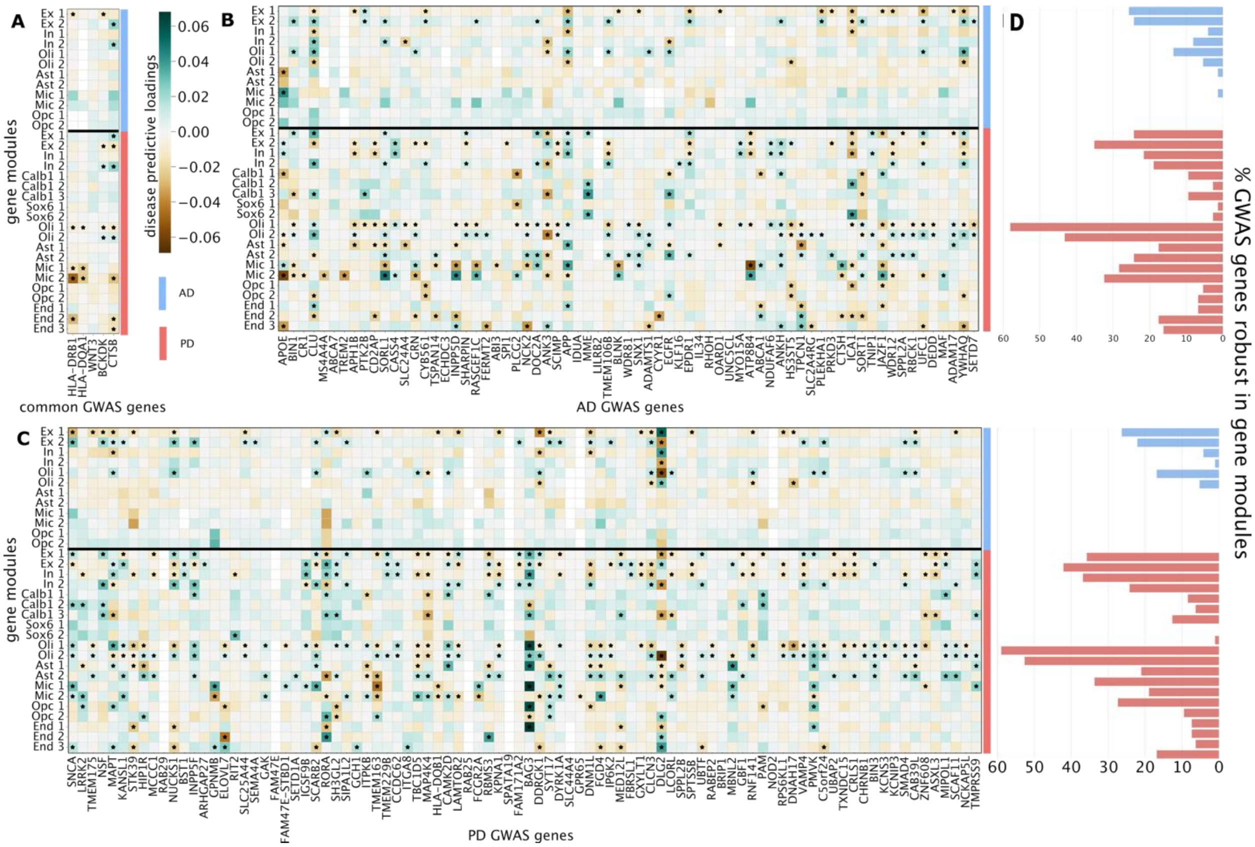

Figure 3. Cell type-specific gene modules reveal GWAS-associated genes as key disease predictors. We mapped the contribution of 164 candidate GWAS-nominated genes, compiled from the largest AD and PD GWAS studies, within our gene modules. Color saturation represents the strength of the predictive weight of a gene in a module as determined by the PLS model. Robust genes are highlighted with black stars, defined as those with bootstrap-derived 2.5/97.5% CIs that do not include zero. **(A-C)** Genes are grouped based on the nominating disease. Group (A) contains 5 common genes nominated independently in both AD and PD. (B) shows the genes implicated in AD GWAS. (C) shows genes implicated in PD GWAS. **(D)** The bars on the right summarize the connection of a module with GWAS mapped genes. The lengths indicate the percentage of GWAS-mapped genes with robust loadings in the module (blue = AD modules, red = PD modules). Cell type-specific module-level localization of risk genes was observed. APOE, a major AD risk gene, showed strong predictive loading in AD microglial and astrocyte modules. For PD, SNCA had strong predictive signals in PD oligodendrocyte, microglia, excitatory neurons, and CALB1 dopaminergic neuron modules. Several genes which were implicated as being AD-relevant in GWAS had robust predictive loadings in PD gene modules and vice versa. For example, APOE, APP, and other AD genes mapped to risk loci had robust predictive weights in PD modules. Likewise, SNCA, MAPT, and other PD risk genes had robust predictive weights in AD modules. Ast, Astrocyte; End, Endothelial; Per, Pericyte; Ex, Excitatory neuron; In, Inhibitory neuron; Mic, Microglia; Oli, Oligodendrocyte; Opc, Oligodendrocyte precursor cell; GWAS, Genome-wide association study.

In our external validation analysis, GWAS-mapped genes exhibited consistently strong disease-predictive loadings across gene modules derived from the Seattle-AD and Smajić-PD datasets (Fig. S4). Notably, all key observations from our primary dataset analysis were replicated.

First, the genes tracked disease-corresponding modules; Microglia and astrocyte AD modules faithfully tracked APOE (interestingly, we observed robust predictive loading for APOE in Seattle-AD OPC modules [36]). Similarly, PD GWAS-mapped genes, such as SNCA and LRRK2, were tracked by PD predictive modules. Second, mirroring our primary dataset pair, several GWAS-mapped genes were enriched in modules associated with the opposite disease (for instance, APOE tracked PD modules and SNCA tracked AD modules), further reinforcing the presence of cross-disease molecular convergence.

In summary, here we contextualized the suspected genes from AD- or PD-GWAS risk loci within the scope of gene modules. This led us to identify significant effects of GWAS genes beyond the disease in which they were initially reported. For example, we observed significant impact of APOE not only in AD (microglia and astrocyte-specific modules), but also in PD-derived modules (microglia, astrocytes, and CALB1 DA neurons). These cross-disease results were replicated in our independent validation datasets, reinforcing the primary observations. Crucially, these observations were only possible through our approach, which evaluates the joint contribution of statistically meaningful gene sets to disease status. Thus, although APOE may not show a strong univariate effect in PD, it plays a significant role when considered within the context of co-expressed genes in PD gene modules.

Overlapping biological functions between AD and PD gene modules

We contextualized the biological relevance of the observed AD-PD overlap using comprehensive gene set enrichment analyses (GSEA). GSEA was performed independently for each gene module (12 Rosmap-AD and 20 Kamath-PD). Notably, in our analysis, a single gene can contribute significantly to a disease via multiple modules within the same cell type. This enabled us to capture likely gene effects on complementary, co-regulated pathways.

By screening the widely relied upon gene ontology (GO) databases corresponding to three complementary domains — biological processes (BP), molecular functions (MF), and cellular components (CC) — we identified AD or PD relevant GO terms. Across all gene modules in AD, 284 BP, 56 MF, and 102 CC terms were identified. In PD, we obtained 715 BP, 152 MF, and 209 CC terms. In total, a universe of 27,993 GO BP, 11,271 GO MF, and 4,039 CC terms were analyzed.

Among all terms across gene modules, 158 BP, 33 MF, and 80 CC terms were shared between AD and PD (table S4). The relatively small subset of overlapping terms highlighted the non-random and specific nature of our gene modules (Fig. 4A). The greatest number of GO term overlaps emerged in neuron-neuron AD-PD module pairs, along with pairs involving oligodendrocytes and oligodendrocyte precursor cells (>160 terms per module pair, BP, MF, and CC combined; Fig. S5A, note Fig. 4B horizontal axis). Additionally, both AD and PD microglial gene modules shared, on average, 20 terms with other gene modules. In contrast, module pairs involving astrocytes featured lower overlaps, with the maximum number of shared terms being 13 between AD Ex 1 and PD Ast 1.

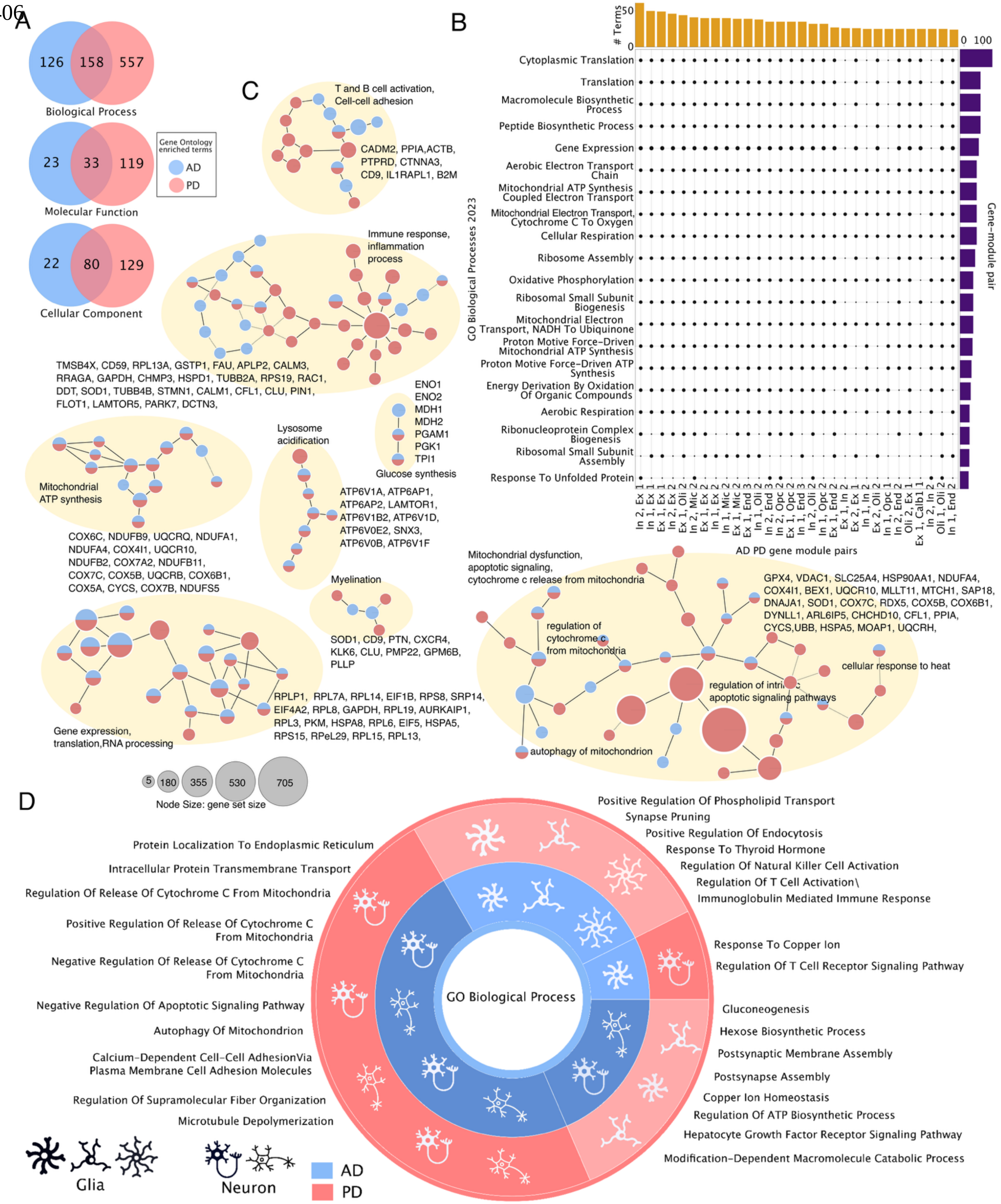
A
126
158
557
Biological Process
23
33
119
Molecular Function
22
80
129
Cellular Component
Gene Ontology enriched terms
AD
PD
B
Terms
50
0
0 100
Cytoplasmic Translation
Translation
Macromolecule Biosynthetic Process
Peptide Biosynthetic Process
Gene Expression
Aerobic Electron Transport Chain
Mitochondrial ATP Synthesis Coupled Electron Transport
Mitochondrial Electron Transport, Cytochrome C To Oxygen
Cellular Respiration
Ribosome Assembly
Oxidative Phosphorylation
Ribosomal Small Subunit Biogenesis
Mitochondrial Electron Transport, NADH To Ubiquinone
Proton Motive Force-Driven Mitochondrial ATP Synthesis
Proton Motive Force-Driven ATP Synthesis
Energy Derivation By Oxidation Of Organic Compounds
Aerobic Respiration
Ribonucleoprotein Complex Biogenesis
Ribosomal Small Subunit Assembly
Response To Unfolded Protein
GO Biological Processes 2023
Gene-module pair
AD PD gene module pairs
C
T and B cell activation, Cell-cell adhesion
CADM2, PPIA,ACTB, PTPRD, CTNNA3, CD9, IL1RAPL1, B2M
Immune response, inflammation process
TMSB4X, CD59, RPL13A, GSTP1, FAU, APLP2, CALM3, RRAGA, GAPDH, CHMP3, HSPD1, TUBB2A, RPS19, RAC1, DDT, SOD1, TUBB4B, STMN1, CALM1, CFL1, CLU, PIN1, FLOT1, LAMTOR5, PARK7, DCTN3,
ENO1
ENO2
MDH1
MDH2
PGAM1
PGK1
TPI1
Glucose synthesis
Mitochondrial ATP synthesis
COX6C, NDUFB9, UQCRQ, NDUFA1, NDUFA4, COX4I1, UQCR10, NDUFB2, COX7A2, NDUFB11, COX7C, COX5B, UQCRB, COX6B1, COX5A, CYCS, COX7B, NDUFS5
Lysosome acidification
ATP6V1A, ATP6AP1, ATP6AP2, LAMTOR1, ATP6V1B2, ATP6V1D, ATP6V0E2, SNX3, ATP6V0B, ATP6V1F
Myelination
SOD1, CD9, PTN, CXCR4, KLK6, CLU, PMP22, GPM6B, PLLP
Gene expression, translation,RNA processing
RPLP1, RPL7A, RPL14, EIF1B, RPS8, SRP14, EIF4A2, RPL8, GAPDH, RPL19, AURKAIP1, RPL3, PKM, HSPA8, RPL6, EIF5, HSPA5, RPS15, RPeL29, RPL15, RPL13,
Mitochondrial dysfunction, apoptotic signaling, cytochrome c release from mitochondria
GPX4, VDAC1, SLC25A4, HSP90AA1, NDUFA4, COX4I1, BEX1, UQCR10, MLLT11, MTCH1, SAP18, DNAJA1, SOD1, COX7C, RDX5, COX5B, COX6B1, DYNLL1, ARL6IP5, CHCHD10, CFL1, PPIA, CYCS,UBB, HSPA5, MOAP1, UQCRH,
regulation of cytochrome c from mitochondria
regulation of intrinsic apoptotic signaling pathways
cellular response to heat
autophagy of mitochondrion
5
180
355
530
705
Node Size: gene set size
D
GO Biological Process
Positive Regulation Of Phospholipid Transport
Synapse Pruning
Positive Regulation Of Endocytosis
Response To Thyroid Hormone
Regulation Of Natural Killer Cell Activation
Regulation Of T Cell Activation\
Immunoglobulin Mediated Immune Response
Response To Copper Ion
Regulation Of T Cell Receptor Signaling Pathway
Gluconeogenesis
Hexose Biosynthetic Process
Postsynaptic Membrane Assembly
Postsynapse Assembly
Copper Ion Homeostasis
Regulation Of ATP Biosynthetic Process
Hepatocyte Growth Factor Receptor Signaling Pathway
Modification-Dependent Macromolecule Catabolic Process
Protein Localization To Endoplasmic Reticulum
Intracellular Protein Transmembrane Transport
Regulation Of Release Of Cytochrome C From Mitochondria
Positive Regulation Of Release Of Cytochrome C From Mitochondria
Negative Regulation Of Release Of Cytochrome C From Mitochondria
Negative Regulation Of Apoptotic Signaling Pathway
Autophagy Of Mitochondrion
Calcium-Dependent Cell-Cell AdhesionVia Plasma Membrane Cell Adhesion Molecules
Regulation Of Supramolecular Fiber Organization
Microtubule Depolymerization
Glia
Neuron
AD
PD

Figure 4. Gene Ontology terms mapped to gene modules are shared between AD and PD. Gene set enrichment analysis results for the derived gene modules. For each gene module in AD or PD, we mapped the ranked genes (based on predictive weights) to terms in the Gene Ontology (GO) database. **(A)** Overlapping terms from GO Biological Process, Cellular Component, Molecular Function. The Venn diagrams depict the number of unique and shared terms across all gene modules, grouped by AD or PD. **(B)** Top 30 most frequently shared GO terms in AD-PD gene-module combinations. Solid black dots indicate that a term (vertical axis) is enriched in the corresponding gene-module pair (horizontal axis). Bar plots on the horizontal axes show counts of the total number of shared terms for the gene-module pair. Bar plots on the vertical axis show the total number of cross-disease gene-module pairs in which a term is present in (top 10 terms). **(C)** Graph visualization of top shared GO biological themes across AD and PD. Nodes are GO terms and colors represent disease label. Node size indicates the GO-term gene-set size. Group names summarize the main themes from the terms in the group. Top AD-PD shared genes (robust PLS loadings) are annotated within each group. This overview highlights key biological processes involved in both AD and PD. **(D)** Shared GO BP terms between AD and PD that are unique to either neurons or glia. The inner circle denotes the cell type group from AD while the outer circle denotes the PD group. Darker shade represent terms enriched exclusively in neuronal modules (excitatory and inhibitory neurons, CALB1, SOX6) and lighter shade represent terms enriched exclusively in glial cell modules (microglia, astrocyte, oligodendrocyte, OPC, endothelial cells). Biological processes related to altered cytoskeleton dynamics, impaired mitochondrial function, and apoptotic signaling are enriched across gene modules from neurons in both AD and PD. Immune response, synapse maintenance, and lipid transport-related terms are enriched in one or more glial cell modules in both AD and PD.

By sorting all biological processes based on their frequency of shared occurrence across AD-PD module pairs, we identified the commonly shared pathways. These were related to protein translation, cellular respiration, and mitochondrial energy synthesis (Fig. 4C). To further summarize the terms systematically, we devised a visualization procedure to obtain a synoptic summary of the overarching biological processes (Methods). Key shared biological themes emerged between AD and PD (Fig. 4D): protein synthesis and misfolding (top shared genes included RPLP1, RPL7A, RPL14, EIF1B, RPS8, SRP14), immune response (TMSB4X, CD59, RPL13A, GSTP1, FAU, APLP2, CALM3, RRAGA, GAPDH, TUBB2A/4B, RPS19, RAC1, DDT, CALM1, CFL1, CLU), lysosome acidification (ATP6V/6A, LAMTOR1, SNX3), glucose metabolism (ENO1, ENO2, MDH1, MDH2, PGAM1, PGK1, TPI1), mitochondrial dysfunction (COX6C/4I1/7A2/7C/5B, NDUFB9/A1/A4/B2/B11/S5, UQCRQ/10/B, CYCS), and myelination (SOD1, CD9, PTN, CXCR4, KLK6, CLU, PMP22, GPM6B, PLLP).

To investigate cell type localized biological processes, we designed a probe to filter out potential cellular injury-related pathways. We first categorized the gene modules into two groups – neuronal modules and glial modules. Given the fundamental anatomical and functional differences between neurons and glia, these groups were expected to exhibit distinct responses to disease. The neuronal group included gene modules from excitatory neurons, inhibitory neurons, CALB1, and SOX6 cell types. The glial group comprised astrocytes, microglia, oligodendrocytes, and OPCs. We then removed all GO BP terms that were enriched in both neuronal and glial modules. This resulted in a refined set of GO terms that were exclusive to either neurons or glia (Fig. 4E; cf. Fig. S5B for gene module level grouping). By comparing these terms between AD and PD, we identified neuron and glia specific shared mechanisms of overlap.

Neurons shared the greatest number of exclusive (unique to cell types belonging to this category) GO BP terms between AD and PD. We identified several shared terms associated with microtubule depolymerization and cytoskeleton dynamics between Kamath-PD and Rosmap-AD neuron modules (PD Calb1 1, Ex 1, Ex 2 and AD In 1, In 2 and Ex 1). Shared genes associated with these terms included MAPT, FKBP4, GBA2, MAP1A, MAP1B, MAP1S, MAP2, MAPRE3, STMN1, STMN2, STMN3, STMN4. We also observed terms related to mitochondrial release of cytochrome c regulation (PINK1, PRELID1, CLU, BNIP3, DNM1L, GHITM, GPX1, MFF, MLLT11, MOAP1). Specifically, Ex 1 and Ex 2 in Kamath-PD, and In 1 and In 2 in Rosmap-AD highlighted these terms. Terms related to iron homeostasis were noted in Ex 1 from PD, and In 1 and Ex 1 from AD (SOD1, several ATP genes, CCDC115, FTH1, FTL, ISCU, NDFIP1, SLC22A17).

We observed that glia-exclusive terms had several themes centered around the immune and complement systems along with synapse pruning, lipid transport, metal ion homeostasis, and thyroid hormone balance. T cell activation pathways were enriched in PD Mic 2, End 2, and AD Mic 2. These modules shared genes including B2M, HLA-A, HLA-B, HLA-C, HLA-DPA1, HLA-DRA, HLA-DRB1, HLA-DRB5, and HLA-E. Lipid and phospholipid transport showed up exclusively in microglial modules, in AD Mic 1 and PD Mic 2 (APOE, TSPO, and PRELID1). Response to thyroid hormone was recorded in PD Mic 2 and AD Mic 1 (CTSB, CTSH).

We also noted a few terms exclusive to opposite categories in AD and PD. For example, "response to copper ion" was identified exclusively in AD glia modules and PD neuron modules. However, functionally related terms, like cellular response to copper ion, copper ion binding, and copper ion homeostasis, were enriched in AD Ast 1, Opc 1, Ex 1, and Ex 2 and in PD Mic 1, End 1, Ex 1, Ex 2, and In 1. A closer inspection of all terms belonging to cross-category modules, AD neuron-PD glia or AD glia-PD neuron suggested that these differences largely reflect the granularity and naming conventions of GO annotations. In contrast, for AD neuron-PD neuron and AD glia-PD glia specific terms were functionally distinct, reflecting meaningful biological differences rather than annotation-related effects. For instance, a manual search for "cytoskeleton" or "microtubule" highlighted only neuronal modules in both AD and PD. These neuron and glia specificity were further supported by replication in independent datasets (Seattle-AD and Smajić-PD).

For validation of these results, we turned to our external dataset pair. Analogous to our first analysis pair, we applied the GSEA pipeline to the gene modules derived from the Seattle-AD and Smajić-PD datasets (Fig. S6A). We confirmed that AD and PD neurons, oligodendrocytes, and OPC modules had the largest occurrence of shared terms (Fig. S6C; table S9). Moreover, these results aligned with our primary analysis derived broad biological themes (Fig. S6B)— including AD and PD shared terms related to mitochondrial energy metabolism (CD44, HSPA1A, CLU), myelination (SOD1, TENM4, PLP1), and glucose metabolism (APOD, RORA, HSPA5).

Thus, our gene set enrichment analysis successfully identified a variety of matching biological, cellular, and molecular processes across AD and PD. Our observations highlighted key themes which localized to certain cell types in AD and PD. Neurons in AD and PD demonstrated enrichment of terms related to cytoskeleton structural integrity, mitochondrial transport, and mitochondrial energy synthesis. Alternatively, glia-derived gene modules highlighted terms

related to several regulatory mechanisms including synapse pruning, lipid transport, immune, and inflammatory systems.

Latent gene modules reveal higher cross disease transcriptomic convergence than traditional differential gene expression

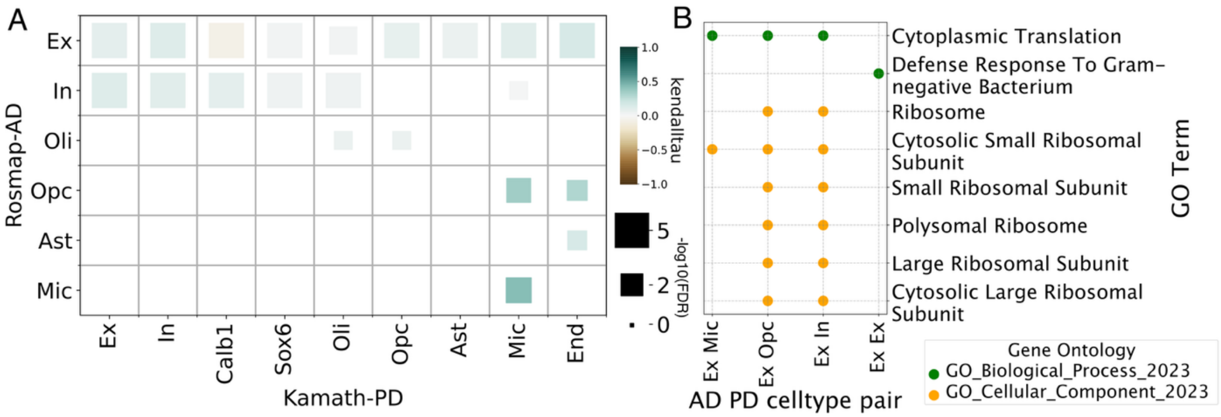

Figure 5. Differential expression analysis revealed modest similarity between AD- and PD-associated transcriptomic signatures. We benchmarked our latent factor model-derived AD-PD overlap with differential gene expression derived AD-PD overlap. (A) Pairwise associations between AD and PD differentially expressed genes are shown (Kendall's tau-b). For each cell type pair, statistical significance of association was assessed using a permutation test. Colored squares indicate significant associations (FDR < 0.05). Darker green (brown) denotes greater similarity (anti) between the log-fold change of significant DEGs from an AD-PD cell type pair. Square size is proportional to $-\log_{10}$(FDR). Compared to our PLS gene-module-based overlap analysis (Fig. 2A), significantly smaller correlations emerge from this univariate approach. The maximum correlation observed was 0.4 between AD and PD microglia. **(B)** Dots represent shared GO terms between AD and PD from gene set enrichment of DGEs. Significant terms in AD or PD from GSEA were assessed (FDR q<0.1). In contrast to 213 shared terms across pairwise AD-PD gene modules (Fig. 4A), only 7 shared terms emerged between AD and PD from our DGE analysis. Ast, astrocyte; Ex, excitatory neuron; In, inhibitory neuron; Mic, microglia; Oli, oligodendrocyte; Opc, oligodendrocyte precursor cell; End, endothelial.

We compared our gene module-based comparative framework to the widely adopted univariate method in RNA-seq — differential gene expression analysis. This method quantifies differences in gene expression between two groups by comparing expression profiles in diseased versus neurotypical cell states. We conducted a parallel AD-PD overlap analysis based differentially expressed genes (DGEs; MAST; Methods) derived independently in the Rosmap-AD (adDGEs) and Kamath-PD (pdDGEs) datasets. Within each dataset, DGEs were computed separately within each cell type (analogous to our main analysis; Methods). Statistically significant DEGs (FDR corrected p-value < 0.05; Methods) were subject to further comparison between AD and PD cell type pairs.

We examined the overlap between the adDEGs and pdDEGs across AD and PD cell types (Kendall's tau-b associations; Fig. 5A; Methods). Across 54 pairwise comparisons (6 AD and 9 PD cell types), the highest significant association observed was 0.4, occurring between microglia adDEGs and pdDEGs (FDR q-value<0.05; p-values corrected for multiple comparisons). Notably, this highest association among all possible cell type pairings, was significantly lower than the maximum similarity observed from our gene module analysis (cf. Fig. 2A).

We then systematically tested the overall difference in mean correlations between AD-PD associations based on pairwise gene module τ_b from PLS (12 x 20) versus pairwise cell type τ_b from DGE (6 x 9). Using Welch's t-test, which accounts for unequal sample sizes, we observed a significant difference in correlation strengths between the two methods. Across pairwise comparisons, PLS_{cell} τ_b values were higher than DGE derived τ_b values (Welch's t = 5.51; p-value<0.001). This finding suggested that associations between AD-PD similarity were systematically stronger when using our gene module approach compared to the classical DGE method.

Next, we performed a gene set enrichment analysis of the differentially expressed genes using GO databases (GO BP, MF, and CC). Independently for each disease, we created our ranked gene list based on the fold change significance level (conditioned on cell type; Methods) and used GSEA to identify significantly enriched terms (FDR q<0.1). We observed 7 common terms, in total, between AD and PD across all 3 GO databases (Fig. 2B). Overall, these terms represented only a small subset of the broader set of shared AD-PD terms identified through our gene-module based analyses (213 GO terms). Notably, the shared terms emerging from DGE analysis (e.g., cytoplasmic translation) appeared among the most frequently recurring terms across gene modules from the PLS analysis (cf. Fig. 4C), suggesting that DGE may primarily capture the strongest disease overlaps from the gene expression matrices.

We reassessed AD-PD overlap with DGEs from Seattle-AD and Smajić-PD dataset pair (Fig. S7). Across all pairwise combinations of relevant AD-PD cell types, the maximum observed correlation was between DEGs was between endothelial cells (-0.5; FDR q-value<0.05) and microglia (0.3; FDR q-value<0.05). In addition, a GSEA comparative analysis between AD and PD revealed extremely low number of shared terms (2 terms; Fig. S7B). Taken together, these results suggested that the univariate DEGs identified limited molecular overlap between AD and PD.

In summary, these findings demonstrated that DGE captured only modest overlaps between the molecular signatures of AD and PD brains. In contrast, our supervised latent factor modeling approach not only recapitulated the strongest DGE-derived AD-PD molecular overlaps (cross-disorder microglia), but also revealed a substantially broader set of shared, disease-relevant signatures. This comparative analysis highlights the added value of identifying cross-disease associations through a multivariate, transcriptome-wide modeling of gene modules, rather than relying solely on individual gene-level differences.

GWAS-seeded co-expression networks also indicate AD-PD genetic overlap

In an alternative set of analyses, we pursued the same research question, the extent of AD-PD overlap, using a complementary quantitative workflow. Devising a top-down framework (Fig. 6A), seeded with 164 genes mapped from GWAS risk loci (GWAS in AD or PD), we constructed disease-specific differential gene co-expression networks (DGCN) using the Rosmap-AD and Kamath-PD datasets. The notion of gene co-expression networks (GCN) rests on the assumption that genes with co-varying expression profiles across cell transcriptomes, often share functional or regulatory relationships [48–50].

We computed DGCNs independently for the Rosmap-AD and Kamath-PD datasets and separately for each cell type within a dataset (Methods). By quantifying the alignment between our two analysis arms (PLS module arm and seed gene derived co-expression analysis arm), we measured the correspondence between the relevant genes from the DGCN analysis (top-down) and the PLS-derived gene modules (bottom-up). Specifically, we looked at the number of robust genes that were common in each gene module - GWAS DGCN pair (Fig. S8A). We observed high PLS_{cell} module-DGCN alignments within the same cell types. For example, co-expression signatures from astrocytes shared, on average, 20% of genes (significant ρ at FDR < 0.01) with PLS_{cell} Rosmap-AD Ast 2. Similarly, excitatory neurons shared, on average, 58.4% of genes (significant ρ at FDR < 0.01) with PLS_{cell} Rosmap-AD Ex 1 and 42.2% of genes with PLS_{cell} Rosmap-AD Ex 1.

We then quantified the extent of AD and PD overlap in yet another way. Using Kendall's tau-b, we computed the similarity between the same seed-derived DGCNs across AD-PD cell type pair (see Methods). Embeddings derived from this similarity matrix (164 GWAS genes x 54 AD-PD cell type pairs; PCA; Methods) revealed systematic AD-PD cell pairings that shared similar co-expression profiles (Fig. 6C). The first principal component, accounting for 25.6% of the variance in seed-derived co-expression patterns, was dominated by robust AD-PD neuronal cell types (subjected to bootstrap robustness check; see Methods). The second component (14.4% of total variance) grouped DA neurons in PD (CALB1 and SOX6) with excitatory and inhibitory neurons from AD. The third component was dominated by combinations of microglia in AD and oligodendrocyte and OPCs in PD (9.5% of total variance).

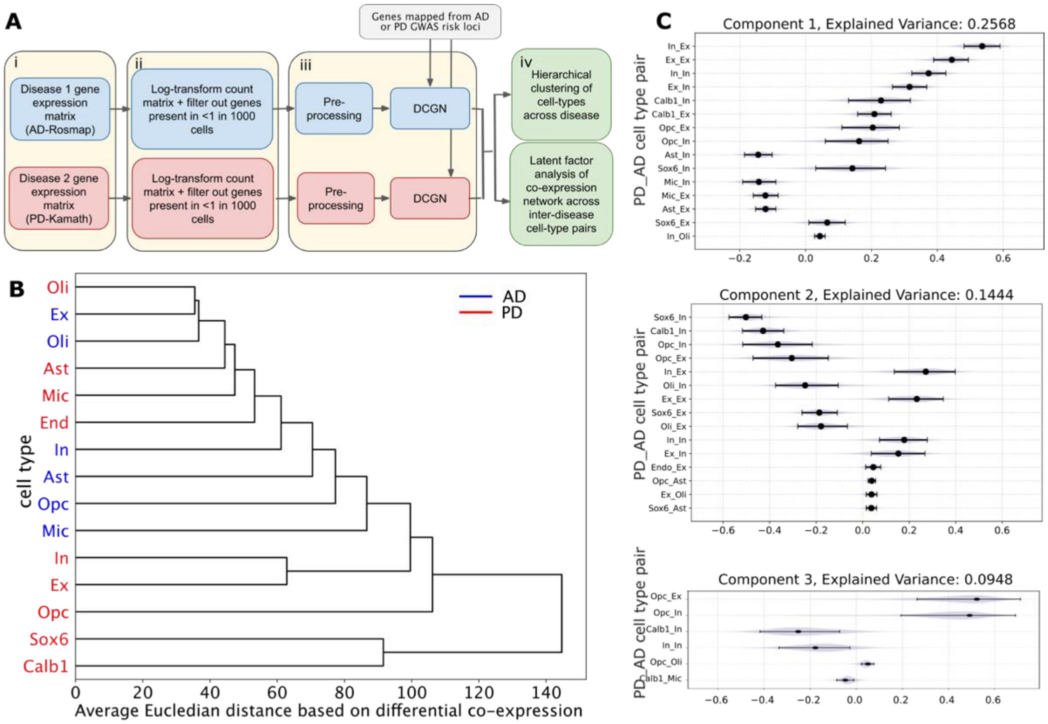

Figure 6. Differential gene co-expression network identifies hints of overlap between AD and PD. To corroborate our findings in a technical replicate, we contrasted differential gene co-expression networks (DGCNs) between AD and PD. We constructed DGCNs based on genes whose expression patterns were co-expressed with seed genes (164 GWAS-nominated genes from AD or PD), but whose synchrony was altered in disease compared with control conditions. **(A)** Overview of workflow. (i) Single-nucleus RNA seq datasets were downloaded from open-source repositories. (ii) Local processing was performed independently for each cell type, following recommended guidelines. (iii) Gene co-expression network analysis. For each gene from a set of GWAS implicated genes from AD and PD, a co-expression network was created for the disease and control groups independently. The results were subtracted to generate the differential gene co-expression networks (DGCN). (iv) The disease-specific DGCNs were compared between AD and PD to identify cross-disease cell type associations. **(B)** Hierarchical clustering of cell types based on the similarity of the co-expression patterns captured by DGCN. The dendrogram captures the average Euclidean distance of DGCNs across all cell type pairs between AD and PD. **(C)** Kendall's tau-b was used to calculate the pairwise associations between DGCNs. Cell type pair loadings for the top 3 principal components are shown. The pairs are presented in descending order, based on the loading magnitude. Dots represent the empirical PCA loading. Error bars represent the 20/80% CI from 1000-iteration bootstrap analysis which sampled rows from a DGCN with replacement. Pairs that did not include zero in their bootstrap interval are shown. The first and second principal components are dominated by cell type pairings involving mainly excitatory and inhibitory neurons in AD and PD. The second component emphasize PD dopaminergic neurons. The third component focuses on glial and vascular cell types from PD across cell types from AD.

Taken together, the results from the DGCN analysis recapitulated our gene module-based observations, confirming the strongest AD-PD overlap between oligodendrocyte- and neuron-

derived modules. Overall, this parallel analysis reinforced the robustness of our earlier results through a technical replication.

Discussion

In this study, we examined the molecular and cellular ties between Alzheimer's and Parkinson's disease. By analyzing the entire protein-coding transcriptome at single-cell resolution, our multivariate approach uncovered shared AD- and PD-deviant genes forming co-expressed modules at sub-cell type granularity. Further, we mapped the gene modules to disease-relevant biological programs, illuminating complex mechanisms that might lead to shared disease neuro-phenotypes. In summary, we provide single-cell genomics scientists with a tool to compare any pair of diseases from a global transcriptome perspective.

Our primary analytical protocol was enabled by access to an AD dataset (Rosmap-AD) with 70,634 nuclei from 8 major cell types, and a PD dataset (Kamath-PD) with 340,902 recorded nuclei from 11 major cell types. From both datasets, we extracted multiple gene modules for each cell type. These signified distinct modes of sub-cell type disease-related transcriptional changes. Our grading of the alignment of gene importance between pairwise gene modules from AD and PD demonstrated sizable overlaps. In the spotlight were modules derived from AD and PD glial cell types — oligodendrocytes, astrocytes and microglia. Significant, albeit more limited overlap, also emerged between AD and PD neuron-derived molecular programs.

This high degree of transcriptional changes mirrored between AD-PD was also highlighted in a secondary analysis arm, which compared gene co-expression networks between the two diseases. Additionally, we were able to replicate these findings in an external AD-PD snRNA-seq dataset pair (Seattle-AD, Smajić-PD). Overall, our multivariate transcriptome-derived analyses offered systematic evidence of shared transcriptomic signatures associated with AD and PD.

To situate our findings within the context of disease-relevant GWAS risk genes, we examined whether GWAS hits, from either AD or PD, were included in our gene modules. We found that these disease marker genes played robust roles within our functionally integrated gene modules, despite being extracted in a transcriptome-wide approach. Additionally, we observed clear cell type localization of the most popular genes within our gene modules. For example, APOE signatures localized to microglia and astrocyte gene modules, agreeing with previous single-cell transcriptomics studies in AD[36,37].

Central to our investigation, several AD-relevant GWAS genes surfaced in PD-associated gene modules and vice versa, highlighting the interconnectedness between the two disease categories. For example, APOE had robust PD associations through several PD gene modules, including those pulled from microglia, astrocytes, oligodendrocytes, and neurons. Indeed, prior clinical studies have highlighted APOE to be predictive of cognitive decline in PD patients[51,52]. Additionally, APOE has been shown to exacerbate PD pathology in mouse models[53]. As another example, we considered SNCA, a major gene implicated in PD GWAS [54]. In the PD brain, misfolded SNCA protein, is a primary neuropathological marker[55]. Here, along with several PD

neuron and glial modules, SNCA emerged as a strong contributor in AD excitatory neuron modules. Previously, APP transgenic mice with SNCA knockout have demonstrated a significant reduction in amyloid burden, hinting at connections between this PD gene and AD pathology[56,57]. Taken together, we have thus situated GWAS AD or PD genes within the fuller context of disease-specific gene modules and further expanded the implication of these genes to potential cross-disease correspondences[12].

Drawing biological insights for our AD and PD gene modules, we identified potential shared disease mechanisms from pre-curated gene ontologies. We identified shared alterations in cellular energy metabolism and stress response, inflammation, lipid signaling, protein folding, and protein degradation cascades. Some of these overarching disruptions in biological processes have been discussed in reviews summarizing the collective understanding from decades of neurodegeneration research[57]. Here, in a clean bottom-up workflow, our study confirmed these prior findings, while going beyond them by carefully localizing these molecular processes to cell-type-specific gene modules.

For example, a shared feature of neurodegeneration is presence of abnormal protein aggregates[58,59]. In line with this, our enrichment analysis revealed that protein misfolding (ER stress) and its associated biological processes were widespread across all major cell types, diseases, and datasets. We also detected extensive alterations in molecular pathways related to protein degradation (ubiquitin protein ligase binding and clathrin-mediated endocytosis), indicating a potential breakdown in protein disposal systems in diseased cells. This aligns with findings from animal models where malfunctions in ubiquitin-dependent protein clearance was linked to neurodegeneration[60–62].

Impaired molecular mechanisms shared between AD and PD in neurons

Neurons are particularly sensitive to proteasomal turnover due to their longevity and delicate synaptic regulatory requirements[62]. The toxic effects of mis-aggregated proteins ultimately lead to neuron death and usually mark the final stages of neurodegeneration. Zooming in on neuronal gene modules, we identified neuron exclusive mechanisms that were shared between AD and PD. These were primarily related to alterations in cytoskeleton dynamics, impaired mitochondrial functions, and apoptosis mechanisms.

Further, we found microtubule-associated processes localized almost uniquely to neuronal modules in both AD and PD (genes included APP, NEFL, TUBA1B, GAPDH, TUBB2A, CALM3, and MAPT). Several lines of prior evidence, including microscopy and genetic studies, have cited defects in cytoskeleton dynamics as major contributors to neuronal death [63]. Further, alterations in post-translational modifications of microtubule acetylation levels have been reported in in-vitro and in-vivo studies of both AD and PD[64,65].

In addition, our neuronal modules are also enriched for terms related to alterations in mitochondrial functions, including axonal transport of mitochondria and protein localization to mitochondria (all 4 datasets). This alludes to a vicious cycle between dysregulated microtubules and impaired mitochondrial transport[66]. Previous research has shown that alterations to these dynamics leads to the overproduction of mitochondrial reactive oxidative species (ROS)[67,68]. ROS,

in turn, exacerbates the levels of free tubulin[69] which in turn has been shown to interact with proteins like α-synuclein, promoting the formation of oligomeric aggregates in the form of Lewy bodies[70], or tau tangles[68].

The involvement of the MAPT gene in disease-relevant neuron modules from both AD and PD was also noteworthy. This gene encodes the protein tau and is responsible for stabilizing axon microtubules. Disruptions and alterations in this gene has been previously associated with multiple tauopathies and PD. Tau, in its aggregated form, demonstrates prion-like behavior, passing from neuron to neuron across synapses, a mechanism increasingly recognized in both AD and PD [71–75]. In our analysis, the emergence of MAPT across both disorders highlights a shared therapeutic target, motivating cross-disease strategies aimed at limiting pathological protein spread and neuronal death.

Apoptosis and cytochrome c regulation pathways

The emergence of terms related to the regulation of cytochrome c release and the apoptotic signaling pathway in several AD and PD neuronal gene modules was notable. In an intact human cell, cytochrome c is present in the mitochondrial intramembranous space. However, oligomeric forms of amyloid-β, α-synuclein, and tau has been shown to increase mitochondrial membrane permeability, causing leakage of cytochrome c into the cell cytosol[76]. This loss disrupts cytochrome c oxidase function and compromises ATP production. Once in the cytosol, cytochrome c initiates mitochondria-mediated apoptosis [77], a form of neuronal death long suspected as an early event in the pathophysiological cascade leading up to AD[78] and PD[79,80]. Specific to PD, in a self-reinforcing manner, impaired cytochrome c release from mitochondria is thought to escalate α-synuclein oligomerization via radical formation[81]. Whether similar mechanisms exacerbate plaque and tangle aggregation in AD remains an important avenue for further investigation.

Oligodendrocyte and Oligodendrocyte precursor cell modules in AD and PD

In oligodendrocytes and OPCs, we located gene modules with high similarity between AD-PD. Three key observations stand out related to these modules.

First, we observed that a sizeable portion of GWAS genes localized to cross-disease oligodendrocyte modules. Notably, PD oligodendrocyte modules contained more GWAS genes than modules from other cell types. Previous transcriptomic studies have pointed out that several disease risk loci are associated with oligodendrocytes in both AD[82] and PD[83]. Our study confirms and expands on this observation.

Second, in our enrichment analysis, several biological processes revolving around myelination and regulation of axonogenesis were specific to these cell type modules and were shared between AD and PD. Prior correlative macroscopic brain-imaging studies have linked deteriorating myelin health to AD progression[84]. Further, a recent invasive experiment posited a causal link between aging myelin and AD[85]. In a few AD mouse models and a human PD model, transcriptomic analysis identified changes in oligodendrocyte transcription specifically related to impaired myelination[35,86,87].

Finally, in addition to strong intra-cell-type module associations, our AD oligodendrocyte modules also exhibited a high degree of association with excitatory neuron modules from PD. Such similarity between oligodendrocyte and excitatory neuron transcriptional modifications in AD was previously reported in the Rosmap-AD transcriptomic study, in terms of shared DEGs[36]. The extension of this overlap to cross-cell-type modules from AD and PD suggests pervasive crosstalk between excitatory neurons and oligodendrocytes in neurodegeneration.

Shared role of heavy metals is highlighted between AD and PD

In our study, gene modules from astrocytes showed exclusive enrichment for response to zinc ions in both AD and PD. Several metallothionein (MT) genes were common between these modules (MT1E, MT1G, MT2A). In general, dysregulated homeostasis of heavy metals is believed to lead to an increased risk of the onset and progression of neurodegenerative diseases, as evidenced by studies in both humans and animals[88,89]. Astrocytes, in particular, have been shown to remove excess heavy metals from the brain parenchyma[90], and in vitro models have shown that zinc induces harmful A1-type reactive astrogliosis which promotes synaptic degeneration in neurons[91].

Consistent enrichment of terms related to copper (Cu) regulation across neuronal and glial modules in AD and PD in our analysis aligns with the previously implicated metal dyshomeostasis axis linked to ROS and protein misfolding [92–94]. Biophysical and biochemical experiments underscore these metal pathways, showing that altered Cu ion levels can trigger misfolding of α-synuclein[95] and amyloid-β[96].

However, here, a key distinction emerged between the copper handling gene modules — glial modules included genes related to buffering/detoxification responses (via MT1E, MT2A, MT3, and APP), whereas excitatory neuron modules uniquely enriched copper-binding genes tied to oxidative stress and proteostasis (via critical antioxidative enzymes (SOD1, PARK), SNCA, and the copper chaperone protein (ATOX1)). Prior in-silico analyses of microarray data on brain tissue had reported a similar grouping of copper-handling genes (metallothionein group and the enzyme binding group)[97]. Therapeutically, these findings support cell type-targeted interventions; for example, enhancing glial copper-buffering capacity (e.g., boosting metallothionein pathways) to stabilize extracellular redox balance, while simultaneously protecting neurons with copper-modulating and antioxidant strategies (e.g., targeting SOD1- or ATOX1-lined pathways) to reduce ROS-driven proteotoxicity. Thus, our cell-type-specific module-based approach provides important clues for precise drug treatment design.

In parallel, iron ion homeostasis terms were found to be enriched in neuron gene modules from both AD and PD. Across all datasets, excitatory and inhibitory neuron modules were involved. In the brain, iron plays a key role in myelin synthesis, neurotransmitter production, and overall metabolism[98]. However, elevated levels of redox-active iron, often originating from degenerating mitochondria, accumulate in several neurodegenerative diseases as evidenced by biochemical, and transgenic animal studies[99–102].

Together, these findings position disruptions of heavy metal pathways as shared therapeutic targets in AD and PD.

Shared microglia-specific molecular changes in AD and PD

Functionally, microglia are the primary immune cells of the CNS[103], and neuroinflammation and immune system dysfunction are believed to be key components of neurodegeneration[104]. Consistently, microglial gene modules from our study showed enrichment for several immune-related processes. For example, T cell activation terms were associated with microglial modules for both AD and PD (all 4 datasets). Microglia, upon activation by neuronal stress, are thought to release pro-inflammatory cytokines and upregulate MHC class I and II molecules[105]. Further, the inflammatory cytokines can induce the expression of adhesion molecules on brain endothelial cells, compromising the integrity of the blood-brain barrier (BBB). This BBB breakdown accelerates peripheral immune cell entry. Thus, in a chicken-egg scenario, microglia and endothelial cells drive a chain reaction of T cell activation, oxidative stress, and neuroinflammation[106–108]. This domino effect may have been captured in one of our PD endothelial modules whose pathways were associated with T cell activation terms as well as association with biological processes like leukocyte adhesion to vascular cells, blood vessel morphogenesis and diameter maintenance, pointing to the dysregulation of the BBB. This cascade of immune response events might exacerbate ROS production and neuronal damage[106,107].

Further, we found a robust response to thyroid hormone (TH) related terms in microglial gene modules from both AD and PD. Within the brain, TH imbalances are an important contributing factor to increased ROS [109]. Epidemiological evidence have linked multiple thyroid-related autoimmune diseases to increased prevalence of both AD[110,111] and PD[112]. However, the exact contribution of TH to either AD or PD pathophysiology has not been fully established. Recently, an AD mouse model has linked brain hypothyroidism with reduced microglial reactions to inflammatory stimuli and aberrant amyloid-β[113]. In PD, an α-synuclein PD mouse model found connections between TH and glucocerebrosidase activity in microglia[114]. Our findings highlighting TH involvement in both AD and PD microglial states warrants further investigation of the thyroid axis.

We also identified microglial gene modules linked to synapse pruning in both AD and PD. These modules implicated key genes, including TREM2 and several C1q genes from the complement system, an integral component for synaptic refinement. Synapse loss is frequently observed as an early event in animal models of neurodegeneration[115–117]. Variants in the TREM2 protein and aberrant activation of C1q genes have been shown to cause irregular synapse pruning in studies on AD mouse models[117–119]. In a PD mouse model, suppressing TREM2 gene products in microglial cells was shown to accelerate the loss of DA neurons[120,121]. Yet, the totality of glia-synapse interactions, especially the role of the complement system in PD, is under-investigated[122,123]. Once again, our bottom-up approach identified a common transcriptomic signature in AD and PD in the form of genes related to synapse loss – a potential area for further investigation.

In addition, lipid transport regulation terms were enriched in both AD and PD microglial gene modules (lipid, phospholipid, cholesterol, and sterol transfer terms) and involved key genes including APOE, TSPO, TMEM30A, several ATP-binding cassette subfamilies (ABC) genes (ABCG1, ABCA5) and NPC2. Excess lipid has been reported to accumulate as droplets in human iPSC-derived microglia, reducing their phagocytic capabilities and increasing the secretion of pro-

inflammatory cytokines[124,125]. To sum up, the above impairments mediated by microglia ultimately exacerbates ROS burden and neurotoxic buildups leading to neurodegeneration.

In conclusion, our proof-of-principle investigation of the transcriptomic terrain intersecting AD and PD identified and characterized the key shared molecular basis of neurodegeneration. We were able to (i) quantify the degree of overlap between AD and PD, and (ii) map these overlapping molecular changes in brain tissue to distinct cell types with shared disease-associated signatures in AD and PD. In addition, the cell type specific genes identified within converging AD-PD associated modules may represent potential therapeutic targets that warrant further investigation.

Several limitations should be considered when interpreting our findings. While the present study focuses on the computational identification of disease-relevant gene programs and a subsequent comparison between AD and PD, future experimental work using cellular systems or animal models targeting the highlighted modules and shared pathways will be necessary to further investigate AD-PD convergence in vivo. For example, recent animal research on gut-brain axis disruptions in AD identified common therapeutic intervention strategies that might be applicable across neurodegenerative disorders [32,126]. In addition, although the datasets analyzed in this study are among the largest currently available, larger and balanced cohorts will likely enable more robust estimation of gene modules, providing more granular insights into sex- and cell type-specific transcriptional basis of AD-PD overlap.

Future work can expand our study to include a greater diversity of neurodegenerative, neurodevelopmental, and psychiatric diseases. Moreover, applying a similar computational framework to different, readily accessible source transcriptomes, like blood or cerebrospinal fluid, can identify critical biomarkers for neurodegeneration.

Materials and Methods

Single genomics data resources

Primary datasets

ROSMAP Alzheimer's dataset[36]: The snRNA-seq dataset was derived from postmortem brain tissue from the prefrontal cortex (BA10) of individuals participating in the Religious Orders Study or the Rush Memory and Aging Project (ROSMAP)[127]. The dataset was collected from 48 subjects who were carefully matched in terms of age and sex (24 males and 24 females). Of these, 24 individuals were diagnosed with AD and 24 were control subjects. The mean age was 85 years. The recorded cell types included excitatory neurons (n = 34,976), oligodendrocytes (n = 18,235), inhibitory neurons (n = 9,196), astrocytes (n = 3392), Opc (n = 2,627), microglia (n = 1,920), pericytes (n = 167), and endothelial cells (n = 121). The dataset encompassed transcript counts for 17,926 protein-coding genes, aligned with the human reference transcriptome hg38 (GRCh38.p5).

Parkinson's disease, Kamath dataset[40], GEO accession number GSE178265: This dataset included snRNA transcriptomes from postmortem human midbrain and dorsal striatum (caudate nucleus and substantia nigra) tissue. Tissue samples were derived from an age and sex-matched cohort of 21 subjects in total (control, PD and Lewy body disease). Samples derived from patients

with Lewy body disease (4) were excluded from our analysis. To match the mean age of the ROSMAP-AD cohort, 3 patients less than 50 years of age were excluded. The final dataset consisted of 6 PD patients, while the remaining 7 were controls. Among this group, 6 were males and 7 were females. The mean age of included individuals was 83 years. Major cell types in this dataset included oligodendrocyte (n = 134,940), excitatory neuron (n = 40,956), inhibitory neuron (n = 31,545), SOX6 (n = 25,482), astrocyte (n = 24,475), microglia (n = 24,038), CALB1 (n = 15,871), endothelial (n = 12,609), Opc (n = 9,603), macrophage (n = 955), ependyma (n = 167). The dataset provided transcript counts for 33,692 genes which were aligned to the hg19 genome.

External validation datasets

Seattle Alzheimer's dataset[37]: The authors of this resource sourced brain specimens from the Adult Changes in Thought Study and the University of Washington's Alzheimer's Disease Research Center. Brain tissue samples were drawn from the middle temporal gyrus. The study included participants from age groups ranging from less than 65 years to more than 90 years. To maintain consistency with our other datasets, we considered only participants over the 65 to 77-year age bracket. This gave us 76 individuals, with 47 females and 29 males. Out of these, 36 subjects had recorded dementia, and 40 were controls. The mean age of the individuals was 88 years. The preprocessed datasets for the cell types labeled 'L4 IT', 'L5 IT', 'Vip', 'Pvalb', 'Sst', 'Sncg', 'Oligodendrocyte', 'Microglia', 'Astrocyte' and 'OPC' were used. This resulted in the following nuclei being available for our analysis: sncg (n = 22,168), sst (n = 58,265), pvalb (n = 90,804), astro (n = 70,009), endo (n = 2,069), opc (n = 32,493), micro (n = 40,000), oligo (n = 111,194), l4_it (n = 168,860), l5_it (n = 128,090), and vip (n = 104,514). In total 36,517 genes were recorded and mapped to hg38 (GRCh38-2020-A) human reference genome.

Parkinson's disease, Smajić dataset[35], GEO accession number GSE157783: The creators of this resource worked with postmortem midbrain tissue sections that were linked with clinical and neuropathological data from the Parkinson's UK Brain Bank and the Newcastle Brain Tissue Resource. The dataset included age and sex-matched nuclei samples from 6 controls and 5 idiopathic PD patients, all of whom exhibited severe neuronal loss in the substantia nigra and had no family history of the disease. The mean age of the subjects was 80 years. The major cell types were oligodendrocytes (n=21,268), astrocyte (n=4,708), microglia (n=3,903), excitatory neurons (n=3,037), OPC (n=2,754), endothelial cells (n=1,723), inhibitory neurons (n=1,548), and pericytes (n=1,229). Four cell types had fewer than 500 nuclei and were excluded from our analysis — ependymal cells (n = 536), GABA (n = 535), CADPS2+ neurons (n = 120), and DaNs (n = 74). The total number of genes was 24,005, and these were mapped to hg38 reference genome.

Control dataset

Lung disease, Kaminski dataset[128], GEO accession number GSE136831: The authors of this chronic obstructive pulmonary disease dataset sourced nuclei from human distal lung parenchyma specimens. In total, 18 COPD patients and 28 control donor lungs were sampled. This resulted in the following nuclei being available for our analysis: myeloid (n = 174,146), multiplet (n = 3,765), lymphoid (n = 34,626), stromal (n = 6,607), endothelial (n = 2,069), epithelial (n =18,030). In total 32,922 genes were recorded and mapped to hg38 (GRCh38) human reference.

Preprocessing pipeline at source

We relied on the preprocessed datasets from the authors responsible for the data collection (cf. above). This maximizes reproducibility and compatibility with other studies working with these resources. The transcriptomic datasets were processed in the source studies using standardized snRNA-seq processing pipelines. This included quality control for cell inclusion, including doublet detection, the removal of low-quality and outlier cells, the removal of lowly expressed genes, and sample-level batch correction procedures.

Cell type classification — which cell belongs to which cell population — was taken from the original studies as a basis for our investigations. Exact details can be found in the Methods sections of the independent research.

Primary local preprocessing

Different cell types perform widely diverse functions, each arising from the functional recruitment of distinct gene groups. In our pursuit to discover biologically meaningful gene groups, we implemented our quantitative analysis pipeline for a given disease on a cell type-by-cell type basis. This agenda enabled us to extract coherent latent components (latent factor/loading vector, hereby referred to as gene module) specific to each cell type.

To ensure an even sample size in the disease and control group, we randomly sub-sampled transcriptomes of a given cell type to have comparable counts of nuclei from both disease and control instances. On this sub-sampled data, we removed genes that were captured in fewer than 1 out of 1000 cells to reduce our model's degrees of freedom (*scanpy.pp.filter_genes*), which is in line with previous research[129,130]. To reduce technical variation from sequencing depth, we normalized the data by dividing the raw UMI count by the total number of detected UMIs in each cell (*scanpy.pp.normalize_total(*data, target_sum=1e4)). To account for the heteroskedasticity originating from differences in highly expressed vs lowly expressed genes, we log-transformed the normalized gene expression data (*scanpy.pp.log1p*(data)). Taken together, these dataset transformations have been shown to work well as a preparatory step for downstream dimensionality reduction [131].

In an additional data cleaning step, postmortem interval (PMI) was included as a covariate to account for potential confounding effects on gene expression. PMI has been shown to be a potential source of confounding elsewhere[132,133]. Specifically, here we regressed out the variation in gene expression attributable to differences in PMI. The resulting adjusted, cleaned and standardized transcriptomic profiles for each examined cell were used for subsequent steps in our modelling pipeline.

We further restricted the feature space to protein coding genes in each dataset. This step reduced the ambient gene space dimensionality which is beneficial for any high dimensional analysis [134,135]. Specifically, for each dataset, we considered the genes that overlapped with the set of 17,019 protein-coding genes in Rosmap-AD. This filtering resulted in 16,936 genes in the Kamath-PD dataset, 12,681 genes in Seattle-AD, and 15,137 genes in Smajić-PD. As a result, the analyzed datasets were embedded in ambient feature space of comparable dimensionality, with a

similar set and order of genes, with broadly consistent biological properties being evaluated across analysis.

Crucially, the transcriptomic datasets from different studies were — at no point — merged or jointly integrated at any stage of the analysis. Instead, each dataset was analyzed independently to derive disease-associated gene modules. For each dataset, model fitting and hyperparameter optimization were performed separately. By avoiding cross-dataset integration, our analysis circumvented the need for batch-effect correction across datasets. Thus, comparisons between AD and PD were performed at the level of model derived gene loadings, not experimentally recorded gene expression, and only after gene modules had been derived independently within each dataset.

Identifying gene modules: supervised latent factor modeling

At the heart of this study, we sought to identify synchronous gene expression changes that occur in the brain in association with disease state and compared them between AD and PD. To achieve this, we employed a latent factor approach to identify gene programs in a multivariate framework, rather than examining individual genes independently. Unsupervised latent factor models are widely used in snRNA-seq analyses to identify hidden gene programs [136–138]. In contrast, here we implemented a supervised variant of this framework that identifies structured patterns in high-dimensional gene expression data while simultaneously modelling the relationship between observed variables and their associated disease status.

Our work builds on a prior study in which the multivariate method, partial least squares discriminant analysis (PLS-DA)[139], was employed to derive AD-predictive gene modules across major brain cell types[42]. In contrast to the original study, where the analyzed dataset had favorable observations to features ratio, several datasets analyzed here comprised substantially fewer samples within individual cell types. In these cases, PLS-DA is susceptible to overfitting, particularly given the inherent noise and sparsity of snRNA-seq data[140]. To address this limitation, we applied principal component analysis (PCA) denoising on the input gene expression matrix, prior to model fitting. Under a low-rank assumption on the input feature space (gene expression), dimensionality reduction with PCA yields an optimal low-rank approximation which is robust to noise and sparsity[27,141]. In practice, such transformations are routinely applied in single-cell and single-nucleus analysis, where the ambient gene space is well approximated by a low-rank structure [142].

Specifically, for each cell type within individual datasets, we applied PCA to the input gene expression matrix. These components were then used as input variables for PLS-DA to obtain a projection maximizing disease versus control class separation. To guard against spurious derived structures [143], we performed strict model validations (see below), including label-shuffled permutation tests to assess the statistical significance of derived components (Fig. S1B, S3B).

Formally, let $X_{ambient} \in R^{N_c \times \mathrm{M}}$ be the input gene expression matrix, and $y \in R^{N_c \times 1}$, where M is the number of genes, and N_c the number of observations (nuclei) for a cell type c. Y represents the disease label (+1 for disease and -1 for control) for each nucleus. Let M_c be the number of chosen PCA components. Then, the input datasets to the PLS-DA models can be denoted as $X \in R^{N_c \times \mathrm{M}_c}$.

Concretely, PLS-DA can be viewed to consist of two key equations:

$$X = TP^T + E,$$

$$Y = UQ^T + F,$$

where T and U are $N_c \times k_c$ score matrices of k_c extracted components, P is a $M_c \times k_c$ loading matrix (effect size) of X, and Q is a $1 \times k_c$ loading vector of Y respectively. E and F are the residual matrices of X and Y respectively. The decomposition of X and Y is set to the solution of the optimization objective:

$$cov(t, u)^2 = cov(Xw, Yh)^2$$

where $cov(t, u)$ is the captured covariance $\frac{t^T u}{N_c}$, w and h are weight vectors that are extracted using the NIPALS algorithm[144].

To transition from the PCA embedding space (M_c) back to the gene space (M), we projected the PLS loadings back into the original gene expression space (approximated using sklearn *PCA.inverse_transform)*. This ensured that the domain interpretability of the PLS estimates was preserved in the biological ambient space — high absolute gene loadings signaled strong contributions (positive to the target disease, negative to the control group), while near-zero loadings indicated minimal impact. To ensure statistical robustness of the derived gene-level loadings, we focused on genes that were consistently selected across model refits based on bootstrap resamples of the dataset (see below). This procedure allowed us to identify genes that were stable across different realizations of the data, further reducing the likelihood that the reported results are driven by sampling noise or unstable features.

Model selection, training, and performance assessment

After preprocessing and cleaning the transcriptomic data resources, we carried out the training of our supervised learning models. First, the number of PCA components was chosen using $min(500, N_c)$, where N_c was the number of recorded nuclei for the given cell type c in the dataset. Next, the optimal number of latent components for each cell type-specific PLS model was determined using a rigorous 10-fold cross-validation (CV) scheme. Selecting this optimal number of latent components is crucial — choosing too few components implies losing out on important information and too many leads to overfitting. To do this hyperparameter selection, the set of transcriptomes was randomly split into 10 equal-sized data point subsets. We ensured that the disease-to-control ratio of cells for each subset reflected that of the full dataset. Screening a range of component choices (1 to 8), in each iteration, 9 out of these 10 data subsets were combined and used for training a PLS model, while the out-of-bag subset was used to evaluate the component number choice. The model's performance was evaluated based on the area under the receiver operating characteristic curve (AUROC) in disease discrimination. This was performed for all combinations of training and validation subsets (*scikit-learn model_selection.GridSearchCV* function with *PLSRegression* as the 'estimator', 'scoring' set to 'roc_auc' and 'n_components'

parameter set to 1-8). The number of components yielding the maximum mean AUROC over the CV subsets was noted as optimal for the given cell type.

For each cell type, we then fitted optimal PLS models on the full set of transcriptome observations (PLS model specified using *sklearn.cross_decomposition* module *PLSRegression*). To audit the performance of the individual PLS models, we employed AUROC of disease classification as our evaluation metric. Given that cell samples from a patient can exhibit significant autocorrelation, it is crucial to account for this when evaluating the model. Traditional test-train splitting methods often involve blindly partitioning the dataset. This can result in overly optimistic test performance and makes it challenging to detect overfitting during the testing phase[145]. In light of this, we employed a variation of cross-validation combined with bootstrapped Latin partitions[146] to ensure patient-level stratification.

Concretely, in each iteration, a random sample of subjects (not cells) was drawn with replacement. This formed the basis of the training dataset's nuclei source. Based on the subset of patients, a random sample of cells was drawn, with replacement, while ensuring that the disease-to-control ratio of nuclei was reflective of the empirical dataset. The percentage of cell samples from any given subject was also preserved in each iteration. This analytical protocol ensured that transcription signatures from the same patient were not present in the training and testing set at the same time. Individual PLS models were fitted on the re-sampled train dataset. This fitted model was then evaluated based on AUROC scores on the out-of-bag nuclei (test set), i.e., the transcripts from subjects that were not included in the training step. We performed 1000 iterations of this bootstrap-based model disease classification performance evaluation. This allowed for a principled assessment of the disease discrimination strength of the PLS solutions based on the cell type-specific gene transcription signatures.

Statistical tests of PLS derived gene modules and gene loadings

Our cell type-specific hyperparameter selection allowed us to independently derive the number of PLS-DA components (gene modules) that maximized disease-vs-control identification within each cell type. The statistical significance of an overall gene module was assessed in a principled, non-parametric permutation procedure. In 1000 permutation iterations, the transcriptome signatures were held constant, while the disease labels (outcome of model) were shuffled randomly across transcriptomes. The resulting surrogate datasets preserved the statistical structure of gene expression profiles while selectively destroying the association of the transcription profiles (model input) with diagnosis (model output). This approach generated a null distribution with minimal modeling assumptions[147,148].

The empirical covariance (test statistic) between the gene expression and disease signature captured by each module ($cov(t, u)$ defined above; t=*model*.x_scores_; u=*model*.y_scores_) was compared with the resulting permutation distribution. This distribution reflected the null hypothesis of random association between gene transcription and the disease designation, which we test the actual model instance against. We deemed significant a module's input-output covariance in the latent space if fewer than 5% of the null models yielded a better covariance strength than the original covariance from the actual model instance (Fig. S1B). In case a module failed to pass this label-shuffling permutation test, it was dropped from further analysis, along with

all underlying gene modules for that cell type. Thus, in a data-driven approach, we were able to determine which gene modules in a cell type at hand carried enough information that allowed us to discern a biological signal from noise.

To identify the subset of the examined genes that robustly contributed to disease detection in each gene module from a cell type, we implemented a 500-iteration bootstrap (BS) scheme. The bootstrap resampling was done by selecting nuclei, with replacement, from the cell type observations before applying dimensionality reduction. This approach simulated random nuclei sample draws that could have been derived from the broader cell population. Dimensionality reduction (PCA) and PLS model estimation were performed on the resampled bootstrap dataset in an identical fashion (cf. above).

An inherent ambiguity of the class of latent factor models (i.e., aspects of model non-identifiability), including PCA and PLS, is the reflection invariance of derived latent vectors. To remedy this source of indeterminacy, we computed the cosine similarity (γ; range -1 to +1), between a BS loading vector and its corresponding empirical loading vector. In the scenario where γ was less than 0, indicating a flipped ("mirrored") loading vector, we multiplied the loading vector of the BS model elementwise by -1 to align it with the original loading vector. This method has been employed by previous authors to address the issue of reflection[42].

The resulting distribution of loadings for a gene in a module was compared to its counterpart in the original model estimate. We disregarded any genes whose model coefficient (PLS loading corresponding to this gene) included zero in its 5/95% BS-confidence interval (CI) for that module. That is, the gene effect was removed by setting the loading value to zero. The resulting 'robust' gene modules are denoted as P^* (dimension $p \times k_c$; cf. PLS definition section above) in future references.

To evaluate the potential influence of demographic confounding factors, specifically age and sex, we modelled the component scores for each latent gene module as a function of diagnosis, age, and sex. For each gene module, component scores were regressed on diagnosis (primary variable of interest) together with age and sex (potential confounders) using ordinary least squares regression from the python *statsmodel* package (v0.14.4). Following model fitting, an analysis of variance was performed to quantify the proportion of variance in component scores (R^2) that was explained by each predictor (anova_lm, typ=2; *statsmodel* v0.14.4). In total, 72gene modules were analyzed (12 Rosmap-AD, 20 Kamath-PD, 25 Seattle-AD, and 15 Smajić-PD). This step provided a unique estimate of the contribution by each predictor, while accounting for other variables in the models.

PHATE visualization

To gain a synopsis of the uniqueness of different gene modules from a single cell type, we created a concise, low-dimensional representation of high-dimensional cellular transcriptomes. For this purpose, we applied dimensionality reduction using PHATE[149]. Compared to prevalent visualization techniques like tSNE or UMAP, PHATE is well suited to preserve both local and global structures in a dataset and can capture non-linear relationships in the transcriptomic information. We estimated a separate PHATE model for each cell type. We projected the

transcriptomes from each cell type into independent low-rank spaces (using the *scanpy external.tl.phate* function with default parameters except n_pca = 500). We colored the cells in the PHATE embedding space based on their PLS score from each gene module (*PLSRegression x_scores_*).

Quantifying degree of associations between cross-disease latent gene modules

To quantify the similarity between AD and PD at the level of gene expression patterns, we computed the association between the AD and PD gene modules. Importantly, a similarity metric (correlation) was computed across the derived model's predictive rules for a disease. That is, we did not pit the raw gene expression measurements against each other.

Formally, for a disease d and cell type c, the ith robust gene module (cf. above) can be denoted as ${p^*}^{d}_{c,i}$, where p^* is a vector of dimension $1 \times M$ (number of genes in the input feature space). A robust gene module can contain tens to thousands of genes with non-zero loadings (out of ~17,000 genes) while the remaining loadings are zero (cf. above). To minimize the impact of tied zero loadings in the correlation metric calculation between two modules, we considered only those genes that had non-zero weights in both modules (AND conjunction). This approach works well to identify groups of genes with similar disease contributions between two modules while ignoring genes that might have robust effect sizes in one disease but not the other.

Concretely, we employed Kendall's tau-b ranked correlation metric (τ_b) to evaluate pairwise correlations between AD and PD gene modules. Kendall's tau-b correlation effectively handles tied ranks and provides a more accurate measure of ordinal association between gene modules. This is unlike Pearson's-r, which assumes monotonicity and is unstable, or Spearman's-r, which is biased and difficult to interpret [150]. For all pairwise gene modules from AD (12 modules across all cell types) and PD (20 modules across all cell types), Kendall's tau-b rank correlation coefficient, $\tau_b\big((c1,i),(c2,j)\big)$, was calculated using the scipy function *stats.kendalltau*(${p^*}^{AD}_{(c1,i)}$, ${p^*}^{PD}_{(c2,j)}$), where $(c1,i)$ is the ith gene module for the cell type $c1$ present in the AD dataset, $(c2,j)$ is the jth gene module from the cell type $c2$ present in the PD dataset.

To independently assess the statistical significance of each coupled module association, we employed a non-parametric permutation procedure with the null hypothesis of random association between gene modules from different diseases. For each gene module pair, we re-utilized the label shuffling derived module weights (cf. above) to calculate a null distribution from 1000 permutation iterations. We only interpreted a module-pair's correlation coefficient that emerged as statistically significant against a 5/95% CI threshold.

In parallel, we reported the FDR corrected q-values for the computed pairwise τ_b, with multiple testing controlled using the Benjamini-Hochberg procedure [151] across all pairwise comparisons.

Yet another test was conducted to estimate the statistical sensitivity to sample-size of the coupled associations of cross-disease gene modules. Across 500 iterations, we randomly bisected the empirical AD and PD datasets (before cleaning and standardizing) into two pairs of AD-PD

subsets. We ensured to preserve the original proportion of cell type nuclei and the disease-control ratio for each cell type. Since the bisection reduced the effective observation size of each dataset to half, the downstream PLS fit enabled a robustness check of sample-size for derived gene modules. For each AD-PD subset pair, we ran our workflow steps A-D in parallel (Fig. 1), resulting in 2 analogous sets of gene modules (a couple of 12 AD and 20 PD modules). We then performed Kendall's tau-b correlation across these modules, giving us 2 correlation matrices of dimension 12 x 20. We unraveled these matrices and calculated Pearson correlated (ρ) of the absolute τ_b values. We used absolute values since we were interested only in association strength, not direction. This analysis allowed us to compare the τ_b correlation levels between different gene module pairs derived from smaller subsets of the AD and PD datasets.

Differential gene expression

Differential gene expression is ubiquitously used in snRNA-seq analysis to identify genes that show statistically significant differences in expression levels between two conditions or groups[152]. It is a univariate method, meaning it examines each gene individually, thus losing key information hidden in gene co-expression patterns. We employed this traditional method to serve as an acid test for our new approach.

Differential gene expression was performed using MAST (v1.36.0), implemented in R and accessed from Python using rpy2 [153]. Within each dataset, log-normalized single-nucleus expression data were analyzed separately for each cell type. For each gene, a generalized linear hurdle model was fitted with diagnosis (disease vs. control) as the primary variable of interest. Cellular detection rates were included to account for differences in gene detection across cells. Age, postmortem interval and sex were included as covariates to account for potential confounding effects.

Significance was assessed using likelihood ratio tests as implemented in MAST. Multiple testing correction was performed across all assessed genes using the Benjamini-Hochberg method[151]. Effect sizes were quantified using the estimated $\log_2$ fold change between disease and control groups. Genes meeting the statistical significance threshold after correction were designated as differentially expressed genes. The final DEGs were referred to as adDEGs for AD and pdDEGs for PD.

To estimate the pairwise association between cell-type-specific adDEGs and pdDEGs, we computed Kendall's tau-b using the log-fold change values of genes in the AD-PD AND conjunction set (see above). This resulted in a similarity matrix of dimension 6 x 9, corresponding to the number of AD (6) and PD (9) cell types. Statistical significance for each association was evaluated using a permutation test with 1000-iterations, in which the fold change values of overlapping genes were randomly shuffled. FDR correction was performed across all comparisons (54) using the Benjamini-Hochberg procedure.

Quantifying difference in association strengths between PLS and DGE derived conclusions: Welch's t-test

To formally quantify the cross-disease association information extracted by our latent factor approach versus DGE, we used a statistical test to compare the central tendencies of the respective correlation measures. Welch's t-test was a natural choice of method here as the number of correlated combinations being compared were different (240 τ_b gene module combinations from PLS and 54 τ_b cell type combinations from DGE), and the variances were not assumed to be equal[154,155]. Welch's t-test can be formally computed as:

$$t = \frac{\overrightarrow{X1} - \overrightarrow{X2}}{\sqrt{(s1^2 \, / \, N1) \; + \; (s2^2 \, / \, N2)}}$$

In our case, $\overrightarrow{X1}$ was the unraveled PLS correlation vector (1 x 240; cf. Fig. 2A) and $\overrightarrow{X2}$ was the unraveled DGE correlation vector (1 x 54; cf. Fig. 5). $s1^2$ and $s2^2$ were the variances of these vectors, $N1$ was the total number of cross-disease gene module pairs from the latent factor analysis and $N2$ was the number of cross-disease cell type pairs considered in the DGE analysis.

Identifying biological signaling pathways from gene modules: Gene Ontology enrichment analysis.

To query gene co-expression patterns regarding possible underlying biologically meaningful gene programs, we performed a gene set enrichment analysis (GSEA) [28]. Here, we used the GSEApy python package [156], which itself uses Enrichr[157]. GSEApy is designed to extract statistically over-represented gene sets (example pathways) from a ranked gene list encompassing the whole genome. We used the gene ontology (GO) biological process (BP), molecular functions (MF), and cellular component (CC)[158,159] databases. We focused on GO, as collectively, they cover the largest fraction of the genome. Concretely, for each gene module, we fed the PLS gene loadings for the entire transcriptome recorded in our datasets to the enrichment tool (*gseapy.Prerank* tool with parameters rnk = gene loadings, min_size = 15, max_size = 1500, and permutation = 1000 for significance testing). We reported the pathways that had an FDR threshold of at most 0.05. This step was repeated identically and independently for all gene modules from each cell type, across all datasets.

To verify that the gene set enrichment results were not an artifact of noise in the PLS modeling but rather had actual biological relevance, we turned to our permutation test-derived gene modules. In 1000 permutation iterations, we destroyed the relation between gene expression and disease label. Thus, the extracted gene modules captured noise. We fed the gene loadings from these modules into our GSEA pipeline to verify the specificity of the empirical enriched terms.

Gene network visualization

GO terms are organized in the form of a hierarchical tree [160] which can be downloaded here (OBO 1.4). This hierarchical organization often results in hundreds of hits from an enrichment analysis. One of the techniques widely used to crunch down this dense information is via network visualization [161,162]. This technique can help identify broad groupings of terms based on a chosen parameter of interest, for example, shared genes between different terms.

For illustration purposes, we utilized Cytoscape [163] to create a structured network of disease-relevant GO biological processes identified by an enrichment analysis (cf. above). Each node in the network represented a GO BP hit. The edges were formed based on predefined relationships between nodes conditioned on shared genes and whether they were part of the same regulatory network. The resulting network layout (*yFiles.organic* layout) automatically clustered the enriched terms into biologically meaningful groups, allowing us to identify major functional themes that were shared between AD and PD.

Differential gene co-expression network

As a complementary analytical pipeline, we sought to explore the transcription profile of the RNA-seq datasets in a top-down gene co-expression network (GCN) analysis approach. Specifically, we started with candidate genes mapped to AD or PD GWAS risk loci. Using these genes as seeds, we created networks of correlated genes, that is, we identified groups of genes whose differential expression change between disease and control closely matched a seed gene. Seeded differential gene co-expression networks (DGCN) have been previously used to identify regulatory changes in gene expressions across various conditions[48,50]. By taking a contrastive approach between disease and control (differential), the effects of housekeeping genes could be limited, and the residual patterns of covariation could be attributed to the effects of a disease.

To identify a set of seed genes, we utilized the most recent GWAS studies that reported AD or PD-associated risk genes significant at the whole genome level. We identified 108 GWAS hits associated with AD[44] and 129 GWAS hits associated with PD[45]. Out of these 237 genes, 5 were common (CTSB, WNT3, BCKDK, HLA-DQA1, and HLA-DRB1) between AD and PD, giving us 232 unique genes. Of these, a total of 164 genes (out of the 232 genes) were present across all considered datasets.

Next, for each individual dataset, we split them into disease and control groups based on the diagnosis labels provided. We further subdivided each of these groups into subgroups based on cell types. For each cell type ct, the co-expression vector for a single GWAS gene i with another gene j recorded in the snRNA-seq dataset was calculated as $\tau_b(e_i, e_j)$, where τ_b is the Kendall's tau-b correlation metric, e_i is the read count vector for i $\forall$ observations (nuclei) and e_j is the read count vector of j $\forall$ observations (nuclei).

Evaluating τ_b across all recorded genes gave us: $g_{ct,i}^{AD} \in R$, where M was the number of genes common to both AD and PD datasets (M = 16,936). Thus, each element of the matrix $g_{ct,i}^{AD}$ was a numerical value between -1 and 1, capturing the degree of correlation of gene j with GWAS gene i. Stacking the vectors for all GWAS genes gave us gene co-expression matrices $G_{C,ct}^{AD} \in R^{N \times M}$ and $G_{D,ct}^{AD} \in R^{N \times M}$, for the control and disease groups respectively, where N was the number of GWAS genes (N=164). From this, we formally computed the differential gene co-expression matrix for a single cell type $G_{ct}^{AD} \in R^{N \times M}$ as follows,

$$G_{ct}^{AD} = G_C^{AD} - G_D^{AD}$$

Next, we systematically explored the mutual relationships between the DGCNs across different cell types, without discriminating them based on disease. To this end, we employed a hierarchical clustering analysis. Our goal was to probe for clusters of cell types that featured similar genome-wide co-deviation of gene transcription.

Concretely, we unraveled G_{ct}^{AD} into a vector $u_{ct}^{AD} \in R^{1 \times NM}$, where $NM = 164 \times 16{,}936 = 2{,}777{,}504$, and combined them across 6 AD and 9 PD cell types to get $U \in R^{C \times NM}$, where $C = 15$. We computed a linkage matrix based on the Euclidean distance between two unraveled differential co-expression vectors for each cell type (*scipy.cluster.hierarchy.linkage*, parameters method = 'average', metric = 'euclidean'). The linkage algorithm hierarchically clustered the 15 cell types, across AD and PD, with the cluster groups indicating cell types with the closest co-expression patterns. We visualized these clusters as a dendrogram in python (*scipy.cluster.hierarchy.dendrogram*).

We refined our clustering-based qualitative approach to rigorously quantify the association between cross disease DGCNs. Towards this goal, for the ith GWAS gene, we computed Kendall's tau-b correlation metric (τ_b) between $g_{ct,i}^{AD}$ and $g_{ct,i}^{PD}$ giving us differential co-expression correlation matrix $G_i \in R^{6 \times 9}$. Each of these matrices encoded a similarity (-1 to +1; 0 being no association) between AD and PD co-expression networks for one gene.

To congregate this cross-association information encoded by 164 GWAS risk genes, we vertically stacked the unraveled matrix G_i (unraveled to $g_i \in R^{1 \times 54}$) into a matrix $P \in R^{164 \times 54}$. Thus, P, in essence, captured multiple modes of information condensed into one matrix: (i) differential gene co-expression between disease and control, (ii) quantified similarity of the expression changes between AD and PD stratified at the level of cell types. Note that the GWAS genes acted as seeds not only for the disease in which they were identified but also for other disease. We finally distilled P using PCA. This uncovered linear combinations of cross-disease cell type-pairs that were most related in terms of their alterations to transcription in response to disease.

The number of significant latent factors that captured biologically meaningful information was determined using a principled permutation testing framework. In 100 permutation iterations, we randomly shuffled the unraveled correlation vector g_i, individually for each i, thus breaking the inherent meaningful patterns of covariation across cell type pairs. Across these 100 iterations, we fitted individual PCA models and computed the explained variances of the derived components. After comparing the permutation variances with our empirical component variances, we retained 4 latent factors as statistically significant based on the 5/95% CI (Fig. S8B). These four embeddings were by construction uncorrelated and rank-ordered, with the first component capturing the highest amount of variance in P.

We conducted a bootstrap analysis on the extracted latent embeddings to formally assess the robustness of the cell type pairs that emerged as being closely associated with each other. Across 1000 bootstrap iterations, we sampled different rows (encapsulating all pairwise cell type co-deviations for a GWAS gene) with replacement to simulate a random seed gene collection that could have been sampled from the empirical population. We fitted individual PCA models to each of the thus derived samples.

To handle the inherent order invariance (changed sequence, especially for later components with small explained variance) and reflection invariance (sign flipping of derived singular vectors) of PCA components [164], we applied the Jonker-Volgenant algorithm for component matching and Pearson's correlation (ρ) for sign matching. The Jonker-Volgenant algorithm is a widely used technique[165] that can identify a one-to-one mapping of latent embeddings derived from two separate bootstrap iterations. The similarity between a pair of components from two runs was scored using the cosine similarity (cf. above). Subsequently, we solved the optimization problem to maximize the similarity between component orderings from two runs across all pairwise combinations of the first 10 empirical and BS-derived PCA components (*scipy.optimize.linear_sum_assignment*, maximize = True). To align directionality, ρ was computed between the empirical PCA component loadings and the BS-component loadings. For cases where ρ was less than 1, the latent vector loadings were multiplied by -1.

Thus, in a complementary data-driven approach to our latent factor modeling, we identified potential combinations of cell types that had the closest associations of gene expression changes between disease and control states in AD and PD.

Data Availability

The snRNA-seq PFC data originated from Mathys, H. et al.[36], are available through Synapse under the doi 10.7303/syn184851755. The data is available under controlled use conditions set by human privacy regulations. The snRNA-seq MTG data originating from Gabitto, M. I. et al.[37], is available through SEA-AD consortium's web portal at SEA-AD.org. The snRNA-seq substantia nigra data originating from Kamath, T. et al. [40], is available at the Gene Expression Omnibus (GEO) with accession number GSE178265. The snRNA-seq midbrain data originating from Smajić, S. et al. [35] is available for download from the Gene Expression Omnibus (GEO) with accession number GSE157783. The snRNA-seq COPD dataset originating from Adams, T. et al. [128], is available for download from the Gene Expression Omnibus (GEO) with accession number GSE136831.

All custom analysis code is available on GitHub at https://github.com/dblabs-mcgill-mila/AD-PD-overlap-study. DOME-ML (Data, Optimization, Model and Evaluation in Machine Learning) annotations are available via the DOME registry (accession v09w7a6a5r) [166]. All additional supporting data are available in the GigaScience repository, GigaDB [167].

Acknowledgements

ROSMAP is supported by P30AG10161, P30AG72975, R01AG15819, R01AG17917, U01AG46152, and U01AG61356. ROSMAP resources can be requested at https://www.radc.rush.edu. DB was supported by the Brain Canada Foundation, through the Canada Brain Research Fund, with the financial support of Health Canada, National Institutes of Health (NIH R01 AG068563A, NIH R01 DA053301-01A1, NIH R01 MH129858-01A1), the Canadian Institute of Health Research (CIHR 438531, CIHR 470425), the Healthy Brains Healthy Lives initiative (Canada First Research Excellence fund), the IVADO R3AI initiative (Canada First Research Excellence fund), and by the CIFAR Artificial Intelligence Chairs program (Canada Institute for Advanced Research).

Author contributions

AB and DB conceptualized the project, planned the experiments and analyzed the results. All authors helped write the manuscript and analyze the results. DB led data analysis.

Ethics declarations

Competing interests

D.B. is an equity holder at MindState Design Labs, USA. The authors declare no other competing interests.

References

1. Bloem BR, Okun MS, Klein C. Parkinson's disease. The Lancet. 2021;397(10291):2284-2303. https://doi.org/10.1016/S0140-6736(21)00218-X

2. Gustavsson A, Norton N, Fast T, et al. Global estimates on the number of persons across the Alzheimer's disease continuum. Alzheimer's & Dementia. 2023;19(2):658-670. https://doi.org/10.1002/alz.12694

3. Melnikova I. Therapies for Alzheimer's disease. Nature Reviews Drug Discovery. 2007;6(5):341-342. https://doi.org/10.1038/nrd2314

4. Kamath T, Macosko EZ. Insights into Neurodegeneration in Parkinson's Disease from Single-Cell and Spatial Genomics. Movement Disorders. 2023;38(4):518-525. https://doi.org/10.1002/mds.29374

5. Albers MW, Gilmore GC, Kaye J, et al. At the interface of sensory and motor dysfunctions and Alzheimer's disease. Alzheimer's & Dementia. 2015;11(1):70-98.

6. Oldham MC, Konopka G, Iwamoto K, et al. Functional organization of the transcriptome in human brain. Nat Neurosci. 2008;11(11):1271-1282. https://doi.org/10.1038/nn.2207

7. Twohig D, Nielsen HM. α-synuclein in the pathophysiology of Alzheimer's disease. Molecular Neurodegeneration. 2019;14(1):23. https://doi.org/10.1186/s13024-019-0320-x

8. Aarsland D, Batzu L, Halliday GM, et al. Parkinson disease-associated cognitive impairment. Nat Rev Dis Primers. 2021;7(1):1. https://doi.org/10.1038/s41572-021-00280-3

9. Cummings J, Lee G, Ritter A, Sabbagh M, Zhong K. Alzheimer's disease drug development pipeline: 2020. Alzheimer's & Dementia: Translational Research & Clinical Interventions. 2020;6(1):e12050. https://doi.org/10.1002/trc2.12050

10. THE BRAINSTORM CONSORTIUM, Anttila V, Bulik-Sullivan B, et al. Analysis of shared heritability in common disorders of the brain. Science. 2018;360(6395):eaap8757. https://doi.org/10.1126/science.aap8757

11. Wightman DP, Savage JE, Tissink E, Romero C, Jansen IE, Posthuma D. The genetic overlap between Alzheimer's disease, amyotrophic lateral sclerosis, Lewy body dementia, and Parkinson's disease. Neurobiology of Aging. 2023;127:99-112. https://doi.org/10.1016/j.neurobiolaging.2023.03.004

12. Sriram Balusu, Praschberger R, Lauwers E. Neurodegeneration cell per cell. Neuron. 2023;111(6):767-786. https://doi.org/10.1016/j.neuron.2023.01.016

13. Zhang X, Gao F, Wang D, et al. Tau Pathology in Parkinson's Disease. Front Neurol. 2018;9:809. https://doi.org/10.3389/fneur.2018.00809

14. Aarsland D, Kurz MW. The epidemiology of dementia associated with Parkinson disease. J Neurol Sci. 2010;289(1-2):18-22. https://doi.org/10.1016/j.jns.2009.08.034

15. Schneider JA, Arvanitakis Z, Leurgans SE, Bennett DA. The Neuropathology of Probable Alzheimer's Disease and Mild Cognitive Impairment. Ann Neurol. 2009;66(2):200-208. https://doi.org/10.1002/ana.21706

16. Schneider JA, Arvanitakis Z, Yu L, Boyle PA, Leurgans SE, Bennett DA. Cognitive impairment, decline and fluctuations in older community-dwelling subjects with Lewy bodies. Brain. 2012;135(10):3005-3014. https://doi.org/10.1093/brain/aws234

17. Schneider JA, Li JL, Li Y, Wilson RS, Kordower JH, Bennett DA. Substantia nigra tangles are related to gait impairment in older persons. Annals of Neurology. 2006;59(1):166-173. https://doi.org/10.1002/ana.20723

18. Armstrong RA, Lantos PL, Cairns NJ. Overlap between neurodegenerative disorders. Neuropathology. 2005;25(2):111-124. https://doi.org/10.1111/j.1440-1789.2005.00605.x

19. Perl DP, Warren CO, Calne D. Alzheimer's disease and parkinson's disease: Distinct entities or extremes of a spectrum of neurodegeneration? Annals of Neurology. 1998;44(S1):S19-S31. https://doi.org/10.1002/ana.410440705

20. Wu Y, Sun R, Ren S, Zengin G, Li M. Neuronal Reshaping of the Tumor Microenvironment in Tumorigenesis and Metastasis: Bench to Clinic. Medicine Advances. 2025;3(4):364-371. https://doi.org/10.1002/med4.70044

21. Lei HY, Pi GL, He T, et al. Targeting vulnerable microcircuits in the ventral hippocampus of male transgenic mice to rescue Alzheimer-like social memory loss. Military Med Res. 2024;11(1):16. https://doi.org/10.1186/s40779-024-00512-z

22. Desikan RS, Schork AJ, Wang Y, et al. Genetic overlap between Alzheimer's disease and Parkinson's disease at the MAPT locus. Mol Psychiatry. 2015;20(12):1588-1595. https://doi.org/10.1038/mp.2015.6

23. Sadeghi I, Gispert JD, Palumbo E, et al. Brain transcriptomic profiling reveals common alterations across neurodegenerative and psychiatric disorders. Computational and Structural Biotechnology Journal. 2022;20:4549-4561. https://doi.org/10.1016/j.csbj.2022.08.037

24. Wingo TS, Liu Y, Gerasimov ES, et al. Shared mechanisms across the major psychiatric and neurodegenerative diseases. Nat Commun. 2022;13(1):1. https://doi.org/10.1038/s41467-022-31873-5

25. Le Bars S, Glaab E. Single-Cell Cortical Transcriptomics Reveals Common and Distinct Changes in Cell-Cell Communication in Alzheimer's and Parkinson's Disease. Mol Neurobiol. 2025;62(3):2655-2673. https://doi.org/10.1007/s12035-024-04419-7

26. Barabási AL, Gulbahce N, Loscalzo J. Network medicine: a network-based approach to human disease. Nat Rev Genet. 2011;12(1):56-68. https://doi.org/10.1038/nrg2918

27. Crow M, Gillis J. Co-expression in Single-Cell Analysis: Saving Grace or Original Sin? Trends in Genetics. 2018;34(11):823-831. https://doi.org/10.1016/j.tig.2018.07.007

28. Subramanian A, Tamayo P, Mootha VK, et al. Gene set enrichment analysis: A knowledge-based approach for interpreting genome-wide expression profiles. Proceedings of the National Academy of Sciences. 2005;102(43):15545-15550. https://doi.org/10.1073/pnas.0506580102

29. Zhang B, Horvath S. A General Framework for Weighted Gene Co-Expression Network Analysis. Statistical Applications in Genetics and Molecular Biology. 2005;4(1). https://doi.org/10.2202/1544-6115.1128

30. Gerstein MB, Kundaje A, Hariharan M, et al. Architecture of the human regulatory network derived from ENCODE data. Nature. 2012;489(7414):91-100. https://doi.org/10.1038/nature11245

31. Saelens W, Cannoodt R, Saeys Y. A comprehensive evaluation of module detection methods for gene expression data. Nat Commun. 2018;9(1):1090. https://doi.org/10.1038/s41467-018-03424-4

32. Zhao Y, Jia M, Ding C, et al. Time-restricted feeding mitigates Alzheimer's disease-associated cognitive impairments via a B. pseudolongum-propionic acid-FFAR3 axis. iMeta. 2025;4(2):e70006. https://doi.org/10.1002/imt2.70006

33. Xiong X, James BT, Boix CA, et al. Epigenomic dissection of Alzheimer's disease pinpoints causal variants and reveals epigenome erosion. Cell. 2023;186(20):4422-4437.e21. https://doi.org/10.1016/j.cell.2023.08.040

34. Mathys H, Peng Z, Boix CA, et al. Single-cell atlas reveals correlates of high cognitive function, dementia, and resilience to Alzheimer's disease pathology. Cell. 2023;186(20):4365-4385.e27. https://doi.org/10.1016/j.cell.2023.08.039

35. Smajić S, Prada-Medina CA, Landoulsi Z, et al. Single-cell sequencing of human midbrain reveals glial activation and a Parkinson-specific neuronal state. Brain. 2022;145(3):964-978. https://doi.org/10.1093/brain/awab446

36. Mathys H, Davila-Velderrain J, Peng Z, et al. Single-cell transcriptomic analysis of Alzheimer's disease. Nature. 2019;570(7761):332-337. https://doi.org/10.1038/s41586-019-1195-2

37. Gabitto MI, Travaglini KJ, Rachleff VM, et al. Integrated multimodal cell atlas of Alzheimer’s disease. Nat Neurosci. Published online October 14, 2024:1-18. https://doi.org/10.1038/s41593-024-01774-5

38. Pak V, Adewale Q, Bzdok D, Dadar M, Zeighami Y, Iturria-Medina Y. Distinctive whole-brain cell types predict tissue damage patterns in thirteen neurodegenerative conditions. Fornito A, Wong ML, eds. eLife. 2024;12:RP89368. https://doi.org/10.7554/eLife.89368

39. Ali M, Timsina J, Xu Y, et al. Large-scale CSF and plasma proteomics reveal immune, synaptic, and extracellular matrix disruptions across neurodegenerative diseases. Neuron. 2026;0(0). https://doi.org/10.1016/j.neuron.2026.02.035

40. Kamath T, Abdulraouf A, Burris SJ, et al. Single-cell genomic profiling of human dopamine neurons identifies a population that selectively degenerates in Parkinson’s disease. Nat Neurosci. 2022;25(5):588-595. https://doi.org/10.1038/s41593-022-01061-1

41. Bzdok D, Ioannidis JPA. Exploration, Inference, and Prediction in Neuroscience and Biomedicine. Trends in Neurosciences. 2019;42(4):251-262. https://doi.org/10.1016/j.tins.2019.02.001

42. Hodgson L, Li Y, Iturria-Medina Y, et al. Supervised latent factor modeling isolates cell-type-specific transcriptomic modules that underlie Alzheimer’s disease progression. Commun Biol. 2024;7(1):1-19. https://doi.org/10.1038/s42003-024-06273-8

43. Zhu B, Park JM, Coffey S, et al. Single-cell transcriptomic and proteomic analysis of Parkinson’s disease Brains. bioRxiv. Preprint posted online February 14, 2022:2022.02.14.480397. https://doi.org/10.1101/2022.02.14.480397

44. Bellenguez C, Küçükali F, Jansen IE, et al. New insights into the genetic etiology of Alzheimer’s disease and related dementias. Nat Genet. 2022;54(4):4. https://doi.org/10.1038/s41588-022-01024-z

45. Nalls MA, Blauwendraat C, Vallerga CL, et al. Identification of novel risk loci, causal insights, and heritable risk for Parkinson’s disease: a meta-genome wide association study. Lancet Neurol. 2019;18(12):1091-1102. https://doi.org/10.1016/S1474-4422(19)30320-5

46. Billingsley KJ, Bandres-Ciga S, Saez-Atienzar S, Singleton AB. Genetic risk factors in Parkinson’s disease. Cell Tissue Res. 2018;373(1):9-20. https://doi.org/10.1007/s00441-018-2817-y

47. Reynolds RH, Botía J, Nalls MA, Hardy J, Gagliano Taliun SA, Ryten M. Moving beyond neurons: the role of cell type-specific gene regulation in Parkinson’s disease heritability. npj Parkinsons Dis. 2019;5(1):1-14. https://doi.org/10.1038/s41531-019-0076-6

48. Langfelder P, Horvath S. WGCNA: an R package for weighted correlation network analysis. BMC Bioinformatics. 2008;9(1):559. https://doi.org/10.1186/1471-2105-9-559

49. Roy S, Lagree S, Hou Z, Thomson JA, Stewart R, Gasch AP. Integrated Module and Gene-Specific Regulatory Inference Implicates Upstream Signaling Networks. PLOS Computational Biology. 2013;9(10):e1003252. https://doi.org/10.1371/journal.pcbi.1003252

50. Watson M. CoXpress: differential co-expression in gene expression data. BMC Bioinformatics. 2006;7(1):509. https://doi.org/10.1186/1471-2105-7-509

51. Szwedo AA, Dalen I, Pedersen KF, et al. and Impact Cognitive Decline in Parkinson’s Disease: A 10-Year Population-Based Study. Movement Disorders. 2022;37(5):1016-1027. https://doi.org/10.1002/mds.28932

52. Zenuni H, Bovenzi R, Bissacco J, et al. Clinical and neurochemical correlates of the APOE genotype in early-stage Parkinson’s disease. Neurobiology of Aging. 2023;131:24-28. https://doi.org/10.1016/j.neurobiolaging.2023.07.011

53. Davis AA, Inman CE, Wargel ZM, et al. APOE genotype regulates pathology and disease progression in synucleinopathy. Science Translational Medicine. 2020;12(529):eaay3069. https://doi.org/10.1126/scitranslmed.aay3069

54. Krüger R, Kuhn W, Müller T, et al. AlaSOPro mutation in the gene encoding α-synuclein in Parkinson’s disease. Nat Genet. 1998;18(2):106-108. https://doi.org/10.1038/ng0298-106

55. Devine MJ, Gwinn K, Singleton A, Hardy J. Parkinson’s disease and α-synuclein expression. Movement Disorders. 2011;26(12):2160-2168. https://doi.org/10.1002/mds.23948

56. Khan SS, LaCroix M, Boyle G, et al. Bidirectional modulation of Alzheimer phenotype by alpha-synuclein in mice and primary neurons. Acta Neuropathol. 2018;136(4):589-605. https://doi.org/10.1007/s00401-018-1886-z

57. Larson ME, Sherman MA, Greimel S, et al. Soluble α-Synuclein Is a Novel Modulator of Alzheimer’s Disease Pathophysiology. J Neurosci. 2012;32(30):10253-10266. https://doi.org/10.1523/JNEUROSCI.0581-12.2012

58. Gan L, Cookson MR, Petrucelli L, La Spada AR. Converging pathways in neurodegeneration, from genetics to mechanisms. Nat Neurosci. 2018;21(10):10. https://doi.org/10.1038/s41593-018-0237-7

59. Gao FB, Richter JD, Cleveland DW. Rethinking Unconventional Translation in Neurodegeneration. Cell. 2017;171(5):994-1000. https://doi.org/10.1016/j.cell.2017.10.042

60. Bence NF, Sampat RM, Kopito RR. Impairment of the Ubiquitin-Proteasome System by Protein Aggregation. Science. 2001;292(5521):1552-1555. https://doi.org/10.1126/science.292.5521.1552

61. Qadir A, Kumar A, Nagpal R, Khan A, Wahi A, Jain P. Understanding the Ubiquitin Proteasome System: History and Revolution. In: Nandave M, Jain P, eds. PROTAC-

Mediated Protein Degradation: A Paradigm Shift in Cancer Therapeutics. Springer Nature; 2024:1-20. https://doi.org/10.1007/978-981-97-5077-1_1

62. Tai HC, Schuman EM. Ubiquitin, the proteasome and protein degradation in neuronal function and dysfunction. Nat Rev Neurosci. 2008;9(11):826-838. https://doi.org/10.1038/nrn2499

63. Millecamps S, Julien JP. Axonal transport deficits and neurodegenerative diseases. Nat Rev Neurosci. 2013;14(3):161-176. https://doi.org/10.1038/nrn3380

64. Andreu-Carbó M, Egoldt C, Velluz MC, Aumeier C. Microtubule damage shapes the acetylation gradient. Nat Commun. 2024;15(1):2029. https://doi.org/10.1038/s41467-024-46379-5

65. Naren P, Samim KS, Tryphena KP, et al. Microtubule acetylation dyshomeostasis in Parkinson’s disease. Translational Neurodegeneration. 2023;12(1):20. https://doi.org/10.1186/s40035-023-00354-0

66. Guedes-Dias P, Holzbaur ELF. Axonal transport: Driving synaptic function. Science. 2019;366(6462):eaaw9997. https://doi.org/10.1126/science.aaw9997

67. Lin MT, Beal MF. Mitochondrial dysfunction and oxidative stress in neurodegenerative diseases. Nature. 2006;443(7113):787-795. https://doi.org/10.1038/nature05292

68. Quntanilla RA, Tapia-Monsalves C. The Role of Mitochondrial Impairment in Alzheimer´s Disease Neurodegeneration: The Tau Connection. Curr Neuropharmacol. 2020;18(11):1076-1091. https://doi.org/10.2174/1570159X18666200525020259

69. Pellegrini L, Wetzel A, Grannó S, Heaton G, Harvey K. Back to the tubule: microtubule dynamics in Parkinson’s disease. Cell Mol Life Sci. 2017;74(3):409-434. https://doi.org/10.1007/s00018-016-2351-6

70. Esteves AR, Arduino DM, Swerdlow RH, Oliveira C, Cardoso SM. Microtubule depolymerization potentiates alpha-synuclein oligomerization. Front Aging Neurosci. 2010;1. https://doi.org/10.3389/neuro.24.005.2009

71. Jaunmuktane Z, Brandner S. Invited Review: The role of prion-like mechanisms in neurodegenerative diseases. Neuropathology and Applied Neurobiology. 2020;46(6):522-545. https://doi.org/10.1111/nan.12592

72. Walker LC, Jucker M. The prion principle and Alzheimer’s disease. Science. 2024;385(6715):1278-1279. https://doi.org/10.1126/science.adq5252

73. Diamond MI. Travels with tau prions. Cytoskeleton. 2024;81(1):83-88. https://doi.org/10.1002/cm.21806

74. Kaufman SK, Sanders DW, Thomas TL, et al. Tau Prion Strains Dictate Patterns of Cell Pathology, Progression Rate, and Regional Vulnerability In Vivo. Neuron. 2016;92(4):796-812. https://doi.org/10.1016/j.neuron.2016.09.055

75. Rauch JN, Olson SH, Gestwicki JE. Interactions between Microtubule-Associated Protein Tau (MAPT) and Small Molecules. Cold Spring Harb Perspect Med. 2017;7(7):a024034. https://doi.org/10.1101/cshperspect.a024034

76. Camilleri A, Zarb C, Caruana M, et al. Mitochondrial membrane permeabilisation by amyloid aggregates and protection by polyphenols. Biochim Biophys Acta. 2013;1828(11):2532-2543. https://doi.org/10.1016/j.bbamem.2013.06.026

77. Cui J, Zhao S, Li Y, et al. Regulated cell death: discovery, features and implications for neurodegenerative diseases. Cell Communication and Signaling. 2021;19(1):120. https://doi.org/10.1186/s12964-021-00799-8

78. Datta SR, Dudek H, Tao X, et al. Akt phosphorylation of BAD couples survival signals to the cell-intrinsic death machinery. Cell. 1997;91(2):231-241. https://doi.org/10.1016/s0092-8674(00)80405-5

79. Erekat NS. Apoptosis and its Role in Parkinson's Disease. In: Stoker TB, Greenland JC, eds. Parkinson's Disease: Pathogenesis and Clinical Aspects. Codon Publications; 2018. Accessed August 1, 2024. http://www.ncbi.nlm.nih.gov/books/NBK536724/

80. Mochizuki H, Goto K, Mori H, Mizuno Y. Histochemical detection of apoptosis in Parkinson's disease. J Neurol Sci. 1996;137(2):120-123. https://doi.org/10.1016/0022-510x(95)00336-z

81. Kumar A, Ganini D, Mason RP. Role of cytochrome c in α-synuclein radical formation: implications of α-synuclein in neuronal death in Maneb- and paraquat-induced model of Parkinson's disease. Molecular Neurodegeneration. 2016;11(1):70. https://doi.org/10.1186/s13024-016-0135-y

82. McKenzie AT, Moyon S, Wang M, et al. Multiscale network modeling of oligodendrocytes reveals molecular components of myelin dysregulation in Alzheimer's disease. Mol Neurodegener. 2017;12:82. https://doi.org/10.1186/s13024-017-0219-3

83. Agarwal D, Sandor C, Volpato V, et al. A single-cell atlas of the human substantia nigra reveals cell-specific pathways associated with neurological disorders. Nat Commun. 2020;11:4183. https://doi.org/10.1038/s41467-020-17876-0

84. Bartzokis G. Age-related myelin breakdown: a developmental model of cognitive decline and Alzheimer's disease. Neurobiol Aging. 2004;25(1):5-18; author reply 49-62. https://doi.org/10.1016/j.neurobiolaging.2003.03.001

85. Depp C, Sun T, Sasmita AO, et al. Myelin dysfunction drives amyloid-β deposition in models of Alzheimer's disease. Nature. 2023;618(7964):349-357. https://doi.org/10.1038/s41586-023-06120-6

86. Kenigsbuch M, Bost P, Halevi S, et al. A shared disease-associated oligodendrocyte signature among multiple CNS pathologies. Nat Neurosci. 2022;25(7):876-886. https://doi.org/10.1038/s41593-022-01104-7

87. Zhou Y, Song WM, Andhey PS, et al. Human and mouse single-nucleus transcriptomics reveal TREM2-dependent and TREM2-independent cellular responses in Alzheimer's disease. Nat Med. 2020;26(1):131-142. https://doi.org/10.1038/s41591-019-0695-9

88. Balali-Mood M, Naseri K, Tahergorabi Z, Khazdair MR, Sadeghi M. Toxic Mechanisms of Five Heavy Metals: Mercury, Lead, Chromium, Cadmium, and Arsenic. Front Pharmacol. 2021;12. https://doi.org/10.3389/fphar.2021.643972

89. Haidar Z, Fatema K, Shoily SS, Sajib AA. Disease-associated metabolic pathways affected by heavy metals and metalloid. Toxicol Rep. 2023;10:554-570. https://doi.org/10.1016/j.toxrep.2023.04.010

90. Li B, Xia M, Zorec R, Parpura V, Verkhratsky A. Astrocytes in heavy metal neurotoxicity and neurodegeneration. Brain Res. 2021;1752:147234. https://doi.org/10.1016/j.brainres.2020.147234

91. Huiliang Z, Mengzhe Y, Xiaochuan W, et al. Zinc induces reactive astrogliosis through ERK-dependent activation of Stat3 and promotes synaptic degeneration. Journal of Neurochemistry. 2021;159(6):1016-1027. https://doi.org/10.1111/jnc.15531

92. Gamez P, Caballero AB. Copper in Alzheimer's disease: Implications in amyloid aggregation and neurotoxicity. AIP Advances. 2015;5(9):092503. https://doi.org/10.1063/1.4921314

93. Pal A, Rani I, Pawar A, Picozza M, Rongioletti M, Squitti R. Microglia and Astrocytes in Alzheimer's Disease in the Context of the Aberrant Copper Homeostasis Hypothesis. Biomolecules. 2021;11(11):1598. https://doi.org/10.3390/biom11111598

94. Zhou Q, Zhang Y, Lu L, et al. Copper induces microglia-mediated neuroinflammation through ROS/NF-κB pathway and mitophagy disorder. Food Chem Toxicol. 2022;168:113369. https://doi.org/10.1016/j.fct.2022.113369

95. Uversky VN, Li J, Fink AL. Metal-triggered structural transformations, aggregation, and fibrillation of human alpha-synuclein. A possible molecular NK between Parkinson's disease and heavy metal exposure. J Biol Chem. 2001;276(47):44284-44296. https://doi.org/10.1074/jbc.M105343200

96. Sarell CJ, Wilkinson SR, Viles JH. Substoichiometric Levels of Cu2+ Ions Accelerate the Kinetics of Fiber Formation and Promote Cell Toxicity of Amyloid-β from Alzheimer

Disease *. Journal of Biological Chemistry. 2010;285(53):41533-41540. https://doi.org/10.1074/jbc.M110.171355

97. Myhre O, Utkilen H, Duale N, Brunborg G, Hofer T. Metal Dyshomeostasis and Inflammation in Alzheimer's and Parkinson's Diseases: Possible Impact of Environmental Exposures. Oxid Med Cell Longev. 2013;2013:726954. https://doi.org/10.1155/2013/726954

98. Acosta-Cabronero J, Betts MJ, Cardenas-Blanco A, Yang S, Nestor PJ. In Vivo MRI Mapping of Brain Iron Deposition across the Adult Lifespan. J Neurosci. 2016;36(2):364-374. https://doi.org/10.1523/JNEUROSCI.1907-15.2016

99. Belaidi AA, Bush AI. Iron neurochemistry in Alzheimer's disease and Parkinson's disease: targets for therapeutics. Journal of Neurochemistry. 2016;139(S1):179-197. https://doi.org/10.1111/jnc.13425

100. Bjørklund G, Hofer T, Nurchi VM, Aaseth J. Iron and other metals in the pathogenesis of Parkinson's disease: Toxic effects and possible detoxification. J Inorg Biochem. 2019;199:110717. https://doi.org/10.1016/j.jinorgbio.2019.110717

101. Thomas GEC, Leyland LA, Schrag AE, Lees AJ, Acosta-Cabronero J, Weil RS. Brain iron deposition is linked with cognitive severity in Parkinson's disease. J Neurol Neurosurg Psychiatry. 2020;91(4):418-425. https://doi.org/10.1136/jnnp-2019-322042

102. Ward RJ, Zucca FA, Duyn JH, Crichton RR, Zecca L. The role of iron in brain ageing and neurodegenerative disorders. Lancet Neurol. 2014;13(10):1045-1060. https://doi.org/10.1016/S1474-4422(14)70117-6

103. Lenz KM, Nelson LH. Microglia and Beyond: Innate Immune Cells As Regulators of Brain Development and Behavioral Function. Front Immunol. 2018;9:698. https://doi.org/10.3389/fimmu.2018.00698

104. Tansey MG, Wallings RL, Houser MC, Herrick MK, Keating CE, Joers V. Inflammation and immune dysfunction in Parkinson disease. Nat Rev Immunol. 2022;22(11):657-673. https://doi.org/10.1038/s41577-022-00684-6

105. Khoury JE, Luster AD. Mechanisms of microglia accumulation in Alzheimer's disease: therapeutic implications. Trends in Pharmacological Sciences. 2008;29(12):626-632. https://doi.org/10.1016/j.tips.2008.08.004

106. Chen X, Firulyova M, Manis M, et al. Microglia-mediated T cell Infiltration Drives Neurodegeneration in Tauopathy. Nature. 2023;615(7953):668-677. https://doi.org/10.1038/s41586-023-05788-0

107. González H, Pacheco R. T-cell-mediated regulation of neuroinflammation involved in neurodegenerative diseases. Journal of Neuroinflammation. 2014;11(1):201. https://doi.org/10.1186/s12974-014-0201-8

108. Xu Y, Li Y, Wang C, et al. The reciprocal interactions between microglia and T cells in Parkinson's disease: a double-edged sword. Journal of Neuroinflammation. 2023;20(1):33. https://doi.org/10.1186/s12974-023-02723-y

109. Villanueva I, Alva-Sánchez C, Pacheco-Rosado J. The role of thyroid hormones as inductors of oxidative stress and neurodegeneration. Oxid Med Cell Longev. 2013;2013:218145. https://doi.org/10.1155/2013/218145

110. Ewins DL, Rossor MN, Butler J, Rogues PK, Mullen MJ, McGregor AM. Association between autoimmune thyroid disease and Familial Alzheimers disease. Clinical Endocrinology. 1991;35(1):93-96. https://doi.org/10.1111/j.1365-2265.1991.tb03502.x

111. Kalmijn S, Mehta KM, Pols HA, Hofman A, Drexhage HA, Breteler MM. Subclinical hyperthyroidism and the risk of dementia. The Rotterdam study. Clin Endocrinol (Oxf). 2000;53(6):733-737. https://doi.org/10.1046/j.1365-2265.2000.01146.x

112. Mohammadi S, Dolatshahi M, Rahmani F. Shedding light on thyroid hormone disorders and Parkinson disease pathology: mechanisms and risk factors. J Endocrinol Invest. 2021;44(1):1-13. https://doi.org/10.1007/s40618-020-01314-5

113. Kim DK, Choi H, Lee W, et al. Brain hypothyroidism silences the immune response of microglia in Alzheimer's disease animal model. Sci Adv. 10(11):eadi1863. https://doi.org/10.1126/sciadv.adi1863

114. Seo BA, Kim D, Hwang H, et al. TRIP12 ubiquitination of glucocerebrosidase contributes to neurodegeneration in Parkinson's disease. Neuron. 2021;109(23):3758-3774.e11. https://doi.org/10.1016/j.neuron.2021.09.031

115. Hong S, Beja-Glasser VF, Nfonoyim BM, et al. Complement and microglia mediate early synapse loss in Alzheimer mouse models. Science. 2016;352(6286):712-716. https://doi.org/10.1126/science.aad8373

116. Song P, Peng W, Sauve V, et al. Parkinson's disease-linked parkin mutation disrupts recycling of synaptic vesicles in human dopaminergic neurons. Neuron. 2023;111(23):3775-3788.e7. https://doi.org/10.1016/j.neuron.2023.08.018

117. Tzioras M, Daniels MJD, Davies C, et al. Human astrocytes and microglia show augmented ingestion of synapses in Alzheimer's disease via MFG-E8. CR Med. 2023;4(9). https://doi.org/10.1016/j.xcrm.2023.101175

118. Das M, Mao W, Voskobiynyk Y, et al. Alzheimer risk-increasing TREM2 variant causes aberrant cortical synapse density and promotes network hyperexcitability in mouse models. Neurobiol Dis. 2023;186:106263. https://doi.org/10.1016/j.nbd.2023.106263

119. Filipello F, Morini R, Corradini I, et al. The Microglial Innate Immune Receptor TREM2 Is Required for Synapse Elimination and Normal Brain Connectivity. Immunity. 2018;48(5):979-991.e8. https://doi.org/10.1016/j.immuni.2018.04.016

120. Guo Y, Wei X, Yan H, et al. TREM2 deficiency aggravates α-synuclein-induced neurodegeneration and neuroinflammation in Parkinson's disease models. FASEB J. 2019;33(11):12164-12174. https://doi.org/10.1096/fj.201900992R

121. Shafi S, Singh A, Ibrahim AM, Alhajri N, Abu Izneid T, Pottoo FH. Role of triggering receptor expressed on myeloid cells 2 (TREM2) in neurodegenerative dementias. Eur J Neurosci. 2021;53(10):3294-3310. https://doi.org/10.1111/ejn.15215

122. Alexander JJ, Anderson AJ, Barnum SR, Stevens B, Tenner AJ. The complement cascade: Yin–Yang in neuroinflammation – neuro-protection and -degeneration. Journal of Neurochemistry. 2008;107(5):1169-1187. https://doi.org/10.1111/j.1471-4159.2008.05668.x

123. Carbutt S, Duff J, Yarnall A, Burn DJ, Hudson G. Variation in complement protein C1q is not a major contributor to cognitive impairment in Parkinson's disease. Neurosci Lett. 2015;594:66-69. https://doi.org/10.1016/j.neulet.2015.03.048

124. Hallett PJ, Engelender S, Isacson O. Lipid and immune abnormalities causing age-dependent neurodegeneration and Parkinson's disease. Journal of Neuroinflammation. 2019;16(1):153. https://doi.org/10.1186/s12974-019-1532-2

125. Sienski G, Narayan P, Bonner JM, et al. APOE4 disrupts intracellular lipid homeostasis in human iPSC-derived glia. Science Translational Medicine. 2021;13(583):eaaz4564. https://doi.org/10.1126/scitranslmed.aaz4564

126. Hou Y fang, Shan C, Zhuang S yue, et al. Gut microbiota-derived propionate mediates the neuroprotective effect of osteocalcin in a mouse model of Parkinson's disease. Microbiome. 2021;9(1):34. https://doi.org/10.1186/s40168-020-00988-6

127. Bennett DA, Buchman AS, Boyle PA, et al. Religious Orders Study and Rush Memory and Aging Project. Journal of Alzheimer's Disease. 2018;64(s1):S161-S189. https://doi.org/10.3233/JAD-179939

128. Adams TS, Schupp JC, Poli S, et al. Single-cell RNA-seq reveals ectopic and aberrant lung-resident cell populations in idiopathic pulmonary fibrosis. Science Advances. 2020;6(28):eaba1983. https://doi.org/10.1126/sciadv.aba1983

129. Krishnaswami SR, Grindberg RV, Novotny M, et al. Using single nuclei for RNA-seq to capture the transcriptome of postmortem neurons. Nat Protoc. 2016;11(3):499-524. https://doi.org/10.1038/nprot.2016.015

130. Ma P, Liu X, Xu Z, et al. Joint profiling of gene expression and chromatin accessibility during amphioxus development at single-cell resolution. Cell Reports. 2022;39(12). https://doi.org/10.1016/j.celrep.2022.110979

131. Ahlmann-Eltze C, Huber W. Comparison of transformations for single-cell RNA-seq data. Nat Methods. 2023;20(5):665-672. https://doi.org/10.1038/s41592-023-01814-1

132. Zhu Y, Wang L, Yin Y, Yang E. Systematic analysis of gene expression patterns associated with postmortem interval in human tissues. Sci Rep. 2017;7(1):1. https://doi.org/10.1038/s41598-017-05882-0

133. Ferreira PG, Muñoz-Aguirre M, Reverter F, et al. The effects of death and post-mortem cold ischemia on human tissue transcriptomes. Nat Commun. 2018;9(1):1. https://doi.org/10.1038/s41467-017-02772-x

134. Hughes G. On the mean accuracy of statistical pattern recognizers. IEEE Transactions on Information Theory. 1968;14(1):55-63. https://doi.org/10.1109/TIT.1968.1054102

135. Jia W, Sun M, Lian J, Hou S. Feature dimensionality reduction: a review. Complex Intell Syst. 2022;8(3):2663-2693. https://doi.org/10.1007/s40747-021-00637-x

136. Thompson JR, Nelson ED, Tippani M, et al. An integrated single-nucleus and spatial transcriptomics atlas reveals the molecular landscape of the human hippocampus. Nat Neurosci. Published online July 30, 2025:1-15. https://doi.org/10.1038/s41593-025-02022-0

137. Kotliar D, Veres A, Nagy MA, et al. Identifying gene expression programs of cell-type identity and cellular activity with single-cell RNA-Seq. Valencia A, Barkai N, Mereu E, Göttgens B, eds. eLife. 2019;8:e43803. https://doi.org/10.7554/eLife.43803

138. Mathys H, Boix CA, Akay LA, et al. Single-cell multiregion dissection of Alzheimer's disease. Nature. 2024;632(8026):858-868. https://doi.org/10.1038/s41586-024-07606-7

139. Barker M, Rayens W. Partial least squares for discrimination. Journal of Chemometrics. 2003;17(3):166-173. https://doi.org/10.1002/cem.785

140. Chun H, Keleş S. Sparse Partial Least Squares Regression for Simultaneous Dimension Reduction and Variable Selection. Accessed March 30, 2026. https://dx.doi.org/10.1111/j.1467-9868.2009.00723.x

141. Agarwal A, Shah D, Shen D, Song D. On Robustness of Principal Component Regression. arXiv. Preprint posted online May 19, 2021:arXiv:1902.10920. https://doi.org/10.48550/arXiv.1902.10920

142. Luecken MD, Theis FJ. Current best practices in single-cell RNA-seq analysis: a tutorial. Molecular Systems Biology. 2019;15(6):e8746. https://doi.org/10.15252/msb.20188746

143. Lee LC, Liong CY, Jemain AA. Partial least squares-discriminant analysis (PLS-DA) for classification of high-dimensional (HD) data: a review of contemporary practice strategies and knowledge gaps. Analyst. 2018;143(15):3526-3539. https://doi.org/10.1039/C8AN00599K

144. Wold HOA. Nonlinear Iterative Partial Least Squares (NIPALS) Modelling: Some Current Developments. In: 1973. https://api.semanticscholar.org/CorpusID:118962244

145. Rodríguez-Pérez R, Fernández L, Marco S. Overoptimism in cross-validation when using partial least squares-discriminant analysis for omics data: a systematic study. Anal Bioanal Chem. 2018;410(23):5981-5992. https://doi.org/10.1007/s00216-018-1217-1

146. de Boves Harrington P. Statistical validation of classification and calibration models using bootstrapped Latin partitions. TrAC Trends in Analytical Chemistry. 2006;25(11):1112-1124. https://doi.org/10.1016/j.trac.2006.10.010

147. Miller KL, Alfaro-Almagro F, Bangerter NK, et al. Multimodal population brain imaging in the UK Biobank prospective epidemiological study. Nat Neurosci. 2016;19(11):1523-1536. https://doi.org/10.1038/nn.4393

148. Spreng RN, Dimas E, Mwilambwe-Tshilobo L, et al. The default network of the human brain is associated with perceived social isolation. Nat Commun. 2020;11(1):6393. https://doi.org/10.1038/s41467-020-20039-w

149. Moon KR, van Dijk D, Wang Z, et al. Visualizing Structure and Transitions in High-Dimensional Biological Data. Nat Biotechnol. 2019;37(12):1482-1492. https://doi.org/10.1038/s41587-019-0336-3

150. Arndt S, Turvey C, Andreasen NC. Correlating and predicting psychiatric symptom ratings: Spearman's r versus Kendall's tau correlation. J Psychiatr Res. 1999;33(2):97-104. https://doi.org/10.1016/s0022-3956(98)90046-2

151. Benjamini Y, Hochberg Y. Controlling the False Discovery Rate: A Practical and Powerful Approach to Multiple Testing. Journal of the Royal Statistical Society: Series B (Methodological). 1995;57(1):289-300. https://doi.org/10.1111/j.2517-6161.1995.tb02031.x

152. Stark R, Grzelak M, Hadfield J. RNA sequencing: the teenage years. Nat Rev Genet. 2019;20(11):631-656. https://doi.org/10.1038/s41576-019-0150-2

153. Finak G, McDavid A, Yajima M, et al. MAST: a flexible statistical framework for assessing transcriptional changes and characterizing heterogeneity in single-cell RNA sequencing data. Genome Biology. 2015;16(1):278. https://doi.org/10.1186/s13059-015-0844-5

154. Ruxton GD. The unequal variance t-test is an underused alternative to Student's t-test and the Mann–Whitney U test. Behavioral Ecology. 2006;17(4):688-690. https://doi.org/10.1093/beheco/ark016

155. WELCH BL. THE GENERALIZATION OF ‘STUDENT’S’ PROBLEM WHEN SEVERAL DIFFERENT POPULATION VARLANCES ARE INVOLVED. Biometrika. 1947;34(1-2):28-35. https://doi.org/10.1093/biomet/34.1-2.28

156. Fang Z, Liu X, Peltz G. GSEApy: a comprehensive package for performing gene set enrichment analysis in Python. Bioinformatics. 2023;39(1):btac757. https://doi.org/10.1093/bioinformatics/btac757

157. Chen EY, Tan CM, Kou Y, et al. Enrichr: interactive and collaborative HTML5 gene list enrichment analysis tool. BMC Bioinformatics. 2013;14(1):128. https://doi.org/10.1186/1471-2105-14-128

158. Ashburner M, Ball CA, Blake JA, et al. Gene Ontology: tool for the unification of biology. Nat Genet. 2000;25(1):25-29. https://doi.org/10.1038/75556

159. The Gene Ontology Consortium, Aleksander SA, Balhoff J, et al. The Gene Ontology knowledgebase in 2023. Genetics. 2023;224(1):iyad031. https://doi.org/10.1093/genetics/iyad031

160. Carbon S, Ireland A, Mungall CJ, et al. AmiGO: online access to ontology and annotation data. Bioinformatics. 2009;25(2):288-289. https://doi.org/10.1093/bioinformatics/btn615

161. Merico D, Isserlin R, Stueker O, Emili A, Bader GD. Enrichment Map: A Network-Based Method for Gene-Set Enrichment Visualization and Interpretation. PLOS ONE. 2010;5(11):e13984. https://doi.org/10.1371/journal.pone.0013984

162. Reimand J, Isserlin R, Voisin V, et al. Pathway enrichment analysis and visualization of omics data using g:Profiler, GSEA, Cytoscape and EnrichmentMap. Nat Protoc. 2019;14(2):2. https://doi.org/10.1038/s41596-018-0103-9

163. Shannon P, Markiel A, Ozier O, et al. Cytoscape: a software environment for integrated models of biomolecular interaction networks. Genome Res. 2003;13(11):2498-2504. https://doi.org/10.1101/gr.1239303

164. Saltoun K, Adolphs R, Paul LK, et al. Dissociable brain structural asymmetry patterns reveal unique phenome-wide profiles. Nat Hum Behav. 2023;7(2):251-268. https://doi.org/10.1038/s41562-022-01461-0

165. Crouse DF. On implementing 2D rectangular assignment algorithms. IEEE Transactions on Aerospace and Electronic Systems. 2016;52(4):1679-1696. https://doi.org/10.1109/TAES.2016.140952

166. Anwesha Bhattacharya DB Edward A Fon, Alain Dagher, Yasser Iturria Medina, Jo Anne Stratton, Chloe Savignac, Jack Stanley, Liam Hodgson, Badr Ait Hammou, David A Bennett. Cell type transcriptomic modules reveal shared molecular mechanisms in Alzheimer's and

Parkinson's disease. GigaScience. Available from: https://registry.dome-ml.org/review/v09w7a6a5r

167. Bhattacharya A;Fon E A;Dagher A;Itturia-Medina Y;Stratton J A;Savignac C;Stanley J;Hodgson L;Hammou B A;Bennett D A;Bzdok D.(): Cell type transcriptomic modules reveal shared molecular mechanisms in in Alzheimer's and Parkinson's disease.GigaScience database.https://doi.org/10.5524/102822

Figure 1

Click here to access/download
Supplementary Material
Fig1.svg

Figure 2

Click here to access/download
Supplementary Material
Fig2.svg

Figure 3

Click here to access/download
Supplementary Material
Fig3.svg

Figure 4

Click here to access/download
Supplementary Material
Fig4_updated_bigger.svg

Figure 5

Click here to access/download
Supplementary Material
Fig5_updated.svg

Figure 6

Click here to access/download
Supplementary Material
Fig6_DGCN_bs.svg

Supplementary Tables

Click here to access/download
Supplementary Material
Supplementary_table_revision.xlsx

Supplementary Figures

Click here to access/download
Supplementary Material
Supplementary.docx

Reviewer reports:

Reviewer #1:

The authors utilized single-nuclei RNA-seq data and GWAS data to study the disease comorbidity between Alzheimer's disease and Parkinson's disease. They also validated their discoveries using independent evaluation cohorts. This is an overall well-conducted and comprehensive study with a solid logical framework and clear presentation of very interesting biological results. I have few points that need clarifications.

1.In section 'Common Mechanisms of microglia's involvement in AD and PD', lines 790-792, the authors mentioned that 'we noted T cell activation in both AD and PD (all 4 datasets).' This is very interesting biological discovery. Based on my knowledge, the amount of T cells extracted from brain snRNA-seq are quite small (I could be wrong). Can authors explain something that could prove the conclusion is not biased due to the small population of T cells?

Response:

We thank the reviewer for raising this important point. To recap, the reviewer is referring to our mention of “T cell activation” in our discussion of the overlap between AD and PD that emerge in microglia-derived gene modules.

We would like to clarify that the reference to “T cell activation” should not be interpreted as a direct measurement of T cell abundance. Rather, it reflects a pathway enrichment analysis result derived from microglial gene modules with high AD- and PD- predictive weights. Since this pathway was shared in both AD and PD analysis, it was highlighted in our discussion.

Specifically, this enrichment reflects the presence of genes involved in antigen presentation in microglia modules from both AD and PD. As highlighted in our Results section, the gene modules involved were PD Mic 2, End 2, and AD Mic 2. The relevant shared genes included B2M, HLA-A, HLA-B, HLA-C, HLA-DPA1, HLA-DRA, HLA-DRB1, HLA-DRB5, and HLA-E. Therefore, the enrichment of these terms in microglial modules likely reflects microglia-mediated immune signaling relevant to T cell activation, rather than the presence of a large number of T cells in the snRNA-seq dataset itself.

To avoid potential misinterpretation of the original wording by future readers of our work, we have revised the text to clarify that this observation refers to pathway enrichment within microglial gene modules.

The revised Discussion text now reads:

“Functionally, microglia are the primary immune cells of the CNS (Lenz and Nelson 2018), and neuroinflammation and immune system dysfunction are believed to be key components of neurodegeneration (Tansey et al. 2022). Consistently, microglial gene modules from our study showed propensity for several immune-related processes. For example, T cell activation terms were associated with microglial modules for both AD and PD (all 4 datasets). Microglia, upon activation by neuronal stress, are thought to release pro-inflammatory cytokines and upregulate

MHC class I and II molecules (Khoury and Luster 2008). Further, the inflammatory cytokines can induce the expression of adhesion molecules on brain endothelial cells, compromising the integrity of the blood-brain barrier (BBB). This BBB breakdown accelerates peripheral immune cell entry. Thus, in a chicken-egg scenario, microglia and endothelial cells drive a chain reaction of T cell activation, oxidative stress, and neuroinflammation (Chen et al. 2023; González and Pacheco 2014; Xu et al. 2023). This domino effect may have been captured in one of our PD endothelial modules whose pathways analysis featured T cell activation terms as well as association with biological processes like leukocyte adhesion to vascular cells, blood vessel morphogenesis and diameter maintenance, pointing to the dysregulation of the BBB. This cascade of immune response events exacerbates ROS production and neuronal damage (Chen et al. 2023; González and Pacheco 2014)."

2. The authors mentioned using PLS-DA method to extract disease modules for AD and PD datasets respectively. if I understand correctly, that the authors apply PLS-DA method to snRNA-seq data instead of for example pseudobulk aggregated for each cell type. Can the authors explain and add some logics in the method section to further clarify why applying PLS-DA to snRNA-seq make sense? As we know snRNA-seq data are quite sparse.

Response:

We appreciate the reviewer's comment, which provides us with an opportunity to clarify why our latent factor modelling pipeline with PLS-DA is a natural choice of methodology in our setting.

To summarize, our end-to-end analysis workflow (Fig. 1) was applied at the single-nucleus level, such that each nucleus transcriptome served as an individual observation. This allowed our model to capture disease-associated transcriptional signals at the cellular level, including variations across orthogonal gene programs within the same annotated cell type.

Importantly, our approach builds upon prior published work from our group in which PLS-DA was successfully used to characterize gene programs associated with AD (Hodgson et al. 2024). In our study, we have used a similar gene module identification pipeline as described in the previous work. Further, we note that PLS-DA was not directly applied to the sparse gene expression matrix. Instead, it was performed on a PCA-derived lower dimensional latent space, which are denser representations of the data, which means PLS-DA itself did not face the sparsity concern raised by the reviewer.

Indeed, analytical results from machine learning theory have demonstrated that PCA is stable and statistically robust in a variety of data settings. In particular, under noisy and sparse conditions, principal components regression (regression method built on principal components) applied to a feature matrix ($\boldsymbol{X}$) is provably robust (Agarwal et al. 2019). The required criterion for such applications is that $\boldsymbol{X}$ allows a suitable low-rank approximation, which is a common, biologically valid, modelling assumption in single-cell analysis (Crow and Gillis 2018). Thus, a rigorous theoretical foundation supports the application of PCA to sparse/noisy matrices such as the transcriptome matrices in our present study.

Practically, dimensionality reduction methods like PCA, non-negative matrix factorization and related methods, are routinely used to extract gene programs directly from single-cell expression matrices (Kotliar et al. 2019; Mathys et al. 2024; Thompson et al. 2025). In contrast, the pseudobulk approach is a strategy used in many single-cell studies where gene expressions are aggregated across cells within replicates/donors. However, this method collapses the transcriptional richness across individual cells within a given cell type. Moreover, applying pseudobulk aggregation here would result in the number of observations being a handful of datapoints (number of donors, N << number of cells). This would lead to a case where N<<p, where p was the number of features (genes) in our analysis.

As the reviewer noted, the primary risk when applying supervised latent factor pipelines to sparse high-dimensional data is capturing components aligned with noise. To guard against this, we have implemented several layers of model validation to detect spurious structures which might be derived by our overall pipeline. These include nested cross-validations, split-half analyses, and donor stratified permutation tests (Fig. S1). In such cases, comparing results to label-shuffled permutation-derived models is a standard approach to detect such effects, and our analysis indicates that the observed signal exceeds what would be expected under this null scenario (Fig. S1B, S3B).

Moreover, we note that in the comparative analysis between AD and PD, we have focused on genes that were consistently selected across model refits based on bootstrap resamples of the transcriptome dataset. This procedure allowed us to identify genes that were stable across different realizations of the data, further reducing the likelihood that the reported results are driven by sampling noise or unstable features.

We have restructured and added explicit explanation in the Methods section to address the reviewer point on data sparsity and explain the reasoning for applying PLS-DA at the single-nucleus level.

“Our work builds on a prior study in which the multivariate method partial least squares discriminant analysis (PLS-DA) (Barker and Rayens 2003) was employed to derive AD-predictive gene modules across major brain cell types (Hodgson et al. 2024). In contrast to the original study, where the analyzed dataset had favorable observations to features ratio, several datasets analyzed here comprised substantially fewer samples within individual cell types. In these cases, PLS-DA is susceptible to overfitting, particularly given the inherent noise and sparsity of snRNA-seq data (Chun and Keleş 2010). To address this limitation, we applied principal component analysis (PCA) denoising to the input gene expression matrix, prior to fitting our supervised model of scientific interest. Under a low-rank assumption on the input feature space (gene expression), dimensionality reduction with PCA is known to yeild an optimal low-rank approximation which is robust to noise and sparsity (Agarwal et al. 2019; Crow and Gillis 2018). In practice, such transformations are routinely applied in single-cell and single-nucleus analysis, where the ambient gene space is well approximated by a low-rank structure (Luecken and Theis 2019).

Specifically, for each cell type within individual datasets, we applied PCA to the input gene expression matrix. These PCA-derived component projections were then used as input variables

for PLS-DA to obtain a projection maximizing disease versus control class separation. To guard against spurious derived structure (Lee, Liong, and Jemain 2018), we performed strict model validations (see below), including label-shuffled permutation tests to assess the statistical significance of derived components (Fig. S1B, S3B)."

The permutation-based validation result and figure is included below (see Fig. S1).

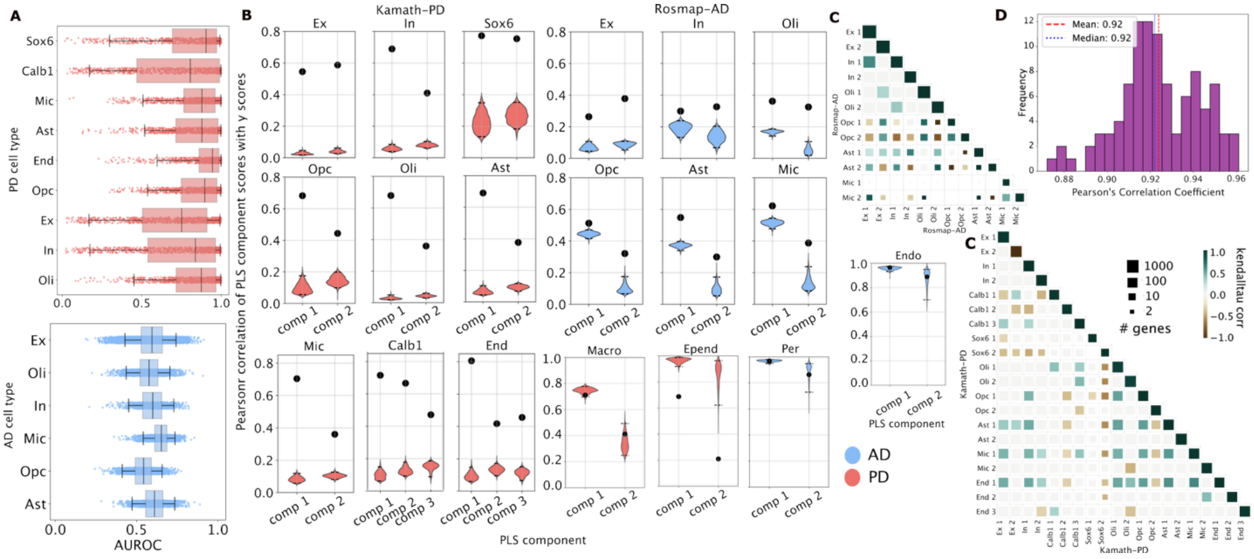

(B) Disease predictive power of PLS components. (Left) Kamath-PD, (right) Rosmap-AD. Component-disease alignment was quantified using Pearson's ρ between component's disease prediction (x-score) and true disease representation (y-score). The black dot represents the model's empirical ρ. Violins depict null distributions of ρ generated from label permutation of the empirical dataset (n=1000). Dashed lines represent 2.5/97.5% CI.

The relevant paragraph in the Results section reads as follows.

"Our thus derived gene modules (PLS_{cell} components) featured a combination of several genes whose co-expression signature was associated with a disease state (AD vs. control or PD vs. control). We assessed the statistical significance of each module by comparing its empirical disease prediction performance to a null distribution of performance metrics derived by a label-shuffling permutation procedure (Methods). Only significant gene modules were considered for subsequent analyses (empirical module ρ > 97.5th percentile of permutation derived ρ distribution; Fig. S1B). In particular, two cell types in Rosmap-AD, pericytes and ependymal cells, did not pass our significance test and were removed from further analysis. Two cell types in Kamath-PD, macrophages and ependymal cells, were similarly removed."

References:

Agarwal, Anish, Devavrat Shah, Dennis Shen, and Dogyoon Song. 2019. “On Robustness of Principal Component Regression.” In Advances in Neural Information Processing Systems, edited by H. Wallach, H. Larochelle, A. Beygelzimer, F. d’Alché-Buc, E. Fox, and R. Garnett, vol. 32. Curran Associates, Inc. https://proceedings.neurips.cc/paper_files/paper/2019/file/923e325e16617477e457f6a468a2d6df-Paper.pdf.

Barker, Matthew, and William Rayens. 2003. “Partial Least Squares for Discrimination.” Journal of Chemometrics 17 (3): 166–73. https://doi.org/10.1002/cem.785.

Chun, Hyonho, and Sündüz Keleş. 2010. “Sparse Partial Least Squares Regression for Simultaneous Dimension Reduction and Variable Selection.” Journal of the Royal Statistical Society Series B: Statistical Methodology 72 (1): 3–25. https://doi.org/10.1111/j.1467-9868.2009.00723.x.

Crow, Megan, and Jesse Gillis. 2018. “Co-Expression in Single-Cell Analysis: Saving Grace or Original Sin?” Trends in Genetics 34 (11): 823–31. https://doi.org/10.1016/j.tig.2018.07.007.

Hodgson, Liam, Yue Li, Yasser Iturria-Medina, et al. 2024. “Supervised Latent Factor Modeling Isolates Cell-Type-Specific Transcriptomic Modules That Underlie Alzheimer’s Disease Progression.” Communications Biology 7 (1): 1–19. https://doi.org/10.1038/s42003-024-06273-8.

Lee, Loong Chuen, Choong-Yeun Liong, and Abdul Aziz Jemain. 2018. “Partial Least Squares-Discriminant Analysis (PLS-DA) for Classification of High-Dimensional (HD) Data: A Review of Contemporary Practice Strategies and Knowledge Gaps.” Analyst 143 (15): 3526–39. https://doi.org/10.1039/C8AN00599K.

Luecken, Malte D., and Fabian J. Theis. 2019. “Current Best Practices in Single-cell RNA-seq Analysis: A Tutorial.” Molecular Systems Biology 15 (6): e8746. https://doi.org/10.15252/msb.20188746.

3. In Figure 4D, authors presented multiple gene modules, can the authors also add gene/protein symbols as well, not just circles? Readers can know directly what are related proteins.

Response:

We thank the reviewer for this helpful suggestion. Accordingly, we have updated Fig. 4C to include the top representative gene symbols, in addition to the graphical representation of the gene ontology (GO) terms. Note that each circle denotes a GO term and not a gene/protein. We agree that the addition of gene symbols will allow readers to more easily situate the key genes within shared pathways between AD and PD.

The update Figure and corresponding caption are as follows.

Figure 1. Gene ontology terms mapped to gene modules are shared between AD and PD. Gene set enrichment analysis results for derived gene modules. For each gene module in AD or PD, we mapped the ranked genes (based on predictive weights) to terms in the gene ontology (GO) database. **(A)** Overlapping terms from GO Biological Process, Cellular Component, Molecular Functions. The Venn diagrams depict the number of unique and shared terms across all gene modules, grouped by AD or PD. **(B)** Zooming in on a few key terms summarizing the most frequent shared terms and gene-module combinations. Solid black dots indicate that a term (in the vertical axis) is enriched in the corresponding gene-module pair (in the horizontal axis). The bar plots on the horizontal axes are counts of the total number of terms common between AD and PD for the gene-module pair (arranged in decreasing order of term counts, first 30 pairs shown). Bar plots on the vertical axis represent the total number of cross-disease gene-module pairs that a term is present in (arranged in decreasing order of gene-module pair counts, first 10 terms shown). **(C)** Graph visualization of select biological processes across AD and PD from GO. Nodes are colored based on disease labels and node size indicates the gene-set size. Group names summarize the main themes from the terms in the group. Top AD-PD shared genes (robust PLS loadings) are annotated within each group. This zoomed-out view highlights key biological processes involved in both AD and PD. **(D)** Shared GO BP terms between AD and PD that are unique to broad cell type groups are shown. The inner circle denotes the cell type group from AD while the outer circle denotes the PD group. Darker shade represents terms enriched exclusively in neuron modules (excitatory and inhibitory neurons, CALB1, SOX6) and lighter shade represents terms enriched exclusively in glial modules (microglia, astrocyte, oligodendrocyte, OPC, endothelial cells). Biological processes related to altered cytoskeleton dynamics, impaired mitochondrial function, and apoptotic signaling are enriched across gene modules from neurons in both AD and PD. Immune response, synapse maintenance, and lipid transport-related terms are altered in one or more glial cell modules in both AD and PD.

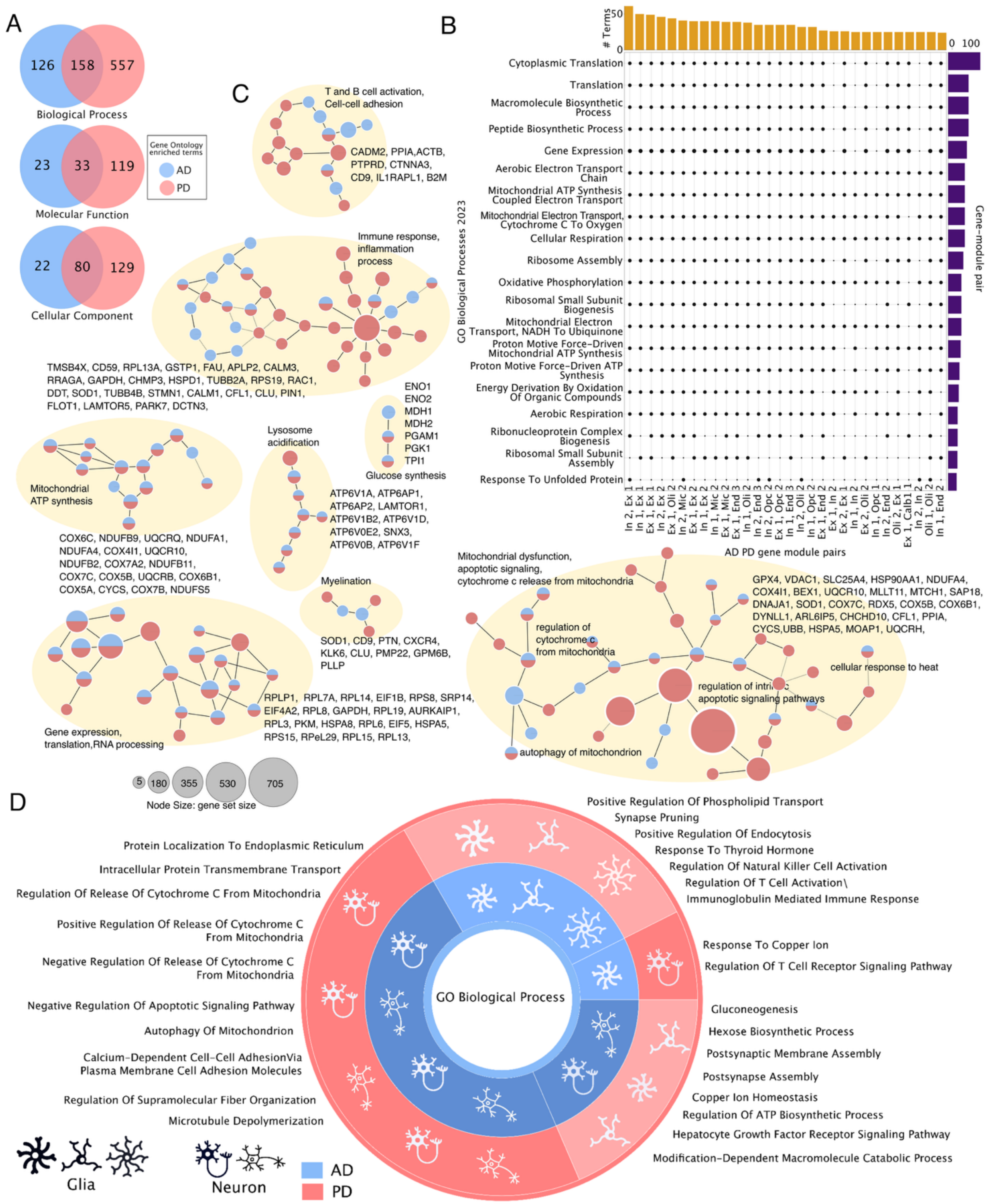

A
126
158
557
Biological Process
23
33
119
Molecular Function
22
80
129
Cellular Component
Gene Ontology enriched terms
AD
PD
B
Terms
50
0
0 100
Cytoplasmic Translation
Translation
Macromolecule Biosynthetic Process
Peptide Biosynthetic Process
Gene Expression
Aerobic Electron Transport Chain
Mitochondrial ATP Synthesis Coupled Electron Transport
Mitochondrial Electron Transport, Cytochrome C To Oxygen
Cellular Respiration
Ribosome Assembly
Oxidative Phosphorylation
Ribosomal Small Subunit Biogenesis
Mitochondrial Electron Transport, NADH To Ubiquinone
Proton Motive Force-Driven Mitochondrial ATP Synthesis
Proton Motive Force-Driven ATP Synthesis
Energy Derivation By Oxidation Of Organic Compounds
Aerobic Respiration
Ribonucleoprotein Complex Biogenesis
Ribosomal Small Subunit Assembly
Response To Unfolded Protein
GO Biological Processes 2023
Gene-module pair
AD PD gene module pairs
C
T and B cell activation, Cell-cell adhesion
CADM2, PPIA,ACTB, PTPRD, CTNNA3, CD9, IL1RAPL1, B2M
Immune response, inflammation process
TMSB4X, CD59, RPL13A, GSTP1, FAU, APLP2, CALM3, RRAGA, GAPDH, CHMP3, HSPD1, TUBB2A, RPS19, RAC1, DDT, SOD1, TUBB4B, STMN1, CALM1, CFL1, CLU, PIN1, FLOT1, LAMTOR5, PARK7, DCTN3,
ENO1 ENO2 MDH1 MDH2 PGAM1 PGK1 TPI1
Glucose synthesis
Lysosome acidification
ATP6V1A, ATP6AP1, ATP6AP2, LAMTOR1, ATP6V1B2, ATP6V1D, ATP6V0E2, SNX3, ATP6V0B, ATP6V1F
Mitochondrial ATP synthesis
COX6C, NDUFB9, UQCRQ, NDUFA1, NDUFA4, COX4I1, UQCR10, NDUFB2, COX7A2, NDUFB11, COX7C, COX5B, UQCRB, COX6B1, COX5A, CYCS, COX7B, NDUFS5
Myelination
SOD1, CD9, PTN, CXCR4, KLK6, CLU, PMP22, GPM6B, PLLP
RPLP1, RPL7A, RPL14, EIF1B, RPS8, SRP14, EIF4A2, RPL8, GAPDH, RPL19, AURKAIP1, RPL3, PKM, HSPA8, RPL6, EIF5, HSPA5, RPS15, RPeL29, RPL15, RPL13,
Gene expression, translation,RNA processing
Mitochondrial dysfunction, apoptotic signaling, cytochrome c release from mitochondria
GPX4, VDAC1, SLC25A4, HSP90AA1, NDUFA4, COX4I1, BEX1, UQCR10, MLLT11, MTCH1, SAP18, DNAJA1, SOD1, COX7C, RDX5, COX5B, COX6B1, DYNLL1, ARL6IP5, CHCHD10, CFL1, PPIA, CYCS,UBB, HSPA5, MOAP1, UQCRH,
regulation of cytochrome c from mitochondria
cellular response to heat
apoptotic signaling pathways
autophagy of mitochondrion
5
180
355
530
705
Node Size: gene set size
D
GO Biological Process
Positive Regulation Of Phospholipid Transport
Synapse Pruning
Positive Regulation Of Endocytosis
Response To Thyroid Hormone
Regulation Of Natural Killer Cell Activation
Regulation Of T Cell Activation\
Immunoglobulin Mediated Immune Response
Response To Copper Ion
Regulation Of T Cell Receptor Signaling Pathway
Gluconeogenesis
Hexose Biosynthetic Process
Postsynaptic Membrane Assembly
Postsynapse Assembly
Copper Ion Homeostasis
Regulation Of ATP Biosynthetic Process
Hepatocyte Growth Factor Receptor Signaling Pathway
Modification-Dependent Macromolecule Catabolic Process
Protein Localization To Endoplasmic Reticulum
Intracellular Protein Transmembrane Transport
Regulation Of Release Of Cytochrome C From Mitochondria
Positive Regulation Of Release Of Cytochrome C From Mitochondria
Negative Regulation Of Release Of Cytochrome C From Mitochondria
Negative Regulation Of Apoptotic Signaling Pathway
Autophagy Of Mitochondrion
Calcium-Dependent Cell-Cell AdhesionVia Plasma Membrane Cell Adhesion Molecules
Regulation Of Supramolecular Fiber Organization
Microtubule Depolymerization
Glia
Neuron
AD
PD

4. In the method section, about the 'Differential gene expression', it seems to me that the authors do not mention covariates adjustments when computing DEGs, so does authors consider any covariates for DEG? for example, sex or PMI?

Response:

We thank the reviewer for highlighting this important point. In our original analysis, we used the Wilcoxon rank sum test to identify differentially expressed genes (DEGs) using binary disease state (disease vs. control) as the outcome, without including covariates. The highest correlation observed was between oligodendrocytes and oligodendrocyte precursor cells (Kendall's tau-b = 0.2; statistically significant based on a label shuffle permutation test with 1000 iterations). Overall, we observed low correlations among DEGs that were identified in AD versus PD across cell type pairs.

However, most commonly used DEG pipelines include covariates such as age, sex, and postmortem interval. To align with current practices, we have recomputed the differential gene expression analysis using the MAST framework, which is specifically designed for single-cell and single-nucleus RNA-seq data. In the updated analysis, we included age, sex and post-mortem interval as covariates in the model to control for potential confounding effects. Differential expression was calculated separately within each cell type, independently for the AD and PD datasets.

The updated results and corresponding figure have been incorporated into the manuscript, and the Methods section has been revised accordingly. The revised text reads as follows:

Results:

"We examined the overlap between the adDEGs and pdDEGs across AD and PD cell types (Kendall's tau-b associations; Fig. 5; Methods). Across 54 pairwise comparisons (6 AD and 9 PD cell types), the highest significant association observed was 0.4, occurring between microglia adDEGs and pdDEGs (FDR q-value<0.05; p-values corrected for multiple comparisons). Notably, this highest association among all possible cell type pairings, was significantly lower than the maximum similarity observed from our gene module analysis (cf. Fig. 2A).

We then systematically tested the overall difference in mean correlations between AD-PD associations based on pairwise gene module τ_b from PLS (12 x 20) versus pairwise cell type τ_b from DGE (6 x 9). Using Welch's t-test, which accounts for unequal sample sizes, we observed a significant difference in correlation strengths between the two methods. Across pairwise comparisons, PLS_{cell} τ_b values were higher than DGE derived τ_b values (Welch's $t = 5.51$; *p*-value<0.001). This finding suggested that associations between AD-PD similarity were systematically stronger when using our gene module approach compared to the classical DGE method.

Next, we performed a gene set enrichment analysis of the differentially expressed genes using GO databases (GO BP, MF, and CC). Independently for each disease, we created our ranked gene list based on the fold change significance level (conditioned on cell type; Methods) and

used GSEA to identify significantly enriched terms (FDR $q<0.1$). We observed 7 common terms, in total, between AD and PD across all 3 GO databases (Fig. S6). Overall, these terms represented only a small subset of the broader set of shared AD-PD terms that were identified through our gene-module based analyses (213 GO terms). Notably, the shared terms emerging from DGE analysis (e.g., cytoplasmic translation) appeared among the most frequently recurring terms across gene modules from the PLS analysis (cf. Fig. 4C and Fig. S6), suggesting that DGE may primarily capture the strongest disease overlaps from the gene expression matrices."

Figure:

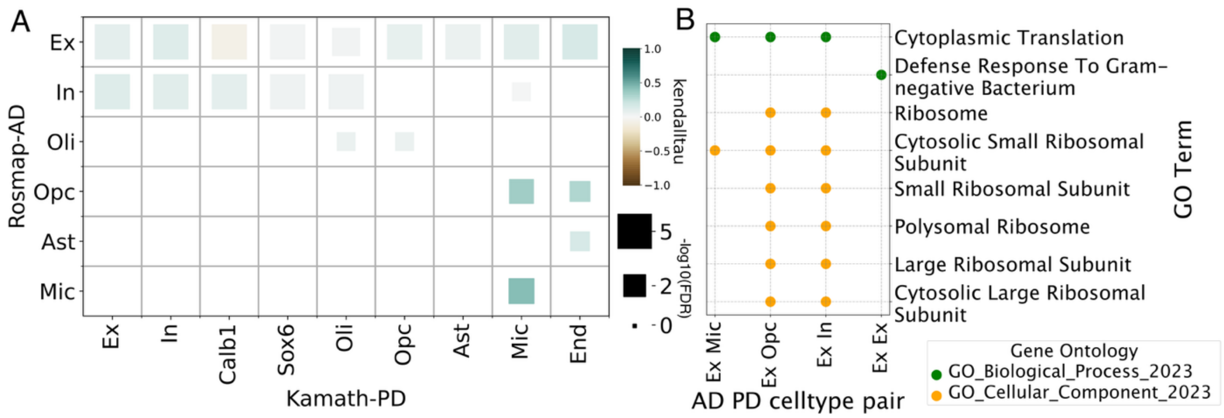

Figure 2. Differential expression analysis revealed modest similarity between AD- and PD-associated transcriptomic signatures. We benchmarked our latent factor model-derived AD-PD overlap with differential gene expression derived AD-PD overlap. (A) Pairwise associations between AD and PD differentially expressed genes are shown (Kendall's tau-b). For each cell type pair, statistical significance of association was assessed using a permutation test. Colored squares indicate significant associations (FDR < 0.05). Darker green (brown) denotes greater similarity (anti) between the log-fold change of significant DEGs from an AD-PD cell type pair. Square size is proportional to $-\log_{10}$(FDR). Compared to our PLS gene-module-based overlap analysis (Fig. 2A), significantly smaller correlations emerge from this univariate approach. The maximum correlation observed was 0.4 between AD and PD microglia. **(B)** Dots represent shared GO terms between AD and PD from gene set enrichment of DGEs. Significant terms in AD or PD from GSEA analysis were assessed (FDR $q<0.1$). In contrast to 213 shared terms across pairwise AD-PD gene modules (Fig. 4A), only 7 shared terms emerged between AD and PD from our DGE analysis. Ast, Astrocyte; Ex, Excitatory neuron; In, Inhibitory neuron; Mic, Microglia; Oli, Oligodendrocyte; Opc, Oligodendrocyte precursor cell; End, Endothelial.

Methods:

"Differential gene expression was performed using the MAST (v1.36.0), implemented in R and accessed from Python using rpy2. Within each dataset, log-normalized single-nucleus expression data were analyzed separately for each cell type. For each gene, a generalized linear hurdle model was fitted with diagnosis (disease vs. control) as the primary variable of interest. Cellular detection rates were included to account for differences in gene detection across cells. Age, post-mortem interval and sex were included as covariates to account for potential confounding effects.

Significance was assessed using likelihood ratio tests as implemented in MAST. Multiple testing correction was performed across all assessed genes using the Benjamini-Hochberg method (Benjamini and Hochberg 1995). Effect sizes were quantified using the estimated $\log_2$ fold change between disease and control groups. Genes meeting the statistical significance threshold after correction were designated as differentially expressed genes. The final DEGs were referred to as adDEGs for AD and pdDEGs for PD.

To estimate the pairwise association between cell-type-specific adDEGs and pdDEGs, we computed Kendall's tau-b using the log-fold change values of genes in the AD-PD AND conjunction set (see above). This resulted in a similarity matrix of dimension 6 x 9, corresponding to the number of AD (6) and PD (9) cell types. Statistical significance for each association was evaluated using a permutation test with 1000-iterations, in which the fold change values of overlapping genes were randomly shuffled. FDR correction was performed across all comparisons (54) using the Benjamini-Hochberg procedure."

Reviewer #2: This study presents an analysis of gene co-expression modules in Alzheimer's disease (AD) and Parkinson's disease (PD), utilizing a pattern learning framework predicated on single-nucleus RNA sequencing data. The research uncovered shared molecular mechanisms between the two diseases within neurons, microglia, oligodendrocytes, and astrocytes. These include cytoskeletal dynamics, mitochondrial stress responses, T cell activation processes, dysregulated myelin synthesis pathways, and abnormal heavy metal handling mechanisms. These findings illustrate common genetic underpinnings of AD and PD in specific cellular contexts, thereby providing novel insights into their pathological overlaps.

1. It is recommended that the author allocate a specific amount of space in the introduction section to elucidating the relationship between neurological disorders and other types of diseases.

2. The following references are closely related to the author's topic and are recommended for the author to cite.

[1] Wu, Yimao, Ruowei Sun, Shuai Ren, Gokhan Zengin, and Mengyao Li. 2025. " Neuronal Reshaping of the Tumor Microenvironment in Tumorigenesis and Metastasis: Bench to Clinic," Medicine Advances: 1-8. https://doi.org/10.1002/med4.70044.

[2] Lei, HY., Pi, GL., He, T. et al. Targeting vulnerable microcircuits in the ventral hippocampus of male transgenic mice to rescue Alzheimer-like social memory loss. Military Med Res 11, 16 (2024). https://doi.org/10.1186/s40779-024-00512-z

[3] Zhao, Yihang, Mengzhen Jia, Chen Ding, Bingkun Bao, Hangqi Li, Jiabin Ma, Weixuan Dong, et al. 2025. " Time-Restricted Feeding Mitigates Alzheimer's Disease-Associated Cognitive Impairments via a B. pseudolongum-Propionic Acid-FFAR3 Axis." iMeta 4, e70006. https://doi.org/10.1002/imt2.70006

Response:

We thank the reviewer for these helpful suggestions and references, which have helped improve our manuscript. Accordingly, we have revised the Introduction and discussion to incorporate the cited references. We have also added a paragraph discussing the relationships among neurological disorders.

"… These observations raise the possibility of disease mechanisms contributing to shared neuropathology between AD and PD at the molecular level (Armstrong, Lantos, and Cairns 2005; Perl, Warren, and Calne 1998).

More broadly, recent systems-level analysis further suggests that multiple neurodegenerative disorders, including AD and PD, share overlapping biological pathways and pathological mechanisms (Armstrong et al. 2005). Proposed mechanisms include mitochondrial dysfunction, neuroinflammation, and dysregulated protein homeostasis (Lei et al. 2024; Perl et al. 1998). Involvement of these processes have also been suggested in several other neurodegenerative disorders, reinforcing the view that neurodegenerative mechanisms may arise from partially overlapping manifestations of shared molecular networks rather than completely independent disease mechanisms (Barabási, Gulbahce, and Loscalzo 2011; Crow and Gillis 2018; Subramanian et al. 2005; Zhang and Horvath 2005).

… In reality, however, gene expression occurs within tightly regulated environments where gene products interact in highly combinatorial ways (Ferreira et al. 2018; Zhu et al. 2017)."

Updated Discussion:

"While the present study focuses on the computational identification of disease-relevant gene programs and a subsequent comparison between AD and PD, future experimental work using cellular systems or animal models targeting the highlighted modules and shared pathways will be necessary to further investigate AD-PD convergence in vivo. For example, recent animal research on gut-brain axis disruptions in AD identified common therapeutic intervention strategies that might be applicable across neurodegenerative disorders (Hou et al. 2021; Zhao et al. 2025)."

In addition, to further examine the disease specificity of the gene programs identified in our study, we performed systematic comparisons with a non-neurological disease, chronic obstructive pulmonary disease (COPD) of the lung. This comparison was intended to contrast gene programs derived from two biologically distinct tissues with markedly different cellular compositions (lung vs. brain).

We observed limited overlap between brain disease modules and lung cell populations overall. Notably, some overlap was detected between lung immune cells (myeloid and lymphoid cells) — our negative control — and brain glial cells (primarily microglia) — our cell population of scientific interest. In contrast, little to no overlap was observed across other lung cell populations, including stromal, epithelial and endothelial cells. These findings highlight that while certain immune-related mechanisms may be shared across diseases and tissues, the

majority of gene programs identified in our analysis appear to be specific to neurodegenerative contexts.

We have incorporated a paragraph in the Results section discussing the broader relationship between neurological and other systemic diseases.

"To assess the specificity of our findings from the AD-PD comparative analysis with respect to other systemic diseases, we performed a comparison of AD or PD derived modules with chronic obstructive pulmonary disease (COPD) of the lung (neurological versus non-neurological). COPD was selected as a negative control condition given the expectedly different cellular composition and tissue context relative to the brain. Following our established analytical pipeline, we computed pairwise similarities between AD- and PD-associated gene programs with COPD-associated gene programs across major recorded lung cell populations (AD-Lung, PD-Lung; derived following Fig.1 pipeline; table S5-S8).

Overall, we observed limited overlap between brain and lung disease modules. The modest similarities that were detected primarily involved immune related cell populations and endothelial cells from the lung and the brain. Specifically, neuronal populations from all 4 AD and PD datasets showed little overlap with the lung gene modules (maximum Mye 1_Ex 1 (Lung_Kamath-PD), τb, abs = 0.49, FDR q = 1.43e-181). In contrast to the neuronal cells from the brain, lung myeloid (Mye) and lymphoid (Lymp) cells showing significant overlap with brain glial cells (maximum Lymp 1_Mic 1 (Lung_Rosmap-AD), τb, abs= 0.74, FDR q = 6.4e-4). Taken together, these results suggest that neuronal gene programs identified in AD and PD are largely brain-specific, while a subset of immune-related signatures are shared across tissues and disorders."

References:

Hou, Y., Shan, C., Zhuang, S., Zhuang, Q., Ghosh, A., Zhu, K., Kong, X., Wang, S., Gong, Y., Yang, Y., Tao, B., Sun, L., Zhao, H.-Y., Guo, X., Wang, W., Ning, G., Gu, Y., Li, S., Liu, J., 2021. Gut microbiota-derived propionate mediates the neuroprotective effect of osteocalcin in a mouse model of Parkinson's disease. Microbiome 9, 34. https://doi.org/10.1186/s40168-020-00988-6

Lei, H.-Y., Pi, G.-L., He, T., Xiong, R., Lv, J.-R., Liu, J.-L., Wu, D.-Q., Li, M.-Z., Shi, K., Li, S.-H., Yu, N.-N., Gao, Y., Yu, H.-L., Wei, L.-Y., Wang, X., Zhou, Q.-Z., Zou, P.-L., Zhou, J.-Y., Liu, Y.-Z., Shen, N.-T., Yang, J., Ke, D., Wang, Q., Liu, G.-P., Yang, X.-F., Wang, J.-Z., Yang, Y., 2024. Targeting vulnerable microcircuits in the ventral hippocampus of male transgenic mice to rescue Alzheimer-like social memory loss. Military Med Res 11, 16. https://doi.org/10.1186/s40779-024-00512-z

Wu, Y., Sun, R., Ren, S., Zengin, G., Li, M., 2025. Neuronal Reshaping of the Tumor Microenvironment in Tumorigenesis and Metastasis: Bench to Clinic. Medicine Advances 3, 364–371. https://doi.org/10.1002/med4.70044

Zhao, Y., Jia, M., Ding, C., Bao, B., Li, H., Ma, J., Dong, W., Gao, R., Chen, X., Chen, J., Dai, X., Zou, Y., Hu, J., Shi, L., Liu, X., Liu, Z., 2025. Time-restricted feeding mitigates Alzheimer's

disease-associated cognitive impairments via a B. pseudolongum-propionic acid-FFAR3 axis. iMeta 4, e70006. https://doi.org/10.1002/imt2.70006

3. Detailed validation results using independent AD and PD datasets should be presented in the paper to enhance the credibility of the study.

Response:

We appreciate this suggestion and note that an explicit external validation using independent datasets has already been incorporated in our study. Specifically, an independent AD snRNA-seq dataset, Seattle-AD, and an independent PD snRNA-seq dataset, Smajić-PD, were used to validate our primary findings from Rosmap-AD and Kamath-PD datasets.

In response to this helpful feedback, to make the AD-PD validation results much more explicit, we have now added a dedicated paragraph early in the Results section highlighting the derivation of the gene modules specific to these external datasets.

"In a stringent external validation analysis in untouched datasets, we derived a full, independent set of AD and PD gene modules in two additional snRNA-seq datasets — Seattle-AD and Smajić-PD (detailed cohort and sample description in Methods). We repeated all main analyses, from scratch, and derived sub-cell-level disease predictive gene modules from these datasets (Fig. S3B; Methods). In Seattle-AD, 15 significant gene modules emerged across ten examined cell types. Independently, in Smajić-PD, 25 significant gene modules emerged across seven cell types. Significance of modules was determined, as before, using a label-shuffle permutation test. These independently derived gene modules were subsequently used to corroborate our findings from the primary AD-PD overlap analysis (see next section).

… To validate our primary findings, we examined the molecular similarity between AD and PD using the gene modules derived in Seattle-AD and Smajić-PD. Our analysis revealed significant gene module overlaps between the AD and PD, scattered across different cell types (Fig. S3A; table S2). Oligodendrocyte module pairs from AD and PD, once again, took center stage with strong associations ($q < 0.05$) between each other as well as with modules from neurons, astrocytes, and microglia. The strongest association was observed between oligodendrocytes from Seattle-AD and astrocytes from Smajić-PD (Oli 1_Oli 1, $\tau_{b, abs}$= 0.51, q = 5.5e-26) followed closely by Ast 1_Oli 1 ($\tau_{b, abs}$= 0.26, q = 9.2e-16). Strong significant associations were also observed between different combinations of neuron and glial cell-derived modules (Ast 1_L4_it 1, $\tau_{b, abs}$= 0.45, q = 1.6e-9; Oli 2_In 3, $\tau_{b, abs}$= 0.86; q = 0.05). As in the primary analysis, inhibitory neuron-derived modules showed sparse similarities between AD and PD. Overall, our external validation of shared genetic signatures between AD and PD replicated the primary findings, indicating that these are unlikely to be driven by dataset-specific factors such as transcriptomic platform, cohort composition, or brain region selection."

4. The Methods section should provide a more comprehensive description of the statistical methods used for batch effect correction and control of potential confounding factors.

Response:

We thank the reviewer for this suggestion and the opportunity to clarify our analytical framework.

We agree that single-cell analysis pipelines are highly sensitive to batch-effects, which can occlude true biological signals. These effects generally stem from two levels of variation: inter-experimental and intra-experimental. Intra-experimental effects, often arise from subtle shifts in environmental conditions, sample handling, or technical processing during a single study. Inter-experimental effects typically include much larger variance, driven by differences in sequencing platforms, distinct library protocols, and variations in experimental design across different laboratories.

Here, we carefully designed a workflow that avoids inter-experimental batch effects. We opted not to merge transcriptomic data (the expression matrices) across the different datasets during our gene module derivations. That is, gene modules were derived independently within each dataset, with separate hyperparameter optimization and model fitting regimes. For comparison between diseases (across datasets), we focussed exclusively on the model derived gene loadings in gene modules, rather than analyzing a merged expression matrix.

Consequently, potential batch effects are restricted to sample-level variability within each source dataset. Notably, the datasets used in this study were generated by the original authors using well-established single-nucleus preprocessing and batch-correction pipelines, as described in their respective high-impact publications. We utilized these expert-validated expression matrices in their provided form, maintaining the integrity of the original authors’ processing without introducing further computational adjustments.

To clarify and emphasize these points, we have expanded our Methods section to provide a detailed description of how batch effects are handled in our analysis framework.

“We relied on the preprocessed datasets from the authors responsible for the data collection (cf. above). This maximizes reproducibility and compatibility with other studies working with these resources. The transcriptomic datasets were processed in the source studies using standardized snRNA-seq processing pipelines. This included quality control for cell inclusion, including doublet detection, the removal of low-quality and outlier cells, the removal of lowly expressed genes, and sample-level batch correction procedures.

Crucially, the transcriptomic datasets from different studies were — at no point — merged or jointly integrated at any stage of the analysis. Instead, each dataset was analyzed independently to derive disease-associated gene modules. For each dataset, model fitting and hyperparameter optimization were performed separately. By avoiding cross-dataset integration, our analysis circumvented the need for batch-effect correction across datasets. Thus, comparisons between AD and PD were performed at the level of model derived gene loadings, not experimentally recorded gene expression, only after gene modules had been derived independently within each dataset.”

Details regarding the handling of potential confounding variables, including age, sex and post-mortem interval, in the differential gene expression analysis are provided in our response to Reviewer 1's comment above.

In our latent factor modelling analyses, postmortem interval was considered a potential confounding factor and regressed out from the expression datasets. This is detailed in the current Methods section.

"In an additional data cleaning step, postmortem interval (PMI) was included as a covariate to account for potential confounding effects on gene expression. PMI has been shown to be a potential source of confounding elsewhere (Diamond 2024; Jaunmuktane and Brandner 2020; Kaufman et al. 2016; Rauch, Olson, and Gestwicki 2017; Walker and Jucker 2024). Specifically, here we regressed out the variation in gene expression attributable to differences in PMI. The resulting adjusted, cleaned and standardized transcriptomic profiles for each examined cell were used for subsequent steps in our modelling pipeline."

Other usual covariates including age and sex were not regressed out during the modelling steps. These variables can represent biologically meaningful sources of variation in neurodegenerative disease and may be intrinsically associated with disease progression or pathology. Removing these effects during preprocessing could therefore distort relevant biological signals.

In response to the reviewer's comment, we have assessed the potential influence of these variables by estimating their possible role on the model-derived component scores for each cell type. These assessments were performed using regression-based models and the resulting effect sizes are reported in Supplementary table 10. This approach allows for transparency of the degree of contribution of age and sex for future readers of our manuscript, while preserving the original biological variation in the data used for gene module discovery.

We have updated the Results section to include these findings.

"To assess whether latent gene modules were influenced by potential demographic variables, we examined the contribution of age and sex to variation in module scores, quantified as the proportion of variance explained by each variable in a linear regression model (Methods). Across gene modules, the contribution of sex towards explaining the variance in module scores was low within each dataset (mean ± standard deviation across modules): Rosmap-AD, R^2 = 0.01±0.01; Kamath-PD, R^2 = 0.07±0.09; Seattle-AD, R^2 = 0.017±0.018; Smajić-PD, R^2 = 0.12±0.10. Similarly, age explained little variance in module scores: Romsap-AD, R^2 = 0.003±0.006; Kamath-PD, R^2 = 0.04±0.08; Seattle-AD, R^2 = 0.018±0.020; Smajić-PD, R^2 = 0.04±0.06. Overall, across all gene modules from the four datasets, age and sex explained only a small fraction of the variance in module scores relative to the variance captured by diagnosis, the primary variable of interest (table S10)."

In addition, the updated Methods section now reads as follows.

"To evaluate the potential influence of demographic confounding factors, specifically age and sex, we modelled the per-nucleus component scores for each latent gene module as a function of diagnosis, age, and sex. For each gene module, component scores were regressed on diagnosis (primary variable of interest) together with age and sex (potential confounders) using ordinary

least squares regression from the python *statsmodel* package (v0.14.4). Following model fitting, an analysis of variance was performed to quantify the proportion of variance in component scores (R^2) that was explained by each predictor (anova_lm, typ=2; *statsmodel* v0.14.4). In total, 72 gene modules were analyzed (12 Rosmap-AD, 20 Kamath-PD, 25 Seattle-AD, and 15 Smajić-PD). This step provided a unique estimate of the contribution by each predictor while accounting for other variables in the model."

References

Ferreira, P.G., Muñoz-Aguirre, M., Reverter, F., Sá Godinho, C.P., Sousa, A., Amadoz, A., Sodaei, R., Hidalgo, M.R., Pervouchine, D., Carbonell-Caballero, J., Nurtdinov, R., Breschi, A., Amador, R., Oliveira, P., Çubuk, C., Curado, J., Aguet, F., Oliveira, C., Dopazo, J., Sammeth, M., Ardlie, K.G., Guigó, R., 2018. The effects of death and post-mortem cold ischemia on human tissue transcriptomes. Nat Commun 9, 490. https://doi.org/10.1038/s41467-017-02772-x

Zhu, Y., Wang, L., Yin, Y., Yang, E., 2017. Systematic analysis of gene expression patterns associated with postmortem interval in human tissues. Sci Rep 7, 5435. https://doi.org/10.1038/s41598-017-05882-0

5. To strengthen the biological relevance of the findings, functional experiments such as in vitro cell assays or animal models should be performed to validate the roles of identified key gene modules in the pathogenesis of AD and PD.

Response:

We thank the reviewer for this valuable suggestion. We agree that functional validation using in vitro or in vivo models would provide additional biological insight into the roles of the identified gene modules in the pathogenesis of AD and PD.

However, the primary aim of this study is to develop and apply a computational framework to compare cross-disease-associated gene programs from large-scale snRNA-seq datasets. As a computational study, our focus was on statistical robustness and in-silico replication of disease-associated gene module-level overlap. To support the biological relevance of our findings, we implemented several validation strategies, including cross-dataset comparisons of implicated biological pathways and cross-referencing with evidence previously reported in animal and in vivo studies.

Experimental validation of the predicted gene modules represents an important next step and could be pursued in future collaborative studies using cellular or animal models. We have added a statement in the Discussion to acknowledge this limitation and to highlight the potential for future experimental investigation of the identified modules.

The extended discussion now reads as follows.

“Several limitations should be considered when interpreting our findings. While the present study focuses on the computational identification of disease-relevant gene programs and a subsequent comparison between AD and PD, future experimental work using cellular systems or animal models targeting the highlighted modules and shared pathways will be necessary to further investigate AD-PD convergence in vivo. For example, recent animal research on gut-brain axis disruptions in AD identified common therapeutic intervention strategies that might be applicable across neurodegenerative disorders (Hou et al. 2021; Zhao et al. 2025). In addition, although the datasets analyzed in this study are among the largest currently available, larger and balanced cohorts will likely enable more robust estimation of gene modules, thus providing more granular insights into sex- and cell type-specific transcriptional basis of AD-PD overlap.”

6. The discussion section should further elaborate on the clinical implications of these shared molecular mechanisms, including their potential as therapeutic targets or biomarkers.

Response:

We thank the reviewer for this helpful suggestion to improve our manuscript. In response, we have expanded the following paragraphs in the Discussion section.

“The involvement of the MAPT gene in disease-relevant neuron modules from both AD and PD was noteworthy. This gene, widely implicated in tauopathy, encodes for the protein tau and is responsible for stabilizing axon microtubules. In its aggregated form, tau demonstrates prion-like behavior, passing from neuron to neuron across synapses, a mechanism increasingly recognized in both AD and PD (Diamond 2024; Jaunmuktane and Brandner 2020; Kaufman et al. 2016; Rauch, Olson, and Gestwicki 2017; Walker and Jucker 2024). In our analysis, the emergence of MAPT across both disorders highlights tau as a shared therapeutic target, motivating cross-disease therapeutic strategies aimed at limiting pathological protein spread and neuronal death.

… However, a key distinction emerged between the copper handling gene modules — glial modules included genes related to buffering/detoxification responses (via MT1E, MT2A, MT3, and APP), whereas excitatory neuron modules uniquely enriched copper-binding genes tied to oxidative stress and proteostasis (via critical antioxidative enzymes (SOD1, PARK), SNCA, and the copper chaperone protein (ATOX1)). Prior in-silico analyses of microarray data on brain tissue had reported a similar grouping of copper-handling genes (metallothionein group and the enzyme binding group)(Myhre et al. 2013). Therapeutically, these findings support cell type-targeted interventions; for example, enhancing glial copper-buffering capacity (e.g., boosting metallothionein pathways) to stabilize extracellular redox balance, while simultaneously protecting neurons with copper-modulating and antioxidant strategies (e.g., targeting SOD1- or ATOX1-lined pathways) to reduce ROS -driven proteotoxicity. Thus, our cellular module-based approach provides important clues for precise treatment design.

… Future work can expand our study to include a greater diversity of neurodegenerative, neurodevelopmental, and psychiatric diseases. In addition, the cell type specific genes identified within converging AD-PD associated modules represent potential therapeutic targets that warrant further investigation. Moreover, applying a similar computational framework to different, readily accessible source transcriptomes, like blood or cerebrospinal fluid, can identify critical biomarkers for neurodegeneration.”

References:

Zhao, Y., Jia, M., Ding, C., Bao, B., Li, H., Ma, J., Dong, W., Gao, R., Chen, X., Chen, J., Dai, X., Zou, Y., Hu, J., Shi, L., Liu, X., Liu, Z., 2025. Time-restricted feeding mitigates Alzheimer’s disease-associated cognitive impairments via a B. pseudolongum-propionic acid-FFAR3 axis. iMeta 4, e70006. https://doi.org/10.1002/imt2.70006

Myhre, O., Utkilen, H., Duale, N., Brunborg, G., Hofer, T., 2013. Metal Dyshomeostasis and Inflammation in Alzheimer’s and Parkinson’s Diseases: Possible Impact of Environmental Exposures. Oxid Med Cell Longev 2013, 726954. https://doi.org/10.1155/2013/726954

Rauch, J.N., Olson, S.H., Gestwicki, J.E., 2017. Interactions between Microtubule-Associated Protein Tau (MAPT) and Small Molecules. Cold Spring Harb Perspect Med 7, a024034. https://doi.org/10.1101/cshperspect.a024034
