# Supplementary material for: Cell type transcriptomic modules reveal shared molecular mechanisms in Alzheimer’s and Parkinson’s disease
Source: Gigascience. 2026 May 21;15:giag059. doi: 10.1093/gigascience/giag059 (PMC13289754; doi:10.1093/gigascience/giag059)
Supplement: giag059_Reviewer_1_Report_original_submission — Reviewer 1 -- 11/9/2025 [file giag059_reviewer_1_report_original_submission.pdf]

Reviewer Report

Title: Cell type transcriptomic modules reveal shared molecular mechanisms in Alzheimer's and Parkinson's disease

Version: Original Submission **Date:** 11/9/2025

Reviewer name: Reviewer 1

Reviewer Comments to Author:

The authors utilized single-nuclei RNA-seq data and GWAS data to study the disease comorbidity between Alzheimer's disease and Parkinson's disease. They also validated their discoveries using independent evaluation cohorts. This is an overall well-conducted and comprehensive study with a solid logical framework and clear presentation of very interesting biological results. I have few points that need clarifications.

1.In section 'Common Mechanisms of microglia's involvement in AD and PD', lines 790-792, the authors mentioned that 'we noted T cell activation in both AD and PD (all 4 datasets).' This is very interesting biological discovery. Based on my knowledge, the amount of T cells extracted from brain snRNA-seq are quite small (I could be wrong). Can authors explain something that could prove the conclusion is not biased due to the small population of T cells?

2. The authors mentioned using PLS-DA method to extract disease modules for AD and PD datasets respectively. if I understand correctly, that the authors apply PLS-DA method to snRNA-seq data instead of for example pseudobulk aggregated for each cell type. Can the authors explain and add some logics in the method section to further clarify why applying PLS-DA to snRNA-seq make sense? As we know snRNA-seq data are quite sparse.

3. In Figure 4D, authors presented multiple gene modules, can the authors also add gene/protein symbols as well, not just circles? Readers can know directly what are related proteins.

4. In the method section, about the 'Differential gene expression', it seems to me that the authors do not mention covariates adjustments when computing DEGs, so does authors consider any covariates for DEG? for example, sex or PMI?

Methods

Are the methods appropriate to the aims of the study, are they well described, and are necessary controls included? Choose an item.

Conclusions

Are the conclusions adequately supported by the data shown? Choose an item.

Reporting Standards

Does the manuscript adhere to the journal's guidelines on minimum standards of reporting? Choose an item.

Choose an item.

Statistics

Are you able to assess all statistics in the manuscript, including the appropriateness of statistical tests used? Choose an item.

Quality of Written English

Please indicate the quality of language in the manuscript: Choose an item.

Declaration of Competing Interests

Please complete a declaration of competing interests, considering the following questions:

- Have you in the past five years received reimbursements, fees, funding, or salary from an organisation that may in any way gain or lose financially from the publication of this manuscript, either now or in the future?
- Do you hold any stocks or shares in an organisation that may in any way gain or lose financially from the publication of this manuscript, either now or in the future?
- Do you hold or are you currently applying for any patents relating to the content of the manuscript?
- Have you received reimbursements, fees, funding, or salary from an organization that holds or has applied for patents relating to the content of the manuscript?
- Do you have any other financial competing interests?
- Do you have any non-financial competing interests in relation to this paper?

If you can answer no to all of the above, write 'I declare that I have no competing interests' below. If your reply is yes to any, please give details below.

I declare that I have no competing interests

I agree to the open peer review policy of the journal. I understand that my name will be included on my report to the authors and, if the manuscript is accepted for publication, my named report including any attachments I upload will be posted on the website along with the authors' responses. I agree for my report to be made available under an Open Access Creative Commons CC-BY license (http://creativecommons.org/licenses/by/4.0/). I understand that any comments which I do not wish to be included in my named report can be included as confidential comments to the editors, which will not be published.

Choose an item.

To further support our reviewers, we have joined with Publons, where you can gain additional credit to further highlight your hard work (see: https://publons.com/journal/530/gigascience). On publication of this paper, your review will be automatically added to Publons, you can then choose whether or not to claim your Publons credit. I understand this statement.

Yes Choose an item.
