# Supplementary material for: Cell type transcriptomic modules reveal shared molecular mechanisms in Alzheimer’s and Parkinson’s disease
Source: Gigascience. 2026 May 21;15:giag059. doi: 10.1093/gigascience/giag059 (PMC13289754; doi:10.1093/gigascience/giag059)
Supplement: giag059_Reviewer_2_Report_original_submission — Reviewer 2 -- 12/29/2025 [file giag059_reviewer_2_report_original_submission.pdf]

Reviewer Report

Title: Cell type transcriptomic modules reveal shared molecular mechanisms in Alzheimer's and Parkinson's disease

Version: Original Submission **Date:** 12/29/2025

Reviewer name: Reviewer 2

Reviewer Comments to Author:

This study presents an analysis of gene co-expression modules in Alzheimer's disease (AD) and Parkinson's disease (PD), utilizing a pattern learning framework predicated on single-nucleus RNA sequencing data. The research uncovered shared molecular mechanisms between the two diseases within neurons, microglia, oligodendrocytes, and astrocytes. These include cytoskeletal dynamics, mitochondrial stress responses, T cell activation processes, dysregulated myelin synthesis pathways, and abnormal heavy metal handling mechanisms. These findings illustrate common genetic underpinnings of AD and PD in specific cellular contexts, thereby providing novel insights into their pathological overlaps.

1. It is recommended that the author allocate a specific amount of space in the introduction section to elucidating the relationship between neurological disorders and other types of diseases.
2. The following references are closely related to the author's topic and are recommended for the author to cite.
[1] Wu, Yimao, Ruowei Sun, Shuai Ren, Gokhan Zengin, and Mengyao Li. 2025. " Neuronal Reshaping of the Tumor Microenvironment in Tumorigenesis and Metastasis: Bench to Clinic," Medicine Advances: 1-8. https://doi.org/10.1002/med4.70044.
[2] Lei, HY., Pi, GL., He, T. et al. Targeting vulnerable microcircuits in the ventral hippocampus of male transgenic mice to rescue Alzheimer-like social memory loss. Military Med Res 11, 16 (2024). https://doi.org/10.1186/s40779-024-00512-z
[3] Zhao, Yihang, Mengzhen Jia, Chen Ding, Bingkun Bao, Hangqi Li, Jiabin Ma, Weixuan Dong, et al. 2025. " Time-Restricted Feeding Mitigates Alzheimer's Disease-Associated Cognitive Impairments via a B. pseudolongum-Propionic Acid-FFAR3 Axis." iMeta 4, e70006. https://doi.org/10.1002/imt2.70006
3. Detailed validation results using independent AD and PD datasets should be presented in the paper to enhance the credibility of the study.
4. The Methods section should provide a more comprehensive description of the statistical methods used for batch effect correction and control of potential confounding factors.
5. To strengthen the biological relevance of the findings, functional experiments such as in vitro cell assays or animal models should be performed to validate the roles of identified key gene modules in the pathogenesis of AD and PD.
6. The discussion section should further elaborate on the clinical implications of these shared molecular mechanisms, including their potential as therapeutic targets or biomarkers.

Methods

Are the methods appropriate to the aims of the study, are they well described, and are necessary controls included? Choose an item.

Conclusions

Are the conclusions adequately supported by the data shown? Choose an item.

Reporting Standards

Does the manuscript adhere to the journal's guidelines on minimum standards of reporting? Choose an item.

Choose an item.

Statistics

Are you able to assess all statistics in the manuscript, including the appropriateness of statistical tests used? Choose an item.

Quality of Written English

Please indicate the quality of language in the manuscript: Choose an item.

Declaration of Competing Interests

Please complete a declaration of competing interests, considering the following questions:

- Have you in the past five years received reimbursements, fees, funding, or salary from an organisation that may in any way gain or lose financially from the publication of this manuscript, either now or in the future?
- Do you hold any stocks or shares in an organisation that may in any way gain or lose financially from the publication of this manuscript, either now or in the future?
- Do you hold or are you currently applying for any patents relating to the content of the manuscript?
- Have you received reimbursements, fees, funding, or salary from an organization that holds or has applied for patents relating to the content of the manuscript?
- Do you have any other financial competing interests?
- Do you have any non-financial competing interests in relation to this paper?

If you can answer no to all of the above, write 'I declare that I have no competing interests' below. If your reply is yes to any, please give details below.

I declare that I have no competing interests.

I agree to the open peer review policy of the journal. I understand that my name will be included on my report to the authors and, if the manuscript is accepted for publication, my named report including any attachments I upload will be posted on the website along with the authors' responses. I agree for my report to be made available under an Open Access Creative Commons CC-BY license (http://creativecommons.org/licenses/by/4.0/). I understand that any comments which I do not wish to be included in my named report can be included as confidential comments to the editors, which will not be published.

Choose an item.

To further support our reviewers, we have joined with Publons, where you can gain additional credit to further highlight your hard work (see: https://publons.com/journal/530/gigascience). On publication of this paper, your review will be automatically added to Publons, you can then choose whether or not to claim your Publons credit. I understand this statement.

Yes Choose an item.
